# Derivatization of Abietane Acids by Peptide-like Substituents Leads to Submicromolar Cytotoxicity at NCI-60 Panel

**DOI:** 10.3390/molecules29153532

**Published:** 2024-07-27

**Authors:** Elena Tretyakova, Anna Smirnova, Denis Babkov, Oxana Kazakova

**Affiliations:** 1 Ufa Institute of Chemistry of the Ufa Federal Research Centre of the Russian Academy of Sciences, 71 Prospect Oktyabrya, 450054 Ufa, Russia; bazunova03@yandex.ru (A.S.); obf@anrb.ru (O.K.); 2 Scientific Center for Innovative Drugs, Volgograd State Medical University, Novorossiyskaya St. 39, 400087 Volgograd, Russia; denis.a.babkov@gmail.com

**Keywords:** diterpenoids, dehydroabietic acid, levopimaric acid, diene adducts, Ugi reaction, Passerini reaction, NCI-60, anticancer activity

## Abstract

Natural compounds, including diterpenoids, play a critical role in various biological processes and are recognized as valuable components in cancer treatment. Isocyanides multicomponent reactions (IsMCRs) are one of the effective methods to obtain adducts at the carboxyl group with a peptide-like substituent. In this study, dehydroabietic acid and levopimaric acid diene adducts as the starting scaffolds were modified by the multicomponent Passerini (P-3CR) and Ugi (U-4CR) reactions to afford α-acyloxycarboxamides and α-acylaminocarboxamides. A group of twenty novel diterpene hybrids was subjected to NCI in vitro assessment, and a consistent structure–activity relationship was established. Eleven of the synthesized derivatives inhibited the growth of cancer cells of 4 to 39 cell lines in one dose assay, and the most active were derivatives **3d**, **9d**, and **10d** holding a fragment of 1a,4a-dehydroquinopimaric acid. They were selected for a five-dose analysis and demonstrated a significant antiproliferative effect towards human cancer cell lines. The outstanding cytotoxic activity was observed for the P-3CR product **3d** with growth inhibitory at submicromolar and micromolar concentrations (GI_50_ = 0.42–3 μM) against the most sensitive cell lines. The U-4CR products **9d** and **10d** showed selective activity against all leukemia cell lines with GI_50_ in the range of 1–17 µM and selectivity indexes of 5.49 and 4.72, respectively. Matrix COMPARE analysis using the GI_50_ vector showed a moderate positive correlation of compound **3d** with standard anticancer agents that can influence kinase receptors and epidermal growth factor receptors (EGFRs). The ADMET analysis acknowledges the favorable prognosis using compounds as potential anticancer agents. The obtained results indicate that these new hybrids could be useful for the further development of anticancer drugs, and 1a,4a-dehydroquinopimaric acid derivatives could be recommended for in-depth studies and the synthesis of new antitumor analogs on their basis.

## 1. Introduction

Cancer remains a global health problem and a leading cause of death worldwide despite significant advances in medicinal chemistry research that have led to the development of various anticancer treatment options [1]. Since a large number of mutagenic processes allow cancer cells to acquire the properties of unlimited proliferation potential and resistance, the synthetic development of potential anticancer agents with low side effects is highly desirable to combat this deadly disease [2,3]. In recent years, modern clinical oncology has increasingly used effective cytostatics based on natural compounds, the action of which is aimed at new cellular targets to solve the problem of multidrug tumor resistance [4,5,6]. A targeted search for new cytostatic agents involves, first of all, the development of molecules that can actively influence the pathological cells of the body and their growth. One approach in the development process is to enhance the therapeutic potential of natural compounds through structural modifications [7,8,9].

Multicomponent reactions (MCRs) have become necessary effective methods for the formation of new structural bonds in the synthesis of a wide range of pharmacologically active derivatives of organic and natural compounds without the separation and purification of intermediates. The most well-known isocyanide-based MCRs are the three-component Passerini reaction (P-3CR) and the four-component Ugi reaction (U-4CR), which are driven by the conversion of a high-energy formal divalent isocyano-carbon into a tetravalent amide carbonyl carbon [10,11]. As a result of the interaction of isocyanides, aldehydes/ketones and carboxylic acids (P-3CR) or isocyanides, aldehydes/ketones, carboxylic acids and amines (U-4CR), the corresponding α-acyloxycarboxamides and α-acylaminocarboxamides are formed. Moreover, the combination of natural compounds and their derivatives via isocyanide-based multicomponent reactions is attracting widespread interest due to the rapid access to the large libraries of diversity-oriented products with promising pharmacological potential [12,13,14]. Thus, there are several examples of the synthesis of U-4CR и P-3CR products based on triterpenoids, in particular glycyrrhizin, glycyrrhetinic, and masticadienonic acids [15,16]. A set of 34 new α-acylamino- and α-acyloxy-oleanolic and maslinic derivatives was successfully synthesized, and the cytotoxic activity evaluation revealed their significant activity against A2780 ovarian carcinoma cells in the low micromolar concentration range and the ability to induce programmed cell death partially through the apoptosis pathway [17]. Using the U-4CR approach, a diverse library of betulinic acid, fusidic acid, and cholic acid conjugates with TEMPO (nitroxide) was obtained, with cytotoxic effects against PC3 prostate cancer cell lines (IC_50_ 6.0–7.4 μM) and HT29 colon cancer cell lines (IC_50_ 7.0–9.0 µM). Studies of the mechanism of action have shown that they are targeted to mitochondria, and the induction of apoptosis occurs due to the activation of the caspase pathway [18].

**Figure 1:**
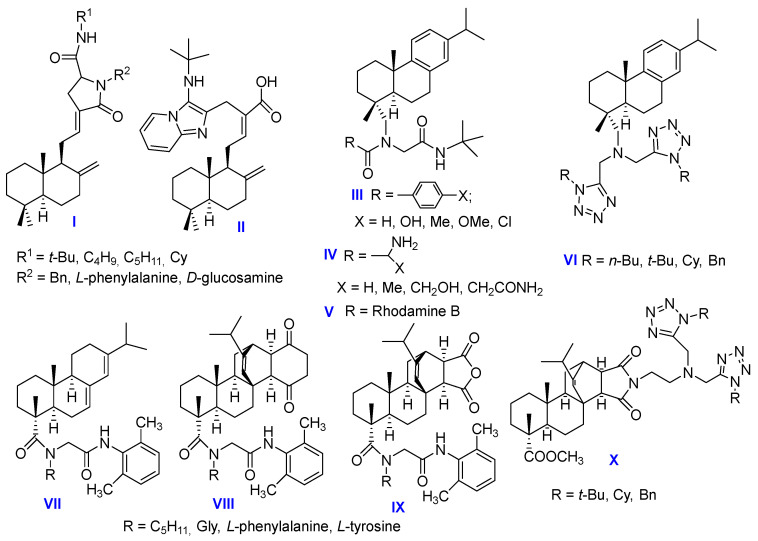
The known U-4CR products of diterpene acids.

There are even fewer examples in the literature of the use of diterpenoids, including the abietane series, in U-4CR and P-3CR (Figure 1). Based on the labdane diterpenoid coronarin D, as a result of the U-4CR with *L*-phenylalanine, 2-aminopyridine, *D*-glucosamine and *tert*-butyl isocyanide, non-toxic derivatives **I** and **II** were synthesized which can reduce LPS-stimulated NO production in RAW cells on a par with the standard anti-inflammatory drug dexamethasone [19]. From dehydroabiethylamine as the amine component in U-4CR, new α-acylaminocarboxamides **III–V** were synthesized and in vitro cytotoxicity was studied against several human tumor cell lines. Rhodamine B conjugate **V** has been found to exhibit cytotoxic activity characterized by EC_50_ values in the low nanomolar range [20]. Dehydroabiethylamine was also used in the pseudo-seven-component Ugi reaction for the synthesis of *bis*-tetrazoles **VI**, which demonstrated significant inhibition of the enzymes acetylcholinesterase and butyrylcholinesterase in Ellman assays [21]. In our laboratory, based on the abietic acid and diene adducts of levopimaric acid using U-4CR, a number of diterpene dipeptides **VII–IX** have been synthesized that effectively inhibit the influenza A (H1N1) virus (IC_50_ 2–32 μM, CC_50_ > 300 μM, SI 200) and are active against the pseudovirus SARS-CoV-2 [22]. Moreover, we recently reported that two diterpene U-4CR products obtained from the reaction of maleopimaric and dihydroquinopimaric acids with paraformaldehyde, benzylamine and ethyl 2-isocyanoacetate effectively inhibit leukemia, colon cancer, ovarian cancer, breast cancer and melanoma cell lines [23]. In addition, it was found that the azido-Ugi reaction of methyl maleopimarate amido-imide with isocyanides, paraformaldehyde and trimethylsilyl azide in one step leads to *bis*-1,5-disubstituted diterpene tetrazoles **X** with a selective cytotoxicity against NCI-60 cancer cell panels [24].

Taking into account the results obtained, here we have synthesized a library of twenty new resin acid derivatives using multicomponent Ugi and Passerini reactions and studied the cytotoxic potential of the synthesized products by high-throughput screening on 60 cell lines of nine different types of human cancer. Based on the screening results, new pharmacologically active derivatives were identified, and some patterns of pharmacological activity dependence on the substituent’s nature and the compound’s chemical structure were established.

## 2. Results and Discussion

### 2.1. Chemistry

**Figure 2:**
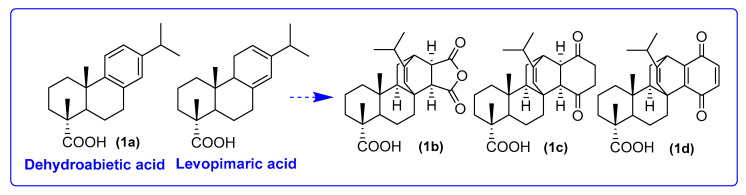
Diterpene carboxylic acids for MCRs in this research.

We employed native dehydroabietic acid **1a** and semi-synthetic derivatives of levopimaric diene adducts **1b**,**c**,**d** as acid components in U-4CR and P-3CR. The most well-known and practically valuable products of the diene synthesis reaction are the adducts of levopimaric acid with maleic anhydride (maleopimaric acid **1b**) and quinones. Quinopimaric acid, an adduct of levopimaric acid with *p*-benzoquinone, due to its easy ability for photocyclization, is most often used for further modifications in the form of its derivatives obtained as a result of reduction or oxidation reactions (2,3-dihydroquinopimaric acid **1c** and 1a,4a -dehydroquinopimaric acid **1d**) (Figure 2) [25].

**Scheme 1:**
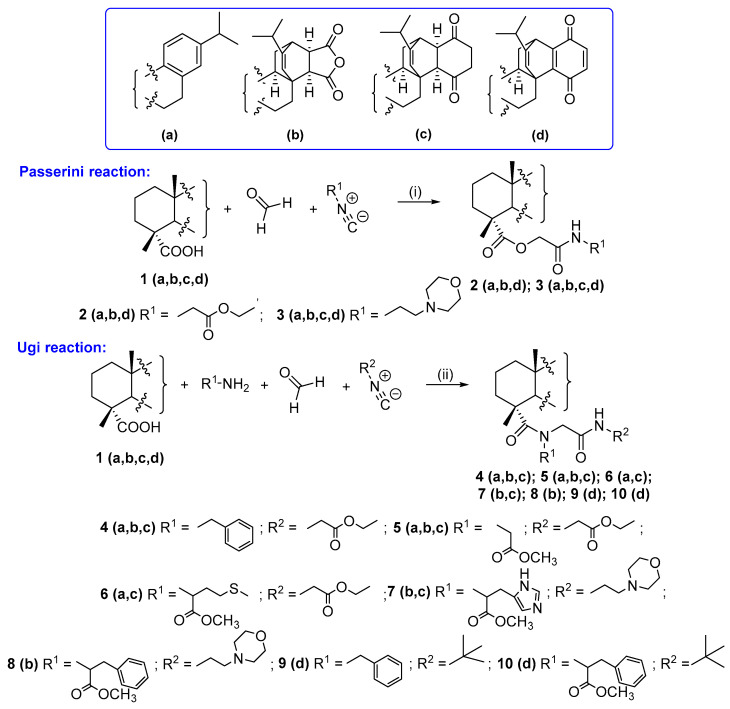
Conditions and reagents: P-3CR: ethyl 2-isocyanoacetate for **2a**,**b**,**d**; 2-morpholinoethyl isocyanate for **3a**,**b**,**c**,**d**; MeOH, rt, 3–4 days. Ugi 4CR: benzylamine and ethyl 2-isocyanoacetate for **4a**,**b**,**c**; glycine methyl ester hydrochloride and ethyl 2-isocyanoacetate for **5a**,**b**,**c**; *L*-methionine methyl ester hydrochloride and ethyl 2-isocyanoacetate for **6a**,**c**; *L*-histidine methyl ester hydrochloride and 2-morpholinoethyl isocyanate for **7b**,**c**; *L*-phenylalanine methyl ester hydrochloride and 2-morpholinoethyl isocyanate for **8b**; benzylamine and *tert*-butyl isocyanide for **9d**; and *L*-phenylalanine methyl ester hydrochloride and *tert*-butyl isocyanide for **10d**, MeOH, rt, 5–7 days.

For the synthesis of the first series of compounds, we exploited the P-3CR, which allows for the rapid and versatile generation of new diterpene derivatives displaying different α-acyloxyamides in the side chain. As it is known, this approach consists of the condensation of a carbonyl compound (aldehyde or ketone), isocyanides and carboxylic acid. With carboxylic acids **1a**,**b**,**c**,**d** in our hands, we performed P-3CR with ethyl 2-isocyanoacetate and 2-morpholinoethyl isocyanate as the isocyanide component and paraformaldehyde as the highly reactive carbonyl partner in methanol for 3–4 days by monitoring the progress of reactions with thin-layer chromatography. It should be mentioned that paraformaldehyde was employed as the oxo-component to avoid stereoisomer formation during the conjugation process. As a result, a series of acyloxycarboxamides of dehydroabietic acid **2a**,**3a**, maleopimaric acid **2b**,**3b**, 2,3-dihydroquinopimaric acid **3c** and 1a,4a-dehydroquinopimaric acid **2d**,**3d** were obtained in 75–82% yields after purification by column chromatography (Scheme 1).

The second set of products was obtained by U-4CR, which included the conversion of a mixture of four components (amine, isocyanide, aldehyde/ketone and carboxylic acid) to α-acetoamidocarboxamides or a peptide-like scaffold. Benzylamine and methyl esters of *L*-amino acids (glycine, *L*-methionine, *L*-histidine and *L*-phenylalanine) were used as the amine equivalent, and ethyl 2-isocyanoacetate, 2-morpholinoethyl isocyanate or tert-butyl isocyanide was the source of isocyanide. Ugi condensation was carried out in methanol at room temperature for 5–7 days using equimolar concentrations of the components. The yields of the products varied in the range of 66–85% depending on the nature of the substituents at the nitrogen atom. As a result, a group of derivatives of dehydroabietic acid **4a–6a**, maleopimaric acid **4b**, **5b**, **7b**, **8b**, 2,3-dihydroquinopimaric acid **4c–7c** and 1a,4a-dehydroquinopimaric acid **9d**, **10d** was obtained (Scheme 1).

The complete identification of the structure of compounds **2–10** was made by means of NMR spectroscopy and mass spectra. The mass spectra of **2–10** exhibited molecular ion peaks corresponding to the molecular masses of the compounds. The ^1^H NMR spectra of the P-3CR and U-4CR products NH- atom signals as broadened signals at δ 5.55–9.15 ppm were contained. In the ^13^C NMR spectra, the CONH- carbon atom signals appeared at the δ 169.6–178.4 ppm, and for U-4CR products **4–10**, CON- signals were also detected in the range of δ 172.8–183.9 ppm. The carbon signals of the dipeptide chain terminal amino acid keto groups in compounds like **5–8**, **10** at δ 167.4–171.1 ppm were observed while the aromatic proton δ signals of the benzylamine substituent and *L*-phenylalanine proton atom signals resonated at δ 7.10–7.45 ppm. The characteristic signals of the amino acid methyl group protons and *tert*-butyl methyl atom protons appeared as singlets at δ 3.65–3.85 and δ 1.25 ppm, respectively (Supplemental Materials, Figures S39–S74).

Finally, in collaboration with the US National Cancer Institute, all the newly synthesized compounds were evaluated for antitumor activity [26,27,28,29,30,31,32].

### 2.2. One-Dose Assay

Among 20 synthesized compounds, 11 inhibited the growth of cancer cells. New derivatives **2a**, **2d**, **3a**, **3b**, **5a**, **5b**, **5c**, **6a**, **6c** and **7b** were inactive whereas compound **8b** showed weak antitumor activity against one leukemia cell line. U-4CR и P-3CR products **2b**, **3c**, **4a**, **4b**, **4c** and **7c** and **3d**, **9d** and **10d** demonstrate favorable cytotoxic activity in vitro, inhibiting the growth of 4 to 39 cell lines. Based on the results of the primary NCI-60 analysis, various structural relationships of activity could be observed. In particular, in a series of diterpene P-3CR products, the part of an acid component significantly affects the cytotoxic activity. α-Acyloxycarboxamides obtained from dehydroabietic acid are found to be non-cytotoxic. Among maleopimaric acid derivatives, activity against 5 cell lines was observed only for compound **2b**, while the most active compounds were adducts of 2,3-dihydroquinopimaric acid **3c** and 1a,4a-dehydroquinopimaric acid **3d**, containing a morpholinoethyl isocyanate residue and showing activity against 10 and 26 cell lines, respectively (Table 1).

In the context of evaluating the cytotoxic activity of Ugi products **4–10**, the following structure–activity relationships were observed. In particular, the part of the amine component significantly affects the cytotoxic activity. The products obtained as a result of an interaction with glycine or *L*-methionine methyl esters do not exhibit cytotoxic activity in vitro. U-4CR products **7b** and **7c**, obtained from the reaction with *L*-histidine methyl ester, exhibit the least cytotoxic activity among this set of derivatives. The decoration of functionalizing diterpene acids with a benzylamine component had the most dramatic effect on cytotoxicity. In this set of derivatives, compounds **4a**, **4b**, and **4c** are characterized by activity against 9–11 cell lines, and leukemia cell lines turned out to be the most sensitive to them. In addition, as in the case of the P-3CR product of 1a,4a-dehydroquinopimaric acid, a noticeable increase in cytotoxicity for a set of U-4CR-1a,4a-dehydroquinopimaric acid derivatives **9d** and **10d** was observed. The details of the utilized cell lines and results are provided in Table 1 and Supplementary Materials, Figures S1–S20.

### 2.3. Five-Dose Assay

**Figure 3:**
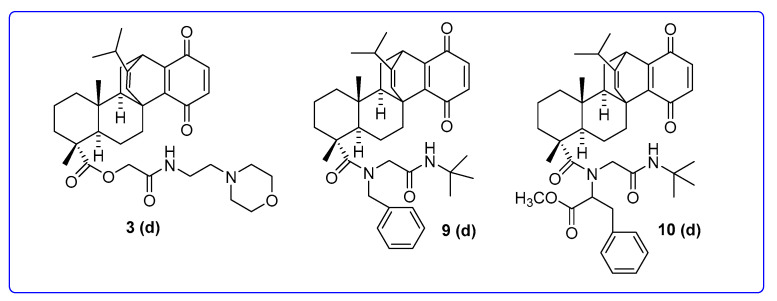
Chemical structures of NSC845565 (**3d**), NSC850906 (**9d**) and NSC850907 (**10d**).

The 1a,4a-dehydroquinopimaric acid derivatives **3d**, **9d** and **10d** with the highest potency in the one-dose assay were selected for the five-dose study (Figure 3).

Protocols for NCI60 cell five-dose screening involved seeding of about 5000~40,000 cells/well (depending on the doubling time of individual cell lines) in 96-well plates, followed by treatment with studied compounds at concentrations of 0.01, 0.1, 1.0, 10 and 100 μM and incubation at 37 °C in 5% humidified CO_2_ for 48 h. Cells were fixed with a sulforhodamine B solution followed by a series of washing and staining to determine their viability. Growth inhibition was calculated relative to cells without drug treatment and time-zero control [27,33]. The results were presented as cell growth relative to the untreated cell control and to the time-zero number of cells. Growth inhibitions were indicated by values between 0 and 100, while lethality (cytotoxic effect) was indicated by values less than 0. The outcomes were used to create log concentration vs. % growth inhibition curves, and three response parameters (GI_50_, TGI and LC_50_) were calculated for each cell line. The GI_50_ value (growth inhibitory activity) corresponds to the concentration of the compound causing a 50% decrease in net cell growth, the TGI value (cytostatic activity) is the concentration of the compound resulting in total growth inhibition and the LC_50_ value (cytotoxic activity) is the concentration of the compound causing net 50% loss of initial cells at the end of the incubation period [33]. The results of the five-dose analysis are given in Table 2. The GI_50_ values for compounds **3d**, **9d** and **10d** are shown in comparison with the standard anticancer agents doxorubicin (DRB) and 5-fluorouracil (5-FU), used by the NCI as a control [30].

**Figure 4:**
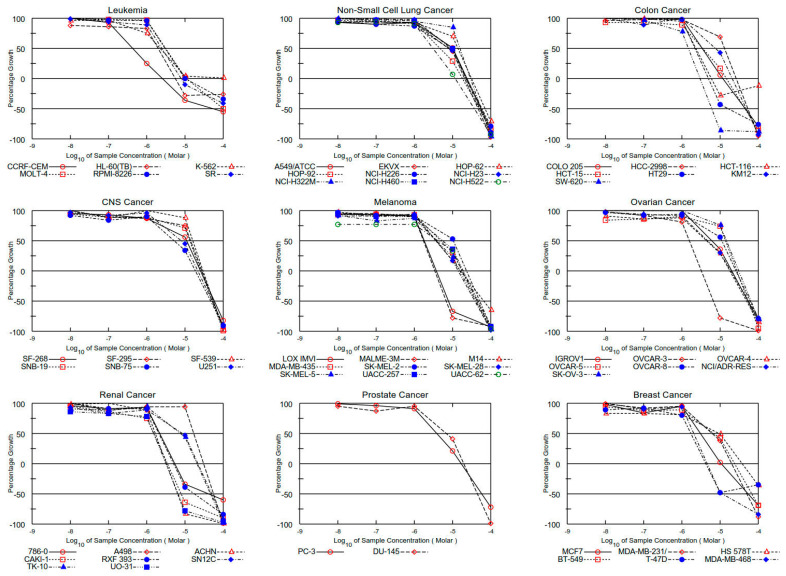
Dose–response curves for all cell lines for compound **3d**.

**Figure 5:**
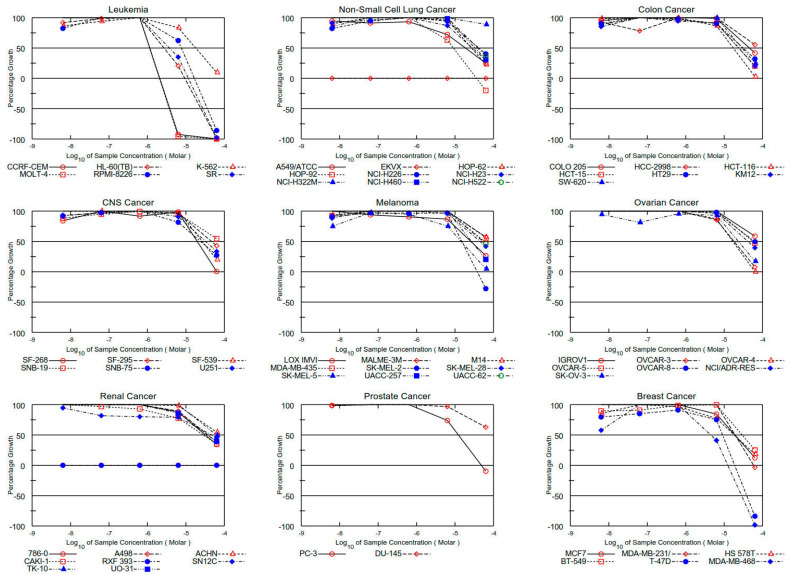
Dose–response curves for all cell lines for compound **9d**.

**Figure 6:**
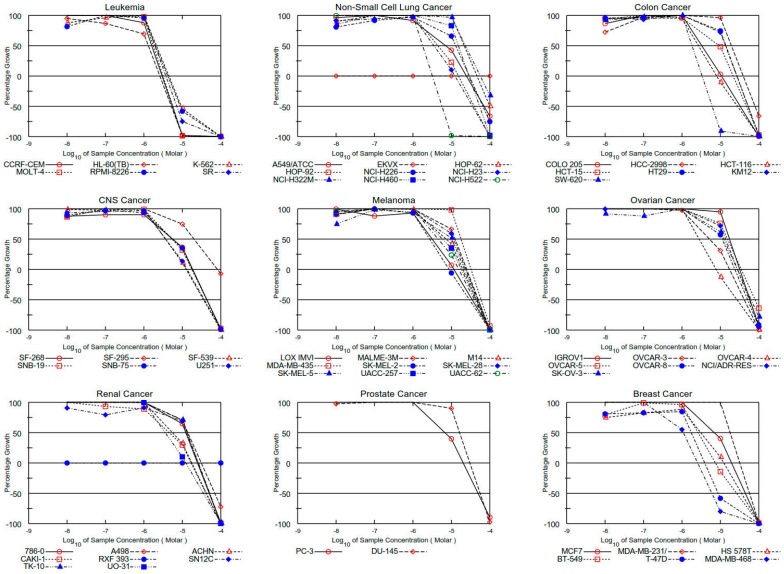
Dose–response curves for all cell lines for compound **10d**.

Thus, all compounds exhibited significant antiproliferative effects towards human cancer cell lines, and among them, the highest cytotoxic activity was observed for the P-3CR product **3d** with growth inhibitory (GI_50_) at submicromolar (0.42 μM, CCRF-CEM) and micromolar concentrations (1–3 μM) and the U-4CR product **10d** with a GI_50_ at micromolar concentrations (1–5 μM) against the most sensitive cell lines, respectively. A comparison of GI_50_ values for derivatives **3d**, **9d**, and **10d** and standard drugs [30] shows that activity of studied compounds is generally lower than the activity of DRB (with the exception of colon cancer cell lines HCT-15 and ovarian cancer NCI/ADR-RES), but they have a higher activity than 5-FU. The Ugi adduct with benzylamine substituent **9d** showed a selective activity against all leukemia cell lines with GI_50_ in the range of 1.21–17.1 μM (Table 2 and Figure 4, Figure 5 and Figure 6).

The mean values of the GI_50_ parameter for each subpanel are presented in Table 2. The data obtained showed that the panel of GI_50_ values of compounds **3d** and **10d** was significantly higher compared to that of compound **9d** (6.26 and 8.49 vs. 30.17 μM). By reducing the average antiproliferative activity against individual subpanels, compound **3d** forms the following cancer series of reduced activity: leukemia > melanoma > breast cancer > colon cancer > renal cancer > prostate cancer > ovarian cancer > NSLC > CNS cancer, whereas for compound **10d**, this sequence is as follows: leukemia > breast cancer > CNS cancer > melanoma > colon cancer > renal cancer > ovarian cancer > prostate cancer > NSLC. For compound **9d**, there is sensitivity only to the leukemia panel, while the difference in sensitivity between the other panels is not significant (Supplementary Materials, Figure S21–S38).

The selectivity index (SI) was calculated by dividing the average GI_50_ value of the full cell lines panel of compounds **3d**, **9d** and **10d** by their average GI_50_ value of the individual subpanel (Table 3). Ratios between 3 and 6 mean moderate selectivity, and ratios greater than 6 indicate high selectivity towards the corresponding cell line, while compounds not meeting either of these criteria are rated nonselective [32]. In this context, compound **3d** was found to be nonselective at all GI_50_ levels (SI 0.67–2.77), and Ugi-4CR products **9d** and **10d** were moderately selective at the GI_50_ levels towards leukemia (SI 5.49 and 4.72, respectively).

### 2.4. COMPARE Correlations

Standard COMPARE analyses allow for the comparison of the selectivity patterns of tested compounds with standard antineoplastic agents of the known mode of action and NCI active synthetic and natural compounds, which are present in publicly available databases [34] (Table 4). The application of this algorithm may provide preliminary information about the mechanism of cell growth inhibition and cell death. A quantitative evaluation of the obtained results was carried out according to the Chaddock scale using the following interpretation of pair correlation coefficients: insignificant (0.00–0.30), weak (0.30–0.50), moderate (0.50–0.70), high (0.70–0.90) and very high (0.9–1.0) [35].

As follows from Table 4, a GI_50_ COMPARE analysis of compound **3d** revealed moderate positive correlations with eprenatapopt, 3-bromopyruvic acid and ixazomib. TGI analysis also showed a moderate positive correlation with eprenetapopt, as well as with olmutinib, tepotinib and sunitinib, respectively. The cytotoxicity vector of compound **3d** had a weak correlation coefficient with tepotinib. The anticarcinogenic effect of most of the identified drugs, the average graph of which correlates with the P-3CR product **3d**, is associated with their apoptotic effect or the ability to inhibit enzymes, which increases the effectiveness of anticancer drugs and even leads to the death of cancer cells. Thus, the quinuclidinone derivative eprenetapopt exhibits antitumor activity through apoptosis of the mutant TP53 cancer cells and also exhibits synergy with other antitumor agents [36]. However, the mechanism of action of eprenetapopt as an anticancer agent still remains unresolved, although some studies suggest its ability to reduce the levels of the cellular antioxidant glutathione by increasing its turnover, causing an iron-dependent form of cell death known as ferroptosis [37]. 3-Bromopyruvic acid is an effective inhibitor of hexokinase II, causing cell death through its dissociation and thereby exerting an antitumor effect [38]. In addition, it reduces the expression of glycolytic enzymes PFKP, BPGM and GPI, depletes cells of glutathione and causes oxidative stress [39]. Ixazomib, a proteasome inhibitor, preferentially binds to and inhibits the chymotrypsin-like activity of the 20S proteasome subunit beta-5 [40], while olmutinib acts by covalently bonding to a cysteine residue near the kinase domain of the epidermal growth factor receptor (EGFR) [41], and the mechanism of action of tepotinib and sunitinib is associated with the selective inhibition of tyrosine kinase receptors involved in the processes of tumor growth, pathological angiogenesis and the formation of metastases [42,43].

According to the Chaddock scale [35], U-4CR analogs 9d and 10d had low positive correlations of the GI_50_ and TGI vectors with 6-mercaptopurine and regorafenib, as well as minor correlations with bendamustine and lapatinib at the LC_50_ level, and therefore, the resulting coefficients do not allow for high probability to explain the cytotoxicity mechanism of the tested compounds.

Taken together, the data obtained suggest that the most likely molecular targets for the derivative 3d may be proteins that carry out mitogenic signals from receptor molecules and epidermal growth factor receptors (EGFRs), and its action is associated with the ability to effectively reduce the activity of oncogenes that affect uncontrolled division tumor cells. However, the absence of high positive correlations with the above compounds requires further experimental studies to correctly interpret the results obtained. The obtained data on the antiproliferative activity of diterpene peptide-like derivatives and their molecular targets will make an important contribution to establishing the mechanisms of the putative action of abietane diterpenoids since such studies in the literature are limited to [20]. The design of such adducts is inspired by our previously described dipeptide derivatives of resin acids, which showed pronounced antiviral activity against the influenza A/Puerto Rico/8/34 (H1N1) virus and the SARS-CoV-2 pseudovirus [22]. Given that the parent 1a,4a-dehydroquinopimaric acid is not active in the NCI60 assay (Supplementary Materials, Figure S39) [44], it is also noteworthy that a rapid and cost-effective one-step protocol based on a multicomponent isocyanide method has yielded a collection of important diterpene derivatives as promising antitumor scaffolds, and the decoration of abietane acids by peptide-like substituents leads to submicromolar cytotoxicity.

### 2.5. ADMET Studies

The main ADMET properties of the tested compounds and the reference drug doxorubicin were determined and analyzed using AdmetLab 3.0 and OSIRIS DataWarrior 5.5.0 [45,46]. The calculated properties are summarized in Table 5. Compounds 3d and 9d have a high probability of bioavailability greater than 50%, while compound 10d has a probability of bioavailability greater than 50% of only 0.52. The bioavailability of doxorubicin is predicted to be less than 20%. Compounds 3d and 9d have a high drug similarity score. None of the compounds presented are predicted to be BCRP inhibitors. In contrast to doxorubicin, the investigated compounds are highly lipophilic and BBB-positive. Derivatives 3d and 9d do not affect the major cytochromes, whereas 10d is an inhibitor of CYP2C9 and CYP3A4 and may therefore increase the plasma concentration of drugs whose metabolism is mediated by these isoenzymes. Compound 10d has been shown to inhibit P-glycoprotein, while 3d and 9d have been identified as non-substrates/non-inhibitors, which may impede their transport in the body. A toxicity analysis showed that the compounds were highly unlikely to be cardiotoxic, cytotoxic or mutagenic, in contrast to the reference drug. The toxicity classes were also lower than those of doxorubicin. It is expected that 3d will be class 6 and 9d and 10d will be class 5. All compounds are expected to have respiratory toxicity and immunotoxicity, while 3d is also expected to have neuro- and nephrotoxicity. The probability of nephrotoxicity for compound 10d would be 0.54.

Thus, these results render the most active compounds as suitable drug candidates, exceeding doxorubicin in terms of toxicity, absorption and distribution. Specific untoward reactions, such as organ toxicity, are to be monitored closely in preclinical studies.

## 3. Materials and Methods

### 3.1. General

The spectra were recorded at the Center for the Collective Use “Chemistry” of the UIC UFRC RAS and RCCU “Agidel” of the UFRC RAS. ^1^H and ^13^C-NMR spectra were recorded on a “Bruker AM-500” (Bruker, Billerica, MA, USA, 500 and 125.5 MHz, respectively, δ, ppm, Hz) in CDCl_3_, internal standard tetramethylsilane. Mass spectra were obtained on a liquid chromatograph–mass spectrometer LCMS-2010 EV (Shimadzu, Kyoto, Japan). Melting points were detected on a micro table “Rapido PHMK05“ (Nagema, Dresden, Germany). Optical rotations were measured on a polarimeter “Perkin-Elmer 241 MC” (Perkin Elmer, Waltham, MA, USA) in a tube length of 1 dm. Elemental analysis was performed on a Euro EA-3000 CHNS analyzer (Eurovector, Milan, Italy); the main standard is acetanilide. Thin-layer chromatography analyses were performed on Sorbfil plates (Sorbpolimer, Krasnodar, Russian Federation), using the solvent system chloroform–ethyl acetate, 40:1. Substances were detected by 10% H_2_SO_4_ with subsequent heating to 100–120 °C for 2–3 min. All the reagents and solvents were purchased from standard commercial vendors and were used without any further purification. For the synthesis of dehydroabietic acid **1a** [47], maleopimaric acid **1b [48]**, 2,3-dihydroquinopimaric acid **1c** [49] and 1a,4a-dehydroquinopimaric acid **1d** [50], pine resin *Pinus silvestris* (containing about 25% levopimaric acid) was used. Compounds **4b** and **4c** were obtained according to the method described previously [23].

### 3.2. General Procedure for Passerini Reaction

Paraformaldehyde (1 mmol) was suspended in 10–20 mL dry methanol. The suspension was stirred for 2 h at room temperature. The diterpenic acid (1 mmol) and the corresponding isocyanide (1 mmol) (ethyl 2-isocyanoacetate for **2a**,**b**,**d** or 2-morpholinoethyl isocyanate for **3a–d**) were added, and the solution was stirred for an additional 3–4 days. The reaction mixture was poured into aqueous HCl (2M), and the precipitate formed was filtered off, washed until neutral, and dried in air. The residue was purified by column chromatography using petroleum ether/ethyl acetate as the eluent.

#### 3.2.1. 2-((2-Ethoxy-2-oxoethyl)amino)-2-oxoethyl-7-isopropyl-1,4a-dimethyl-1,2,3,4,4a,9,10,10a-octahydrophenanthrene-1-carboxylate (**2a**)

Yield 75%, mp: 62 °C, [*α*]_20_^D^ +51 (c 0.05, CHCl_3_), Rf 0.63. ^1^H NMR (δ, ppm, CDCl_3_, 500 MHz): 0.98–1.20 (4H, m, CH, CH_2_), 1.18 (3H, s, H-20), 1.20 (3H, s, H-16), 1.22 (3H, s, H-15), 1.24 (3H, s, H-19), 1.40 (3H, s, CH_3_), 1.45–2.37 (4H, m, CH, CH_2_), 2.75–3.00 (4H, m, H-6, H-7), 4.53–5.10 (6H, m, CH_2_), 5.55 (1H, br.s., NH), 6.85 (1H, s, H-17), 7.00 (1H, d, J = 7.5 Hz, H-12), 7.18 (1H, d, J = 7.5 Hz, H-11); ^13^C NMR (δ, ppm, CDCl_3_, 125.5 MHz): 179.7 (C-18), 169.6 (CO), 169.4 (CO), 146.9 (C-9), 145.6 (C-13), 136.3 (C-8), 135.1 (C-17), 124.0 (C-11), 123.7 (C-12), 61.5, 61.4, 53.2, 51.4, 47.3, 45.4, 41.2, 37.7, 36.2, 33.6, 31.9, 25.4, 24.6, 22.1, 19.5, 18.8, 14.2. Analysis calculated for C_26_H_37_NO_5_: C, 70.40; H, 8.41; N, 3.16. Found: C, 70.35; H, 8.39; N, 3.20. MS(APCI) m/z [M ]^+^ 444.3, calculated for C_26_H_38_NO_5_: 444.58.

#### 3.2.2. 2-((2-Ethoxy-2-oxoethyl)amino)-2-oxoethyl-12-isopropyl-6,9a-dimethyl-1,3-dioxo-3,3a,4,5,5a,6,7,8,9,9a,9b,10,11,11a-tetradecahydro-1*H*-3b,11-ethenophenanthro[1,2-c]furan-6-carboxylate (**2b**)

Yield 81%, mp: 55 °C, [*α*]_20_^D^ +21 (c 0.05, CHCl_3_), Rf 0.58. ^1^H NMR (δ, ppm, CDCl_3_+MeOD, 500 MHz): 0.55 (3H, s, CH_3_), 0.81–0.99 (2H, m, CH_2_), 0.98 (3H, d, J = 6.9 Hz, CH_3_), 1.00 (3H, d, J = 6.9 Hz, CH_3_), 1.10 (3H, s, CH_3_), 1.21 (3H, s, CH_3_), 1.24–1.95 (12H, m, CH, CH_2_), 2.65 (d, 1H, J = 6.8 Hz, H-1a), 2.95–3.05 (2H, m, CH_2_,), 3.15 (1H, s, H-12), 3.50–4.10 (4H, m, CH_2_), 4.50–4.65 (2H, m, CH_2_), 5.50 (1H, br. s, H-14), 7.95 (1H, br.s., NH); ^13^C NMR (δ, ppm, CDCl_3_+MeOD, 125.5 MHz): 181.9 (C-22), 178.7 (CO), 173.8 (C-24), 173.0 (C-23), 171.2 (CO), 148.0 (C-13), 125.2 (C-14), 63.5, 61.0, 53.4, 53.0, 49.7, 46.6, 45.7, 39.1, 38.0, 37.6, 35.8, 35.7, 34.9, 32.7, 29.6, 27.1, 22.0, 21.9, 20.6, 19.9, 18.6, 17.2, 15.8. Analysis calculated for C_30_H_41_NO_8_: C, 66.28; H, 7.60; N, 2.58. Found: C, 66.32; H, 7.63; N, 2.54. MS(APCI) m/z [M + H]^+^ 544.4 calculated for C_30_H_42_NO_8_: 544.66.

#### 3.2.3. 2-((2-Ethoxy-2-oxoethyl)amino)-2-oxoethyl-13-isopropyl-7,10a-dimethyl-1,4-dioxo-4,5,6,6a,7,8,9,10,10a,10b,11,12-dodecahydro-1*H*-4b,12-ethenochrysene-7-carboxylate (**2d**)

Yield 80%, mp: 75 °C, [*α*]_20_^D^ +14 (c 0.05, CHCl_3_), Rf 0.55. ^1^H NMR (δ, ppm, CDCl_3_, 500 MHz): 0.66 (3H, s, H-18), 0.68–0.73 (3H, m, CH, CH_2_), 0.90 (3H, d, J = 6.9 Hz, H-16/H-17), 0.94 (3H, d, J = 6.9 Hz, H-16/H-17), 1.15 (3H, s, H-19), 1.20 (3H, s, CH_3_), 1.18–1.32 (5H, m, CH_2_), 1.40–1.68 (6H, m, CH, CH_2_), 2.35–2.99 (2H, m, CH), 3.23 (2H, s, CH_2_), 3.80 (2H, s, CH_2_), 4.10 (2H, s, CH_2_), 5.55 (1H, br.s., NH), 5.60 (1H, s, H-14), 6.50 (2H, dd, J = 6.8 Hz, 2H, H-2, H-3); ^13^C NMR (δ, ppm, CDCl_3_, 125.5 MHz): 185.3 (C-1), 184.7 (C-4), 179.1 (C-20), 170.8 (CO), 169.9 (CO), 152.8 (C-1a), 150.7 (C-4a), 149.6 (C-13), 137.6 (C-3), 133.6 (C-2), 125.4 (C-14), 60.7, 60.6, 56.0, 54.9, 49.3, 49.1, 47.1, 39.4, 39.0, 38.5, 37.8, 36.4, 32.3, 27.0, 21.7, 20.5, 20.2, 17.1, 16.7, 16.5, 16.3. Analysis calculated for C_32_H_41_NO_7_: C, 69.67; H, 7.49; N, 2.54. Found: C, 69.62; H, 7.48; N, 2.58. MS(APCI) m/z 552.9 [M + H]^+^, calculated for C_32_H_42_NO_7_: 552.68.

#### 3.2.4. 2-((2-Morpholinoethyl)amino)-2-oxoethyl-7-isopropyl-1,4a-dimethyl-1,2,3,4,4a,9,10,10a-octahydrophenanthrene-1-carboxylate (**3a**)

Yield 77%, mp: 53 °C, [*α*]_20_^D^ +8 (c 0.05, CHCl_3_), Rf 0.45. ^1^H NMR (δ, ppm, CDCl_3_, 500 MHz): 0.98–1.20 (4H, m, CH, CH_2_), 1.18 (3H, s, H-20), 1.20 (3H, s, H-16), 1.22 (3H, s, H-15), 1.24 (3H, s, H-19), 1.45–2.07 (8H, m, CH, CH_2_), 2.35–3.00 (4H, m, H-6, H-7), 3.80–4.80 (10H, m, CH_2_), 7.15 (1H, s, H-17), 7.18 (1H, d, J = 7.5 Hz, H-12), 7.28 (1H, d, J = 7.5 Hz, H-11), 7.98 (1H, br.s., NH); ^13^C NMR (δ, ppm, CDCl_3_, 125.5 MHz): 179.7 (C-18), 169.6 (CO), 153.0 (C-9), 146.9 (C-13), 132.7 (C-8), 130.6 (C-17), 125.1 (C-11), 123.5 (C-12), 61.5, 61.4, 61.2, 61.0, 60.8, 60.2, 43.6, 37.7, 37.2, 37.1, 36.5, 33.6, 32.5, 30.2, 25.4, 24.6, 23.7, 19.5, 18.8, 16.1. Analysis calculated for C_28_H_42_N_2_O_4_: C, 71.46; H, 9.00; N, 5.95. Found: C, 71.50; H, 8.99; N, 6.00. MS(APCI) m/z 471.3 [M]^+^, calculated for C_28_H_42_N_2_O_4_: 470.65.

#### 3.2.5. 2-((2-Morpholinoethyl)amino)-2-oxoethyl-12-isopropyl-6,9a-dimethyl-1,3-dioxo-3,3a,4,5,5a,6,7,8,9,9a,9b,10,11,11a-tetradecahydro-1*H*-3b,11-ethenophenanthro[1,2-c]furan-6-carboxylate (**3b**)

Yield 82%, mp: 48 °C, [*α*]_20_^D^ +18 (c 0.05, CHCl_3_), Rf 0.49. ^1^H NMR (δ, ppm, CDCl_3_+MeOD, 500 MHz): 0.55 (3H, s, CH_3_), 0.81–0.99 (2H, m, CH_2_), 0.98 (3H, d, J = 6.9 Hz, CH_3_), 1.00 (3H, d, J = 6.9 Hz, CH_3_), 1.10 (3H, s, CH_3_), 1.24–1.95 (16H, m, CH, CH_2_), 2.65 (d, 1H, J = 6.8 Hz, H-1a), 2.95–3.05 (2H, m, CH_2_,), 3.15 (1H, s, H-12), 3.50–4.10 (8H, m, CH_2_), 5.49 (2H, br.s., CH_2_), 5.65 (1H, br. s, H-14), 7.95 (1H, br.s., NH); ^13^C NMR (δ, ppm, CDCl_3_+MeOD, 125.5 MHz): 178.7 (C-22), 173.7 (C-24), 173.0 (C-23), 171.2 (CO), 148.0 (C-13), 125.2 (C-14), 66.7, 66.7, 63.2, 53.4, 53.2, 53.0, 52.7, 49.7, 46.6, 45.7, 39.1, 38.0, 37.6, 35.8, 35.7, 34.9, 32.7, 29.6, 27.1, 22.0, 21.9, 20.6, 19.9, 18.6, 17.2, 15.8. Analysis calculated for C_32_H_46_N_2_O_7_: C, 67.34; H, 8.12; N, 4.91. Found: C, 67.32; H, 8.15; N, 4.94. MS(APCI) m/z 571.4 [M + H]^+^, calculated for C_32_H_47_N_2_O_7_: 571.73.

#### 3.2.6. 2-((2-Morpholinoethyl)amino)-2-oxoethyl-13-isopropyl-7,10a-dimethyl-1,4-dioxo-2,3,4,4a,5,6,6a,7,8,9,10,10a,10b,11,12,12a-hexadecahydro-1*H*-4b,12-ethenochrysene-7-carboxylate (**3c**)

Yield 79%, mp: 92 °C, [*α*]_20_^D^ +11 (c 0.05, CHCl_3_), Rf 0.40.^1^H NMR (δ, ppm, CDCl_3_, 500 MHz): 0.59 (3H, s, CH_3_), 0.61–0.90 (2H, m, CH_2_), 0.98 (3H, d, J = 6.9 Hz, CH_3_), 1.00 (3H, d, J = 6.9 Hz, CH_3_), 1.18 (3H, s, CH_3_), 1.28–1.92 (6H, m, CH, CH_2_), 2.14–2.25 (11H, m, CH, CH_2_), 2.62–2.88 (9H, m, CH, CH_2_), 3.25 (2H, s, CH_2_), 3.60–3.80 (4H, m, CH_2_, CH_3_), 5.40 (1H, br. s, H-14), 5.60–5.75 (2H, m, CH_2_), 6.25 (1H, br.s, NH); ^13^C NMR (δ, ppm, CDCl_3_, 125.5 MHz): 210.1 (C-4), 208.9 (C-1), 184.8 (C-20), 179.1 (CO), 147.4 (C-13), 125.4 (C-14), 60.6, 60.5, 58.7, 56.0, 55.9, 54.9, 52.0, 50.6, 49.3, 49.0, 41.5, 39.2, 38.9, 37.7, 37.0, 36.7, 35.1, 34.7, 32.9, 27.7, 25.4, 21.8, 20.8, 19.9, 16.9, 16.7, 16.4, 15.9. Analysis calculated for C_34_H_50_N_2_O_6_: C, 70.07; H, 8.65; N, 4.81. Found: C, 70.00; H, 8.60; N, 4.89. MS(APCI) m/z 583.9 [M + H]^+^, calculated for C_34_H_51_N_2_O_6_: 583.78.

#### 3.2.7. 2-((2-Morpholinoethyl)amino)-2-oxoethyl-13-isopropyl-7,10a-dimethyl-1,4-dioxo-4,5,6,6a,7,8,9,10,10a,10b,11,12-dodecahydro-1*H*-4b,12-ethenochrysene-7-carboxylate (**3d**)

Yield 75%, mp: 110 °C, [*α*]_20_^D^ +20 (c 0.05, CHCl_3_), Rf 0.39. ^1^H NMR (δ, ppm, CDCl_3_, 500 MHz): 0.64 (3H, s, H-18), 0.68–0.73 (3H, m, CH, CH_2_), 0.90 (3H, d, J = 6.9 Hz, H-16/H-17), 0.94 (3H, d, J = 6.9 Hz, H-16/H-17), 1.15 (3H, s, H-19), 1.20 (3H, s, CH_3_), 1.18–1.33 (5H, m, CH_2_), 1.40–1.68 (9H, m, CH, CH_2_), 2.35–2.99 (2H, m, CH), 2.90–2.93 (2H, м, CH_2_), 4.05–4.10 (2H, м, CH_2_), 4.20–4.30 (4H, м, CH_2_), 5.55 (1H, s, H-14), 5.62 (1H, br.s., NH), 6.50 (2H, dd, J = 6.8 Hz, 2H, H-2, H-3); ^13^C NMR (δ, ppm, CDCl_3_, 125.5 MHz): 200.4 (C-1), 195.9 (C-4), 184.1 (C-20), 167.8 (CO), 151.2 (C-1a), 144.7 (C-13), 142.8 (C-4a), 137.6 (C-3), 135.6 (C-2), 124.9 (C-14), 68.2, 67.6, 59.2, 59.0, 54.9, 49.3, 49.1, 47.1, 39.4, 39.0, 38.2, 38.5, 37.8, 36.4, 32.3, 27.0, 25.3, 22.0, 21.7, 20.5, 20.2, 17.1, 16.8, 16.6. Analysis calculated for C_34_H_46_N_2_O_6_: C, 70.56; H, 8.01; N, 4.84. Found: C, 70.60; H, 8.08; N, 4.90. MS(APCI) m/z 579.9 [M + H]^+^, calculated for C_34_H_47_N_2_O_6_: 579.75.

### 3.3. General Procedure for Ugi Reaction

Paraformaldehyde (1 mmol) was suspended in 10–20 mL dry methanol, followed by the addition of an amine or methyl ester of the corresponding amino acid (1.2 mmol) (benzylamine for **4a–c**, **9d**, methyl esters of glycine for **5a–c**, methyl esters of *L*-methionine for **6a**,**c**, methyl esters of *L*-histidine for **7b**,**c** and methyl esters of *L*-phenylalanine for **8b**, **10d**). The suspension was stirred for 2 h at room temperature. The diterpenic acid (1 mmol) and the corresponding isocyanide (1 mmol) (ethyl 2-isocyanoacetate for **4a–c**, **5a–c**, **6a**,**c**, 2-morpholinoethyl isocyanate for **7b**,**c** or *tert*-butyl isocyanide for **9d**, **10d**) were added, and the solution was stirred for an additional 5–7 days. The reaction mixture was poured into aqueous HCl (2M), and the precipitate formed was filtered off, washed until neutral, and dried in air. The residue was purified by column chromatography using petroleum ether/ethyl acetate as the eluent.

#### 3.3.1. Ethyl *N*-benzyl-*N*-(7-isopropyl-1,4a-dimethyl-1,2,3,4,4a,9,10,10a-octahydrophenanthrene-1-carbonyl)glycylglycinate (**4a**)

Yield 85%, mp: 61 °C, [*α*]_20_^D^ -16 (c 0.05, CHCl_3_), Rf 0.50. ^1^H NMR (δ, ppm, CDCl_3_, 500 MHz): 0.98–1.20 (4H, m, CH, CH_2_), 1.18 (3H, s, H-20), 1.20 (3H, s, H-16), 1.22 (3H, s, H-15), 1.24 (3H, s, H-19), 1.42 (3H, s, CH_3_), 1.45–2.37 (4H, m, CH, CH_2_), 2.75–3.10 (4H, m, H-6, H-7), 3.73 (2H, d, J = 16.4 Hz, CH_2_), 4.00 (2H, d, J = 16.4 Hz, CH_2_), 4.15–4.20 (2H, m, CH_2_), 4.65 (1H, d, J = 16.4 Hz, CH_2_), 5.10 (1H, d, J = 16.4 Hz, CH_2_), 6.65 (1H, br.s., NH), 6.85 (1H, s, H-17), 7.00 (1H, d, J = 7.5 Hz, H-12), 7.18 (1H, d, J = 7.5 Hz, H-11), 7.20–7.45 (5H, m, H-Ar); ^13^C NMR (δ, ppm, CDCl_3_, 125.5 MHz): 179.7 (C-18), 169.6 (CO), 169.4 (CO), 146.9 (C-9), 145.6 (C-13), 136.3 (C-8), 135.1, 128.9, 128.8, 127.7, 127.6, 127.0, 126.9 (C-17), 124.0 (C-11), 123.7 (C-12), 61.5, 53.2, 51.4, 47.3, 45.4, 41.2, 37.7, 36.2, 33.6, 32.5, 31.9, 30.2, 25.4, 24.6, 22.1, 19.5, 18.8, 14.2. Analysis calculated for C_33_H_44_N_2_O_4_: C, 74.40; H, 8.33; N, 5.26. Found: C, 74.50; H, 8.29; N, 5.30. MS(APCI) m/z 532.3 [M]^+^, calculated for C_33_H_45_N_2_O_4_: 532.73.

#### 3.3.2. Ethyl *N*-(7-isopropyl-1,4a-dimethyl-1,2,3,4,4a,9,10,10a-octahydrophenanthrene-1-carbonyl)-*N*-(2-methoxy-2-oxoethyl)glycylglycinate (**5a**)

Yield 72%, mp: 51 °C, [*α*]_20_^D^ -17 (c 0.05, CHCl_3_), Rf 0.42. ^1^H NMR (δ, ppm, CDCl_3_, 500 MHz): 0.78–1.10 (4H, m, CH, CH_2_), 1.18 (3H, s, H-20), 1.20 (3H, s, H-16), 1.22 (3H, s, H-15), 1.24 (3H, s, H-19), 1.42 (3H, s, CH_3_), 1.45–2.37 (4H, m, CH, CH_2_), 2.75–3.00 (4H, m, H-6, H-7), 3.65–3.73 (5H, m, CH_2_, CH_3_), 3.85–4.40 (6H, m, CH_2_), 6.85 (1H, s, H-17), 7.00 (1H, d, J = 7.5 Hz, H-12), 7.18 (1H, d, J = 7.5 Hz, H-11), 7.95 (1H, br.s., NH); ^13^C NMR (δ, ppm, CDCl_3_, 125.5 MHz): 179.6 (C-18), 171.2 (CO), 169.4 (CO), 169.3 (CO), 146.8 (C-9), 145.6 (C-13), 134.9 (C-8), 126.9 (C-17), 124.1 (C-11), 123.8 (C-12), 61.3, 54.4, 52.6, 52.4, 45.3, 42.6, 41.2, 37.6, 35.8, 33.4, 32.5, 30.1, 27.1, 26.6, 25.2, 23.9, 18.6, 17.9, 14.1. Analysis calculated for C_29_H_42_N_2_O_6_: C, 67.68; H, 8.23; N, 5.44. Found: C, 67.71; H, 8.20; N, 5.40. MS(APCI) m/z 514.3 [M]^+^, calculated for C_29_H_42_N_2_O_6_: 514.66.

#### 3.3.3. Ethyl *N*-(12-isopropyl-6,9a-dimethyl-1,3-dioxo-3,3a,4,5,5a,6,7,8,9,9a,9b,10,11,11a-tetradecahydro-1H-3b,11-ethenophenanthro[1,2-c]furan-6-carbonyl)-*N*-(2-methoxy-2-oxoethyl)glycylglycinate (**5b**)

Yield 67%, mp: 60 °C, [*α*]_20_^D^ -15 (c 0.05, CHCl_3_), Rf 0.40. ^1^H NMR (δ, ppm, CDCl_3_, 500 MHz): 0.55 (3H, s, CH_3_), 0.81–0.99 (2H, m, CH_2_), 0.98 (3H, d, J = 6.9 Hz, CH_3_), 1.00 (3H, d, J = 6.9 Hz, CH_3_), 1.10 (3H, s, CH_3_), 1.24–1.35 (4H, m, CH, CH_2_), 1.42 (3H, s, CH_3_), 1.44–1.95 (3H, m, CH, CH_2_), 2.25–2.45 (5H, m, CH, CH_2_), 2.55 (1H, d, J = 6.8 Hz, H-1a), 2.95–3.05 (2H, m, CH_2_), 3.10 (1H, s, H-12), 3.45–3.79 (5H, m, CH_2_, CH_3_), 3.95–4.50 (6H, m, CH_2_), 5.50 (1H, br. s, H-14), 7.95 (1H, br.s., NH); ^13^C NMR (δ, ppm, CDCl_3_, 125.5 MHz): 179.4 (C-22), 172.9 (C-24), 171.4 (C-23), 169.8 (CO), 169.4 (CO), 169.3 (CO), 148.0 (C-13), 125.2 (C-14), 61.3, 54.4, 53.6, 53.0, 49.7, 46.6, 45.7, 39.1, 38.0, 37.6, 35.8, 35.7, 35.6, 34.9, 32.7, 29.6, 27.1, 22.0, 21.9, 20.6, 19.9, 18.6, 17.2, 15.8, 14.1. Analysis calculated for C_33_H_46_N_2_O_9_: C, 64.48; H, 7.54; N, 4.56. Found: C, 64.46; H, 7.53; N, 4.54. MS(APCI) m/z 615.4 [M + H]^+^, calculated for C_33_H_47_N_2_O_9_: 615.74.

#### 3.3.4. Ethyl *N*-(13-isopropyl-7,10a-dimethyl-1,4-dioxo-2,3,4,4a,5,6,6a,7,8,9,10,10a,10b,11,12,12a-hexadecahydro-1*H*-4b,12-ethenochrysene-7-carbonyl)-*N*-(2-methoxy-2-oxoethyl)glycylglycinate (**5c**)

Yield 70%, mp: 67 °C, [*α*]_20_^D^ +26 (c 0.05, CHCl_3_), Rf 0.41. ^1^H NMR (δ, ppm, CDCl_3_, 500 MHz): 0.60 (3H, s, CH_3_), 0.61–0.90 (2H, m, CH_2_), 0.98 (3H, d, J = 6.9 Hz, CH_3_), 1.00 (3H, d, J = 6.9 Hz, CH_3_), 1.18 (3H, s, CH_3_), 1.22 (3H, s, CH_3_), 1.28–1.92 (6H, m, CH, CH_2_), 2.14–2.25 (9H, m, CH, CH_2_), 2.62–2.88 (4H, m, CH, CH_2_), 3.15 (1H, s, H-12), 3.65–3.73 (5H, m, CH_2_, CH_3_), 3.85–4.40 (6H, m, CH_2_), 5.49 (1H, br. s, H-14), 6.75 (1H, br.s, NH), ^13^C NMR (δ, ppm, CDCl_3_, 125.5 MHz): 209.6 (C-4), 208.7 (C-1), 179.1 (C-20), 170.6 (CO), 169.8 (CO), 167.4 (CO), 149.4 (C-13), 125.5 (C-14), 62.7, 60.5, 56.2, 55.2, 54.8, 52.5, 49.1, 48.9, 48.7, 41.3, 39.2, 38.6, 37.9, 37.0, 35.7, 34.9, 32.9, 32.6, 27.5, 25.4, 24.7, 22.3, 20.8, 19.9, 16.8, 16.0, 15.8. Analysis calculated for C_35_H_50_N_2_O_8_: C, 67.07; H, 8.04; N, 4.47. Found: C, 67.03; H, 8.01; N, 4.44. MS(APCI) m/z 627.4 [M + H]^+^, calculated for C_35_H_51_N_2_O_8_: 627.79.

#### 3.3.5. Methyl *N*-(2-((2-ethoxy-2-oxoethyl)amino)-2-oxoethyl)-*N*-(7-isopropyl-1,4a-dimethyl-1,2,3,4,4a,9,10,10a-octahydrophenanthrene-1-carbonyl)methioninate (**6a**)

Yield 69%, mp: 50 °C, [*α*]_20_^D^ -57 (c 0.05, CHCl_3_), Rf 0.35. ^1^H NMR (δ, ppm, CDCl_3_, 500 MHz): 0.78–1.10 (4H, m, CH, CH_2_), 1.18 (3H, s, H-20), 1.20 (3H, s, H-16), 1.22 (3H, s, H-15), 1.24 (3H, s, H-19), 1.38 (3H, s, CH_3_), 1.40–2.00 (4H, m, CH, CH_2_), 2.01 (3H, s, CH_3_), 2.20–3.00 (4H, m, H-6, H-7), 3.65–3.73 (6H, m, CH, CH_2_, CH_3_), 3.90–4.20 (6H, m, CH_2_), 4.50–4.70 (2H, m, CH_2_), 6.85 (1H, s, H-17), 7.00 (1H, d, J = 7.5 Hz, H-12), 7.18 (1H, d, J = 7.5 Hz, H-11), 8.20 (1H, br.s., NH); ^13^C NMR (δ, ppm, CDCl_3_, 125.5 MHz): 178.1 (C-18), 171.9 (CO), 169.8 (CO), 167.4 (CO), 146.9 (C-9), 145.6 (C-13), 134.9 (C-8), 126.9 (C-17), 124.1 (C-11), 123.8 (C-12), 62.8, 61.3, 52.8, 52.1, 47.8, 46.9, 45.2, 41.2, 37.9, 36.8, 35.8, 34.4, 33.4, 32.5, 30.1, 27.1, 25.2, 23.9, 18.6, 17.9, 15.4, 14.1. Analysis calculated for C_32_H_48_N_2_O_6_S: C, 65.28; H, 8.22; N, 4.76; S, 5.44. Found: C, 65.30; H, 8.20; N, 5.00; S, 5.40. MS(APCI) m/z 588.3 [M]^+^, calculated for C_32_H_48_N_2_O_6_S: 588.80.

#### 3.3.6. Methyl *N*-(2-((2-ethoxy-2-oxoethyl)amino)-2-oxoethyl)-*N*-(13-isopropyl-7,10a-dimethyl-1,4-dioxo-2,3,4,4a,5,6,6a,7,8,9,10,10a,10b,11,12,12a-hexadecahydro-1*H*-4b,12-ethenochrysene-7-carbonyl)methioninate (**6c**)

Yield 74%, mp: 74 °C, [*α*]_20_^D^ -24 (c 0.05, CHCl_3_), Rf 0.30. ^1^H NMR (δ, ppm, CDCl_3_, 500 MHz): 0.50 (3H, s, CH_3_), 0.61–0.90 (2H, m, CH_2_), 0.98 (3H, d, J = 6.9 Hz, CH_3_), 1.00 (3H, d, J = 6.9 Hz, CH_3_), 1.18 (3H, s, CH_3_), 1.28–1.92 (9H, m, CH, CH_2_), 2.00 (3H, s, CH_3_), 2.14–2.25 (8H, m, CH, CH_2_), 2.62–2.88 (4H, m, CH, CH_2_), 3.15 (1H, s, H-12), 3.65–3.73 (6H, m, CH, CH_2_, CH_3_), 3.85–4.40 (6H, m, CH_2_), 4.50–4.70 (2H, m, CH_2_), 5.50 (1H, br. s, H-14), 8.75 (1H, br.s, NH), ^13^C NMR (δ, ppm, CDCl_3_, 125.5 MHz): 209.8 (C-4), 208.7 (C-1), 178.3 (C-20), 172.5 (CO), 169.7 (CO), 169.5 (CO), 149.4 (C-13), 125.7 (C-14), 62.9, 60.5, 56.3, 55.2, 54.9, 52.9, 52.2, 49.4, 47.2, 46.6, 41.2, 41.1, 40.9, 39.0, 38.4, 37.1, 37.0, 35.8, 34.7, 32.9, 31.1, 27.6, 21.9, 20.8, 19.9, 17.3, 17.0, 16.4, 16.0, 15.4. Analysis calculated for C_38_H_56_N_2_O_8_S: C, 65.12; H, 8.05; N, 4.00; S, 4.57. Found: C, 65.20; H, 8.00; N, 4.10; S, 4.60. MS(APCI) m/z 701.4 [M + H]^+^, calculated for C_38_H_57_N_2_O_8_S: 701.93.

#### 3.3.7. Methyl *N*-(12-isopropyl-6,9a-dimethyl-1,3-dioxo-3,3a,4,5,5a,6,7,8,9,9a,9b,10,11,11a-tetradecahydro-1*H*-3b,11-ethenophenanthro[1,2-c]furan-6-carbonyl)-*N*-(2-((2-morpholinoethyl)amino)-2-oxoethyl)histidinate (**7b**)

Yield 66%, mp: 55 °C, [*α*]_20_^D^ +15 (c 0.05, CHCl_3_), Rf 0.30. ^1^H NMR (δ, ppm, CDCl_3_, 500 MHz): 0.55 (3H, s, CH_3_), 0.81–0.99 (2H, m, CH_2_), 0.98 (3H, d, J = 6.9 Hz, CH_3_), 1.00 (3H, d, J = 6.9 Hz, CH_3_), 1.10 (3H, s, CH_3_), 1.24–1.95 (12H, m, CH, CH_2_), 2.25–2.45 (5H, m, CH, CH_2_), 2.95–3.05 (8H, m, CH_2_,), 3.15 (1H, s, H-12), 3.57–3.75 (7H, m, CH_2_, CH_3_), 4.05–4.10 (2H, br.s., CH_2_), 5.42 (1H, br. s, H-14), 5.60–5.72 (1H, m, CH), 6.25 (1H, s, CH), 6.50 (1H, s, CH), 7.90 (1H, br.s, NH), 9.11 (1H, br.s, NH); ^13^C NMR (δ, ppm, CDCl_3_, 125.5 MHz): 178.4 (C-20), 172.9 (CO), 172.0 (C-24), 171.9 (C-23), 171.1 (CO), 151.1, 148.2 (C-13), 141.1, 125.5 (C-14), 125.4, 60.6, 60.5, 60.3, 58.6, 58.7, 56.0, 55.9, 54.9, 53.6, 53.0, 49.7, 46.6, 45.7, 39.1, 38.0, 37.6, 35.8, 35.7, 34.9, 32.7, 29.6, 27.1, 22.0, 21.9, 20.6, 19.9, 18.6, 17.2, 15.8. 52.0, 50.6, 49.3, 49.0, 41.5, 39.2, 38.9, 38.5, 37.7, 37.0, 36.7, 35.1, 34.7, 32.9, 27.7, 25.4, 21.8, 20.8, 19.9, 16.9, 16.7, 16.4, 15.9. Analysis calculated for C_39_H_55_N_5_O_8_: C, 64.89; H, 7.68; N, 9.70. Found: C, 64.90; H, 7.77; N, 9.74. MS(APCI) m/z 722.4 [M + H]^+^, calculated for C_39_H_56_N_5_O_8_: 722.90.

#### 3.3.8. Methyl *N*-(13-isopropyl-7,10a-dimethyl-1,4-dioxo-2,3,4,4a,5,6,6a,7,8,9,10,10a,10b,11,12,12a-hexadecahydro-1*H*-4b,12-ethenochrysene-7-carbonyl)-*N*-(2-((2-morpholinoethyl)amino)-2-oxoethyl)histidinate (**7c**)

Yield 69%. Here, mp: 75 °C, [*α*]_20_^D^ +14 (c 0.05, CHCl_3_), Rf 0.33. ^1^H NMR (δ, ppm, CDCl_3_, 500 MHz): 0.60 (3H, s, CH_3_), 0.61–0.90 (2H, m, CH_2_), 0.98 (3H, d, J = 6.9 Hz, CH_3_), 1.00 (3H, d, J = 6.9 Hz, CH_3_), 1.18 (3H, s, CH_3_), 1.28–1.92 (6H, m, CH, CH_2_), 2.14–2.25 (15H, m, CH, CH_2_), 2.62–2.88 (8H, m, CH, CH_2_), 3.15 (1H, s, H-12), 3.60–3.80 (7H, m, CH_2_, CH_3_), 4.08–4.11 (2H, br.s., CH_2_), 5.40 (1H, br. s, H-14), 5.60–5.75 (1H, m, CH), 6.29 (1H, s, CH), 6.45 (1H, s, CH), 7.95 (1H, br.s, NH), 9.15 (1H, br.s, NH),^13^C NMR (δ, ppm, CDCl_3_, 125.5 MHz): 210.1 (C-4), 208.9 (C-1), 179.1 (C-20), 172.9 (CO), 170.2 (CO), 151.2, 149.6 (C-13), 141.1, 125.5 (C-14), 125.4, 60.6, 60.5, 60.3, 58.6, 58.7, 56.0, 55.9, 54.9, 52.0, 50.6, 49.3, 49.0, 41.5, 39.2, 38.9, 38.5, 37.7, 37.0, 36.7, 35.1, 34.7, 32.9, 27.7, 25.4, 21.8, 20.8, 19.9, 16.9, 16.7, 16.4, 15.9. Analysis calculated for C_41_H_59_N_5_O_7_: C, 67.10; H, 8.10; N, 9.54. Found: C, 67.20; H, 8.10; N, 9.60. MS(APCI) m/z 734.4 [M + H]^+^, calculated for C_41_H_60_N_5_O_7_: 734.95.

#### 3.3.9. Methyl *N*-(12-isopropyl-6,9a-dimethyl-1,3-dioxo-3,3a,4,5,5a,6,7,8,9,9a,9b,10,11,11a-tetradecahydro-1*H*-3b,11-ethenophenanthro[1,2-c]furan-6-carbonyl)-*N*-(2-((2-morpholinoethyl)amino)-2-oxoethyl)phenylalaninate (**8b**)

Yield 82%, mp: 63 °C, [*α*]_20_^D^ -42 (c 0.05, CHCl_3_), Rf 0.45. ^1^H NMR (δ, ppm, CDCl_3_, 500 MHz): 0.55 (3H, s, CH_3_), 0.81–0.99 (2H, m, CH_2_), 0.98 (3H, d, J = 6.9 Hz, CH_3_), 1.00 (3H, d, J = 6.9 Hz, CH_3_), 1.10 (3H, s, CH_3_), 1.24–1.95 (15H, m, CH, CH_2_), 2.25–2.95 (8H, m, CH, CH_2_), 3.15–3.20 (4H, m, H-12, CH_3_), 3.25–4.08 (8H, m, CH_2_), 4.50–4.70 (1H, m, CH), 5.50 (1H, br. s, H-14), 7.10–7.22 (5H, m, H-Ar), 8.50 (1H, br.s., NH); ^13^C NMR (δ, ppm, CDCl_3_, 125.5 MHz): 178.4 (C-24), 177.2 (C-23), 172.8 (CO), 170.9 (CO), 167.7 (CO), 148.1 (C-13), 137.2, 129.5, 129.4, 129.1, 128.9, 127.1, 125.2 (C-14), 66.7, 66.5, 56.9, 56.1, 53.7, 53.2, 53.1, 49.6, 49.2, 45.7, 39.1, 38.0, 37.4, 36.8, 36.2, 35.7, 34.8, 34.7, 32.7, 29.6, 27.2, 21.9, 20.6, 19.9, 18.6, 17.1, 16.6, 16.0, 15.5. Analysis calculated for C_42_H_57_N_3_O_8_: C, 68.92; H, 7.85; N, 5.74. Found: C, 69.00; H, 7.90; N, 5.80. MS(APCI) m/z 732.4 [M + H]^+^, calculated for C_42_H_58_N_3_O_8_: 732.93.

#### 3.3.10. *N*-Benzyl-*N*-(2-(tert-butylamino)-2-oxoethyl)-13-isopropyl-7,10a-dimethyl-1,4-dioxo-4,5,6,6a,7,8,9,10,10a,10b,11,12-dodecahydro-1*H*-4b,12-ethenochrysene-7-carboxamide (**9d**)

Yield 83%, mp: 51 °C, [*α*]_20_^D^ -14 (c 0.05, CHCl_3_), Rf 0.41. ^1^H NMR (δ, ppm, CDCl_3_, 500 MHz): 0.70 (3H, s, H-18), 0.71–0.75 (3H, m, CH, CH_2_), 0.99 (3H, d, J = 6.9 Hz, H-16/H-17), 1.02 (3H, d, J = 6.9 Hz, H-16/H-17), 1.22 (3H, s, H-19), 1.25 (9H, s, 3CH_3_), 1.28–1.35 (5H, m, CH_2_), 1.40–1.68 (6H, m, CH, CH_2_), 2.35–2.99 (2H, m, CH), 3.70 (2H, s, CH_2_), 4.95 (2H, dd, J = 6.8 Hz, H-2, H-3); 5.60 (2H, s, CH_2_), 5.70 (1H, s, H-14), 6.10 (1H, br.s., NH), 7.10–7.22 (5H, m, H-Ar); ^13^C NMR (δ, ppm, CDCl_3_, 125.5 MHz): 183.9 (C-20), 179.9 (C-1), 178.9 (C-4), 168.4 (CO), 158.9 (C-1a), 153.8 (C-4a), 151.1 (C-2), 148.9 (C-13), 136.5 (C-3), 128.9, 128.8, 127.8, 127.6, 127.5, 127.4, 126.9 (C-14), 56.4, 55.4, 53.2, 53.1, 51.2, 50.2, 49.2, 47.2, 39.0, 36.6, 36.2, 32.0, 31.9, 28.8 (3CH_3_), 24.5, 22.3, 20.7, 20.4, 18.8, 17.3, 16.7. Analysis calculated for C_39_H_50_N_2_O_4_: C, 76.69; H, 8.25; N, 4.59. Found: C, 76.71; H, 8.28; N, 4.60. MS(APCI) m/z 611.4 [M + H]^+^, calculated for C_39_H_51_N_2_O_4_: 611.84.

#### 3.3.11. Methyl *N*-(2-(tert-butylamino)-2-oxoethyl)-*N*-(13-isopropyl-7,10a-dimethyl-1,4-dioxo-4,5,6,6a,7,8,9,10,10a,10b,11,12-dodecahydro-1*H*-4b,12-ethenochrysene-7-carbonyl)phenylalaninate (**10d**)

Yield 80%, mp: 59 °C, [*α*]_20_^D^ -93 (c 0.05, CHCl_3_), Rf 0.40. ^1^H NMR (δ, ppm, CDCl_3_, 500 MHz): 0.70 (3H, s, H-18), 0.71–0.75 (3H, m, CH, CH_2_), 0.99 (3H, d, J = 6.9 Hz, H-16/H-17), 1.02 (3H, d, J = 6.9 Hz, H-16/H-17), 1.22 (3H, s, H-19), 1.25 (9H, s, 3CH_3_), 1.28–1.35 (5H, m, CH_2_), 1.40–1.68 (6H, m, CH, CH_2_), 2.35–2.99 (2H, m, CH), 3.23–3.40 (2H, m, CH_2_), 3.75 (3H, s, CH_3_), 3.95–4.0 (2H, m, CH_2_), 5.10 (1H, s, CH), 5.60 (1H, s, H-14), 6.49 (2H, dd, J = 6.8 Hz, 2H, H-2, H-3); 7.10–7.22 (5H, m, H-Ar), 7.95 (1H, br.s., NH); ^13^C NMR (δ, ppm, CDCl_3_, 125.5 MHz): 185.2 (C-1), 184.0 (C-4), 178.3 (C-20), 171.6 (CO), 168.1 (CO), 152.8 (C-1a), 150.9 (C-4a), 150.7 (C-13), 137.5 (C-2), 137.4 (C-3), 133.6, 129.4, 128.9, 127.7, 127.4, 127.3, 127.2, 127.1 (C-14), 67.3, 55.4, 55.3, 54.9, 52.8, 51.6, 49.5, 49.3, 49.2, 46.8, 46.5, 39.4, 39.0, 36.6, 36.2, 32.0, 31.9, 29.8, 28.8 (3CH_3_), 27.2, 24.5, 22.3, 20.7, 20.4, 18.8, 17.3, 16.7. Analysis calculated for C_42_H_54_N_2_O_6_: C, 73.87; H, 7.97; N, 4.10. Found: C, 73.90; H, 7.95; N, 4.10. MS(APCI) m/z 682.4 [M]^+^, calculated for C_42_H_54_N_2_O_6_: 682.90.

### 3.4. Biological Assay

The in vitro anticancer screening of the tested compounds is given in Supplementary Materials.

## 4. Conclusions

Thus, the potential of natural and semisynthetic diterpene acids as carboxyl moieties for modification under Ugi and Passerini MCR conditions was demonstrated using dehydroabietic acid and levopimaric acid diene adducts. The first set of P-3CR products was obtained by varying isocyanides, while the second set of U-4CR products used various isocyanides and amines, including amino acid methyl esters. The novel series of diterpene α-acyloxycarboxamide и α-acylamidocarboxamides have been synthesized in good yields and displayed different antiproliferative effects on an NCI-60 panel of tumor cell lines. Among them, eleven compounds inhibited the growth of cancer cells in 4 to 39 cell lines in a one-dose assay. The greatest activity in the five-dose assay was shown by adducts **3d**, **9d** and **10d**, which not only stopped the growth of cancer cells but also caused their death. Compound **3d**, in contrast to **9d** and **10d**, showed moderate positive correlations with standard anticancer drugs that can influence the function of kinase receptors and EGFRs, and thereby effectively reduce the activity of oncogenes that affect the uncontrolled division of tumor cells. The ADMET analysis acknowledges the favorable prognosis using compounds as potential anticancer agents, exceeding doxorubicin in terms of toxicity, absorption and distribution. Based on the obtained data, it can be concluded that among the synthesized diterpene α-acyloxycarboxamide and α-acylamidocarboxamides, the derivatives of 1a,4a-dehydroquinopimaric acid are the most promising compounds for further study of their antitumor activity, and, in general, the decoration of abietane acids with peptide-like substituents leads to submicromolar cytotoxicity.

## Figures and Tables

**Table 1:** Cytotoxicity of compounds **2a**, **2b**, **2d**, **3a**, **3b**, **3c**, **3d**, **4a**, **4b**, **4c**, **5a**, **5b**, **5c**, **6a**, **6c**, **7b**, **7c**, **8b**, **9d** and **10d** at a concentration of 10^−5^ M on NCI-60 cancer cell lines.

Comp.	60 Cell Lines Assay in 1 Dose 10 µM Conc.		The Most Sensitive Cell Lines (Growth, %),
	Mean Growth, %	Range of Growth, %	
**2a**	100.70	79.35–120.08	Not active
**2b**	62.71	−6.49–122.05	Leukemia: CCRF-CEM (26.08), HL-60(TB) (−6.49), K-562 (20.12), MOLT-4 (7.97), Breast cancer: MDA-MB-468 (−4.84)
**2d**	94.00	71.28–123.81	Not active
**3a**	95.39	71.02–129.93	Not active
**3b**	95.80	81.07–120.81	Not active
**3c**	54.57	5.55–122.22	Leukemia: CCRF-CEM (22.23), HL-60(TB) (14.38), K-562 (26.34), MOLT-4 (7.23), RPMI-8226 (30.27); Non-small cell lung cancer: NCI-H23 (27.10); Colon cancer: HCT-15 (23.52); CNS cancer: SF-295 (30.63); Breast cancer: MCF7 (27.74), MDA-MB-468 (5.55)
**3d**	13.04	−91.75–130.07	Leukemia: CCRF-CEM (6.56), HL-60(TB) (−21.04), K-562 (4.78), MOLT-4 (−10.26), RPMI-8226 (4.56); Non-small cell lung cancer: EKVX (26.13), NCI-H23 (19.00); Colon cancer: COLO 205 (−74.16), HCT-116 (−52.25), HCT-15 (−34.49), HT29 (16.18), SW-620 (−62.07); Melanoma: LOX IMVI (−90.25), M14 (23.24), SK-MEL-28 (−84.21), SK-MEL-5 (10.35), UACC-257 (8.02); Renal cancer: 786–0 (−47.24), ACHN (−89.15), RXF 393 (−72.41); Prostate cancer: PC-3 (24.47); Breast cancer: MCF7 (0.08), MDA-MB-231/ATCC (5.52), BT-549 (16.70), T-47D (−64.38), MDA-MB-468 (−91.75)
**4a**	62.87	8.35–120.50	Leukemia: CCRF-CEM (26.59), HL-60(TB) (14.83), K-562 (29.77), MOLT-4 (8.35), RPMI-8226 (19.69), SR (17.58); Colon cancer: HCT-116 (24.61); Prostate cancer: PC-3 (15.70); Breast cancer: MCF7 (25.47), MDA-MB-468 (10.22)
**4b**	63.00	4.44–109.54	Leukemia: CCRF-CEM (15.58), HL-60(TB) (9.46), K-562 (24.90), MOLT-4 (27.78), RPMI-8226 (15.16), SR (4.44); Colon cancer: HCT-15 (18.05); Melanoma: LOX IMVI (24.03); Breast cancer: MCF7 (30.70)
**4c**	54.63	−75.11–120.11	Leukemia: CCRF-CEM (16.43), HL-60(TB) (2.73), K-562 (25.32), MOLT-4 (15.79), RPMI-8226 (19.80), SR (8.03); Colon cancer: HCT-15 (17.64); Melanoma: LOX IMVI (−75.11); Ovarian cancer: IGROV1 (18.24); Breast cancer: MCF7 (13.09), MDA-MB-468 (3.12)
**5a**	96.93	53.19–148.98	Not active
**5b**	93.20	45.12–139.93	Not active
**5c**	96.87	77.93–116.93	Not active
**6a**	96.91	68.74–156.30	Not active
**6c**	94.76	65.96–135.59	Not active
**7b**	97.61	74.39–122.20	Not active
**7c**	70.95	−0.15–139.14	Leukemia: CCRF-CEM (24.83), MOLT-4 (29.95); Ovarian cancer: OVCAR3 (31.67); Breast cancer: MDA-MB-468 (−0.15)
**8b**	85.76	28.01–126.65	Leukemia: RPMI-8226 (28.01);
**9d**	23.98	−98.89–84.16	Leukemia: CCRF-CEM (−98.89), HL-60(TB) (−98.56), MOLT-4 (−99.48), RPMI-8226 (−97.65); Non-small cell lung cancer: HOP-62 (−29.84) HOP-92 (−17.26); Colon cancer: KM-12 (−22.70); CNS cancer: SF-268 (19.57), SNB-75 (−8.25), Melanoma: MDA-MB-435 (23.74), SK-MEL-2 (−65.65); Ovarian cancer: IGROV1 (22.80), OVCAR-3 (26.94), Renal cancer: RXF-393 (−24.68); Prostate cancer: PC-3 (6.90); Breast cancer: MCF7 (−2.81), HS 578T (−13.39), BT-549 (15.85), MDA-MB-468 (−73.94)
**10d**	−1.86	−99.18–87.09	Leukemia: CCRF-CEM (−98.14), HL-60(TB) (−99.18), MOLT-4 (−99.21), RPMI-8226 (−98.36); Non-small cell lung cancer: EKVX (5.96), HOP-62 (−4.39), HOP-92 (−5.69), NCI-H226 (−3.96), NCI-H23 (−22.24), NCI-H522 (−98.70); Colon cancer: COLO 205 (22.31), HCT-116 (−4.09), HCT-15 (−10.28), KM-12 (−38.06), SW-620 (−60.75); CNS cancer: SF-268 (6.85), SF-539 (−5.90), SNB-19 (13.62), SNB-75 (−14.67), U251 (22.59); Melanoma: LOX IMVI (5.13), MALME-3M (−65.32), SK-MEL-2 (−71.35), UACC-257 (7.82), UACC-62 (22.38); Ovarian cancer: IGROV1 (26.89), OVCAR-3 (−34.84), OVCAR-4 (−20.86), OVCAR-8 (31.67); Renal cancer: ACHN (11.90), CAKI-1 (−66.46), RXF-393 (−76.56), SN12C (30.34), TK-10 (12.97); Prostate cancer: PC-3 (−10.13); Breast cancer: MCF7 (−13.16), HS 578T (−30.68), BT-549 (−63.49), MDA-MB-468 (−87.09)

**Table 2:** In vitro cytotoxic effects of compounds **3d, 9d and 10d** and standard drugs doxorubicin and 5-fluorouracil against NCI’s human tumor cell line screen in a five-dose analysis.

Cancer Cell Lines	Antiproliferative Activity In Vitro, μM										
	3d			9d			10d			DRB	5-FU
	GI_50_	TGI	LC_50_	GI_50_	TGI	LC_50_	GI_50_	TGI	LC_50_	GI_50_	GI_50_
Leukemia											
CCRF-CEM	0.42	2.56	54.9	1.21	2.13	3.73	1.6	2.97	5.51	0.08	9.97
HL-60(TB)	1.98	5.56	>100	2.58	8.91	23.3	1.31	2.60	5.17	0.19	2.30
K-562	2.26	>100	>100	17.1	>60	>60	2.16	4.52	9.45	-	3.58
MOLT-4	3.15	11.1	98.4	1.23	2.12	3.65	1.75	3.15	5.66	0.03	0.35
RPMI-8226	3.01	10.0	>100	7.26	15.8	34.3	1.98	4.18	8.85	0.08	0.04
SR	2.48	7.99	>100	3.63	11.0	26.3	2.05	3.86	7.29	0.03	-
Non-Small Cell Lung Cancer											
A549/ATCC	10.1	23.8	56.1	17.7	>60	>60	7.07	24.6	71.0	0.06	0.18
EKVX	7.99	20.9	46.8	-	-	-	-	-	-	0.41	-
HOP-62	13.9	31.5	71.3	25.5	34.5	>60	25.2	50.2	>100	0.07	0.39
HOP-92	4.65	18.0	50.3	8.65	>60	>60	4.56	15.3	39.2	0.10	77.9
NCI-H226	8.25	23.5	58.9	39.7	>60	>60	12.9	29.3	66.4	0.05	54.7
NCI-H23	9.17	22.5	52.3	26.8	>60	>60	3.51	12.4	35.5	0.15	0.33
NCI-H322M	15.5	29.4	55.5	>60	>60	>60	23.1	56.4	>100	-	-
NCI-H460	9.89	22.5	50.9	31.6	>60	>60	15.2	28.7	54.3	0.02	0.05
NCI-H522	3.07	11.9	37.5	37.2	>60	>60	1.81	3.22	5.74	0.03	7.27
Colon Cancer											
COLO 205	3.68	11.8	45.8	43.2	>60	>60	3.81	10.7	32.8	0.18	0.15
HCC-2998	13.1	26.5	53.3	>60	>60	>60	19.3	39.2	79.7	0.26	0.05
HCT-116	2.34	5.96	>100	16.4	>60	>60	2.68	7.99	28.0	0.08	0.22
HCT-15	3.46	14.5	44.5	22.6	>60	>60	9.20	21.5	47.5	6.46	0.11
HT29	2.20	4.96	16.2	29.6	>60	>60	13.9	27.0	52.6	0.12	0.17
KM12	7.74	20.6	4.81	23.0	>60	>60	13.5	26.7	52.8	0.27	0.21
SW-620	1.48	2.99	6.05	27.8	>60	>60	1.83	3.34	6.11	0.09	0.92
CNS Cancer											
SF-268	11.0	25.3	58.3	18.7	>60	>60	5.60	18.6	43.5	0.10	1.62
SF-295	14.0	27.6	54.6	43.9	>60	>60	20.1	81.9	>100	0.10	-
SF-539	16.1	29.9	55.4	27.9	>60	>60	3.80	12.5	35.5	0.12	0.06
SNB-19	13.3	26.1	51.3	>60	>60	>60	5.37	17.6	43.1	0.04	3.81
SNB-75	5.12	18.6	47.0	22.9	>60	>60	5.59	18.3	43.4	0.07	78.7
U251	8.09	21.8	51.2	30.8	>60	>60	3.67	13.2	37.2	0.04	0.92
Melanoma											
LOX IMVI	1.81	3.75	7.76	24.5	>60	>60	3.16	11.6	34.7	0.07	0.24
MALME-3M	1.73	3.42	6.78	>60	>60	>60	12.6	25.1	50.2	0.12	0.05
M14	3.60	16.0	65.4	>60	>60	>60	7.29	19.9	44.7	0.18	0.98
MDA-MB-435	4.96	17.6	43.8	55.9	>60	>60	17.8	32.5	59.3	0.25	0.07
SK-MEL-2	10.5	23.2	51.6	14.2	35.8	>60	2.73	8.73	29.5	0.17	56.7
SK-MEL-28	3.68	13.9	38.0	42.6	>60	>60	11.4	23.7	49.1	0.21	1.03
SK-MEL-5	3.77	15.4	40.5	13.7	>60	>60	10.3	22.0	47.1	0.08	0.46
UACC-257	5.51	19.1	46.8	26.9	>60	>60	6.31	18.2	42.7	0.14	3.55
UACC-62	4.16	18.0	43.9	50.8	>60	>60	5.04	15.6	39.6	0.12	0.52
Ovarian Cancer											
IGROV1	6.14	20.3	53.9	>60	>60	>60	17.3	31.8	58.5	0.17	1.22
OVCAR-3	1.56	3.22	6.65	17.2	>60	>60	5.17	17.3	41.8	0.39	0.01
OVCAR-4	4.39	17.9	49.4	16.0	59.9	>60	2.95	7.85	27.0	0.37	4.43
OVCAR-5	13.9	27.4	53.9	54.1	>60	>60	15.3	3.49	79.6	0.41	10.9
OVCAR-8	11.0	26.0	61.2	58.6	>60	>60	11.1	2.38	51.0	0.10	1.74
NCI/ADR-RES	4.67	18.8	54.8	39.2	>60	>60	13.6	2.78	56.5	7.16	0.31
SK-OV-3	14.8	30.9	64.6	22.3	>60	>60	12.2	2.77	63.0	0.22	21.8
Renal Cancer											
786-0	2.18	5.38	39.8	30.6	>60	>60	12.3	24.8	50.0	0.13	0.72
A498	16.9	30.7	55.8	35.4	>60	>60	13.9	31.1	69.7	0.10	0.35
ACHN	1.66	3.27	6.42	>60	>60	>60	5.53	17.7	42.2	0.08	0.27
CAKI-1	1.52	3.46	7.92	27.0	>60	>60	4.53	16.9	4.13	0.95	0.07
RXF 393	2.06	5.01	17.7	-	-	-	-	-	-	0.10	2.61
SN12C	8.62	21.9	50.1	44.6	>60	>60	13.0	25.7	51.0	0.07	0.49
TK-10	7.72	20.6	46.3	58.9	>60	>60	13.3	26.2	51.7	-	1.12
UO-31	1.52	3.16	6.58	35.5	>60	>60	3.58	12.4	35.4	0.49	1.42
Prostate Cancer											
PC-3	3.85	16.8	57.8	11.6	46.0	>60	6.85	20.3	49.4	0.32	2.36
DU-145	6.86	19.7	44.7	>60	>60	>60	16.4	30.2	55.9	0.11	0.36
Breast Cancer											
MCF7	3.04	10.7	54.2	18.0	>60	>60	7.17	19.5	44.7	0.03	0.07
MDA-MB-31/ATCC	6.25	20.2	50.3	21.0	56.1	>60	19.0	33.3	58.3	0.51	6.60
HS 578T	9.09	37.4	>100	17.4	>60	>60	3.08	12.3	35.4	0.33	9.77
BT-549	6.78	23.9	67.2	27.8	>60	>60	2.62	7.43	26.4	0.23	10.6
T-47D	1.71	4.19	>100	8.64	17.8	36.7	1.75	3.91	8.75	0.06	8.12
MDA-MB-468	2.05	4.61	11.5	4.74	11.8	27.0	1.09	2.56	6.00	0.05	-

**Table 3:** Average values of GI_50_ (µM) and selectivity ratios of compounds **3d**, **9d** and **10d** against NCI subpanels in five-dose analysis.

Subpanel	Compound (Mean GI_50_)/(SI)		
	3d	9d	10d
Leukemia	2.22/2.77	5.50/5.49	1.80/4.72
NSCL Cancer	9.17/0.67	30.89/0.98	11.67/0.73
Colon Cancer	4.86/1.27	31.80/0.95	9.17/0.93
CNS Cancer	11.27/0.55	34.03/0.89	7.36/1.15
Melanoma	4.41/1.39	38.73/0.78	8.51/0.99
Ovarian Cancer	8.07/0.76	36.77/0.82	11.09/0.77
Renal Cancer	5.27/1.17	41.71/0.72	9.42/0.90
Prostate Cancer	5.35/1.15	35.80/0.84	11.62/0.73
Breast Cancer	4.82/1.28	16.26/1.86	5.79/1.47
Full Panel	6.26	30.17	8.49

**Table 4:** Standard agent COMPARE correlations for compounds **3d, 9d and 10d** according to five-dose assays.

Compound	Vector		
	GI_50_	TGI	LC_50_
**3d**	Eprenatapopt r = 0.65 (58) [a] 3-Bromopyruvic acid r = 0.55 (59) Ixazomib r = 0.52 (58)	Eprenetapopt 0.58 (58) Olmutinib 0.57 (55) Tepotinib 0.54 (59) Sunitinib 0.51 (59)	Tepotinib 0.42 (59)
**9d**	6-mercaptopurine 0.42 (59)	Regorafenib 0.37 (57)	Bendamustine 0.21 (59)
**10d**	6-mercaptopurine 0.41 (59)	Regorafenib 0.34 (57)	Lapatinib 0.18 (59)

[a] r—correlation coefficient (number of standard cell lines).

**Table 5:** In silico ADMET-selected characteristics of compounds **3d**, **9d** and **10d**, including probability ^[a]^.

ADMET Predicted Profile	3d		9d		10d		Doxorubicin	
	Value	Probability	Value	Probability	Value	Probability	Value	Probability
cLogP	3.1959		5.8837		5.8247		0.1673	
F	>50%	0.894	>50%	0.995	>50%	0.52	<20%	0.959
Drug likeness	3.9501		3.4304		1.5298		6.6484	
Predicted toxicity class	6		5		5		3	
Hepatotoxicity	−	0.86	−	0.82	−	0.80	−	0.86
Neurotoxicity	+	0.58	−	0.59	−	0.57	+	0.74
Nephrotoxicity	+	0.56	−	0.67	+	0.54	+	0.80
Respiratory toxicity	+	0.85	+	0.86	+	0.87	+	0.91
Cardiotoxicity	−	0.79	−	0.71	−	0.54	+	0.64
Immunotoxicity	+	0.91	+	0.85	+	0.60	+	0.99
Mutagenicity	−	0.65	−	0.74	−	0.73	+	0.98
BCRP inhibitor	−	~1	−	~1	−	~1	−	~1
P-glycoprotein inhibitor	−	0.99	−	0.79	+	0.81	−	~1
P-glycoprotein substrate	−	~1	−	~1	−	~1	+	0.99
AR-LBD	−	0.98	−	0.97	−	0.95	−	0.55
Aromatase	−	0.80	−	0.86	−	0.86	+	0.52
Estrogen receptor	−	0.95	−	0.95	−	0.92	−	0.74
THRα	−	0.90	−	0.90	−	0.90	−	0.90
THRβ	−	0.78	−	0.78	−	0.78	−	0.78
TTR	−	0.97	−	0.97	−	0.97	−	0.97
RYR	−	0.98	−	0.98	−	0.98	−	0.98
GABAR	−	0.96	−	0.96	−	0.96	−	0.96
AChE	−	0.86	−	0.82	−	0.80	−	0.86
PPAR-γ	−	0.95	−	0.95	−	0.93	−	0.97
Nrf2/ARE	−	0.89	−	0.88	−	0.89	−	0.98
HSE	−	0.89	−	0.88	−	0.89	−	0.98
MMP	−	0.78	−	0.82	−	0.76	−	0.56
ATAD5	−	0.94	−	0.96	−	0.92	−	0.63
NIS	−	0.98	−	0.98	−	0.98	−	0.98
BBB	+	0.93	+	0.99	+	0.93	−	~1
BSEP inhibitor	+	~1	+	~1	+	~1	−	0.81
Caco-2	−5.27		−4.96		−5.09		−6.26	
CYP1A2	−	0.99	−	0.96	−	0.97	−	0.99
CYP2C19	−	0.91	−	0.82	−	0.78	−	0.97
CYP2C9	−	0.68	−	0.55	+	0.52	−	0.73
CYP2D6	−	0.72	−	0.68	−	0.73	−	0.92
CYP3A4	−	0.70	−	0.61	+	0.81	−	0.98
CYP2E1	−	0.99	−	~1	−	~1	−	0.99

^[a]^ “− “—negative prediction, “+”—positive prediction.

## Data Availability

Data are contained within the article and Supplementary Materials.

## Supplementary Materials

The following supporting information can be downloaded at: https://www.mdpi.com/article/10.3390/molecules29153532/s1, Pages S1 and S2: Biological assay, Figures S1–S20: One-dose mean graph of NCI-60 cell line screening data for compounds, Figures S21–S38: Anticancer screening data of compounds at five-dose assay, Figure S39: One-dose mean graph of NCI-60 cell line screening data for compound **1d**, Figures S40–S75: NMR spectra of products. Reference [51] is cited in the supplementary materials.
