# Supplementary material for: Derivatization of Abietane Acids by Peptide-like Substituents Leads to Submicromolar Cytotoxicity at NCI-60 Panel
Source: Molecules. 2024 Jul 27;29(15):3532. doi: 10.3390/molecules29153532 (PMC11313996; doi:10.3390/molecules29153532)

# Supporting Information

## Derivatization of Abietane Acids by Peptide-like Substituents Leads to Submicromolar Cytotoxicity at NCI-60 Panel

Elena Tretyakova [1,]*, Anna Smirnova [1], Denis Babkov[2] and Oxana Kazakova [1]

[1] Ufa Institute of Chemistry of the Ufa Federal Research Centre of the Russian Academy of Sciences, 71 Prospect Oktyabrya, 450054, Ufa, Russian Federation; tretyakovaelv@gmail.com (E.T.); bazunova03@yandex.ru (A.S.), obf@anrb.ru (O.K.)

[2] Scientific Center for Innovative Drugs, Volgograd State Medical University, Novorossiyskaya st. 39, 400087 Volgograd, Russian Federation; denis.a.babkov@gmail.com (D.B.)

* Correspondence: tretyakovaelv@gmail.com (E.T.)

Supporting Information

Table of contents


| | |
|---|---|
| Biological assay | S1-S2 |
| Figures S1-S20. One dose mean graph of NCI-60 cell line screening data for compounds | S3-S22 |
| Figures S21-S38. Anticancer screening data of compounds at a 5-dose assay | S23-S40 |
| Figure S39. One dose mean graph of NCI-60 cell line screening data for compound 1d | S41 |
| Figures S40-S75. $^{1}H$ and $^{13}C$ NMR Spectra of Products | S42-S61 |

## Biological assay

Anticancer activity study was studied through the National Institute of Health's (NIH) NCI-60 human cancer cell line screening program [1] at the Chemotherapeutic Agents Repository, c/o Fisher Bio Services, 20301, Century Boulevard, Building 6, Suite 800, Germantown, MD 20874, United States.

### In vitro anticancer screening of the tested compounds

#### One dose assay

Synthesized compounds 2a, 2b, 2d, 3a, 3b, 3c, 3d, 4a, 4b, 4c, 5a, 5b, 5c, 6a, 6c, 7b, 7c, 8b, 9d, 10d were investigated in the National Cancer Institute NCI, Bethesda, Maryland, U.S.A. under the Developmental Therapeutic Program DTP. Primary in vitro one dose anticancer screening was initiated by cell inoculating into a series of standard 96-well microtiter plates at 5000-40000 cells/well in RPMI 1640 medium containing 5% fetal bovine serum and 2 µM L-glutamine (day 0), and then preincubated in absence of drug at 37 °C and 5% $CO_2$ for 24 h. Test compounds were then added into the plates at one concentration of $10^{-5}$ M (day 1) followed by incubation for a further 48 h at the same conditions. Then the media were removed, the cells were fixed in situ, washed, and dried (day 3). The sulforhodanine B assay was used for cell density determination, based on the measurement of cellular protein content. After an incubation period, cell monolayers were fixed with 10% (wt/vol) trichloroacetic acid and stained for 30 min, after which the excess dye was removed by washing repeatedly with 1% (vol/vol) acetic acid. The bound stain was resolubilized in 10 µM Tris base solution and measured spectrophotometrically on automated microplate readers for OD determination at 510 nm.

#### Five doses assay

Compounds exhibited significant growth inhibition in the one dose screen were evaluated against the 60-cell panel at five concentration levels (0.01, 0.1, 1, 10 and 100 µM). The outcomes were used to create three dose-response parameters ($GI_{50}$, TGI and $LC_{50}$) calculated for each cell line. The $GI_{50}$ value (50% growth inhibition) is measure of the sensitivity of a cells to the effect of the drug and corresponds to the concentration of

the compound causing 50% decrease in net cell growth. The TGI (total growth inhibition) refers to the maximum effect of a drug and it is the concentration of the study drug that causes total inhibition of cell growth. The $LC_{50}$ value (cytotoxic activity) is the concentration of the compound causing net 50% loss of initial cells at the end of the



incubation period of 48 h. The three dose-response parameters $GI_{50}$, TGI and $LC_{50}$ were calculated for each experimental compound. Data calculations were performed according to the method described by the NCI/NIH Development Therapeutics Program (https://dtp.cancer.gov/discovery_development/nci-60/methodology.htm).

Growth inhibition of 50% ($GI_{50}$) is calculated from: $[(T–T0)/(C–T_0)] \times 100 = 50$. The TGI is calculated from: $100\times(T–T_0)/(C–T_0) = 0$. Thus, the TGI signifies a cytostatic effect. The $LC_{50}$, which means a cytotoxic effect, is calculated as: $[(T–T_0)/T_0] \times 100 = –50$, where: $T_0$ is the cell count at day 0; C is the vehicle control (cell count without drug), and T is the cell count at the test concentration of drug at the end of the incubation period.

NCI 60 cell panel COMPARE correlations

The graph of mean values for each of compounds was subsequently used to run the COMPARE algorithm from the Developmental Therapeutics Program, NCI, and calculate the correlation coefficient with respect to compounds from the standard agent database with a known mechanism of action (https://dtp.cancer.gov/databases_tools/compare.htm). Pairwise correlation coefficients of greater than 0.3 were used as the cut-off for assessing whether two agents were likely to share a similar mechanism of action. Briefly, vectors of $GI_{50}$, TGI, and $LC_{50}$ concentrations for tested compound were correlated with the set of average $GI_{50}$, TGI, and $LC_{50}$ vectors for all public NCI-60 vectors for the full public standard agent's database.

[1] Monga, M.; Sausville, E. A. Developmental Therapeutics Program at the NCI: Molecular Target and Drug Discovery Process. Leukemia 2002, 16 (4), 520–526

Figure S1. One dose mean graph of NCI-60 cell line screening data for 2a.

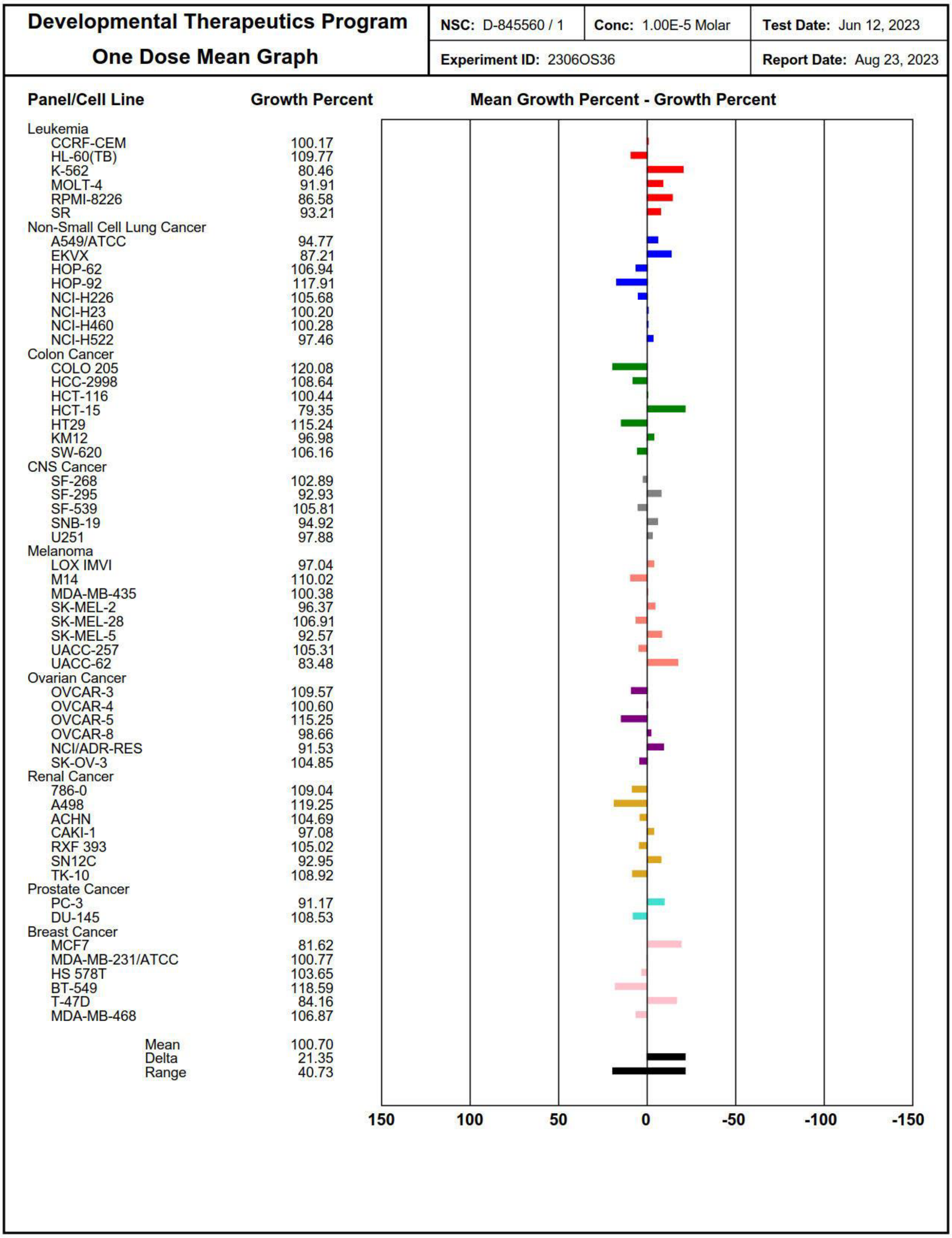

**Developmental Therapeutics Program**
**One Dose Mean Graph**

**NSC:** D-845560 / 1 | **Conc:** 1.00E-5 Molar | **Test Date:** Jun 12, 2023
**Experiment ID:** 2306OS36 | **Report Date:** Aug 23, 2023

| Panel/Cell Line | Growth Percent |
|---|---|
| Leukemia | |
| CCRF-CEM | 100.17 |
| HL-60(TB) | 109.77 |
| K-562 | 80.46 |
| MOLT-4 | 91.91 |
| RPMI-8226 | 86.58 |
| SR | 93.21 |
| Non-Small Cell Lung Cancer | |
| A549/ATCC | 94.77 |
| EKVX | 87.21 |
| HOP-62 | 106.94 |
| HOP-92 | 117.91 |
| NCI-H226 | 105.68 |
| NCI-H23 | 100.20 |
| NCI-H460 | 100.28 |
| NCI-H522 | 97.46 |
| Colon Cancer | |
| COLO 205 | 120.08 |
| HCC-2998 | 108.64 |
| HCT-116 | 100.44 |
| HCT-15 | 79.35 |
| HT29 | 115.24 |
| KM12 | 96.98 |
| SW-620 | 106.16 |
| CNS Cancer | |
| SF-268 | 102.89 |
| SF-295 | 92.93 |
| SF-539 | 105.81 |
| SNB-19 | 94.92 |
| U251 | 97.88 |
| Melanoma | |
| LOX IMVI | 97.04 |
| M14 | 110.02 |
| MDA-MB-435 | 100.38 |
| SK-MEL-2 | 96.37 |
| SK-MEL-28 | 106.91 |
| SK-MEL-5 | 92.57 |
| UACC-257 | 105.31 |
| UACC-62 | 83.48 |
| Ovarian Cancer | |
| OVCAR-3 | 109.57 |
| OVCAR-4 | 100.60 |
| OVCAR-5 | 115.25 |
| OVCAR-8 | 98.66 |
| NCI/ADR-RES | 91.53 |
| SK-OV-3 | 104.85 |
| Renal Cancer | |
| 786-0 | 109.04 |
| A498 | 119.25 |
| ACHN | 104.69 |
| CAKI-1 | 97.08 |
| RXF 393 | 105.02 |
| SN12C | 92.95 |
| TK-10 | 108.92 |
| Prostate Cancer | |
| PC-3 | 91.17 |
| DU-145 | 108.53 |
| Breast Cancer | |
| MCF7 | 81.62 |
| MDA-MB-231/ATCC | 100.77 |
| HS 578T | 103.65 |
| BT-549 | 118.59 |
| T-47D | 84.16 |
| MDA-MB-468 | 106.87 |
| Mean | 100.70 |
| Delta | 21.35 |
| Range | 40.73 |

Figure S2. One dose mean graph of NCI-60 cell line screening data for 2b

| Developmental Therapeutics Program | NSC: D-845561 / 1 | Conc: 1.00E-5 Molar | Test Date: Jun 12, 2023 |
|---|---|---|---|
| One Dose Mean Graph | Experiment ID: 2306OS36 | | Report Date: Aug 23, 2023 |

| Panel/Cell Line | Growth Percent |
|---|---|
| **Leukemia** | |
| CCRF-CEM | 26.08 |
| HL-60(TB) | -6.49 |
| K-562 | 20.12 |
| MOLT-4 | 7.97 |
| RPMI-8226 | 36.18 |
| SR | 52.09 |
| **Non-Small Cell Lung Cancer** | |
| A549/ATCC | 64.13 |
| EKVX | 54.75 |
| HOP-62 | 91.56 |
| HOP-92 | 89.36 |
| NCI-H226 | 86.05 |
| NCI-H23 | 46.41 |
| NCI-H460 | 59.93 |
| NCI-H522 | 47.03 |
| **Colon Cancer** | |
| COLO 205 | 86.72 |
| HCC-2998 | 62.25 |
| HCT-116 | 55.15 |
| HCT-15 | 41.70 |
| HT29 | 83.78 |
| KM12 | 58.67 |
| SW-620 | 67.80 |
| **CNS Cancer** | |
| SF-268 | 75.19 |
| SF-295 | 43.76 |
| SF-539 | 62.35 |
| SNB-19 | 59.04 |
| U251 | 48.13 |
| **Melanoma** | |
| LOX IMVI | 35.95 |
| M14 | 81.64 |
| MDA-MB-435 | 85.37 |
| SK-MEL-2 | 79.92 |
| SK-MEL-28 | 70.51 |
| SK-MEL-5 | 73.21 |
| UACC-257 | 88.27 |
| UACC-62 | 54.80 |
| **Ovarian Cancer** | |
| OVCAR-3 | 42.21 |
| OVCAR-4 | 70.23 |
| OVCAR-5 | 111.11 |
| OVCAR-8 | 68.89 |
| NCI/ADR-RES | 56.85 |
| SK-OV-3 | 89.64 |
| **Renal Cancer** | |
| 786-0 | 99.05 |
| A498 | 122.05 |
| ACHN | 48.18 |
| CAKI-1 | 54.97 |
| RXF 393 | 66.56 |
| SN12C | 69.88 |
| TK-10 | 101.27 |
| **Prostate Cancer** | |
| PC-3 | 54.44 |
| DU-145 | 84.62 |
| **Breast Cancer** | |
| MCF7 | 43.36 |
| MDA-MB-231/ATCC | 78.43 |
| HS 578T | 63.41 |
| BT-549 | 88.58 |
| T-47D | 50.98 |
| MDA-MB-468 | -4.84 |
| Mean | 62.71 |
| Delta | 69.20 |
| Range | 128.54 |

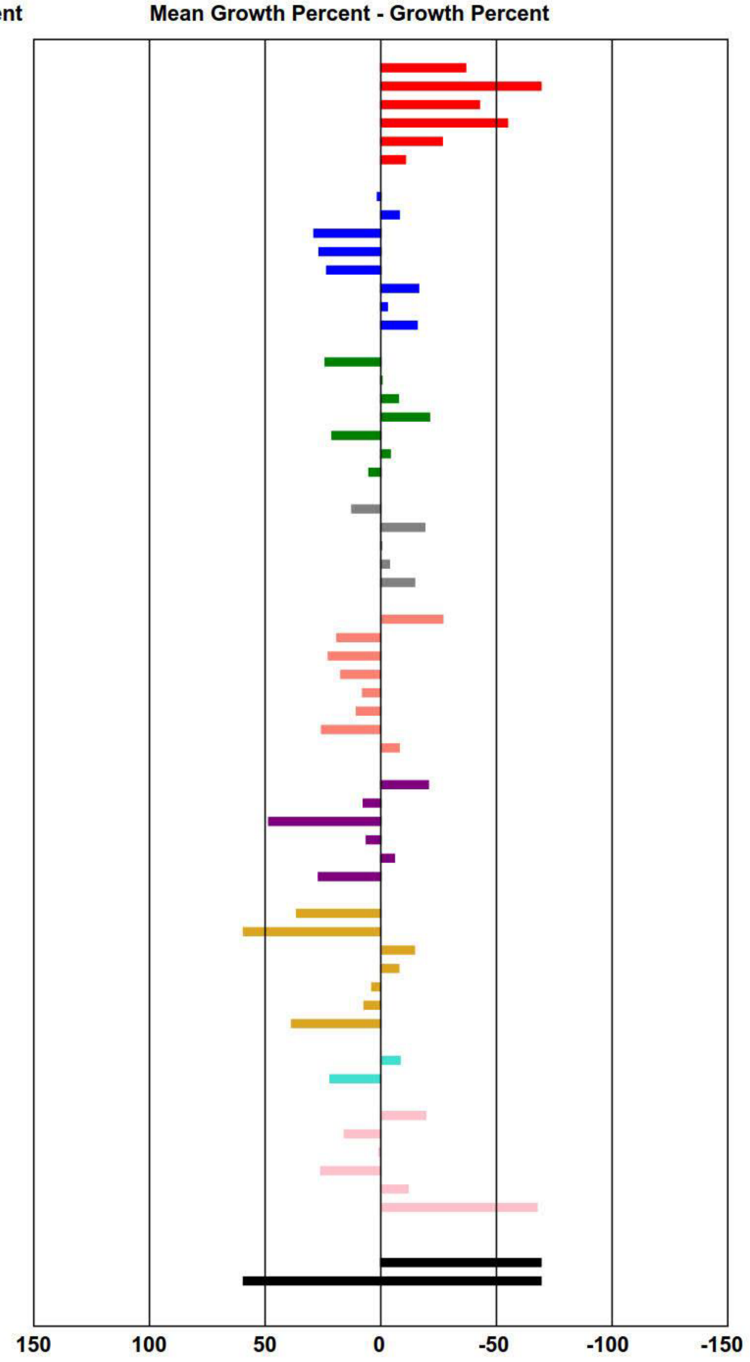

Figure S3. One dose mean graph of NCI-60 cell line screening data for 2d

| **Developmental Therapeutics Program** | **NSC:** D-845562 / 1 | **Conc:** 1.00E-5 Molar | **Test Date:** Jun 12, 2023 |
|---|---|---|---|
| **One Dose Mean Graph** | **Experiment ID:** 2306OS36 | | **Report Date:** Aug 23, 2023 |

| Panel/Cell Line | Growth Percent |
|---|---|
| Leukemia | |
| CCRF-CEM | 82.14 |
| HL-60(TB) | 96.14 |
| K-562 | 86.42 |
| MOLT-4 | 81.97 |
| RPMI-8226 | 65.93 |
| Non-Small Cell Lung Cancer | |
| A549/ATCC | 101.99 |
| EKVX | 90.58 |
| HOP-62 | 94.27 |
| HOP-92 | 97.38 |
| NCI-H226 | 83.85 |
| NCI-H23 | 92.51 |
| NCI-H460 | 100.81 |
| NCI-H522 | 100.44 |
| Colon Cancer | |
| COLO 205 | 120.07 |
| HCC-2998 | 89.04 |
| HCT-116 | 98.66 |
| HCT-15 | 76.13 |
| HT29 | 107.07 |
| KM12 | 94.20 |
| SW-620 | 104.39 |
| CNS Cancer | |
| SF-268 | 102.93 |
| SF-295 | 82.36 |
| SF-539 | 92.51 |
| SNB-19 | 84.57 |
| U251 | 101.64 |
| Melanoma | |
| LOX IMVI | 88.38 |
| M14 | 99.34 |
| MDA-MB-435 | 96.69 |
| SK-MEL-2 | 99.87 |
| SK-MEL-28 | 105.29 |
| SK-MEL-5 | 95.42 |
| UACC-257 | 102.80 |
| UACC-62 | 87.93 |
| Ovarian Cancer | |
| OVCAR-3 | 123.81 |
| OVCAR-4 | 95.15 |
| OVCAR-5 | 106.94 |
| OVCAR-8 | 99.28 |
| NCI/ADR-RES | 78.25 |
| SK-OV-3 | 100.42 |
| Renal Cancer | |
| 786-0 | 96.26 |
| A498 | 111.57 |
| ACHN | 91.87 |
| CAKI-1 | 84.48 |
| RXF 393 | 89.14 |
| SN12C | 94.09 |
| TK-10 | 104.27 |
| Prostate Cancer | |
| PC-3 | 79.60 |
| DU-145 | 104.49 |
| Breast Cancer | |
| MCF7 | 82.51 |
| MDA-MB-231/ATCC | 96.29 |
| HS 578T | 86.66 |
| BT-549 | 92.54 |
| T-47D | 83.15 |
| MDA-MB-468 | 71.28 |
| Mean | 94.00 |
| Delta | 28.07 |
| Range | 57.88 |

**Mean Growth Percent - Growth Percent**

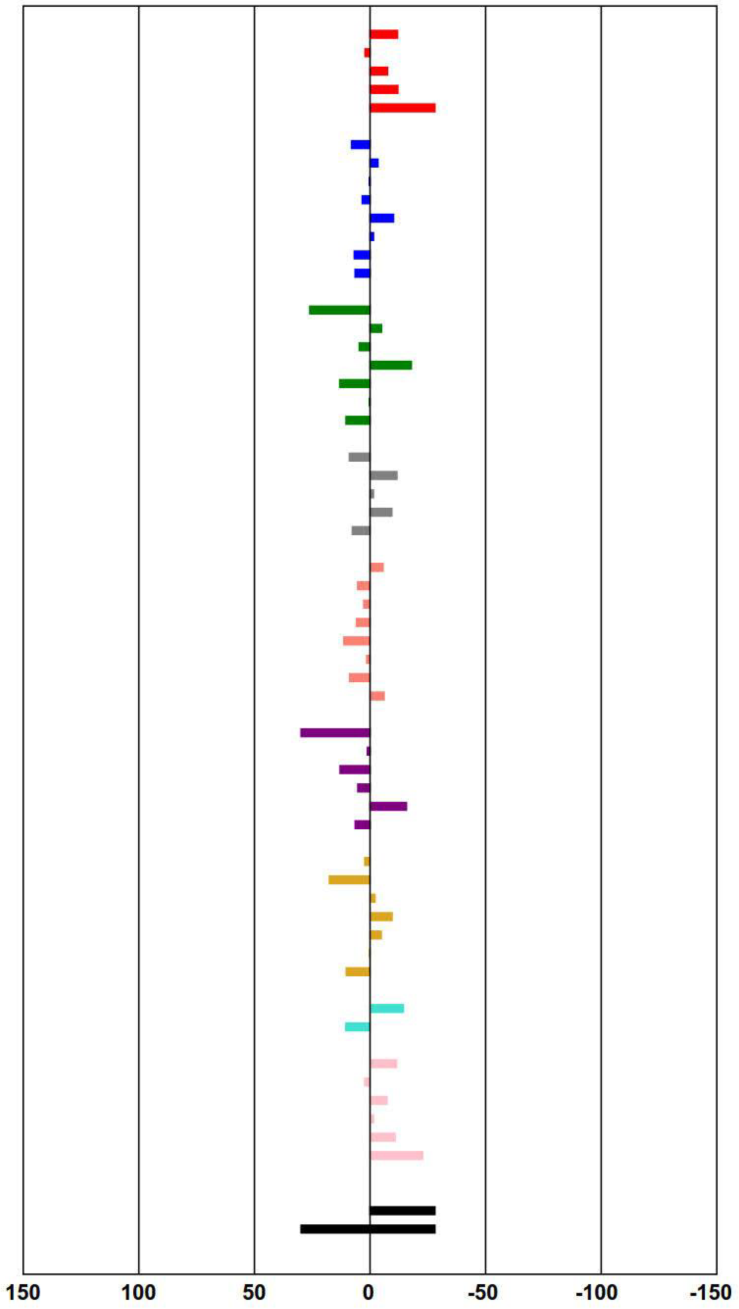

Figure S4. One dose mean graph of NCI-60 cell line screening data for 3a

| Developmental Therapeutics Program One Dose Mean Graph | NSC: D-845566 / 1 | Conc: 1.00E-5 Molar | Test Date: Jun 12, 2023 |
|---|---|---|---|
| | Experiment ID: 2306OS36 | | Report Date: Aug 23, 2023 |

| Panel/Cell Line | Growth Percent |
|---|---|
| Leukemia | |
| CCRF-CEM | 91.62 |
| HL-60(TB) | 104.18 |
| K-562 | 72.31 |
| MOLT-4 | 86.20 |
| RPMI-8226 | 71.02 |
| Non-Small Cell Lung Cancer | |
| A549/ATCC | 92.33 |
| EKVX | 88.12 |
| HOP-62 | 95.49 |
| HOP-92 | 115.50 |
| NCI-H226 | 86.41 |
| NCI-H23 | 94.15 |
| NCI-H460 | 96.70 |
| NCI-H522 | 91.17 |
| Colon Cancer | |
| COLO 205 | 120.39 |
| HCC-2998 | 103.07 |
| HCT-116 | 94.55 |
| HCT-15 | 87.35 |
| HT29 | 104.87 |
| KM12 | 96.19 |
| SW-620 | 106.37 |
| CNS Cancer | |
| SF-268 | 91.50 |
| SF-295 | 88.80 |
| SF-539 | 94.73 |
| SNB-19 | 90.31 |
| U251 | 91.07 |
| Melanoma | |
| LOX IMVI | 90.35 |
| M14 | 94.39 |
| MDA-MB-435 | 96.34 |
| SK-MEL-2 | 96.47 |
| SK-MEL-28 | 110.15 |
| SK-MEL-5 | 91.44 |
| UACC-257 | 98.63 |
| UACC-62 | 80.83 |
| Ovarian Cancer | |
| OVCAR-3 | 112.57 |
| OVCAR-4 | 89.78 |
| OVCAR-5 | 122.74 |
| OVCAR-8 | 98.08 |
| NCI/ADR-RES | 87.89 |
| SK-OV-3 | 95.33 |
| Renal Cancer | |
| 786-0 | 105.64 |
| A498 | 129.93 |
| ACHN | 86.68 |
| CAKI-1 | 87.67 |
| RXF 393 | 89.01 |
| SN12C | 96.97 |
| TK-10 | 112.58 |
| Prostate Cancer | |
| PC-3 | 83.84 |
| DU-145 | 104.80 |
| Breast Cancer | |
| MCF7 | 76.75 |
| MDA-MB-231/ATCC | 101.19 |
| HS 578T | 85.61 |
| BT-549 | 96.88 |
| T-47D | 86.34 |
| MDA-MB-468 | 87.71 |
| Mean | 95.39 |
| Delta | 24.37 |
| Range | 58.91 |

Mean Growth Percent - Growth Percent

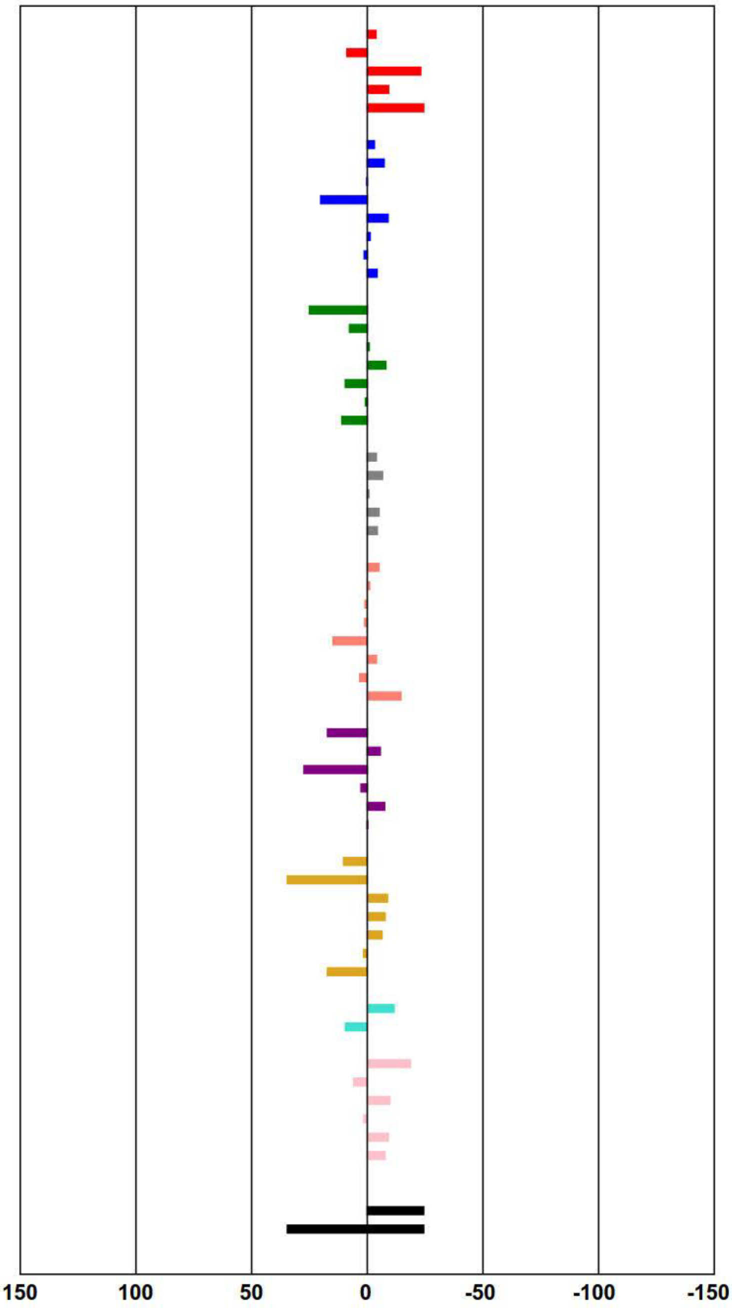

Figure S5. One dose mean graph of NCI-60 cell line screening data for 3b

| Developmental Therapeutics Program One Dose Mean Graph | NSC: D-845563 / 1 | Conc: 1.00E-5 Molar | Test Date: Jun 12, 2023 |
|---|---|---|---|
| | Experiment ID: 2306OS36 | | Report Date: Aug 23, 2023 |

| Panel/Cell Line | Growth Percent |
|---|---|
| **Leukemia** | |
| CCRF-CEM | 89.54 |
| HL-60(TB) | 98.65 |
| K-562 | 86.26 |
| MOLT-4 | 84.50 |
| RPMI-8226 | 81.07 |
| **Non-Small Cell Lung Cancer** | |
| A549/ATCC | 100.42 |
| EKVX | 89.25 |
| HOP-62 | 95.45 |
| HOP-92 | 110.58 |
| NCI-H226 | 90.77 |
| NCI-H23 | 95.90 |
| NCI-H460 | 103.07 |
| NCI-H522 | 100.98 |
| **Colon Cancer** | |
| COLO 205 | 117.05 |
| HCC-2998 | 90.57 |
| HCT-116 | 99.41 |
| HCT-15 | 83.17 |
| HT29 | 108.50 |
| KM12 | 94.78 |
| SW-620 | 106.56 |
| **CNS Cancer** | |
| SF-268 | 101.35 |
| SF-295 | 83.93 |
| SF-539 | 93.63 |
| SNB-19 | 85.68 |
| U251 | 109.93 |
| **Melanoma** | |
| LOX IMVI | 85.47 |
| M14 | 98.49 |
| MDA-MB-435 | 98.52 |
| SK-MEL-2 | 101.82 |
| SK-MEL-28 | 109.96 |
| SK-MEL-5 | 96.87 |
| UACC-257 | 99.82 |
| UACC-62 | 88.63 |
| **Ovarian Cancer** | |
| OVCAR-3 | 120.81 |
| OVCAR-4 | 91.09 |
| OVCAR-5 | 115.50 |
| OVCAR-8 | 101.07 |
| NCI/ADR-RES | 83.62 |
| SK-OV-3 | 100.67 |
| **Renal Cancer** | |
| 786-0 | 94.17 |
| A498 | 60.78 |
| ACHN | 96.93 |
| CAKI-1 | 84.25 |
| RXF 393 | 91.36 |
| SN12C | 98.88 |
| TK-10 | 107.66 |
| **Prostate Cancer** | |
| PC-3 | 95.60 |
| DU-145 | 112.95 |
| **Breast Cancer** | |
| MCF7 | 84.86 |
| MDA-MB-231/ATCC | 98.45 |
| HS 578T | 89.61 |
| BT-549 | 94.89 |
| T-47D | 87.30 |
| MDA-MB-468 | 82.16 |
| Mean | 95.80 |
| Delta | 35.02 |
| Range | 60.03 |

**Mean Growth Percent - Growth Percent**

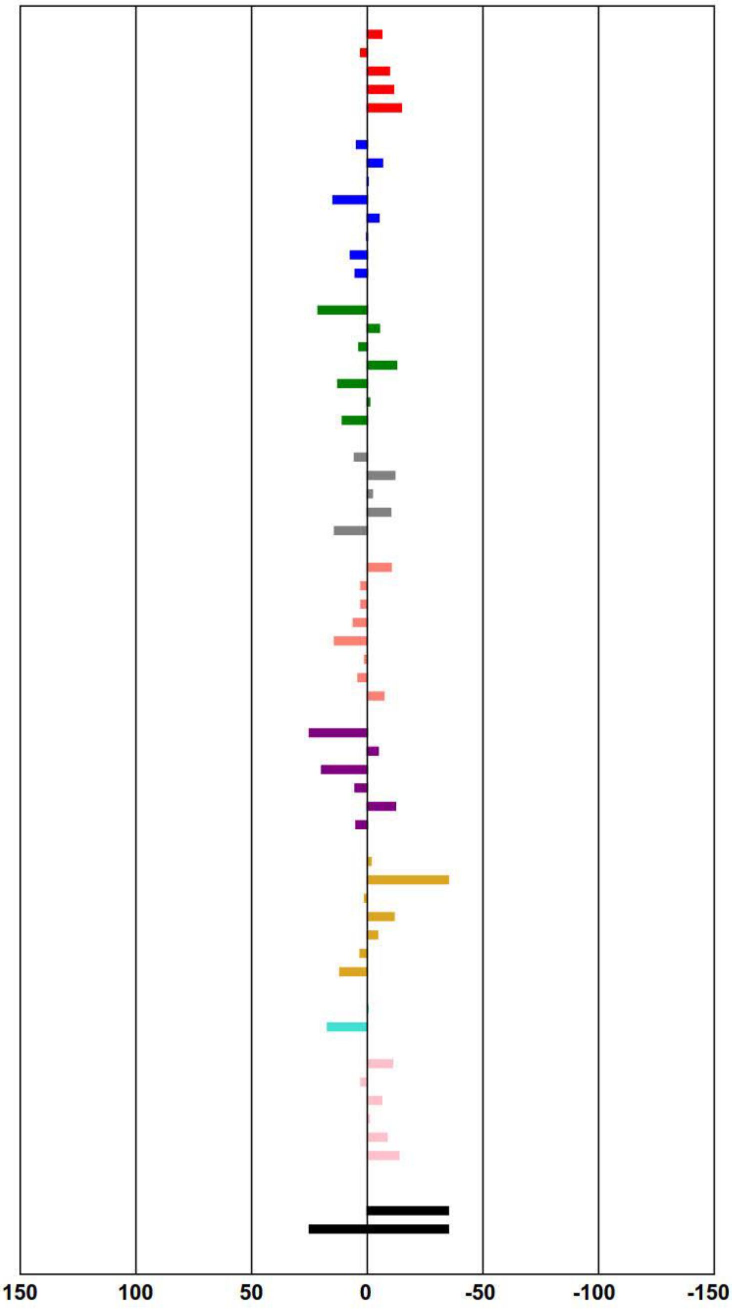

Figure S6. One dose mean graph of NCI-60 cell line screening data for 3c

| Developmental Therapeutics Program One Dose Mean Graph | NSC: D-845564 / 1 | Conc: 1.00E-5 Molar | Test Date: Jun 12, 2023 |
|---|---|---|---|
| | Experiment ID: 2306OS36 | | Report Date: Aug 23, 2023 |

| Panel/Cell Line | Growth Percent |
|---|---|
| **Leukemia** | |
| CCRF-CEM | 22.23 |
| HL-60(TB) | 14.38 |
| K-562 | 26.34 |
| MOLT-4 | 7.23 |
| RPMI-8226 | 30.27 |
| **Non-Small Cell Lung Cancer** | |
| A549/ATCC | 50.08 |
| EKVX | 36.26 |
| HOP-62 | 82.36 |
| HOP-92 | 89.04 |
| NCI-H226 | 72.29 |
| NCI-H23 | 27.10 |
| NCI-H460 | 40.75 |
| NCI-H522 | 50.13 |
| **Colon Cancer** | |
| COLO 205 | 58.24 |
| HCC-2998 | 36.20 |
| HCT-116 | 32.12 |
| HCT-15 | 23.52 |
| HT29 | 52.97 |
| KM12 | 34.61 |
| SW-620 | 56.74 |
| **CNS Cancer** | |
| SF-268 | 65.73 |
| SF-295 | 30.63 |
| SF-539 | 38.31 |
| SNB-19 | 33.39 |
| U251 | 43.75 |
| **Melanoma** | |
| LOX IMVI | 33.14 |
| M14 | 69.65 |
| MDA-MB-435 | 72.17 |
| SK-MEL-2 | 70.92 |
| SK-MEL-28 | 52.98 |
| SK-MEL-5 | 47.98 |
| UACC-257 | 92.69 |
| UACC-62 | 58.90 |
| **Ovarian Cancer** | |
| OVCAR-3 | 91.77 |
| OVCAR-4 | 78.67 |
| OVCAR-5 | 111.70 |
| OVCAR-8 | 62.51 |
| NCI/ADR-RES | 61.34 |
| SK-OV-3 | 99.01 |
| **Renal Cancer** | |
| 786-0 | 75.90 |
| A498 | 122.22 |
| ACHN | 45.25 |
| CAKI-1 | 45.10 |
| RXF 393 | 59.53 |
| SN12C | 53.55 |
| TK-10 | 93.21 |
| **Prostate Cancer** | |
| PC-3 | 42.04 |
| DU-145 | 80.01 |
| **Breast Cancer** | |
| MCF7 | 27.74 |
| MDA-MB-231/ATCC | 72.97 |
| HS 578T | 65.55 |
| BT-549 | 50.17 |
| T-47D | 49.78 |
| MDA-MB-468 | 5.55 |
| Mean | 54.57 |
| Delta | 49.02 |
| Range | 116.67 |

**Mean Growth Percent - Growth Percent**

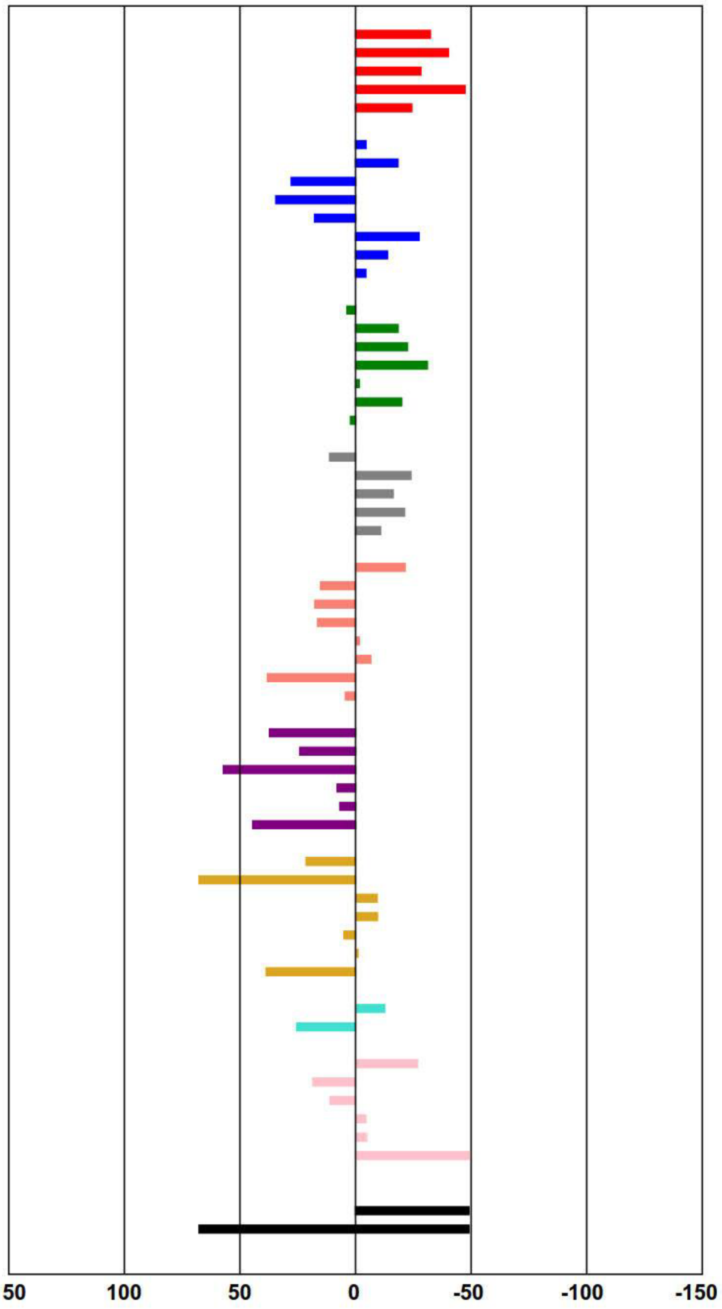

Figure S7. One dose mean graph of NCI-60 cell line screening data for 3d

| Developmental Therapeutics Program One Dose Mean Graph | NSC: D-845565 / 1 | Conc: 1.00E-5 Molar | Test Date: Jun 12, 2023 |
|---|---|---|---|
| | Experiment ID: 2306OS36 | | Report Date: Aug 23, 2023 |

| Panel/Cell Line | Growth Percent |
|---|---|
| **Leukemia** | |
| CCRF-CEM | 6.56 |
| HL-60(TB) | -21.04 |
| K-562 | 4.78 |
| MOLT-4 | -10.26 |
| RPMI-8226 | 4.56 |
| **Non-Small Cell Lung Cancer** | |
| A549/ATCC | 57.81 |
| EKVX | 26.13 |
| HOP-62 | 82.07 |
| HOP-92 | 51.55 |
| NCI-H226 | 59.14 |
| NCI-H23 | 19.00 |
| NCI-H460 | 62.20 |
| NCI-H522 | -62.24 |
| **Colon Cancer** | |
| COLO 205 | -74.16 |
| HCC-2998 | 33.77 |
| HCT-116 | -52.25 |
| HCT-15 | -34.49 |
| HT29 | 16.18 |
| KM12 | 61.72 |
| SW-620 | -62.07 |
| **CNS Cancer** | |
| SF-268 | 60.13 |
| SF-295 | 59.65 |
| SF-539 | 40.33 |
| SNB-19 | 51.45 |
| U251 | 48.34 |
| **Melanoma** | |
| LOX IMVI | -90.25 |
| M14 | 23.24 |
| MDA-MB-435 | 45.68 |
| SK-MEL-2 | 78.02 |
| SK-MEL-28 | -84.21 |
| SK-MEL-5 | 10.35 |
| UACC-257 | 8.02 |
| UACC-62 | 34.22 |
| **Ovarian Cancer** | |
| OVCAR-3 | -64.63 |
| OVCAR-4 | 38.43 |
| OVCAR-5 | 91.13 |
| OVCAR-8 | 48.08 |
| NCI/ADR-RES | 38.53 |
| SK-OV-3 | 71.56 |
| **Renal Cancer** | |
| 786-0 | -47.24 |
| A498 | 130.07 |
| ACHN | -89.15 |
| CAKI-1 | 41.05 |
| RXF 393 | -72.41 |
| SN12C | 39.87 |
| TK-10 | 36.28 |
| **Prostate Cancer** | |
| PC-3 | 24.47 |
| DU-145 | 49.63 |
| **Breast Cancer** | |
| MCF7 | 0.08 |
| MDA-MB-231/ATCC | 5.52 |
| HS 578T | 48.25 |
| BT-549 | 16.70 |
| T-47D | -64.38 |
| MDA-MB-468 | -91.75 |
| Mean | 13.04 |
| Delta | 104.79 |
| Range | 221.82 |

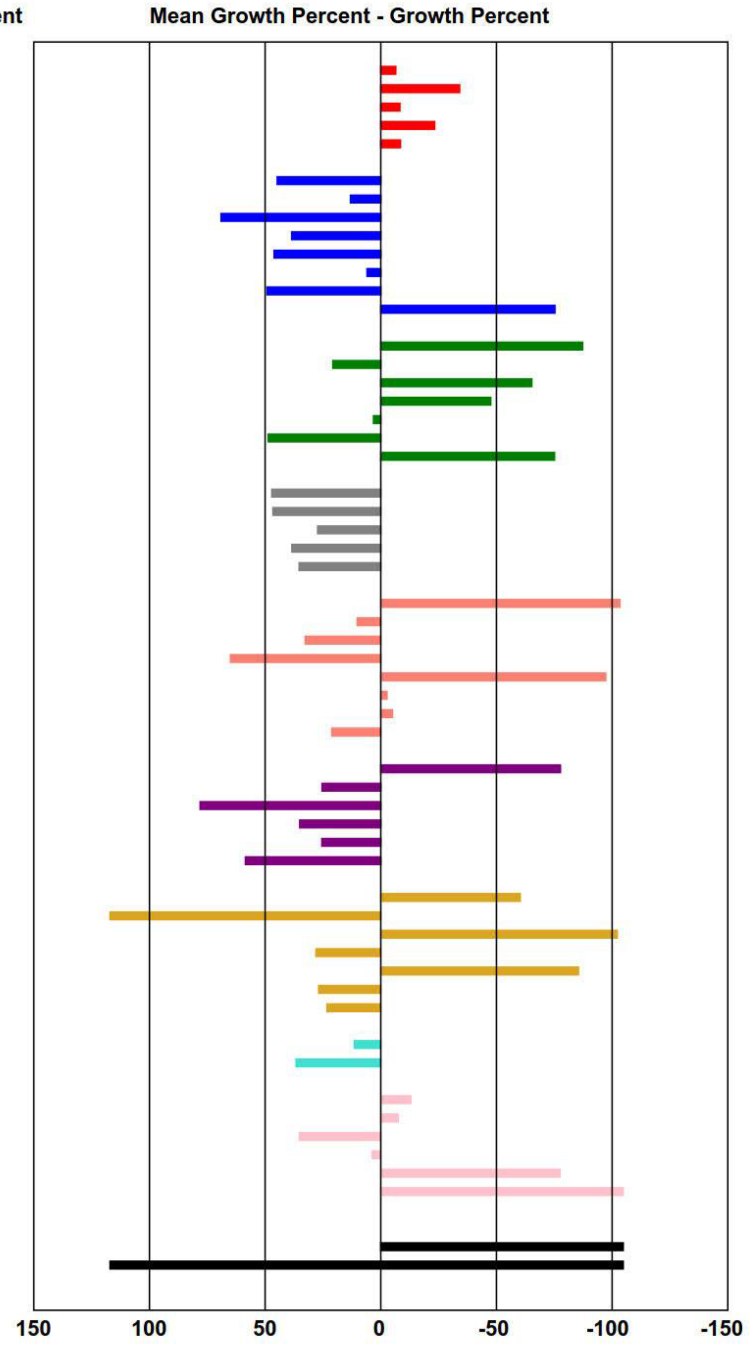

Figure S8. One dose mean graph of NCI-60 cell line screening data for 4a

**Developmental Therapeutics Program**

**One Dose Mean Graph**

| | | |
|---|---|---|
| **NSC:** D-845474 / 1 | **Conc:** 1.00E-5 Molar | **Test Date:** Jun 12, 2023 |
| **Experiment ID:** 2306OS36 | | **Report Date:** Jul 18, 2023 |

| Panel/Cell Line | Growth Percent |
|---|---|
| Leukemia | |
| CCRF-CEM | 26.59 |
| HL-60(TB) | 14.83 |
| K-562 | 29.77 |
| MOLT-4 | 8.35 |
| RPMI-8226 | 19.69 |
| SR | 17.58 |
| Non-Small Cell Lung Cancer | |
| A549/ATCC | 59.03 |
| EKVX | 64.70 |
| HOP-62 | 85.73 |
| HOP-92 | 65.21 |
| NCI-H226 | 76.89 |
| NCI-H23 | 72.44 |
| NCI-H460 | 67.05 |
| NCI-H522 | 65.01 |
| Colon Cancer | |
| COLO 205 | 70.57 |
| HCC-2998 | 80.39 |
| HCT-116 | 24.61 |
| HCT-15 | 37.39 |
| HT29 | 45.01 |
| KM12 | 87.95 |
| SW-620 | 68.85 |
| CNS Cancer | |
| SF-268 | 75.48 |
| SF-295 | 72.43 |
| SF-539 | 82.26 |
| SNB-19 | 75.97 |
| U251 | 75.25 |
| Melanoma | |
| LOX IMVI | 66.42 |
| M14 | 70.54 |
| MDA-MB-435 | 74.84 |
| SK-MEL-2 | 90.20 |
| SK-MEL-28 | 86.97 |
| SK-MEL-5 | 49.50 |
| UACC-257 | 83.09 |
| UACC-62 | 58.84 |
| Ovarian Cancer | |
| OVCAR-3 | 63.47 |
| OVCAR-4 | 62.40 |
| OVCAR-5 | 120.50 |
| OVCAR-8 | 71.70 |
| NCI/ADR-RES | 67.28 |
| SK-OV-3 | 79.90 |
| Renal Cancer | |
| 786-0 | 69.96 |
| A498 | 114.91 |
| ACHN | 71.94 |
| CAKI-1 | 55.80 |
| RXF 393 | 77.86 |
| SN12C | 74.34 |
| TK-10 | 94.28 |
| Prostate Cancer | |
| PC-3 | 15.70 |
| DU-145 | 81.12 |
| Breast Cancer | |
| MCF7 | 25.47 |
| MDA-MB-231/ATCC | 66.16 |
| HS 578T | 80.83 |
| BT-549 | 66.78 |
| T-47D | 37.72 |
| MDA-MB-468 | 10.22 |
| Mean | 62.87 |
| Delta | 54.52 |
| Range | 112.15 |

**Mean Growth Percent - Growth Percent**

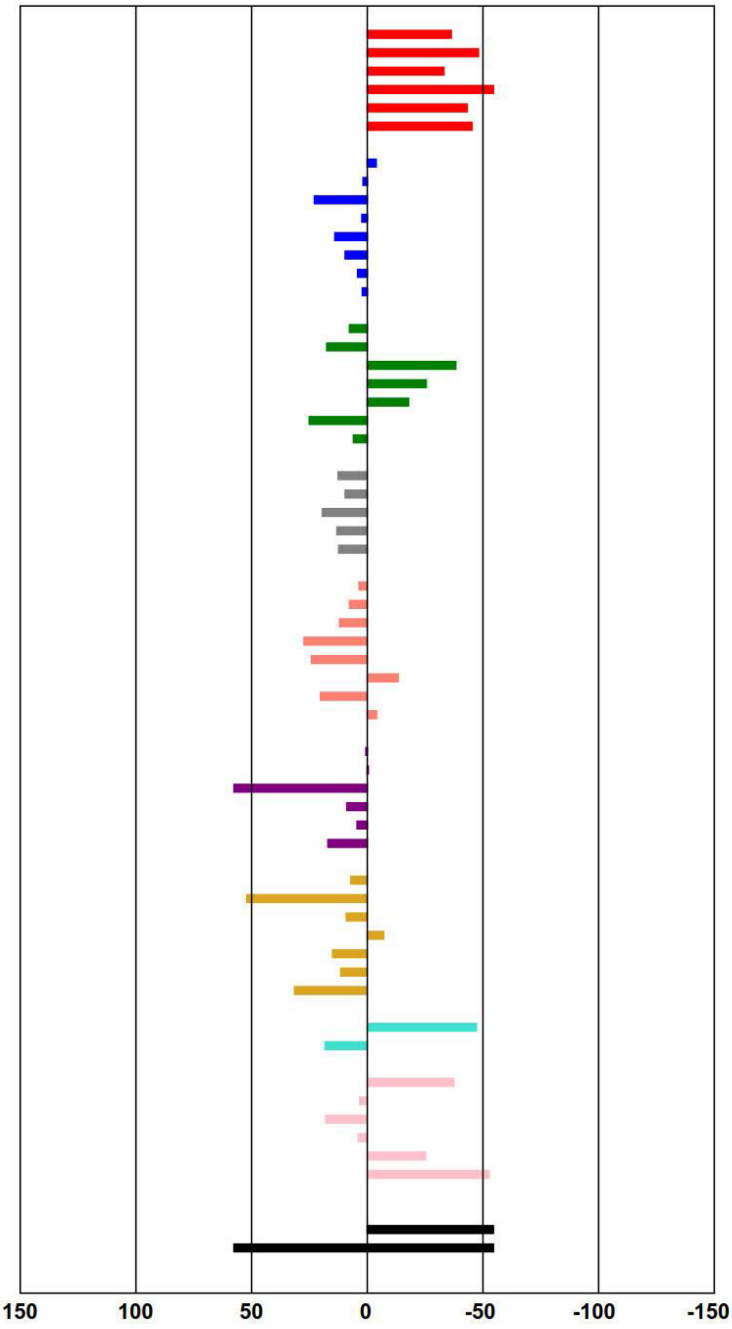

Figure S9. One dose mean graph of NCI-60 cell line screening data for 4b

| **Developmental Therapeutics Program** | **NSC:** D-835665 / 1 | **Conc:** 1.00E-5 Molar | **Test Date:** Feb 28, 2022 |
|---|---|---|---|
| **One Dose Mean Graph** | **Experiment ID:** 2202OS75 | | **Report Date:** Mar 18, 2022 |

| Panel/Cell Line | Growth Percent |
|---|---|
| Leukemia | |
| CCRF-CEM | 16.43 |
| HL-60(TB) | 2.73 |
| K-562 | 25.32 |
| MOLT-4 | 15.79 |
| RPMI-8226 | 19.80 |
| SR | 8.03 |
| Non-Small Cell Lung Cancer | |
| A549/ATCC | 65.04 |
| EKVX | 66.20 |
| HOP-62 | 92.32 |
| HOP-92 | 80.47 |
| NCI-H226 | 49.25 |
| NCI-H23 | 48.93 |
| NCI-H322M | 86.51 |
| NCI-H460 | 65.02 |
| NCI-H522 | 36.70 |
| Colon Cancer | |
| COLO 205 | 79.40 |
| HCC-2998 | 37.81 |
| HCT-116 | 36.50 |
| HCT-15 | 17.64 |
| HT29 | 41.07 |
| KM12 | 77.70 |
| SW-620 | 49.75 |
| CNS Cancer | |
| SF-268 | 64.67 |
| SF-295 | 54.43 |
| SF-539 | 77.18 |
| SNB-19 | 81.09 |
| SNB-75 | 86.18 |
| U251 | 63.84 |
| Melanoma | |
| LOX IMVI | -75.11 |
| MALME-3M | 83.01 |
| M14 | 72.19 |
| MDA-MB-435 | 70.56 |
| SK-MEL-2 | 47.39 |
| SK-MEL-28 | 62.66 |
| SK-MEL-5 | 68.62 |
| UACC-257 | 70.19 |
| UACC-62 | 38.94 |
| Ovarian Cancer | |
| IGROV1 | 18.24 |
| OVCAR-3 | 61.13 |
| OVCAR-4 | 49.39 |
| OVCAR-5 | 97.84 |
| OVCAR-8 | 58.28 |
| NCI/ADR-RES | 47.91 |
| SK-OV-3 | 96.60 |
| Renal Cancer | |
| 786-0 | 78.05 |
| A498 | 120.11 |
| ACHN | 52.88 |
| CAKI-1 | 81.33 |
| SN12C | 50.02 |
| TK-10 | 98.08 |
| UO-31 | 47.88 |
| Prostate Cancer | |
| PC-3 | 35.29 |
| DU-145 | 92.61 |
| Breast Cancer | |
| MCF7 | 13.09 |
| MDA-MB-231/ATCC | 64.78 |
| HS 578T | 52.55 |
| BT-549 | 67.79 |
| T-47D | 52.22 |
| MDA-MB-468 | 3.12 |
| Mean | 54.63 |
| Delta | 129.74 |
| Range | 195.22 |

**Mean Growth Percent - Growth Percent**

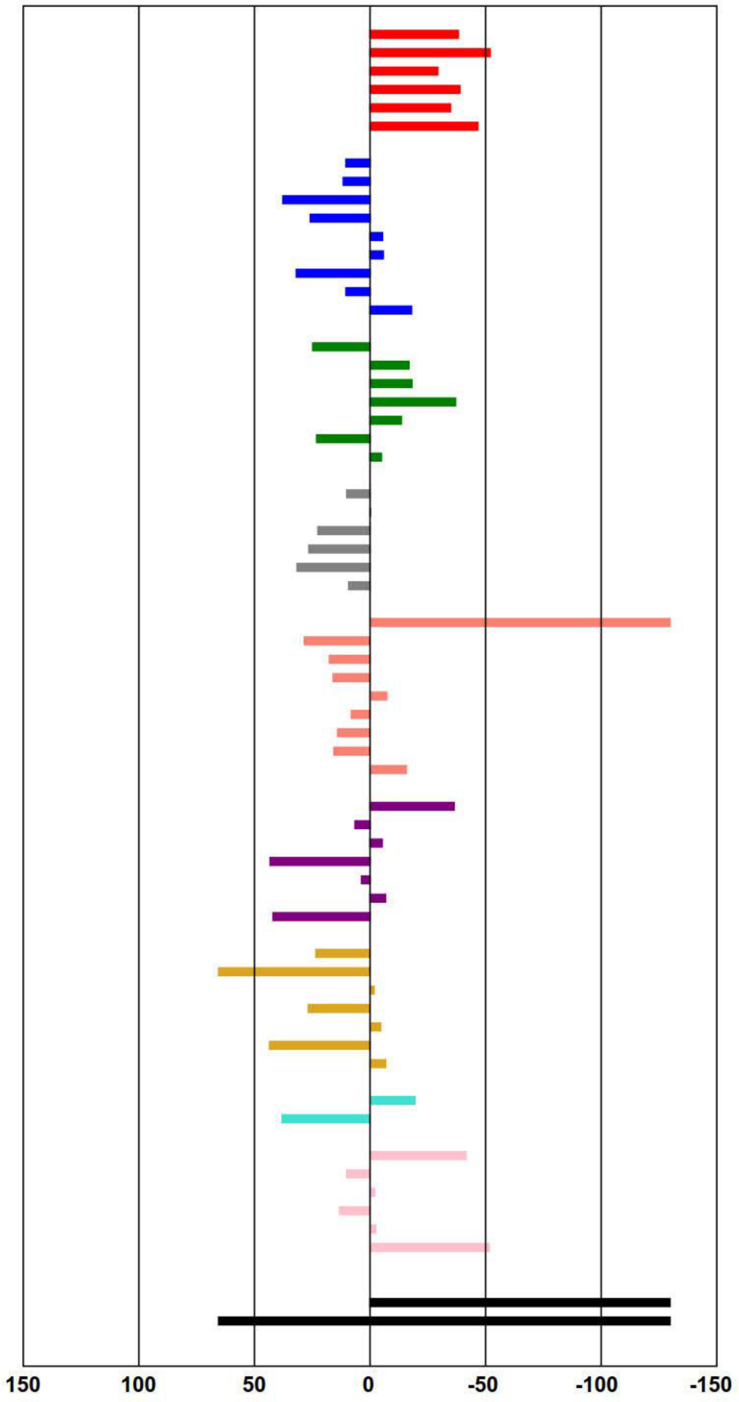

Figure S10. One dose mean graph of NCI-60 cell line screening data for 4c

| Developmental Therapeutics Program One Dose Mean Graph | NSC: D-835664 / 1 | Conc: 1.00E-5 Molar | Test Date: Feb 28, 2022 |
|---|---|---|---|
| | Experiment ID: 2202OS75 | | Report Date: Mar 18, 2022 |

| Panel/Cell Line | Growth Percent |
|---|---|
| Leukemia | |
| CCRF-CEM | 15.58 |
| HL-60(TB) | 9.46 |
| K-562 | 24.90 |
| MOLT-4 | 27.78 |
| RPMI-8226 | 15.16 |
| SR | 4.44 |
| Non-Small Cell Lung Cancer | |
| A549/ATCC | 53.95 |
| EKVX | 67.60 |
| HOP-62 | 82.81 |
| HOP-92 | 74.63 |
| NCI-H226 | 71.31 |
| NCI-H23 | 46.63 |
| NCI-H322M | 105.76 |
| NCI-H460 | 61.10 |
| NCI-H522 | 35.63 |
| Colon Cancer | |
| COLO 205 | 103.33 |
| HCC-2998 | 69.58 |
| HCT-116 | 37.38 |
| HCT-15 | 18.05 |
| HT29 | 41.98 |
| KM12 | 52.97 |
| SW-620 | 79.36 |
| CNS Cancer | |
| SF-268 | 92.72 |
| SF-295 | 48.64 |
| SF-539 | 91.75 |
| SNB-19 | 67.70 |
| SNB-75 | 81.69 |
| U251 | 44.44 |
| Melanoma | |
| LOX IMVI | 24.03 |
| MALME-3M | 100.68 |
| M14 | 73.35 |
| MDA-MB-435 | 78.49 |
| SK-MEL-2 | 82.30 |
| SK-MEL-28 | 82.49 |
| SK-MEL-5 | 83.52 |
| UACC-257 | 89.47 |
| UACC-62 | 64.41 |
| Ovarian Cancer | |
| IGROV1 | 41.80 |
| OVCAR-3 | 44.06 |
| OVCAR-4 | 62.24 |
| OVCAR-5 | 105.07 |
| OVCAR-8 | 55.86 |
| NCI/ADR-RES | 45.25 |
| SK-OV-3 | 89.42 |
| Renal Cancer | |
| 786-0 | 84.75 |
| A498 | 109.54 |
| ACHN | 57.02 |
| CAKI-1 | 88.30 |
| SN12C | 69.02 |
| TK-10 | 90.30 |
| UO-31 | 65.53 |
| Prostate Cancer | |
| PC-3 | 39.17 |
| DU-145 | 92.84 |
| Breast Cancer | |
| MCF7 | 30.70 |
| MDA-MB-231/ATCC | 88.11 |
| HS 578T | 60.30 |
| BT-549 | 76.20 |
| T-47D | 67.42 |
| MDA-MB-468 | 48.94 |
| Mean | 63.00 |
| Delta | 58.56 |
| Range | 105.10 |

Mean Growth Percent - Growth Percent

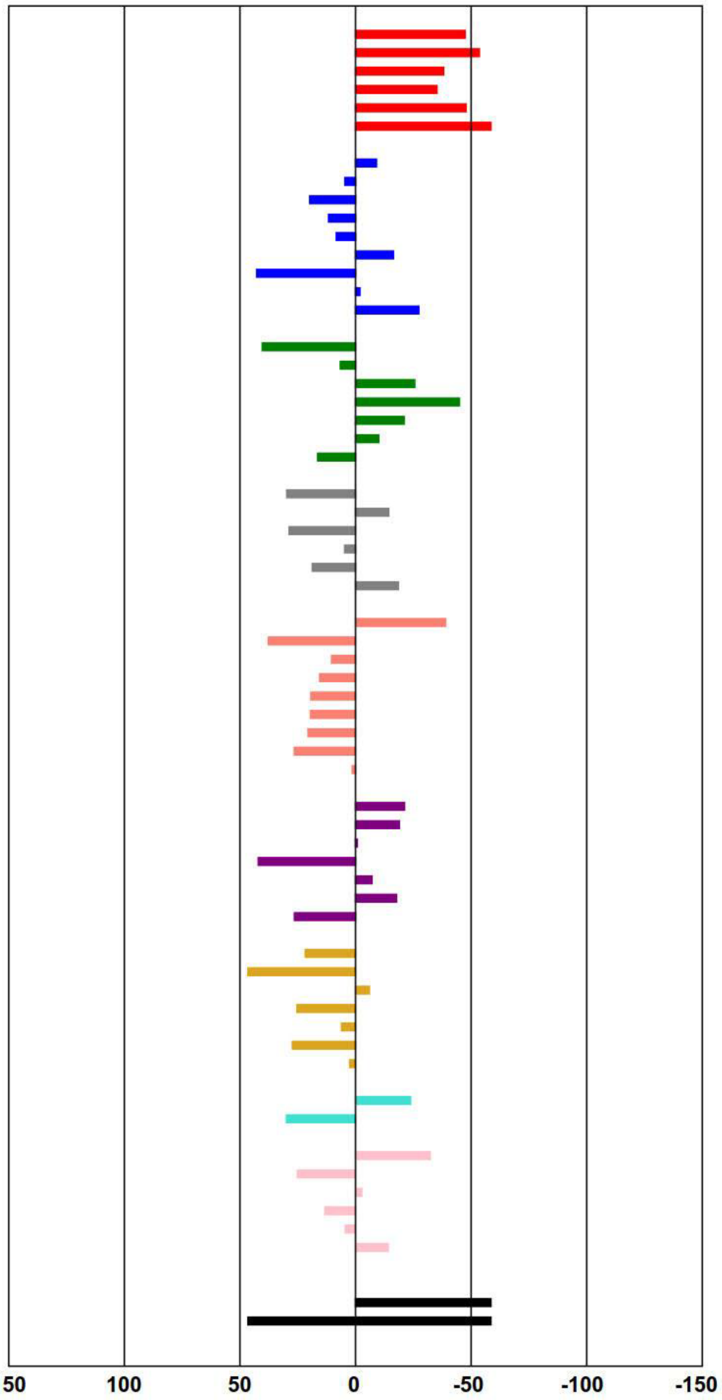

Figure S11. One dose mean graph of NCI-60 cell line screening data for 5a

| Developmental Therapeutics Program One Dose Mean Graph | NSC: D-845475 / 1 | Conc: 1.00E-5 Molar | Test Date: Jun 12, 2023 |
|---|---|---|---|
| | Experiment ID: 2306OS36 | | Report Date: Jul 18, 2023 |

| Panel/Cell Line | Growth Percent |
|---|---|
| **Leukemia** | |
| CCRF-CEM | 79.02 |
| HL-60(TB) | 78.50 |
| K-562 | 75.21 |
| MOLT-4 | 70.00 |
| RPMI-8226 | 53.19 |
| SR | 79.53 |
| **Non-Small Cell Lung Cancer** | |
| A549/ATCC | 89.88 |
| EKVX | 95.26 |
| HOP-62 | 101.20 |
| HOP-92 | 88.30 |
| NCI-H226 | 112.97 |
| NCI-H23 | 99.77 |
| NCI-H460 | 99.49 |
| NCI-H522 | 90.06 |
| **Colon Cancer** | |
| COLO 205 | 112.04 |
| HCC-2998 | 107.18 |
| HCT-116 | 90.82 |
| HCT-15 | 89.23 |
| HT29 | 96.65 |
| KM12 | 102.42 |
| SW-620 | 112.47 |
| **CNS Cancer** | |
| SF-268 | 104.21 |
| SF-295 | 95.28 |
| SF-539 | 99.72 |
| SNB-19 | 91.39 |
| U251 | 98.97 |
| **Melanoma** | |
| LOX IMVI | 102.46 |
| M14 | 102.01 |
| MDA-MB-435 | 102.03 |
| SK-MEL-2 | 100.86 |
| SK-MEL-28 | 108.91 |
| SK-MEL-5 | 80.99 |
| UACC-257 | 106.68 |
| UACC-62 | 83.69 |
| **Ovarian Cancer** | |
| OVCAR-3 | 148.98 |
| OVCAR-4 | 97.83 |
| OVCAR-5 | 131.45 |
| OVCAR-8 | 101.94 |
| NCI/ADR-RES | 91.72 |
| SK-OV-3 | 102.97 |
| **Renal Cancer** | |
| 786-0 | 94.82 |
| A498 | 131.75 |
| ACHN | 107.23 |
| CAKI-1 | 95.68 |
| RXF 393 | 104.87 |
| SN12C | 99.44 |
| TK-10 | 106.89 |
| **Prostate Cancer** | |
| PC-3 | 71.72 |
| DU-145 | 110.35 |
| **Breast Cancer** | |
| MCF7 | 83.06 |
| MDA-MB-231/ATCC | 90.41 |
| HS 578T | 99.77 |
| BT-549 | 86.75 |
| T-47D | 84.84 |
| MDA-MB-468 | 88.22 |
| Mean | 96.93 |
| Delta | 43.74 |
| Range | 95.79 |

Mean Growth Percent - Growth Percent

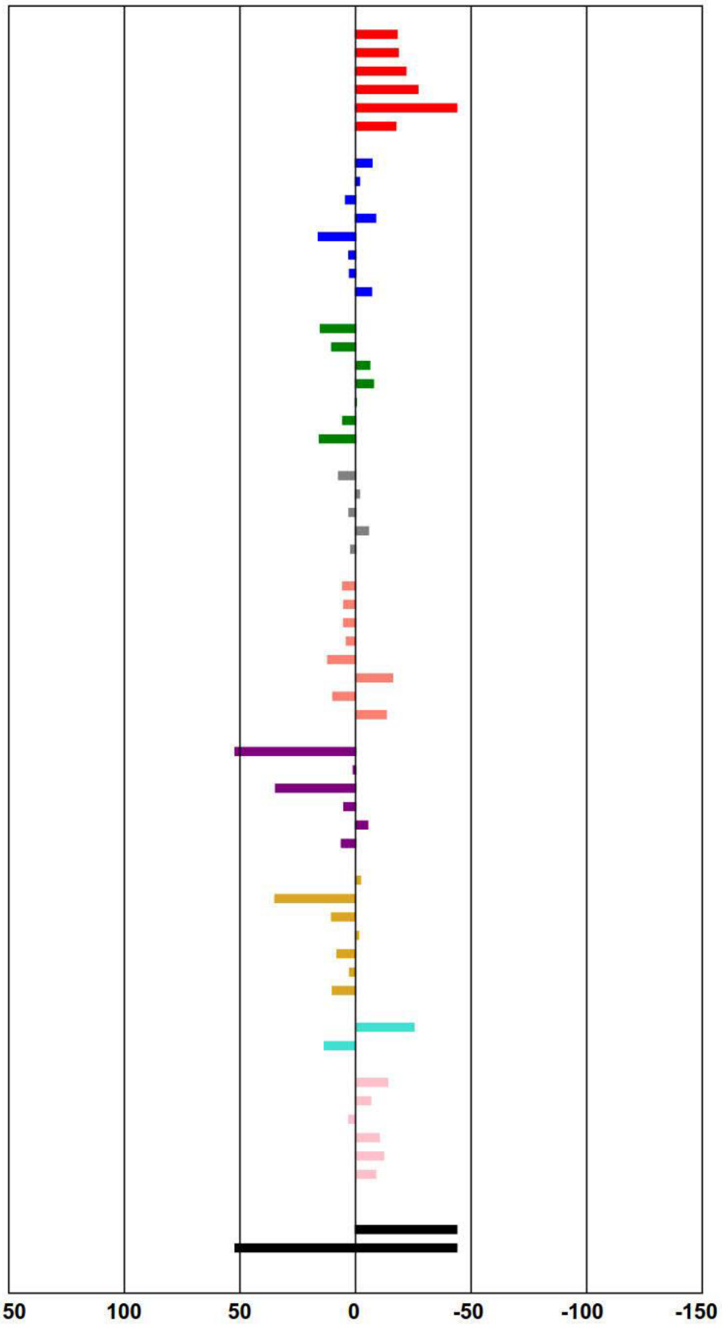

Figure S12. One dose mean graph of NCI-60 cell line screening data for 5b

| Developmental Therapeutics Program | NSC: D-845481 / 1 | Conc: 1.00E-5 Molar | Test Date: Jun 12, 2023 |
|---|---|---|---|
| One Dose Mean Graph | Experiment ID: 2306OS36 | | Report Date: Jul 18, 2023 |

| Panel/Cell Line | Growth Percent |
|---|---|
| Leukemia | |
| CCRF-CEM | 92.61 |
| HL-60(TB) | 95.90 |
| K-562 | 99.21 |
| MOLT-4 | 96.37 |
| RPMI-8226 | 76.17 |
| SR | 107.36 |
| Non-Small Cell Lung Cancer | |
| A549/ATCC | 96.05 |
| EKVX | 96.61 |
| HOP-62 | 103.81 |
| HOP-92 | 94.40 |
| NCI-H226 | 113.25 |
| NCI-H23 | 97.43 |
| NCI-H460 | 102.79 |
| NCI-H522 | 97.22 |
| Colon Cancer | |
| COLO 205 | 115.38 |
| HCC-2998 | 98.04 |
| HCT-116 | 98.35 |
| HCT-15 | 81.79 |
| HT29 | 108.52 |
| KM12 | 97.94 |
| SW-620 | 108.01 |
| CNS Cancer | |
| SF-268 | 109.87 |
| SF-295 | 79.51 |
| SF-539 | 96.01 |
| SNB-19 | 95.71 |
| U251 | 92.14 |
| Melanoma | |
| LOX IMVI | 99.48 |
| M14 | 95.55 |
| MDA-MB-435 | 102.43 |
| SK-MEL-2 | 103.40 |
| SK-MEL-28 | 97.38 |
| SK-MEL-5 | 95.11 |
| UACC-257 | 94.93 |
| UACC-62 | 94.55 |
| Ovarian Cancer | |
| OVCAR-3 | 135.02 |
| OVCAR-4 | 64.57 |
| OVCAR-5 | 104.44 |
| OVCAR-8 | 98.66 |
| NCI/ADR-RES | 89.18 |
| SK-OV-3 | 102.18 |
| Renal Cancer | |
| 786-0 | 85.38 |
| A498 | 110.59 |
| ACHN | 98.12 |
| CAKI-1 | 89.45 |
| RXF 393 | 113.17 |
| SN12C | 101.24 |
| TK-10 | 97.24 |
| Prostate Cancer | |
| PC-3 | 79.45 |
| DU-145 | 119.70 |
| Breast Cancer | |
| MCF7 | 94.80 |
| MDA-MB-231/ATCC | 91.93 |
| HS 578T | 95.41 |
| BT-549 | 92.41 |
| T-47D | 92.06 |
| MDA-MB-468 | 91.85 |
| Mean | 97.82 |
| Delta | 33.25 |
| Range | 70.45 |

Mean Growth Percent - Growth Percent

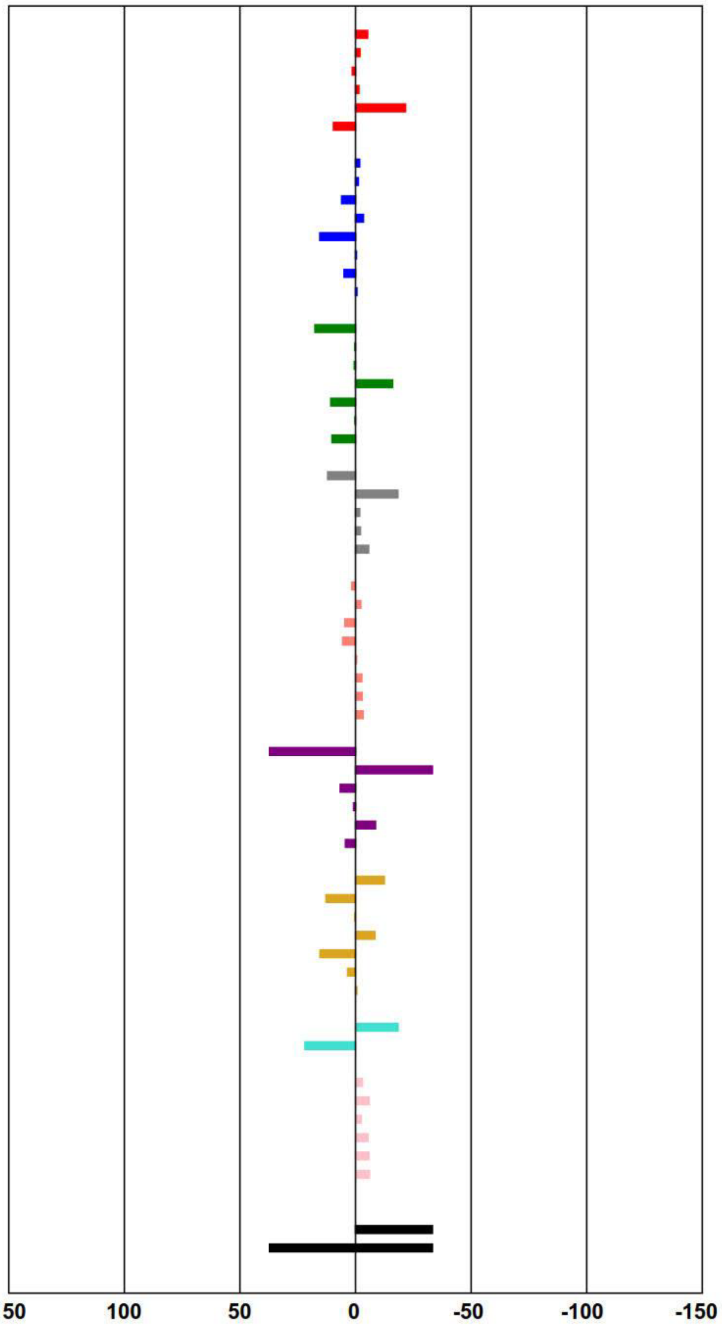

Figure S13. One dose mean graph of NCI-60 cell line screening data for 5c

| Developmental Therapeutics Program One Dose Mean Graph | NSC: D-835666 / 1 | Conc: 1.00E-5 Molar | Test Date: Feb 28, 2022 |
|---|---|---|---|
| | Experiment ID: 2202OS75 | | Report Date: Mar 18, 2022 |

| Panel/Cell Line | Growth Percent |
|---|---|
| **Leukemia** | |
| CCRF-CEM | 95.79 |
| HL-60(TB) | 94.48 |
| K-562 | 94.61 |
| MOLT-4 | 80.93 |
| RPMI-8226 | 84.30 |
| SR | 73.10 |
| **Non-Small Cell Lung Cancer** | |
| A549/ATCC | 92.19 |
| EKVX | 93.04 |
| HOP-62 | 98.97 |
| HOP-92 | 101.65 |
| NCI-H226 | 96.33 |
| NCI-H23 | 93.59 |
| NCI-H322M | 101.98 |
| NCI-H460 | 111.99 |
| NCI-H522 | 78.43 |
| **Colon Cancer** | |
| COLO 205 | 122.01 |
| HCC-2998 | 94.79 |
| HCT-116 | 90.50 |
| HCT-15 | 88.79 |
| HT29 | 107.18 |
| KM12 | 100.78 |
| SW-620 | 108.78 |
| **CNS Cancer** | |
| SF-268 | 95.32 |
| SF-295 | 92.85 |
| SF-539 | 100.45 |
| SNB-19 | 94.92 |
| SNB-75 | 95.26 |
| U251 | 100.38 |
| **Melanoma** | |
| LOX IMVI | 92.40 |
| MALME-3M | 107.56 |
| M14 | 100.33 |
| MDA-MB-435 | 102.69 |
| SK-MEL-2 | 98.31 |
| SK-MEL-28 | 101.60 |
| SK-MEL-5 | 99.55 |
| UACC-257 | 105.35 |
| UACC-62 | 78.72 |
| **Ovarian Cancer** | |
| IGROV1 | 100.54 |
| OVCAR-3 | 103.15 |
| OVCAR-4 | 91.91 |
| OVCAR-5 | 101.74 |
| OVCAR-8 | 101.59 |
| NCI/ADR-RES | 89.48 |
| SK-OV-3 | 99.63 |
| **Renal Cancer** | |
| 786-0 | 87.87 |
| A498 | 121.64 |
| ACHN | 101.02 |
| CAKI-1 | 98.65 |
| SN12C | 107.42 |
| TK-10 | 110.43 |
| UO-31 | 77.93 |
| **Prostate Cancer** | |
| PC-3 | 80.77 |
| DU-145 | 116.93 |
| **Breast Cancer** | |
| MCF7 | 91.59 |
| MDA-MB-231/ATCC | 91.43 |
| HS 578T | 84.32 |
| BT-549 | 93.40 |
| T-47D | 93.31 |
| MDA-MB-468 | 100.76 |
| Mean | 96.87 |
| Delta | 23.77 |
| Range | 48.91 |

Mean Growth Percent - Growth Percent

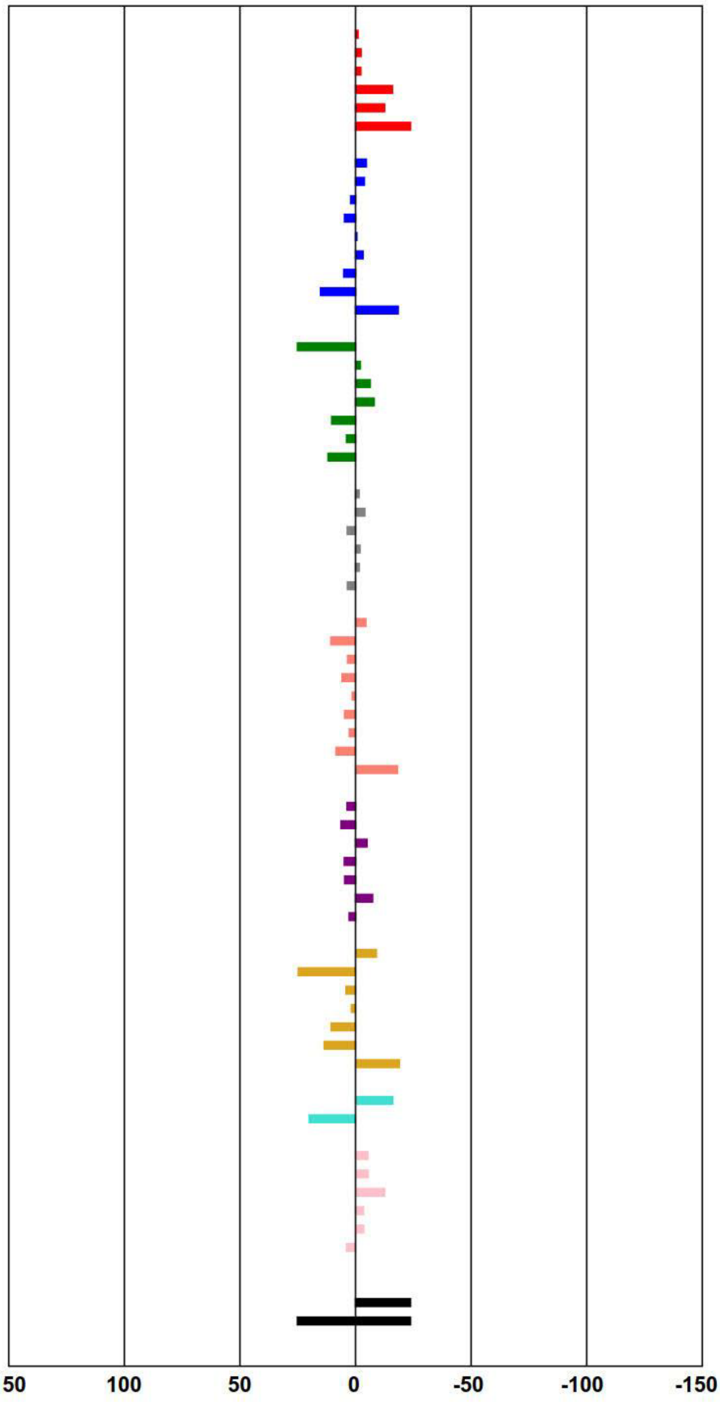

Figure S14. One dose mean graph of NCI-60 cell line screening data for 6a

| **Developmental Therapeutics Program** | **NSC:** D-845473 / 1 | **Conc:** 1.00E-5 Molar | **Test Date:** Jun 12, 2023 |
|---|---|---|---|
| **One Dose Mean Graph** | **Experiment ID:** 2306OS36 | | **Report Date:** Jul 18, 2023 |

| Panel/Cell Line | Growth Percent |
|---|---|
| Leukemia | |
| CCRF-CEM | 78.53 |
| HL-60(TB) | 102.98 |
| K-562 | 73.04 |
| MOLT-4 | 69.01 |
| RPMI-8226 | 73.93 |
| SR | 89.44 |
| Non-Small Cell Lung Cancer | |
| A549/ATCC | 97.50 |
| EKVX | 93.65 |
| HOP-62 | 101.56 |
| HOP-92 | 83.35 |
| NCI-H226 | 101.47 |
| NCI-H23 | 99.35 |
| NCI-H460 | 104.44 |
| NCI-H522 | 86.43 |
| Colon Cancer | |
| COLO 205 | 111.65 |
| HCC-2998 | 102.10 |
| HCT-116 | 89.06 |
| HCT-15 | 94.73 |
| HT29 | 101.51 |
| KM12 | 103.05 |
| SW-620 | 107.31 |
| CNS Cancer | |
| SF-268 | 114.98 |
| SF-295 | 93.75 |
| SF-539 | 97.72 |
| SNB-19 | 93.79 |
| U251 | 99.31 |
| Melanoma | |
| LOX IMVI | 90.73 |
| M14 | 100.32 |
| MDA-MB-435 | 96.07 |
| SK-MEL-2 | 100.98 |
| SK-MEL-28 | 103.40 |
| SK-MEL-5 | 87.15 |
| UACC-257 | 104.23 |
| UACC-62 | 86.12 |
| Ovarian Cancer | |
| OVCAR-3 | 156.30 |
| OVCAR-4 | 91.89 |
| OVCAR-5 | 114.58 |
| OVCAR-8 | 102.03 |
| NCI/ADR-RES | 92.93 |
| SK-OV-3 | 103.85 |
| Renal Cancer | |
| 786-0 | 92.67 |
| A498 | 114.34 |
| ACHN | 98.61 |
| CAKI-1 | 78.11 |
| RXF 393 | 105.29 |
| SN12C | 105.69 |
| TK-10 | 101.10 |
| Prostate Cancer | |
| PC-3 | 79.37 |
| DU-145 | 113.34 |
| Breast Cancer | |
| MCF7 | 81.47 |
| MDA-MB-231/ATCC | 95.14 |
| HS 578T | 104.91 |
| BT-549 | 112.24 |
| T-47D | 84.68 |
| MDA-MB-468 | 68.74 |
| Mean | 96.91 |
| Delta | 28.17 |
| Range | 87.56 |

**Mean Growth Percent - Growth Percent**

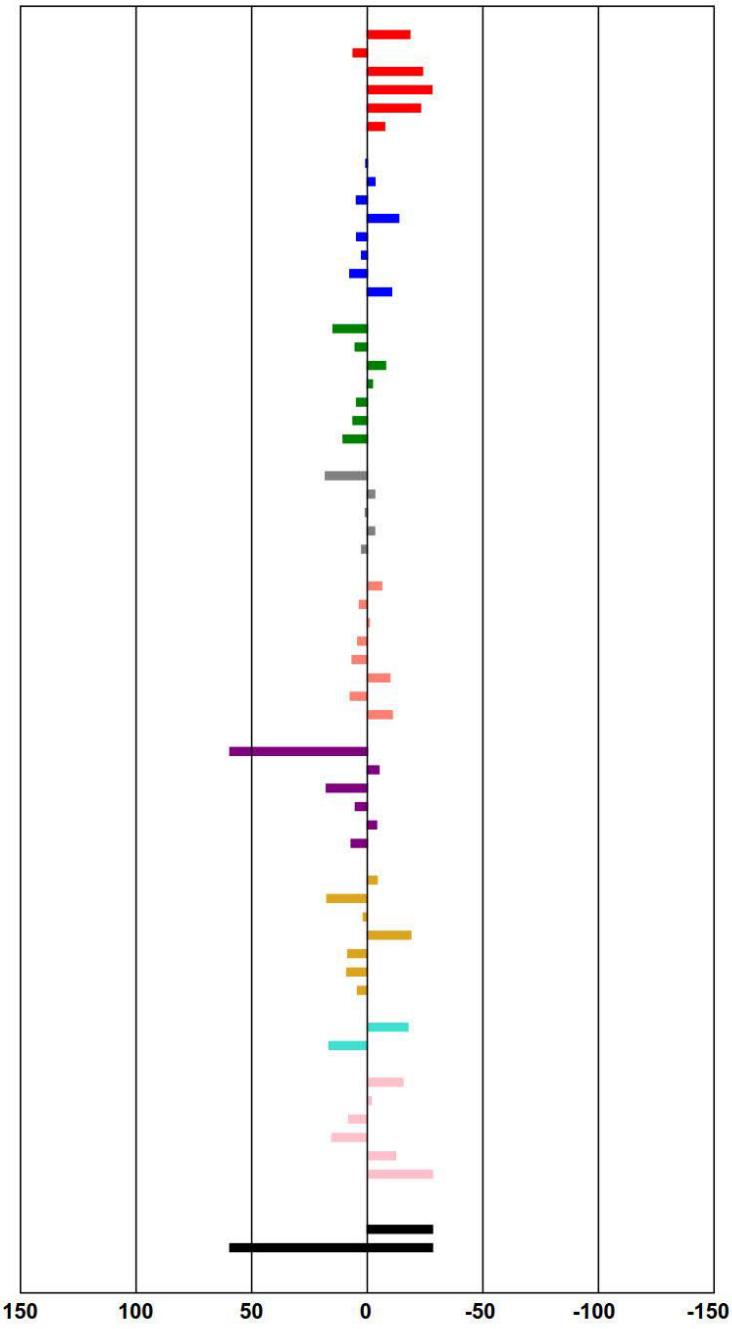

Figure S15. One dose mean graph of NCI-60 cell line screening data for 6c

| Developmental Therapeutics Program | NSC: D-845476 / 1 | Conc: 1.00E-5 Molar | Test Date: Jun 12, 2023 |
|---|---|---|---|
| One Dose Mean Graph | Experiment ID: 2306OS36 | | Report Date: Jul 18, 2023 |

| Panel/Cell Line | Growth Percent |
|---|---|
| Leukemia | |
| CCRF-CEM | 76.24 |
| HL-60(TB) | 102.82 |
| K-562 | 84.87 |
| MOLT-4 | 66.66 |
| RPMI-8226 | 69.04 |
| SR | 80.70 |
| Non-Small Cell Lung Cancer | |
| A549/ATCC | 97.30 |
| EKVX | 101.00 |
| HOP-62 | 102.01 |
| HOP-92 | 92.03 |
| NCI-H226 | 100.05 |
| NCI-H23 | 98.99 |
| NCI-H460 | 99.75 |
| NCI-H522 | 83.49 |
| Colon Cancer | |
| COLO 205 | 111.10 |
| HCC-2998 | 102.00 |
| HCT-116 | 87.65 |
| HCT-15 | 86.54 |
| HT29 | 97.44 |
| KM12 | 103.68 |
| SW-620 | 106.95 |
| CNS Cancer | |
| SF-268 | 98.44 |
| SF-295 | 96.41 |
| SF-539 | 97.95 |
| SNB-19 | 96.72 |
| U251 | 98.48 |
| Melanoma | |
| LOX IMVI | 94.18 |
| M14 | 100.35 |
| MDA-MB-435 | 105.65 |
| SK-MEL-2 | 95.78 |
| SK-MEL-28 | 101.33 |
| SK-MEL-5 | 86.12 |
| UACC-257 | 101.61 |
| UACC-62 | 86.02 |
| Ovarian Cancer | |
| OVCAR-3 | 135.59 |
| OVCAR-4 | 95.25 |
| OVCAR-5 | 109.75 |
| OVCAR-8 | 98.71 |
| NCI/ADR-RES | 86.65 |
| SK-OV-3 | 99.56 |
| Renal Cancer | |
| 786-0 | 91.70 |
| A498 | 112.99 |
| ACHN | 97.83 |
| CAKI-1 | 73.73 |
| RXF 393 | 100.75 |
| SN12C | 108.72 |
| TK-10 | 102.86 |
| Prostate Cancer | |
| PC-3 | 66.66 |
| DU-145 | 112.80 |
| Breast Cancer | |
| MCF7 | 84.28 |
| MDA-MB-231/ATCC | 89.34 |
| HS 578T | 91.80 |
| BT-549 | 92.29 |
| T-47D | 85.04 |
| MDA-MB-468 | 65.96 |
| Mean | 94.76 |
| Delta | 28.80 |
| Range | 69.63 |

Mean Growth Percent - Growth Percent

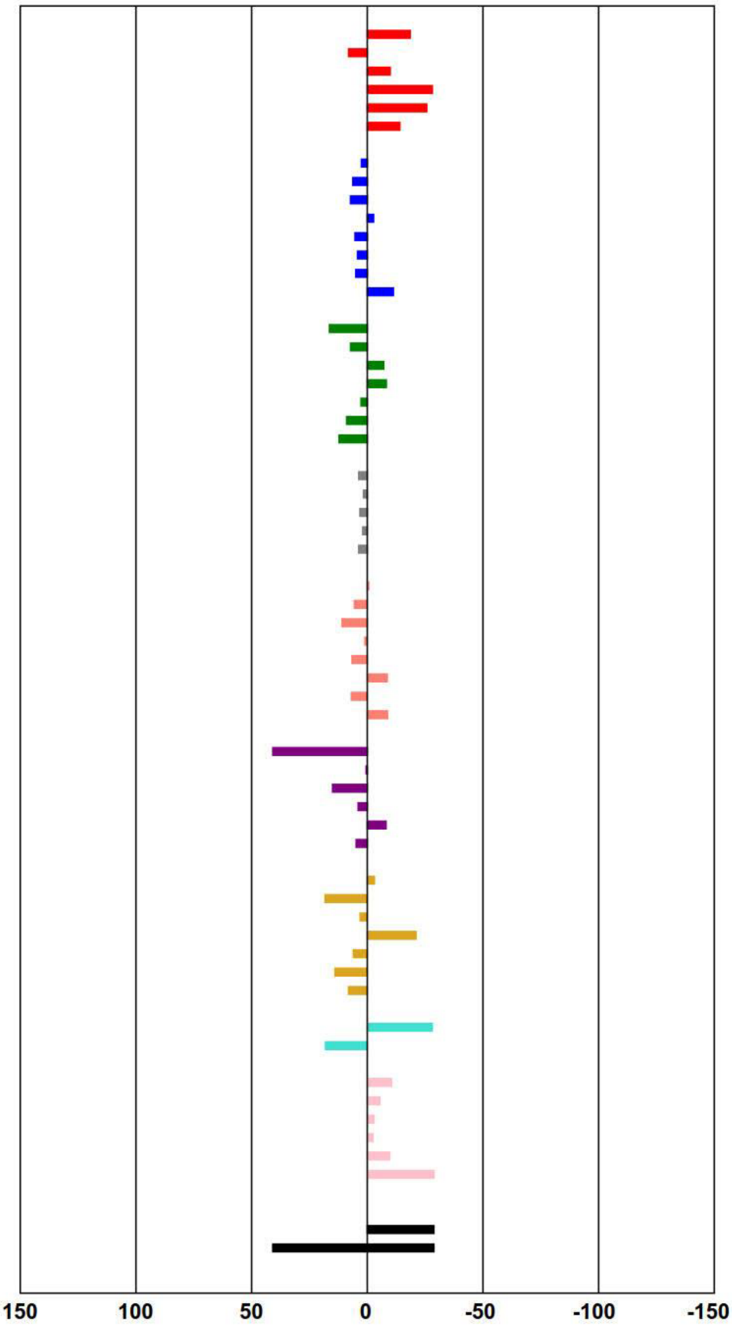

Figure S16. One dose mean graph of NCI-60 cell line screening data for 7b

| Developmental Therapeutics Program One Dose Mean Graph | NSC: D-845480 / 1 | Conc: 1.00E-5 Molar | Test Date: Jun 12, 2023 |
|---|---|---|---|
| | Experiment ID: 2306OS36 | | Report Date: Jul 18, 2023 |

| Panel/Cell Line | Growth Percent |
|---|---|
| Leukemia | |
| CCRF-CEM | 90.53 |
| HL-60(TB) | 93.38 |
| K-562 | 99.75 |
| MOLT-4 | 95.60 |
| RPMI-8226 | 74.63 |
| SR | 106.39 |
| Non-Small Cell Lung Cancer | |
| A549/ATCC | 97.31 |
| EKVX | 96.63 |
| HOP-62 | 96.36 |
| HOP-92 | 87.22 |
| NCI-H226 | 107.34 |
| NCI-H23 | 98.97 |
| NCI-H460 | 102.65 |
| NCI-H522 | 101.24 |
| Colon Cancer | |
| COLO 205 | 115.16 |
| HCC-2998 | 100.04 |
| HCT-116 | 93.99 |
| HCT-15 | 85.07 |
| HT29 | 107.83 |
| KM12 | 97.80 |
| SW-620 | 102.05 |
| CNS Cancer | |
| SF-268 | 103.55 |
| SF-295 | 86.82 |
| SF-539 | 95.05 |
| SNB-19 | 96.28 |
| U251 | 97.74 |
| Melanoma | |
| LOX IMVI | 100.61 |
| M14 | 96.88 |
| MDA-MB-435 | 103.14 |
| SK-MEL-2 | 105.64 |
| SK-MEL-28 | 100.11 |
| SK-MEL-5 | 89.87 |
| UACC-257 | 104.16 |
| UACC-62 | 90.44 |
| Ovarian Cancer | |
| OVCAR-3 | 122.20 |
| OVCAR-4 | 98.59 |
| OVCAR-5 | 100.81 |
| OVCAR-8 | 100.69 |
| NCI/ADR-RES | 86.40 |
| SK-OV-3 | 103.11 |
| Renal Cancer | |
| 786-0 | 95.37 |
| A498 | 106.95 |
| ACHN | 101.12 |
| CAKI-1 | 89.05 |
| RXF 393 | 107.78 |
| SN12C | 94.35 |
| TK-10 | 97.89 |
| Prostate Cancer | |
| PC-3 | 74.39 |
| DU-145 | 110.93 |
| Breast Cancer | |
| MCF7 | 91.01 |
| MDA-MB-231/ATCC | 97.29 |
| HS 578T | 90.25 |
| BT-549 | 96.58 |
| T-47D | 91.83 |
| MDA-MB-468 | 91.67 |
| Mean | 97.61 |
| Delta | 23.22 |
| Range | 47.81 |

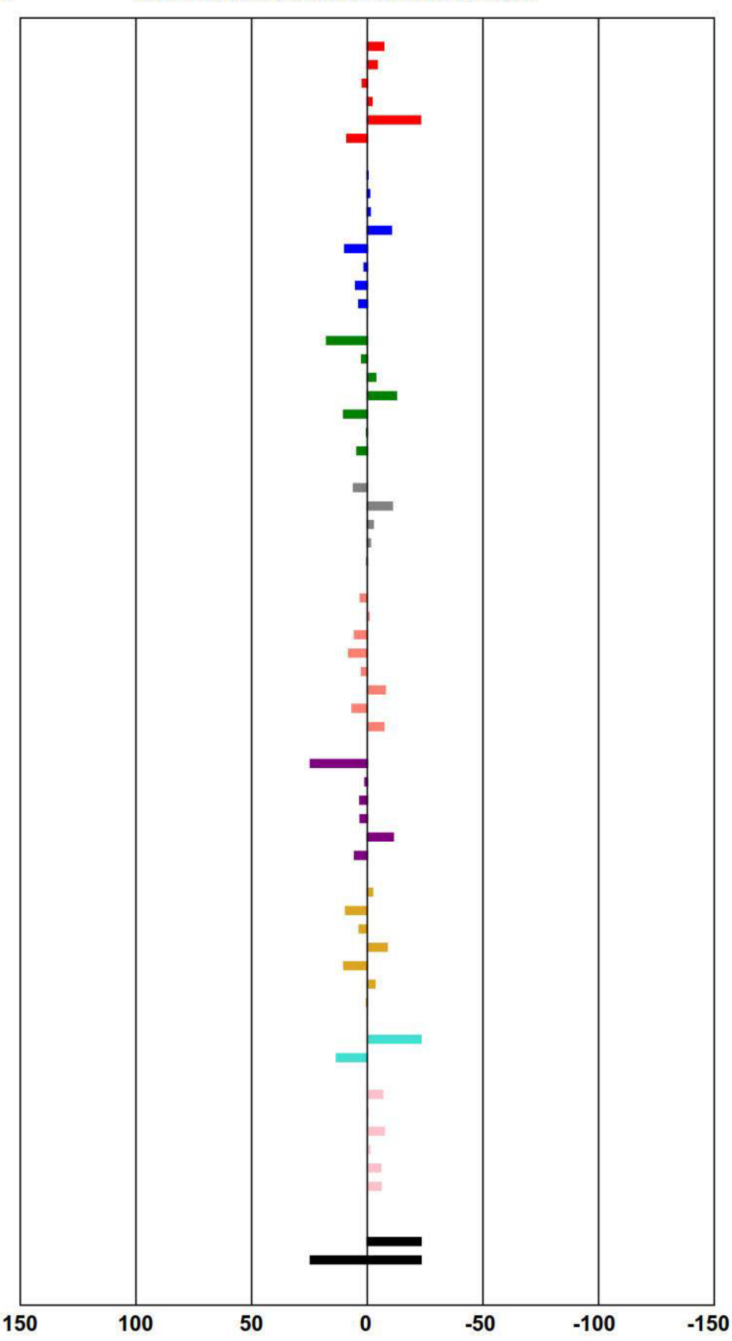

Figure S17. One dose mean graph of NCI-60 cell line screening data for 7c

| Developmental Therapeutics Program One Dose Mean Graph | NSC: D-845477 / 1 | Conc: 1.00E-5 Molar | Test Date: Jun 12, 2023 |
|---|---|---|---|
| | Experiment ID: 2306OS36 | | Report Date: Jul 18, 2023 |

| Panel/Cell Line | Growth Percent |
|---|---|
| **Leukemia** | |
| CCRF-CEM | 24.83 |
| HL-60(TB) | 34.95 |
| K-562 | 43.12 |
| MOLT-4 | 29.95 |
| RPMI-8226 | 41.78 |
| SR | 37.50 |
| **Non-Small Cell Lung Cancer** | |
| A549/ATCC | 75.68 |
| EKVX | 67.60 |
| HOP-62 | 96.90 |
| HOP-92 | 68.67 |
| NCI-H226 | 89.79 |
| NCI-H23 | 78.51 |
| NCI-H460 | 76.94 |
| NCI-H522 | 54.31 |
| **Colon Cancer** | |
| COLO 205 | 95.47 |
| HCC-2998 | 81.51 |
| HCT-116 | 33.48 |
| HCT-15 | 53.47 |
| HT29 | 83.88 |
| KM12 | 76.94 |
| SW-620 | 86.94 |
| **CNS Cancer** | |
| SF-268 | 89.29 |
| SF-295 | 61.98 |
| SF-539 | 85.16 |
| SNB-19 | 82.60 |
| U251 | 73.25 |
| **Melanoma** | |
| LOX IMVI | 73.75 |
| M14 | 86.42 |
| MDA-MB-435 | 100.30 |
| SK-MEL-2 | 85.24 |
| SK-MEL-28 | 85.57 |
| SK-MEL-5 | 58.59 |
| UACC-257 | 90.80 |
| UACC-62 | 66.62 |
| **Ovarian Cancer** | |
| OVCAR-3 | 31.67 |
| OVCAR-4 | 75.71 |
| OVCAR-5 | 139.14 |
| OVCAR-8 | 83.31 |
| NCI/ADR-RES | 72.25 |
| SK-OV-3 | 90.50 |
| **Renal Cancer** | |
| 786-0 | 79.69 |
| A498 | 121.97 |
| ACHN | 43.50 |
| CAKI-1 | 61.56 |
| RXF 393 | 69.98 |
| SN12C | 90.09 |
| TK-10 | 102.49 |
| **Prostate Cancer** | |
| PC-3 | 53.12 |
| DU-145 | 90.79 |
| **Breast Cancer** | |
| MCF7 | 49.91 |
| MDA-MB-231/ATCC | 85.02 |
| HS 578T | 69.40 |
| BT-549 | 77.59 |
| T-47D | 42.92 |
| MDA-MB-468 | -0.15 |
| Mean | 70.95 |
| Delta | 71.10 |
| Range | 139.29 |

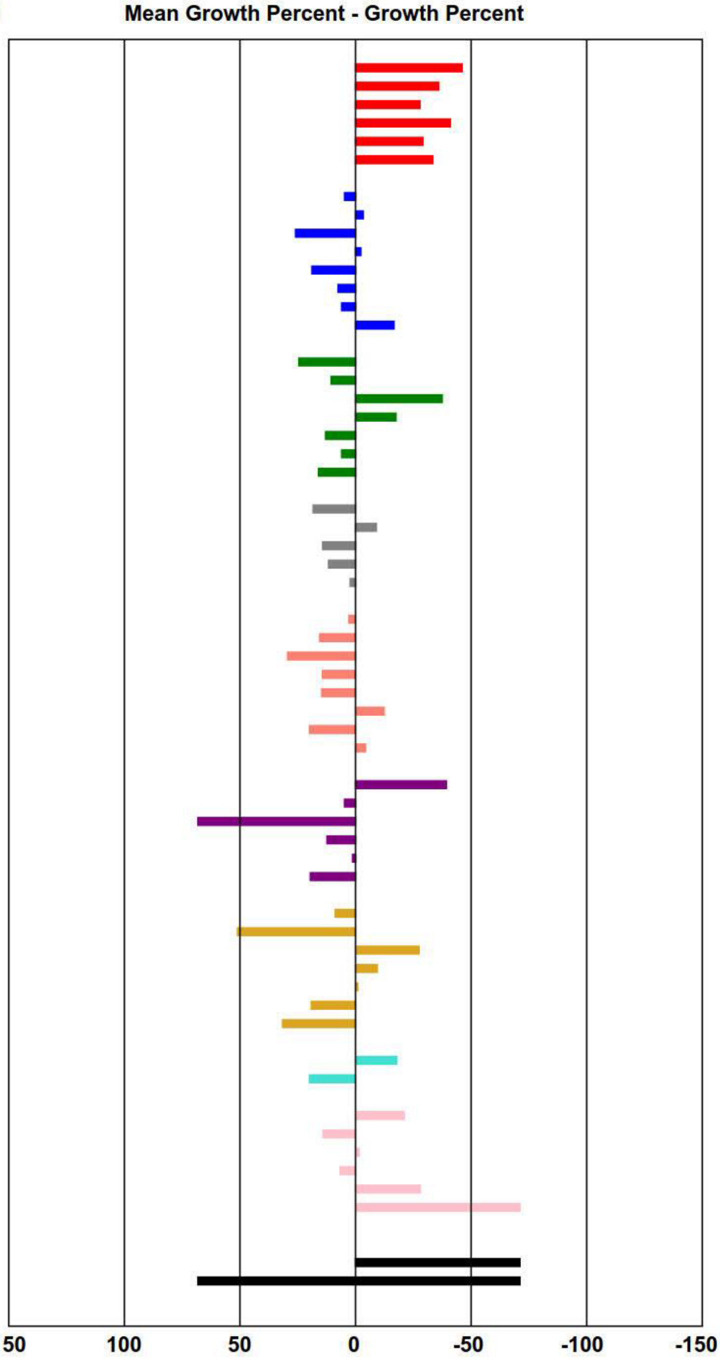

Figure S18. One dose mean graph of NCI-60 cell line screening data for 8b

| Developmental Therapeutics Program | NSC: D-845479 / 1 | Conc: 1.00E-5 Molar | Test Date: Jun 12, 2023 |
|---|---|---|---|
| One Dose Mean Graph | Experiment ID: 2306OS36 | | Report Date: Jul 18, 2023 |

| Panel/Cell Line | Growth Percent |
|---|---|
| Leukemia | |
| CCRF-CEM | 52.77 |
| HL-60(TB) | 72.41 |
| K-562 | 70.52 |
| MOLT-4 | 64.26 |
| RPMI-8226 | 28.01 |
| SR | 45.11 |
| Non-Small Cell Lung Cancer | |
| A549/ATCC | 83.94 |
| EKVX | 88.06 |
| HOP-62 | 98.38 |
| HOP-92 | 80.57 |
| NCI-H226 | 111.91 |
| NCI-H23 | 88.12 |
| NCI-H460 | 91.10 |
| NCI-H522 | 86.71 |
| Colon Cancer | |
| COLO 205 | 99.69 |
| HCC-2998 | 89.91 |
| HCT-116 | 82.04 |
| HCT-15 | 58.25 |
| HT29 | 89.10 |
| KM12 | 86.31 |
| SW-620 | 90.10 |
| CNS Cancer | |
| SF-268 | 102.72 |
| SF-295 | 70.07 |
| SF-539 | 95.08 |
| SNB-19 | 79.79 |
| U251 | 77.71 |
| Melanoma | |
| LOX IMVI | 93.26 |
| M14 | 93.98 |
| MDA-MB-435 | 97.73 |
| SK-MEL-2 | 97.70 |
| SK-MEL-28 | 97.83 |
| SK-MEL-5 | 88.17 |
| UACC-257 | 95.95 |
| UACC-62 | 85.84 |
| Ovarian Cancer | |
| OVCAR-3 | 126.65 |
| OVCAR-4 | 92.77 |
| OVCAR-5 | 101.02 |
| OVCAR-8 | 86.84 |
| NCI/ADR-RES | 72.98 |
| SK-OV-3 | 96.21 |
| Renal Cancer | |
| 786-0 | 97.05 |
| A498 | 109.62 |
| ACHN | 91.49 |
| CAKI-1 | 82.21 |
| RXF 393 | 90.45 |
| SN12C | 90.57 |
| TK-10 | 94.10 |
| Prostate Cancer | |
| PC-3 | 63.04 |
| DU-145 | 98.80 |
| Breast Cancer | |
| MCF7 | 72.37 |
| MDA-MB-231/ATCC | 96.10 |
| HS 578T | 77.39 |
| BT-549 | 103.25 |
| T-47D | 76.74 |
| MDA-MB-468 | 64.05 |
| Mean | 85.76 |
| Delta | 57.75 |
| Range | 98.64 |

Mean Growth Percent - Growth Percent

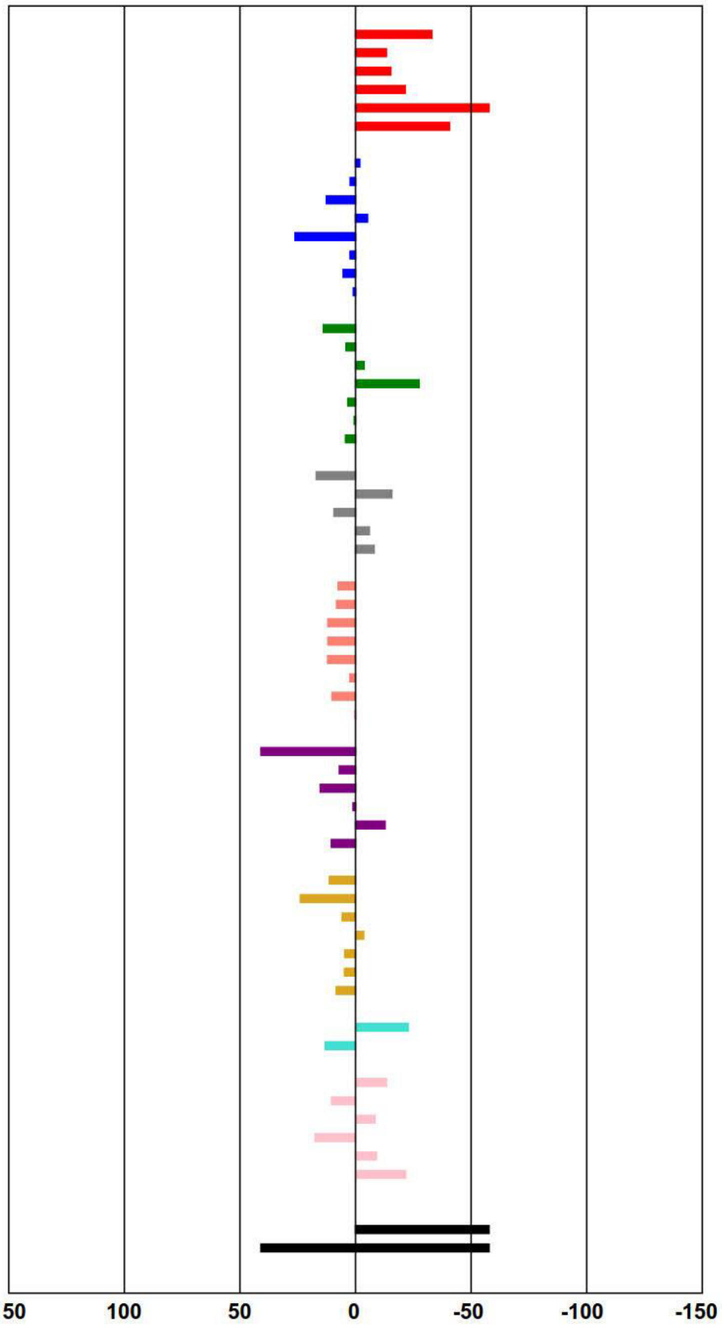

Figure S19. One dose mean graph of NCI-60 cell line screening data for 9d

| Developmental Therapeutics Program One Dose Mean Graph | NSC: D-850906 / 1 | Conc: 1.000E-5 M | Test Date: Jan 8, 2024 |
|---|---|---|---|
| | Experiment ID: 2401HT24 | | Report Date: Feb 20, 2024 |

| Panel/Cell Line | Growth Percent |
|---|---|
| Leukemia | |
| CCRF-CEM | -98.89 |
| HL-60(TB) | -98.56 |
| K-562 | 48.36 |
| MOLT-4 | -99.48 |
| RPMI-8226 | -97.65 |
| Non-Small Cell Lung Cancer | |
| A549/ATCC | 57.25 |
| EKVX | 52.92 |
| HOP-62 | -29.84 |
| HOP-92 | -17.26 |
| NCI-H226 | 47.15 |
| NCI-H23 | 41.45 |
| NCI-H322M | 84.16 |
| NCI-H460 | 38.44 |
| NCI-H522 | 32.38 |
| Colon Cancer | |
| COLO 205 | 53.20 |
| HCC-2998 | 50.48 |
| HCT-116 | 38.38 |
| HCT-15 | 43.78 |
| HT29 | 33.58 |
| KM12 | -22.70 |
| SW-620 | 44.76 |
| CNS Cancer | |
| SF-268 | 19.57 |
| SF-295 | 55.36 |
| SF-539 | 33.28 |
| SNB-19 | 50.51 |
| SNB-75 | -8.25 |
| U251 | 65.40 |
| Melanoma | |
| LOX IMVI | 53.34 |
| MALME-3M | 60.21 |
| M14 | 51.22 |
| MDA-MB-435 | 23.74 |
| SK-MEL-2 | -65.65 |
| SK-MEL-28 | 59.43 |
| SK-MEL-5 | 33.48 |
| UACC-257 | 42.16 |
| UACC-62 | 51.26 |
| Ovarian Cancer | |
| IGROV1 | 22.80 |
| OVCAR-3 | 26.94 |
| OVCAR-4 | 40.28 |
| OVCAR-8 | 49.09 |
| NCI/ADR-RES | 60.17 |
| SK-OV-3 | 74.36 |
| Renal Cancer | |
| 786-0 | 59.35 |
| A498 | 59.29 |
| ACHN | 70.59 |
| CAKI-1 | 49.54 |
| RXF 393 | -24.68 |
| SN12C | 45.67 |
| TK-10 | 61.09 |
| Prostate Cancer | |
| PC-3 | 6.90 |
| DU-145 | 64.93 |
| Breast Cancer | |
| MCF7 | -2.81 |
| HS 578T | -13.39 |
| BT-549 | 15.85 |
| MDA-MB-468 | -73.94 |
| Mean | 23.98 |
| Delta | 123.46 |
| Range | 183.64 |

Mean Growth Percent - Growth Percent

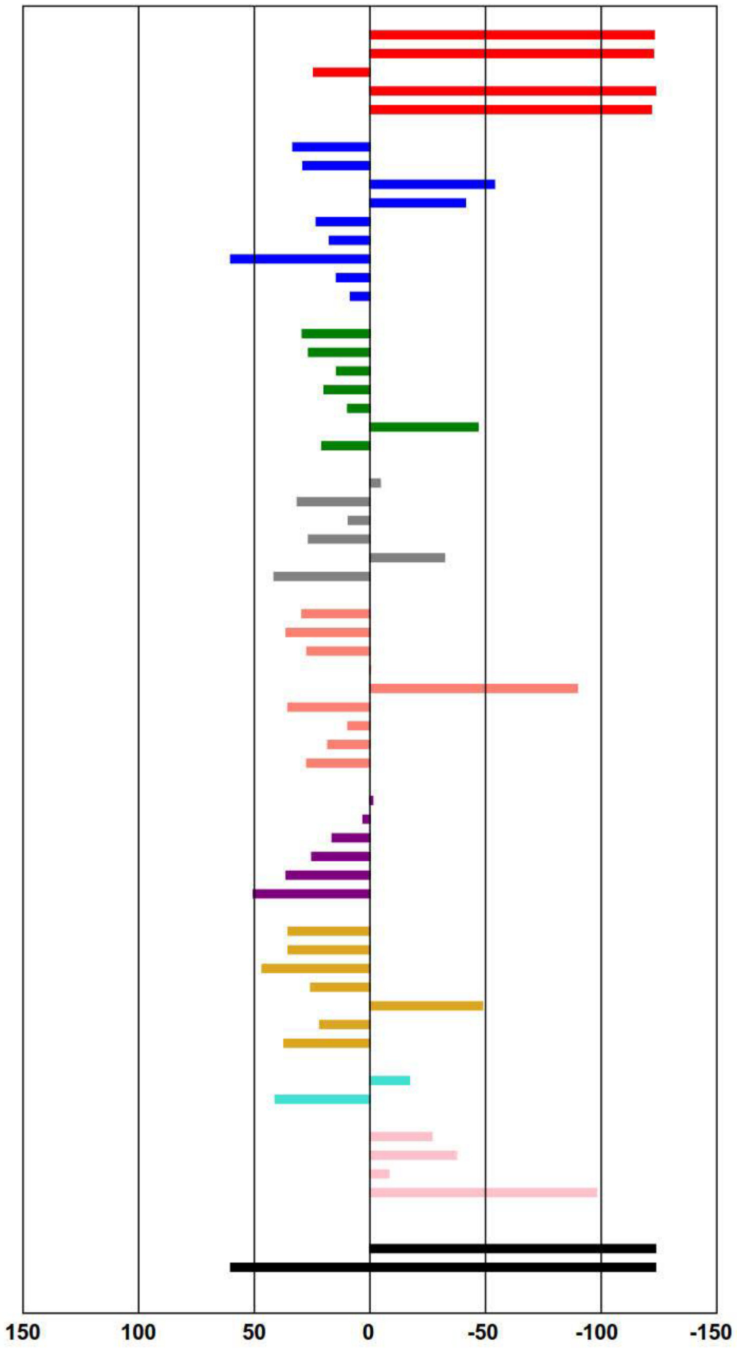

Figure S20. One dose mean graph of NCI-60 cell line screening data for 10d

| Developmental Therapeutics Program One Dose Mean Graph | NSC: D-850907 / 1 | Conc: 1.000E-5 M | Test Date: Jan 8, 2024 |
|---|---|---|---|
| | Experiment ID: 2401HT24 | | Report Date: Feb 20, 2024 |

| Panel/Cell Line | Growth Percent |
|---|---|
| Leukemia | |
| CCRF-CEM | -98.14 |
| HL-60(TB) | -99.18 |
| K-562 | 87.09 |
| MOLT-4 | -99.21 |
| RPMI-8226 | -98.36 |
| Non-Small Cell Lung Cancer | |
| A549/ATCC | 37.01 |
| EKVX | 5.96 |
| HOP-62 | -4.39 |
| HOP-92 | -5.69 |
| NCI-H226 | -3.96 |
| NCI-H23 | -22.24 |
| NCI-H322M | 76.73 |
| NCI-H460 | 45.00 |
| NCI-H522 | -98.70 |
| Colon Cancer | |
| COLO 205 | 22.31 |
| HCC-2998 | 85.18 |
| HCT-116 | -4.09 |
| HCT-15 | -10.28 |
| HT29 | 32.34 |
| KM12 | -38.06 |
| SW-620 | -60.75 |
| CNS Cancer | |
| SF-268 | 6.85 |
| SF-295 | 56.68 |
| SF-539 | -5.90 |
| SNB-19 | 13.62 |
| SNB-75 | -14.67 |
| U251 | 22.59 |
| Melanoma | |
| LOX IMVI | 5.13 |
| MALME-3M | -65.32 |
| M14 | 34.81 |
| MDA-MB-435 | 53.88 |
| SK-MEL-2 | -71.35 |
| SK-MEL-28 | 54.60 |
| SK-MEL-5 | 32.00 |
| UACC-257 | 7.82 |
| UACC-62 | 22.38 |
| Ovarian Cancer | |
| IGROV1 | 26.89 |
| OVCAR-3 | -34.84 |
| OVCAR-4 | -20.86 |
| OVCAR-8 | 31.67 |
| NCI/ADR-RES | 52.05 |
| SK-OV-3 | 66.98 |
| Renal Cancer | |
| 786-0 | 49.50 |
| A498 | 47.88 |
| ACHN | 11.90 |
| CAKI-1 | -66.46 |
| RXF 393 | -76.56 |
| SN12C | 30.34 |
| TK-10 | 12.97 |
| Prostate Cancer | |
| PC-3 | -10.13 |
| DU-145 | 69.27 |
| Breast Cancer | |
| MCF7 | -13.16 |
| HS 578T | -30.68 |
| BT-549 | -63.49 |
| MDA-MB-468 | -87.09 |
| Mean | -1.86 |
| Delta | 97.35 |
| Range | 186.30 |

Mean Growth Percent - Growth Percent

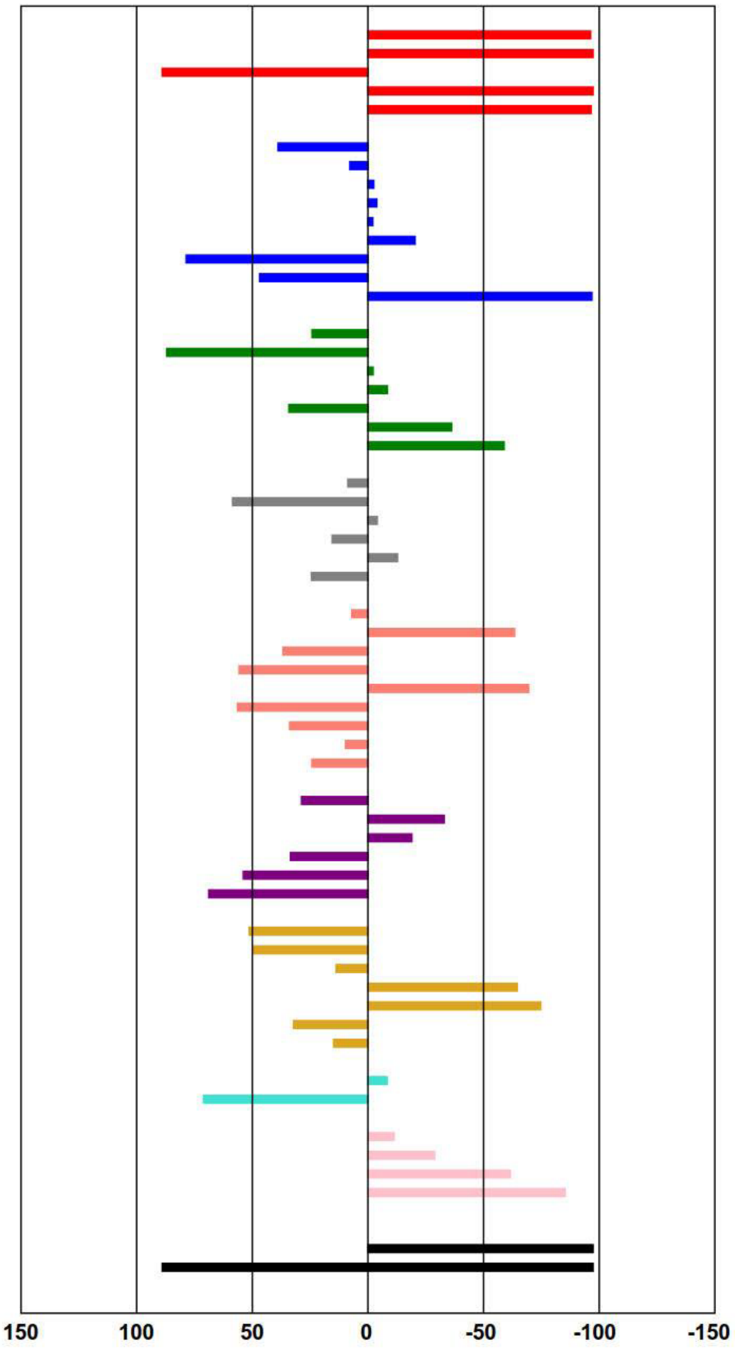

Figure S21. Anticancer screening data of compound 3d at a 5-dose assay

## National Cancer Institute Developmental Therapeutics Program
## In-Vitro Testing Results

| | | | |
|---|---|---|---|
| NSC : D - 845565 / 1 | Experiment ID : 2310NS90 | Test Type : 08 | Units : Molar |
| Report Date : June 06, 2024 | Test Date : October 10, 2023 | QNS : | MC : |
| COMI : TEV-036 | Stain Reagent : SRB Dual-Pass Related | SSPL : 1AJD | |

| Panel/Cell Line | Time Zero | Ctrl | Mean Optical Densities -8.0 | -7.0 | -6.0 | -5.0 | -4.0 | Percent Growth -8.0 | -7.0 | -6.0 | -5.0 | -4.0 | GI50 | TGI | LC50 |
|---|---|---|---|---|---|---|---|---|---|---|---|---|---|---|---|
| **Leukemia** | | | | | | | | | | | | | | | |
| CCRF-CEM | 0.316 | 1.599 | 1.602 | 1.505 | 0.632 | 0.204 | 0.142 | 100 | 93 | 25 | -36 | -55 | 4.24E-7 | 2.56E-6 | 5.49E-5 |
| HL-60(TB) | 0.474 | 2.346 | 2.131 | 2.093 | 2.026 | 0.340 | 0.352 | 88 | 86 | 83 | -28 | -26 | 1.98E-6 | 5.56E-6 | > 1.00E-4 |
| K-562 | 0.135 | 1.335 | 1.286 | 1.303 | 1.034 | 0.189 | 0.151 | 96 | 97 | 75 | 4 | 1 | 2.26E-6 | > 1.00E-4 | > 1.00E-4 |
| MOLT-4 | 0.532 | 2.718 | 2.761 | 2.673 | 2.659 | 0.584 | 0.264 | 102 | 98 | 97 | 2 | -50 | 3.15E-6 | 1.11E-5 | 9.84E-5 |
| RPMI-8226 | 0.801 | 2.611 | 2.671 | 2.561 | 2.538 | 0.802 | 0.531 | 103 | 97 | 96 | 0 | -34 | 3.01E-6 | 1.00E-5 | > 1.00E-4 |
| SR | 0.510 | 2.498 | 2.475 | 2.379 | 2.277 | 0.461 | 0.302 | 99 | 94 | 89 | -10 | -41 | 2.48E-6 | 7.99E-6 | > 1.00E-4 |
| **Non-Small Cell Lung Cancer** | | | | | | | | | | | | | | | |
| A549/ATCC | 0.316 | 1.677 | 1.577 | 1.541 | 1.585 | 1.007 | 0.052 | 93 | 90 | 93 | 51 | -84 | 1.01E-5 | 2.38E-5 | 5.61E-5 |
| EKVX | 0.746 | 2.012 | 1.938 | 1.939 | 1.912 | 1.322 | 0.023 | 94 | 94 | 92 | 45 | -97 | 7.99E-6 | 2.09E-5 | 4.68E-5 |
| HOP-62 | 0.472 | 1.813 | 1.816 | 1.743 | 1.718 | 1.413 | 0.139 | 100 | 95 | 93 | 70 | -71 | 1.39E-5 | 3.15E-5 | 7.13E-5 |
| HOP-92 | 1.030 | 1.870 | 1.848 | 1.820 | 1.809 | 1.272 | 0.171 | 97 | 94 | 93 | 29 | -83 | 4.65E-6 | 1.80E-5 | 5.03E-5 |
| NCI-H226 | 1.191 | 2.423 | 2.350 | 2.305 | 2.264 | 1.766 | 0.253 | 94 | 90 | 87 | 47 | -79 | 8.25E-6 | 2.35E-5 | 5.89E-5 |
| NCI-H23 | 0.671 | 2.096 | 2.084 | 2.071 | 2.065 | 1.357 | 0.078 | 99 | 98 | 98 | 48 | -88 | 9.17E-6 | 2.25E-5 | 5.23E-5 |
| NCI-H322M | 0.838 | 2.138 | 2.125 | 2.175 | 2.079 | 1.938 | 0.031 | 99 | 103 | 95 | 85 | -96 | 1.55E-5 | 2.94E-5 | 5.55E-5 |
| NCI-H460 | 0.323 | 1.915 | 2.099 | 1.869 | 1.853 | 1.116 | 0.028 | 112 | 97 | 96 | 50 | -91 | 9.89E-6 | 2.25E-5 | 5.09E-5 |
| NCI-H522 | 0.964 | 2.681 | 2.553 | 2.581 | 2.518 | 1.091 | 0.072 | 93 | 94 | 91 | 7 | -93 | 3.07E-6 | 1.19E-5 | 3.75E-5 |
| **Colon Cancer** | | | | | | | | | | | | | | | |
| COLO 205 | 0.530 | 2.242 | 2.267 | 2.279 | 2.368 | 0.634 | 0.113 | 101 | 102 | 107 | 6 | -79 | 3.68E-6 | 1.18E-5 | 4.58E-5 |
| HCC-2998 | 1.164 | 3.292 | 3.207 | 3.260 | 3.259 | 2.642 | 0.060 | 96 | 98 | 98 | 69 | -95 | 1.31E-5 | 2.65E-5 | 5.33E-5 |
| HCT-116 | 0.194 | 2.510 | 2.553 | 2.476 | 2.401 | 0.141 | 0.171 | 102 | 99 | 95 | -28 | -12 | 2.34E-6 | 5.96E-6 | > 1.00E-4 |
| HCT-15 | 0.361 | 2.409 | 2.257 | 2.258 | 2.181 | 0.705 | 0.050 | 93 | 93 | 89 | 17 | -86 | 3.46E-6 | 1.45E-5 | 4.45E-5 |
| HT29 | 0.203 | 1.186 | 1.206 | 1.215 | 1.169 | 0.116 | 0.049 | 102 | 103 | 98 | -43 | -76 | 2.20E-6 | 4.96E-6 | 1.62E-5 |
| KM12 | 0.662 | 2.149 | 2.244 | 1.988 | 2.254 | 1.299 | 0.045 | 106 | 89 | 107 | 43 | -93 | 7.74E-6 | 2.06E-5 | 4.81E-5 |
| SW-620 | 0.309 | 1.468 | 1.518 | 1.439 | 1.211 | 0.044 | 0.039 | 104 | 97 | 78 | -86 | -88 | 1.48E-6 | 2.99E-6 | 6.05E-6 |
| **CNS Cancer** | | | | | | | | | | | | | | | |
| SF-268 | 0.932 | 2.386 | 2.320 | 2.278 | 2.202 | 1.739 | 0.165 | 95 | 93 | 87 | 56 | -82 | 1.10E-5 | 2.53E-5 | 5.83E-5 |
| SF-295 | 1.028 | 2.931 | 2.949 | 2.728 | 2.676 | 2.447 | 0.057 | 101 | 89 | 87 | 75 | -94 | 1.40E-5 | 2.76E-5 | 5.46E-5 |
| SF-539 | 0.944 | 2.642 | 2.665 | 2.441 | 2.686 | 2.446 | 0.021 | 101 | 88 | 103 | 88 | -98 | 1.61E-5 | 2.99E-5 | 5.54E-5 |
| SNB-19 | 0.787 | 2.163 | 2.081 | 2.016 | 2.015 | 1.764 | 0.005 | 94 | 89 | 89 | 71 | -99 | 1.33E-5 | 2.61E-5 | 5.13E-5 |
| SNB-75 | 1.063 | 1.967 | 1.897 | 1.824 | 1.876 | 1.368 | 0.098 | 92 | 84 | 90 | 34 | -91 | 5.12E-6 | 1.86E-5 | 4.70E-5 |
| U251 | 0.232 | 1.205 | 1.178 | 1.125 | 1.165 | 0.673 | 0.026 | 97 | 92 | 96 | 45 | -89 | 8.09E-6 | 2.18E-5 | 5.12E-5 |
| **Melanoma** | | | | | | | | | | | | | | | |
| LOX IMVI | 0.425 | 2.170 | 2.074 | 2.045 | 2.011 | 0.139 | 0.028 | 94 | 93 | 91 | -67 | -93 | 1.81E-6 | 3.75E-6 | 7.76E-6 |
| MALME-3M | 0.650 | 1.429 | 1.391 | 1.392 | 1.351 | 0.141 | 0.054 | 95 | 95 | 90 | -78 | -92 | 1.73E-6 | 3.42E-6 | 6.78E-6 |
| M14 | 0.408 | 1.736 | 1.692 | 1.647 | 1.628 | 0.629 | 0.143 | 97 | 93 | 92 | 17 | -65 | 3.60E-6 | 1.60E-5 | 6.54E-5 |
| MDA-MB-435 | 0.636 | 2.329 | 2.208 | 2.226 | 2.218 | 1.161 | 0.030 | 93 | 94 | 93 | 31 | -95 | 4.96E-6 | 1.76E-5 | 4.38E-5 |
| SK-MEL-2 | 1.172 | 2.487 | 2.445 | 2.380 | 2.365 | 1.868 | 0.099 | 97 | 92 | 91 | 53 | -92 | 1.05E-5 | 2.32E-5 | 5.16E-5 |
| SK-MEL-28 | 0.693 | 2.184 | 2.044 | 2.048 | 2.090 | 0.940 | 0.013 | 91 | 91 | 94 | 17 | -98 | 3.68E-6 | 1.39E-5 | 3.80E-5 |
| SK-MEL-5 | 1.062 | 2.947 | 2.799 | 2.635 | 2.708 | 1.487 | 0.034 | 92 | 83 | 87 | 23 | -97 | 3.77E-6 | 1.54E-5 | 4.05E-5 |
| UACC-257 | 1.151 | 2.615 | 2.538 | 2.469 | 2.465 | 1.680 | 0.087 | 95 | 90 | 90 | 36 | -92 | 5.51E-6 | 1.91E-5 | 4.68E-5 |
| UACC-62 | 0.704 | 2.441 | 2.045 | 2.035 | 2.048 | 1.280 | 0.027 | 77 | 77 | 77 | 33 | -96 | 4.16E-6 | 1.80E-5 | 4.39E-5 |
| **Ovarian Cancer** | | | | | | | | | | | | | | | |
| IGROV1 | 0.591 | 1.916 | 2.085 | 1.948 | 1.923 | 1.074 | 0.108 | 113 | 102 | 100 | 36 | -82 | 6.14E-6 | 2.03E-5 | 5.39E-5 |
| OVCAR-3 | 0.858 | 2.355 | 2.331 | 2.228 | 2.067 | 0.188 | 0.007 | 98 | 92 | 81 | -78 | -99 | 1.56E-6 | 3.22E-6 | 6.65E-6 |
| OVCAR-4 | 0.794 | 2.358 | 2.216 | 2.144 | 2.171 | 1.246 | 0.121 | 91 | 86 | 88 | 29 | -85 | 4.39E-6 | 1.79E-5 | 4.94E-5 |
| OVCAR-5 | 0.643 | 1.628 | 1.475 | 1.487 | 1.530 | 1.373 | 0.029 | 84 | 86 | 90 | 74 | -95 | 1.39E-5 | 2.74E-5 | 5.39E-5 |
| OVCAR-8 | 0.495 | 2.114 | 2.060 | 1.983 | 2.023 | 1.399 | 0.106 | 97 | 92 | 94 | 56 | -79 | 1.10E-5 | 2.60E-5 | 6.12E-5 |
| NCI/ADR-RES | 0.372 | 1.207 | 1.179 | 1.158 | 1.133 | 0.620 | 0.081 | 97 | 94 | 91 | 30 | -78 | 4.67E-6 | 1.88E-5 | 5.48E-5 |
| SK-OV-3 | 0.624 | 1.676 | 1.756 | 1.753 | 1.760 | 1.428 | 0.127 | 108 | 107 | 108 | 76 | -80 | 1.48E-5 | 3.09E-5 | 6.46E-5 |
| **Renal Cancer** | | | | | | | | | | | | | | | |
| 786-0 | 0.440 | 2.571 | 2.492 | 2.384 | 2.428 | 0.289 | 0.174 | 96 | 91 | 93 | -34 | -60 | 2.18E-6 | 5.38E-6 | 3.98E-5 |
| A498 | 1.307 | 2.125 | 2.135 | 2.026 | 2.075 | 2.073 | 0.017 | 101 | 88 | 94 | 94 | -99 | 1.69E-5 | 3.07E-5 | 5.58E-5 |
| ACHN | 0.426 | 1.786 | 1.757 | 1.794 | 1.617 | 0.074 | 0.003 | 98 | 101 | 88 | -83 | -99 | 1.66E-6 | 3.27E-6 | 6.42E-6 |
| CAKI-1 | 0.462 | 1.732 | 1.666 | 1.557 | 1.416 | 0.166 | 0.044 | 95 | 86 | 75 | -64 | -90 | 1.52E-6 | 3.46E-6 | 7.92E-6 |
| RXF 393 | 1.053 | 1.610 | 1.643 | 1.562 | 1.558 | 0.645 | 0.167 | 106 | 91 | 91 | -39 | -84 | 2.06E-6 | 5.01E-6 | 1.77E-5 |
| SN12C | 0.552 | 1.978 | 1.874 | 1.735 | 1.827 | 1.226 | 0.046 | 93 | 83 | 89 | 47 | -92 | 8.62E-6 | 2.19E-5 | 5.01E-5 |
| TK-10 | 0.990 | 2.242 | 2.117 | 2.108 | 2.171 | 1.546 | 0.026 | 90 | 89 | 94 | 44 | -97 | 7.72E-6 | 2.06E-5 | 4.63E-5 |
| UO-31 | 0.704 | 2.093 | 1.895 | 1.853 | 1.794 | 0.152 | 0.025 | 86 | 83 | 78 | -78 | -97 | 1.52E-6 | 3.16E-6 | 6.58E-6 |
| **Prostate Cancer** | | | | | | | | | | | | | | | |
| PC-3 | 0.414 | 1.800 | 1.781 | 1.744 | 1.678 | 0.703 | 0.116 | 99 | 96 | 91 | 21 | -72 | 3.85E-6 | 1.68E-5 | 5.78E-5 |
| DU-145 | 0.339 | 1.322 | 1.275 | 1.198 | 1.275 | 0.744 | 0.004 | 95 | 87 | 95 | 41 | -99 | 6.86E-6 | 1.97E-5 | 4.47E-5 |
| **Breast Cancer** | | | | | | | | | | | | | | | |
| MCF7 | 0.420 | 1.907 | 1.873 | 1.670 | 1.830 | 0.452 | 0.131 | 98 | 84 | 95 | 2 | -69 | 3.04E-6 | 1.07E-5 | 5.42E-5 |
| MDA-MB-231/ATCC | 0.549 | 1.132 | 1.124 | 1.084 | 1.105 | 0.773 | 0.068 | 99 | 92 | 95 | 38 | -88 | 6.25E-6 | 2.02E-5 | 5.03E-5 |
| HS 578T | 1.661 | 2.664 | 2.493 | 2.489 | 2.474 | 2.149 | 1.060 | 83 | 83 | 81 | 49 | -36 | 9.09E-6 | 3.74E-5 | > 1.00E-4 |
| BT-549 | 1.156 | 2.417 | 2.347 | 2.267 | 2.282 | 1.686 | 0.357 | 94 | 88 | 89 | 42 | -69 | 6.78E-6 | 2.39E-5 | 6.72E-5 |
| T-47D | 0.631 | 1.623 | 1.517 | 1.544 | 1.422 | 0.326 | 0.412 | 89 | 92 | 80 | -48 | -35 | 1.71E-6 | 4.19E-6 | > 1.00E-4 |
| MDA-MB-468 | 1.202 | 1.926 | 1.955 | 1.856 | 1.885 | 0.628 | 0.192 | 104 | 90 | 94 | -48 | -84 | 2.05E-6 | 4.61E-6 | 1.15E-5 |

Figure S22. Anticancer screening data of compound 3d at a 5-dose assay

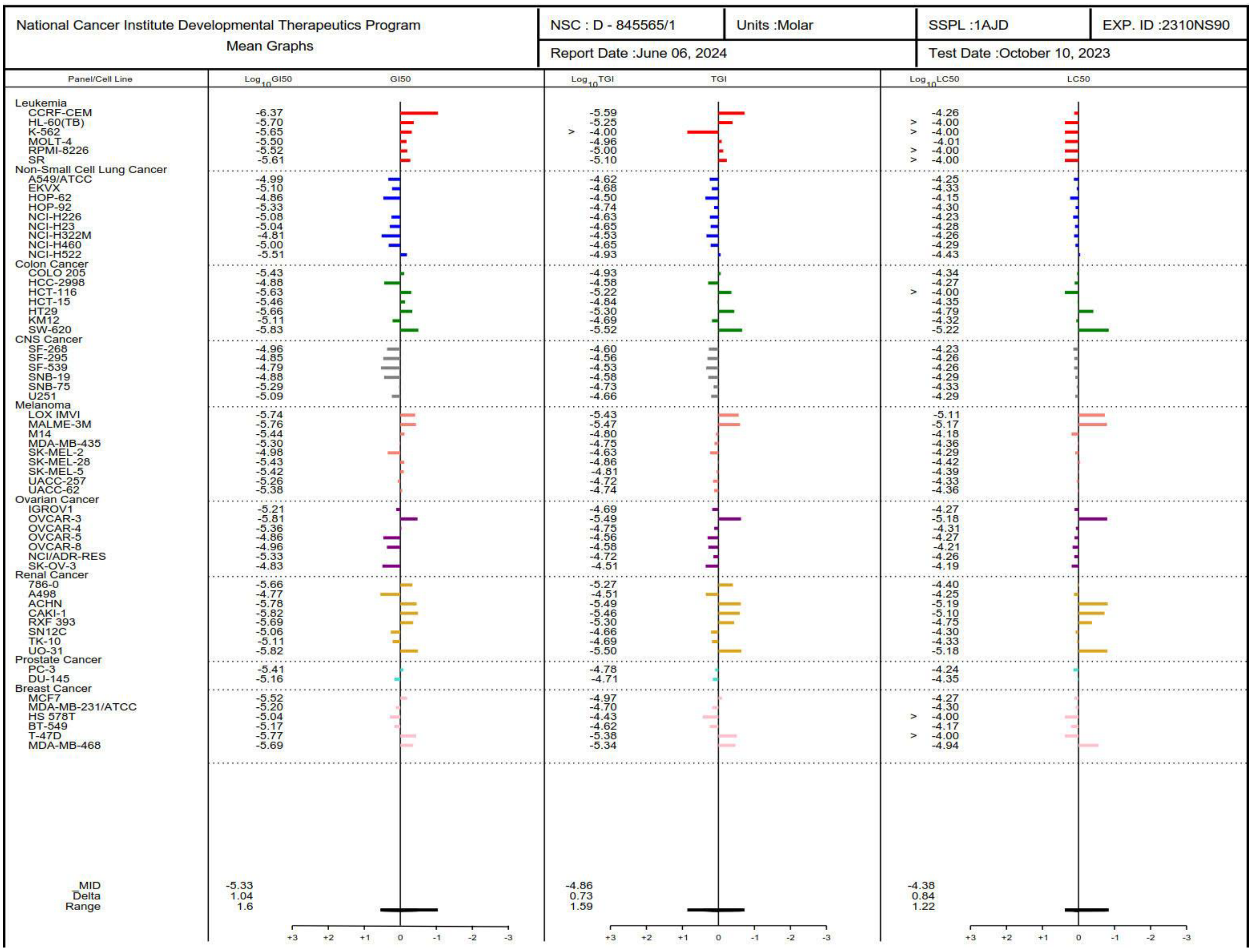

Figure S23. Anticancer screening data of compound 3d at a 5-dose assay

| National Cancer Institute Developmental Therapeutics Program Waterfall Graph GI50 | NSC : D - 845565/1 | Units :Molar | SSPL :1AJD | EXP. ID :2310NS90 |
|---|---|---|---|---|
| | Report Date :June 06, 2024 | | Test Date :October 10, 2023 | |

| Panel | Cell Name | Hollow Fiber | GI50 |
|---|---|---|---|
| Leukemia | CCRF-CEM | | -6.37 |
| Colon Cancer | SW-620 | * | -5.83 |
| Renal Cancer | CAKI-1 | | -5.82 |
| Renal Cancer | UO-31 | | -5.82 |
| Ovarian Cancer | OVCAR-3 | * | -5.81 |
| Renal Cancer | ACHN | | -5.78 |
| Breast Cancer | T-47D | | -5.77 |
| Melanoma | MALME-3M | | -5.76 |
| Melanoma | LOX IMVI | * | -5.74 |
| Leukemia | HL-60(TB) | | -5.70 |
| Breast Cancer | MDA-MB-468 | | -5.69 |
| Renal Cancer | RXF 393 | | -5.69 |
| Renal Cancer | 786-0 | | -5.66 |
| Colon Cancer | HT29 | | -5.66 |
| Leukemia | K-562 | | -5.65 |
| Colon Cancer | HCT-116 | | -5.63 |
| Leukemia | SR | | -5.61 |
| Leukemia | RPMI-8226 | | -5.52 |
| Breast Cancer | MCF7 | | -5.52 |
| Non-Small Cell Lung Cancer | NCI-H522 | * | -5.51 |
| Leukemia | MOLT-4 | | -5.50 |
| Colon Cancer | HCT-15 | | -5.46 |
| Melanoma | M14 | | -5.44 |
| Melanoma | SK-MEL-28 | | -5.43 |
| Colon Cancer | COLO 205 | * | -5.43 |
| Melanoma | SK-MEL-5 | | -5.42 |
| Prostate Cancer | PC-3 | | -5.41 |
| Melanoma | UACC-62 | * | -5.38 |
| Ovarian Cancer | OVCAR-4 | | -5.36 |
| Non-Small Cell Lung Cancer | HOP-92 | | -5.33 |
| Ovarian Cancer | NCI/ADR-RES | | -5.33 |
| Melanoma | MDA-MB-435 | * | -5.30 |
| CNS Cancer | SNB-75 | | -5.29 |
| Melanoma | UACC-257 | | -5.26 |
| Ovarian Cancer | IGROV1 | | -5.21 |
| Breast Cancer | MDA-MB-231/ATCC | * | -5.20 |
| Breast Cancer | BT-549 | | -5.17 |
| Prostate Cancer | DU-145 | | -5.16 |
| Renal Cancer | TK-10 | | -5.11 |
| Colon Cancer | KM12 | | -5.11 |
| Non-Small Cell Lung Cancer | EKVX | | -5.10 |
| CNS Cancer | U251 | * | -5.09 |
| Non-Small Cell Lung Cancer | NCI-H226 | | -5.08 |
| Renal Cancer | SN12C | | -5.06 |
| Breast Cancer | HS 578T | | -5.04 |
| Non-Small Cell Lung Cancer | NCI-H23 | * | -5.04 |
| Non-Small Cell Lung Cancer | NCI-H460 | | -5.00 |
| Non-Small Cell Lung Cancer | A549/ATCC | | -4.99 |
| Melanoma | SK-MEL-2 | | -4.98 |
| CNS Cancer | SF-268 | | -4.96 |
| Ovarian Cancer | OVCAR-8 | | -4.96 |
| Colon Cancer | HCC-2998 | | -4.88 |
| CNS Cancer | SNB-19 | | -4.88 |
| Ovarian Cancer | OVCAR-5 | * | -4.86 |
| Non-Small Cell Lung Cancer | HOP-62 | | -4.86 |
| CNS Cancer | SF-295 | * | -4.85 |
| Ovarian Cancer | SK-OV-3 | | -4.83 |
| Non-Small Cell Lung Cancer | NCI-H322M | | -4.81 |
| CNS Cancer | SF-539 | | -4.79 |
| Renal Cancer | A498 | | -4.77 |

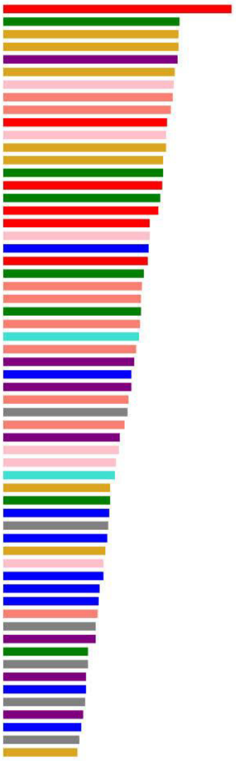

Log10 High Conc :-4.0

Figure S24. Anticancer screening data of compound 3d at a 5-dose assay

| National Cancer Institute Developmental Therapeutics Program Waterfall Graph TGI | NSC : D - 845565/1 | Units :Molar | SSPL :1AJD | EXP. ID :2310NS90 |
|---|---|---|---|---|
| | Report Date :June 06, 2024 | | Test Date :October 10, 2023 | |

| Panel | Cell Name | Hollow Fiber | TGI |
|---|---|---|---|
| Leukemia | CCRF-CEM | | -5.59 |
| Colon Cancer | SW-620 | * | -5.52 |
| Renal Cancer | UO-31 | | -5.50 |
| Ovarian Cancer | OVCAR-3 | * | -5.49 |
| Renal Cancer | ACHN | | -5.49 |
| Melanoma | MALME-3M | | -5.47 |
| Renal Cancer | CAKI-1 | | -5.46 |
| Melanoma | LOX IMVI | * | -5.43 |
| Breast Cancer | T-47D | | -5.38 |
| Breast Cancer | MDA-MB-468 | | -5.34 |
| Colon Cancer | HT29 | | -5.30 |
| Renal Cancer | RXF 393 | | -5.30 |
| Renal Cancer | 786-0 | | -5.27 |
| Leukemia | HL-60(TB) | | -5.25 |
| Colon Cancer | HCT-116 | | -5.22 |
| Leukemia | SR | | -5.10 |
| Leukemia | RPMI-8226 | | -5.00 |
| Breast Cancer | MCF7 | | -4.97 |
| Leukemia | MOLT-4 | | -4.96 |
| Colon Cancer | COLO 205 | * | -4.93 |
| Non-Small Cell Lung Cancer | NCI-H522 | * | -4.93 |
| Melanoma | SK-MEL-28 | | -4.86 |
| Colon Cancer | HCT-15 | | -4.84 |
| Melanoma | SK-MEL-5 | | -4.81 |
| Melanoma | M14 | | -4.80 |
| Prostate Cancer | PC-3 | | -4.78 |
| Melanoma | MDA-MB-435 | * | -4.75 |
| Ovarian Cancer | OVCAR-4 | | -4.75 |
| Non-Small Cell Lung Cancer | HOP-92 | | -4.74 |
| Melanoma | UACC-62 | * | -4.74 |
| CNS Cancer | SNB-75 | | -4.73 |
| Ovarian Cancer | NCI/ADR-RES | | -4.72 |
| Melanoma | UACC-257 | | -4.72 |
| Prostate Cancer | DU-145 | | -4.71 |
| Breast Cancer | MDA-MB-231/ATCC | * | -4.70 |
| Ovarian Cancer | IGROV1 | | -4.69 |
| Renal Cancer | TK-10 | | -4.69 |
| Colon Cancer | KM12 | | -4.69 |
| Non-Small Cell Lung Cancer | EKVX | | -4.68 |
| CNS Cancer | U251 | * | -4.66 |
| Renal Cancer | SN12C | | -4.66 |
| Non-Small Cell Lung Cancer | NCI-H460 | | -4.65 |
| Non-Small Cell Lung Cancer | NCI-H23 | * | -4.65 |
| Melanoma | SK-MEL-2 | | -4.63 |
| Non-Small Cell Lung Cancer | NCI-H226 | | -4.63 |
| Non-Small Cell Lung Cancer | A549/ATCC | | -4.62 |
| Breast Cancer | BT-549 | | -4.62 |
| CNS Cancer | SF-268 | | -4.60 |
| Ovarian Cancer | OVCAR-8 | | -4.58 |
| CNS Cancer | SNB-19 | | -4.58 |
| Colon Cancer | HCC-2998 | | -4.58 |
| Ovarian Cancer | OVCAR-5 | * | -4.56 |
| CNS Cancer | SF-295 | * | -4.56 |
| Non-Small Cell Lung Cancer | NCI-H322M | | -4.53 |
| CNS Cancer | SF-539 | | -4.53 |
| Renal Cancer | A498 | | -4.51 |
| Ovarian Cancer | SK-OV-3 | | -4.51 |
| Non-Small Cell Lung Cancer | HOP-62 | | -4.50 |
| Breast Cancer | HS 578T | | -4.43 |
| Leukemia | K-562 | | > -4.00 |

Log10 High Conc : -4.0

Figure S25. Anticancer screening data of compound 3d at a 5-dose assay

| National Cancer Institute Developmental Therapeutics Program Waterfall Graph LC50 | NSC : D - 845565/1 | Units :Molar | SSPL :1AJD | EXP. ID :2310NS90 |
|---|---|---|---|---|
| | Report Date :June 06, 2024 | | Test Date :October 10, 2023 | |

| Panel | Cell Name | Hollow Fiber | LC50 |
|---|---|---|---|
| Colon Cancer | SW-620 | * | -5.22 |
| Renal Cancer | ACHN | | -5.19 |
| Renal Cancer | UO-31 | | -5.18 |
| Ovarian Cancer | OVCAR-3 | * | -5.18 |
| Melanoma | MALME-3M | | -5.17 |
| Melanoma | LOX IMVI | * | -5.11 |
| Renal Cancer | CAKI-1 | | -5.10 |
| Breast Cancer | MDA-MB-468 | | -4.94 |
| Colon Cancer | HT29 | | -4.79 |
| Renal Cancer | RXF 393 | | -4.75 |
| Non-Small Cell Lung Cancer | NCI-H522 | * | -4.43 |
| Melanoma | SK-MEL-28 | | -4.42 |
| Renal Cancer | 786-0 | | -4.40 |
| Melanoma | SK-MEL-5 | | -4.39 |
| Melanoma | MDA-MB-435 | * | -4.36 |
| Melanoma | UACC-62 | * | -4.36 |
| Colon Cancer | HCT-15 | | -4.35 |
| Prostate Cancer | DU-145 | | -4.35 |
| Colon Cancer | COLO 205 | * | -4.34 |
| Renal Cancer | TK-10 | | -4.33 |
| Melanoma | UACC-257 | | -4.33 |
| Non-Small Cell Lung Cancer | EKVX | | -4.33 |
| CNS Cancer | SNB-75 | | -4.33 |
| Colon Cancer | KM12 | | -4.32 |
| Ovarian Cancer | OVCAR-4 | | -4.31 |
| Renal Cancer | SN12C | | -4.30 |
| Breast Cancer | MDA-MB-231/ATCC | * | -4.30 |
| Non-Small Cell Lung Cancer | HOP-92 | | -4.30 |
| Non-Small Cell Lung Cancer | NCI-H460 | | -4.29 |
| CNS Cancer | U251 | * | -4.29 |
| CNS Cancer | SNB-19 | | -4.29 |
| Melanoma | SK-MEL-2 | | -4.29 |
| Non-Small Cell Lung Cancer | NCI-H23 | * | -4.28 |
| Colon Cancer | HCC-2998 | | -4.27 |
| Ovarian Cancer | IGROV1 | | -4.27 |
| Ovarian Cancer | OVCAR-5 | * | -4.27 |
| Breast Cancer | MCF7 | | -4.27 |
| CNS Cancer | SF-295 | * | -4.26 |
| Ovarian Cancer | NCI/ADR-RES | | -4.26 |
| Leukemia | CCRF-CEM | | -4.26 |
| CNS Cancer | SF-539 | | -4.26 |
| Non-Small Cell Lung Cancer | NCI-H322M | | -4.26 |
| Renal Cancer | A498 | | -4.25 |
| Non-Small Cell Lung Cancer | A549/ATCC | | -4.25 |
| Prostate Cancer | PC-3 | | -4.24 |
| CNS Cancer | SF-268 | | -4.23 |
| Non-Small Cell Lung Cancer | NCI-H226 | | -4.23 |
| Ovarian Cancer | OVCAR-8 | | -4.21 |
| Ovarian Cancer | SK-OV-3 | | -4.19 |
| Melanoma | M14 | | -4.18 |
| Breast Cancer | BT-549 | | -4.17 |
| Non-Small Cell Lung Cancer | HOP-62 | | -4.15 |
| Leukemia | MOLT-4 | | -4.01 |
| Leukemia | HL-60(TB) | | > -4.00 |
| Leukemia | K-562 | | > -4.00 |
| Leukemia | RPMI-8226 | | > -4.00 |
| Leukemia | SR | | > -4.00 |
| Colon Cancer | HCT-116 | | > -4.00 |
| Breast Cancer | HS 578T | | > -4.00 |
| Breast Cancer | T-47D | | > -4.00 |

Log10 High Conc : -4.0

Figure S26. Anticancer screening data of compound 3d at a 5-dose assay

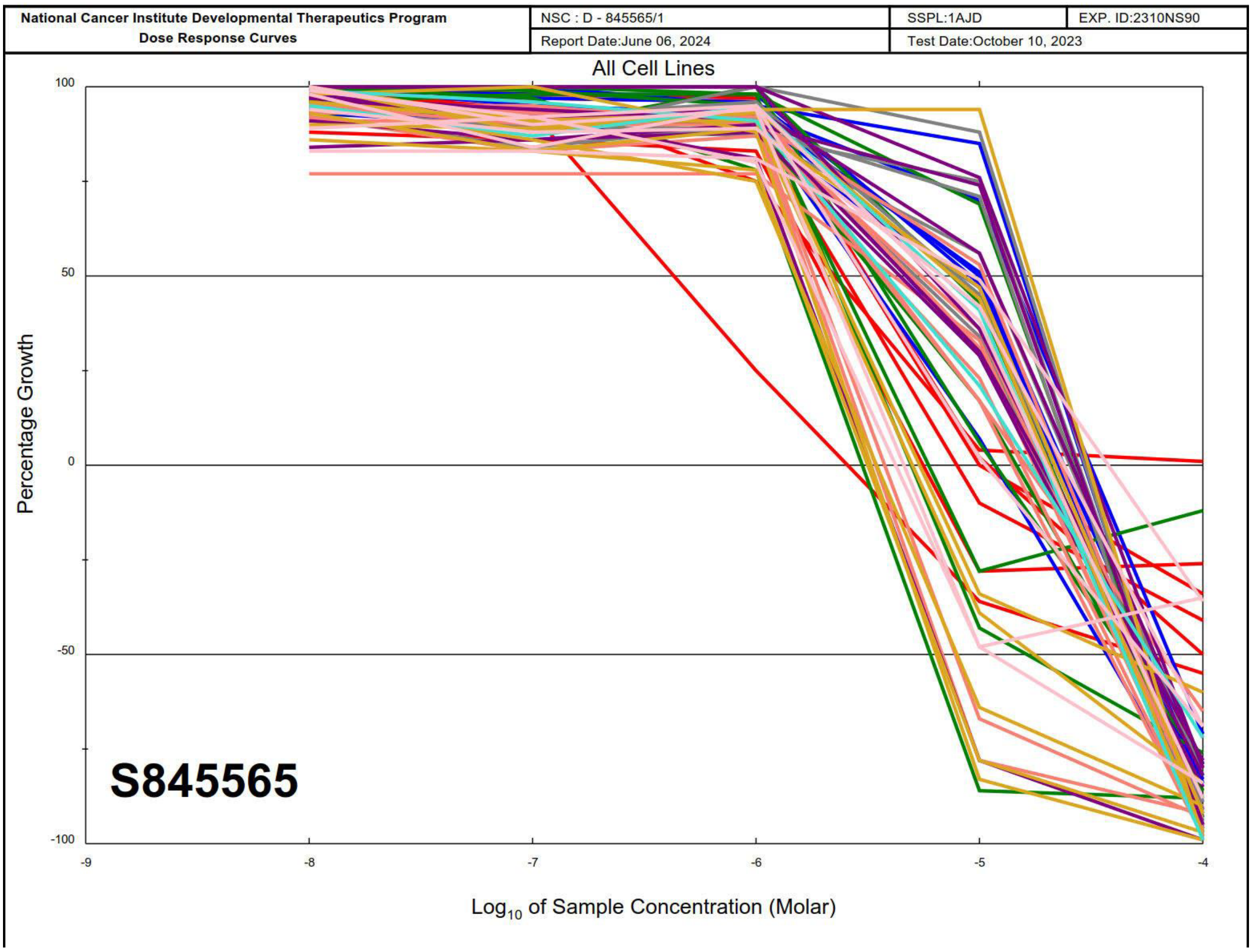

Figure S27. Anticancer screening data of compound 9d at a 5-dose assay

## National Cancer Institute Developmental Therapeutics Program
## In-Vitro Testing Results

| NSC : D - 850906 / 1 | Experiment ID : 2405HT48 | Test Type : HTS | Units : Molar |
|---|---|---|---|
| Report Date : June 18, 2024 | Test Date : May 6, 2024 | QNS : | MC : |
| COMI : SA23 | Stain Reagent : | SSPL : 0XHQ | |

| | | | | | | Log10 Concentration | | | | | | | | | |
|---|---|---|---|---|---|---|---|---|---|---|---|---|---|---|---|
| | Time | | Mean Optical Densities | | | | | Percent Growth | | | | | | | |
| Panel/Cell Line | Zero | Ctrl | -8.2 | -7.2 | -6.2 | -5.2 | -4.2 | -8.2 | -7.2 | -6.2 | -5.2 | -4.2 | GI50 | TGI | LC50 |
| Leukemia | | | | | | | | | | | | | | | |
| CCRF-CEM | 0.669 | 2.838 | 3.305 | 3.289 | 3.116 | 0.051 | 0.001 | 122 | 121 | 113 | -92 | -100 | * 1.21E-6 | * 2.13E-6 | * 3.73E-6 |
| HL-60(TB) | 1.211 | 5.868 | 5.488 | 5.794 | 5.897 | 2.165 | 0.008 | 92 | 99 | 101 | 21 | -99 | * 2.58E-6 | * 8.91E-6 | * 2.33E-5 |
| K-562 | 0.743 | 8.968 | 7.849 | 8.477 | 9.067 | 7.611 | 1.541 | 86 | 94 | 101 | 84 | 10 | * 1.71E-5 | > 6.00E-5 | > 6.00E-5 |
| MOLT-4 | 0.968 | 4.279 | 4.859 | 4.675 | 4.785 | 0.043 | 0.002 | 118 | 112 | 116 | -96 | -100 | * 1.23E-6 | * 2.12E-6 | * 3.65E-6 |
| RPMI-8226 | 5.315 | 17.383 | 15.250 | 17.615 | 17.708 | 12.830 | 0.742 | 82 | 102 | 103 | 62 | -86 | * 7.26E-6 | * 1.58E-5 | * 3.43E-5 |
| SR | 1.414 | 5.975 | 6.301 | 6.302 | 6.124 | 3.016 | 0.034 | 107 | 107 | 103 | 35 | -98 | * 3.63E-6 | * 1.10E-5 | * 2.63E-5 |
| Non-Small Cell Lung Cancer | | | | | | | | | | | | | | | |
| A549/ATCC | 0.758 | 5.377 | 5.127 | 4.967 | 5.065 | 4.091 | 1.914 | 95 | 91 | 93 | 72 | 25 | * 1.77E-5 | > 6.00E-5 | > 6.00E-5 |
| EKVX | 0.660 | NaN | NaN | NaN | NaN | NaN | NaN | 0 | 0 | 0 | 0 | 0 | | | |
| HOP-62 | 1.006 | 2.539 | 2.385 | 3.018 | 2.928 | 2.478 | 1.355 | 90 | 131 | 126 | 96 | 23 | * 2.55E-5 | > 6.00E-5 | > 6.00E-5 |
| HOP-92 | 8.105 | 13.512 | 13.736 | 14.132 | 13.655 | 11.525 | 6.487 | 104 | 111 | 103 | 63 | -20 | * 8.65E-6 | * 3.45E-5 | > 6.00E-5 |
| NCI-H226 | 8.365 | 13.833 | 12.857 | 13.545 | 14.039 | 13.519 | 10.571 | 82 | 95 | 104 | 94 | 40 | * 3.97E-5 | > 6.00E-5 | > 6.00E-5 |
| NCI-H23 | 5.909 | 16.258 | 15.358 | 15.835 | 16.271 | 14.916 | 9.023 | 91 | 96 | 100 | 87 | 30 | * 2.68E-5 | > 6.00E-5 | > 6.00E-5 |
| NCI-H322M | 7.676 | 18.605 | 17.040 | 19.447 | 19.893 | 20.614 | 17.395 | 86 | 108 | 112 | 118 | 89 | > 6.00E-5 | > 6.00E-5 | > 6.00E-5 |
| NCI-H460 | 1.046 | 14.611 | 14.780 | 15.549 | 16.444 | 14.394 | 5.296 | 101 | 107 | 113 | 98 | 31 | * 3.16E-5 | > 6.00E-5 | > 6.00E-5 |
| NCI-H522 | 8.799 | 15.782 | 16.431 | 15.974 | 16.525 | 15.809 | 11.365 | 109 | 103 | 111 | 100 | 37 | * 3.72E-5 | > 6.00E-5 | > 6.00E-5 |
| Colon Cancer | | | | | | | | | | | | | | | |
| COLO 205 | 0.280 | 2.013 | 1.938 | 2.168 | 2.093 | 1.999 | 1.012 | 95 | 109 | 104 | 99 | 42 | * 4.32E-5 | > 6.00E-5 | > 6.00E-5 |
| HCC-2998 | 3.611 | 12.959 | 12.263 | 10.965 | 12.982 | 14.655 | 8.845 | 93 | 78 | 100 | 117 | 56 | > 6.00E-5 | > 6.00E-5 | > 6.00E-5 |
| HCT-116 | 0.468 | 4.957 | 4.891 | 5.188 | 4.931 | 4.350 | 0.594 | 98 | 105 | 99 | 86 | 3 | * 1.64E-5 | > 6.00E-5 | > 6.00E-5 |
| HCT-15 | 1.955 | 16.026 | 16.079 | 16.964 | 15.566 | 14.685 | 4.801 | 100 | 107 | 97 | 90 | 20 | * 2.26E-5 | > 6.00E-5 | > 6.00E-5 |
| HT29 | 0.854 | 6.582 | 6.034 | 6.609 | 6.472 | 6.032 | 2.696 | 90 | 100 | 98 | 90 | 32 | * 2.96E-5 | > 6.00E-5 | > 6.00E-5 |
| KM12 | 0.558 | 3.578 | 3.134 | 3.758 | 3.409 | 3.324 | 1.173 | 85 | 106 | 94 | 92 | 20 | * 2.30E-5 | > 6.00E-5 | > 6.00E-5 |
| SW-620 | 0.670 | 4.418 | 3.980 | 4.444 | 4.482 | 4.401 | 1.613 | 88 | 101 | 102 | 100 | 25 | * 2.78E-5 | > 6.00E-5 | > 6.00E-5 |
| CNS Cancer | | | | | | | | | | | | | | | |
| SF-268 | 1.531 | 3.971 | 3.577 | 4.053 | 3.763 | 3.934 | 1.565 | 84 | 103 | 92 | 98 | 0 | * 1.87E-5 | > 6.00E-5 | > 6.00E-5 |
| SF-295 | 2.119 | 4.891 | 5.198 | 5.054 | 5.041 | 4.793 | 3.303 | 111 | 106 | 105 | 96 | 43 | * 4.39E-5 | > 6.00E-5 | > 6.00E-5 |
| SF-539 | 3.584 | 12.803 | 12.889 | 12.791 | 12.891 | 13.718 | 5.415 | 101 | 100 | 101 | 110 | 20 | * 2.79E-5 | > 6.00E-5 | > 6.00E-5 |
| SNB-19 | 1.306 | 4.951 | 4.517 | 4.784 | 4.934 | 4.798 | 3.289 | 88 | 95 | 99 | 96 | 54 | > 6.00E-5 | > 6.00E-5 | > 6.00E-5 |
| SNB-75 | 2.147 | 3.873 | 3.737 | 3.830 | 3.913 | 3.557 | 2.617 | 92 | 98 | 102 | 82 | 27 | * 2.29E-5 | > 6.00E-5 | > 6.00E-5 |
| U251 | 1.315 | 5.918 | 5.598 | 5.760 | 5.927 | 5.492 | 2.853 | 93 | 97 | 100 | 91 | 33 | * 3.08E-5 | > 6.00E-5 | > 6.00E-5 |
| Melanoma | | | | | | | | | | | | | | | |
| LOX IMVI | 0.775 | 6.026 | 6.093 | 5.696 | 5.523 | 5.327 | 2.172 | 101 | 94 | 90 | 87 | 27 | * 2.45E-5 | > 6.00E-5 | > 6.00E-5 |
| MALME-3M | 9.192 | 13.815 | 14.756 | 15.319 | 15.782 | 15.404 | 11.842 | 120 | 132 | 143 | 134 | 57 | > 6.00E-5 | > 6.00E-5 | > 6.00E-5 |
| M14 | 5.061 | 15.142 | 14.480 | 15.467 | 15.531 | 15.480 | 10.753 | 93 | 103 | 104 | 103 | 57 | > 6.00E-5 | > 6.00E-5 | > 6.00E-5 |
| MDA-MB-435 | 1.306 | 3.821 | 3.631 | 4.119 | 4.255 | 4.034 | 2.516 | 92 | 112 | 117 | 108 | 48 | * 5.59E-5 | > 6.00E-5 | > 6.00E-5 |
| SK-MEL-2 | 2.498 | 6.005 | 5.691 | 5.904 | 5.883 | 5.888 | 1.801 | 91 | 97 | 96 | 97 | -28 | * 1.42E-5 | * 3.58E-5 | > 6.00E-5 |
| SK-MEL-28 | 2.074 | 4.559 | 4.268 | 4.645 | 4.618 | 4.469 | 3.115 | 88 | 103 | 102 | 96 | 42 | * 4.26E-5 | > 6.00E-5 | > 6.00E-5 |
| SK-MEL-5 | 4.050 | 16.902 | 13.660 | 16.506 | 16.205 | 13.699 | 4.655 | 75 | 97 | 95 | 75 | 5 | * 1.37E-5 | > 6.00E-5 | > 6.00E-5 |
| UACC-257 | 2.506 | 5.907 | 6.003 | 5.964 | 6.878 | 6.095 | 3.195 | 103 | 102 | 129 | 106 | 20 | * 2.69E-5 | > 6.00E-5 | > 6.00E-5 |
| UACC-62 | 0.762 | 2.500 | 2.714 | 2.732 | 2.768 | 2.500 | 1.562 | 112 | 113 | 116 | 100 | 46 | * 5.08E-5 | > 6.00E-5 | > 6.00E-5 |
| Ovarian Cancer | | | | | | | | | | | | | | | |
| IGROV1 | 1.580 | 4.838 | 4.982 | 4.984 | 5.225 | 5.288 | 3.487 | 104 | 105 | 112 | 114 | 59 | > 6.00E-5 | > 6.00E-5 | > 6.00E-5 |
| OVCAR-3 | 3.561 | 11.424 | 11.699 | 12.544 | 11.945 | 10.285 | 4.165 | 103 | 114 | 107 | 86 | 8 | * 1.72E-5 | > 6.00E-5 | > 6.00E-5 |
| OVCAR-4 | 6.720 | 10.763 | 11.291 | 11.365 | 11.591 | 10.239 | 6.636 | 113 | 115 | 120 | 87 | -0 | * 1.60E-5 | * 5.99E-5 | > 6.00E-5 |
| OVCAR-5 | 7.651 | 17.282 | 18.372 | 18.219 | 18.078 | 17.935 | 12.208 | 111 | 110 | 108 | 107 | 47 | * 5.41E-5 | > 6.00E-5 | > 6.00E-5 |
| OVCAR-8 | 1.001 | 4.164 | 4.387 | 4.404 | 4.421 | 4.094 | 2.568 | 107 | 108 | 108 | 98 | 50 | * 5.86E-5 | > 6.00E-5 | > 6.00E-5 |
| NCI/ADR-RES | 6.594 | 17.239 | 17.442 | 18.389 | 18.247 | 16.847 | 10.797 | 102 | 111 | 110 | 96 | 40 | * 3.92E-5 | > 6.00E-5 | > 6.00E-5 |
| SK-OV-3 | 3.385 | 8.802 | 8.502 | 7.786 | 8.542 | 8.413 | 4.331 | 95 | 81 | 96 | 93 | 17 | * 2.23E-5 | > 6.00E-5 | > 6.00E-5 |
| Renal Cancer | | | | | | | | | | | | | | | |
| 786-0 | 1.052 | 4.985 | 5.124 | 5.116 | 5.024 | 4.508 | 2.401 | 104 | 103 | 101 | 88 | 34 | * 3.06E-5 | > 6.00E-5 | > 6.00E-5 |
| A498 | 4.985 | 13.176 | 13.502 | 14.066 | 14.019 | 12.306 | 8.124 | 104 | 111 | 110 | 89 | 38 | * 3.54E-5 | > 6.00E-5 | > 6.00E-5 |
| ACHN | 1.200 | 4.467 | 4.710 | 4.645 | 4.476 | 4.415 | 2.982 | 107 | 105 | 100 | 98 | 54 | > 6.00E-5 | > 6.00E-5 | > 6.00E-5 |
| CAKI-1 | 1.194 | 4.909 | 5.025 | 4.789 | 4.643 | 4.080 | 2.505 | 103 | 97 | 93 | 78 | 35 | * 2.70E-5 | > 6.00E-5 | > 6.00E-5 |
| RXF 393 | 2.364 | NaN | NaN | NaN | NaN | NaN | NaN | 0 | 0 | 0 | 0 | 0 | | | |
| SN12C | 1.357 | 4.501 | 4.330 | 3.928 | 3.874 | 3.842 | 2.793 | 95 | 82 | 80 | 79 | 46 | * 4.46E-5 | > 6.00E-5 | > 6.00E-5 |
| TK-10 | 7.325 | 15.791 | 16.183 | 16.765 | 17.344 | 15.826 | 11.521 | 105 | 111 | 118 | 100 | 50 | * 5.89E-5 | > 6.00E-5 | > 6.00E-5 |
| UO-31 | 1.566 | 4.750 | 5.071 | 4.941 | 4.920 | 4.304 | 2.820 | 110 | 106 | 105 | 86 | 39 | * 3.55E-5 | > 6.00E-5 | > 6.00E-5 |
| Prostate Cancer | | | | | | | | | | | | | | | |
| PC-3 | 4.573 | 16.417 | 16.244 | 17.076 | 17.852 | 13.341 | 4.131 | 99 | 106 | 112 | 74 | -10 | * 1.16E-5 | * 4.60E-5 | > 6.00E-5 |
| DU-145 | 1.298 | 4.171 | 4.115 | 4.288 | 4.263 | 4.080 | 3.106 | 98 | 104 | 103 | 97 | 63 | > 6.00E-5 | > 6.00E-5 | > 6.00E-5 |
| Breast Cancer | | | | | | | | | | | | | | | |
| MCF7 | 2.355 | 12.995 | 13.732 | 14.157 | 14.694 | 11.335 | 3.673 | 107 | 111 | 116 | 84 | 12 | * 1.80E-5 | > 6.00E-5 | > 6.00E-5 |
| MDA-MB-231/ATCC | 5.369 | 11.133 | 11.593 | 12.202 | 12.433 | 11.934 | 5.207 | 108 | 119 | 123 | 114 | -3 | * 2.10E-5 | * 5.61E-5 | > 6.00E-5 |
| HS 578T | 1.822 | 3.614 | 3.350 | 3.962 | 3.555 | 3.209 | 2.149 | 85 | 120 | 97 | 77 | 18 | * 1.74E-5 | > 6.00E-5 | > 6.00E-5 |
| BT-549 | 6.607 | 13.936 | 13.181 | 13.263 | 13.840 | 13.904 | 8.446 | 90 | 91 | 99 | 100 | 25 | * 2.78E-5 | > 6.00E-5 | > 6.00E-5 |
| T-47D | 4.991 | 12.143 | 10.681 | 11.078 | 11.504 | 10.371 | 0.805 | 80 | 85 | 91 | 75 | -84 | * 8.64E-6 | * 1.78E-5 | * 3.67E-5 |
| MDA-MB-468 | 7.624 | 8.604 | 8.192 | 8.844 | 8.869 | 8.028 | 0.122 | 58 | 125 | 127 | 41 | -98 | * 4.74E-6 | * 1.18E-5 | * 2.70E-5 |

Figure S28. Anticancer screening data of compound 9d at a 5-dose assay

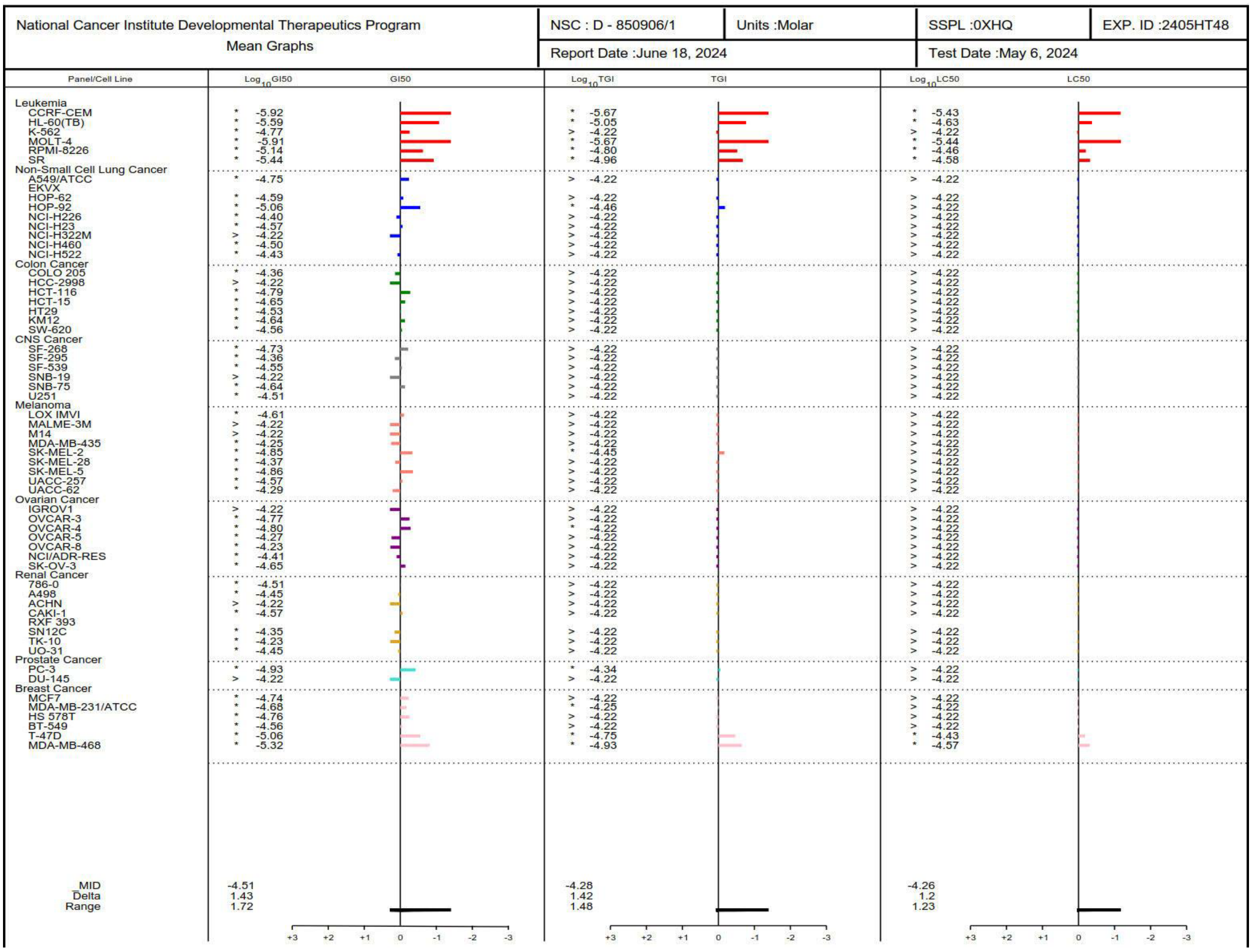

Figure S29. Anticancer screening data of compound 9d at a 5-dose assay

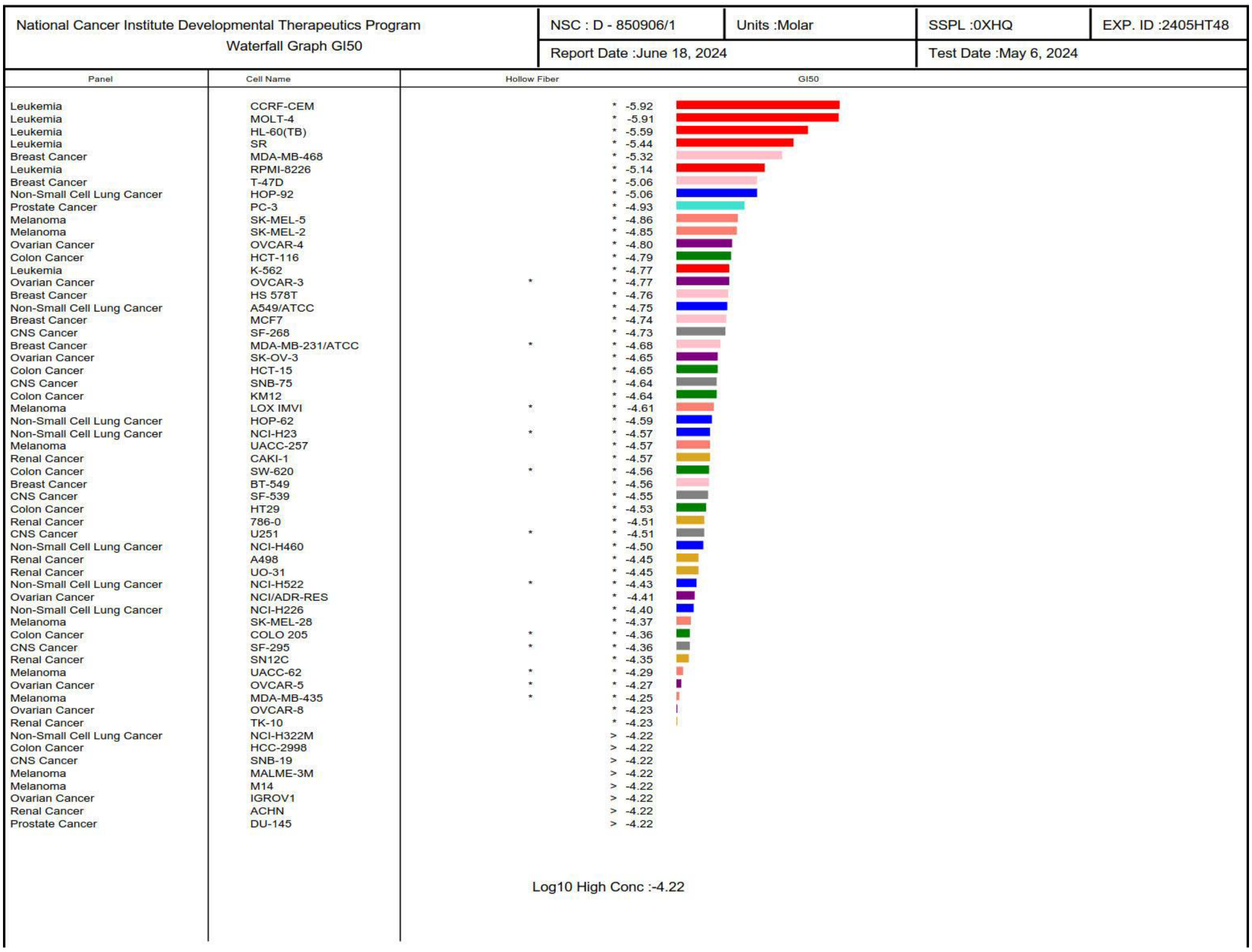

Figure S30. Anticancer screening data of compound 9d at a 5-dose assay

| National Cancer Institute Developmental Therapeutics Program<br>Waterfall Graph TGI | NSC : D - 850906/1 | Units :Molar | SSPL :0XHQ | EXP. ID :2405HT48 |
|---|---|---|---|---|
| | Report Date :June 18, 2024 | | Test Date :May 6, 2024 | |

| Panel | Cell Name | Hollow Fiber | TGI |
|---|---|---|---|
| Leukemia | MOLT-4 | | * -5.67 |
| Leukemia | CCRF-CEM | | * -5.67 |
| Leukemia | HL-60(TB) | | * -5.05 |
| Leukemia | SR | | * -4.96 |
| Breast Cancer | MDA-MB-468 | | * -4.93 |
| Leukemia | RPMI-8226 | | * -4.80 |
| Breast Cancer | T-47D | | * -4.75 |
| Non-Small Cell Lung Cancer | HOP-92 | | * -4.46 |
| Melanoma | SK-MEL-2 | | * -4.45 |
| Prostate Cancer | PC-3 | | * -4.34 |
| Breast Cancer | MDA-MB-231/ATCC | * | * -4.25 |
| Ovarian Cancer | OVCAR-4 | | * -4.22 |
| Leukemia | K-562 | | > -4.22 |
| Non-Small Cell Lung Cancer | A549/ATCC | | > -4.22 |
| Non-Small Cell Lung Cancer | HOP-62 | | > -4.22 |
| Non-Small Cell Lung Cancer | NCI-H226 | | > -4.22 |
| Non-Small Cell Lung Cancer | NCI-H23 | * | > -4.22 |
| Non-Small Cell Lung Cancer | NCI-H322M | | > -4.22 |
| Non-Small Cell Lung Cancer | NCI-H460 | | > -4.22 |
| Non-Small Cell Lung Cancer | NCI-H522 | * | > -4.22 |
| Colon Cancer | COLO 205 | * | > -4.22 |
| Colon Cancer | HCC-2998 | | > -4.22 |
| Colon Cancer | HCT-116 | | > -4.22 |
| Colon Cancer | HCT-15 | | > -4.22 |
| Colon Cancer | HT29 | | > -4.22 |
| Colon Cancer | KM12 | | > -4.22 |
| Colon Cancer | SW-620 | * | > -4.22 |
| CNS Cancer | SF-268 | | > -4.22 |
| CNS Cancer | SF-295 | * | > -4.22 |
| CNS Cancer | SF-539 | | > -4.22 |
| CNS Cancer | SNB-19 | | > -4.22 |
| CNS Cancer | SNB-75 | | > -4.22 |
| CNS Cancer | U251 | * | > -4.22 |
| Melanoma | LOX IMVI | * | > -4.22 |
| Melanoma | MALME-3M | | > -4.22 |
| Melanoma | M14 | | > -4.22 |
| Melanoma | MDA-MB-435 | * | > -4.22 |
| Melanoma | SK-MEL-28 | | > -4.22 |
| Melanoma | SK-MEL-5 | | > -4.22 |
| Melanoma | UACC-257 | | > -4.22 |
| Melanoma | UACC-62 | * | > -4.22 |
| Ovarian Cancer | IGROV1 | | > -4.22 |
| Ovarian Cancer | OVCAR-3 | * | > -4.22 |
| Ovarian Cancer | OVCAR-5 | * | > -4.22 |
| Ovarian Cancer | OVCAR-8 | | > -4.22 |
| Ovarian Cancer | NCI/ADR-RES | | > -4.22 |
| Ovarian Cancer | SK-OV-3 | | > -4.22 |
| Renal Cancer | 786-0 | | > -4.22 |
| Renal Cancer | A498 | | > -4.22 |
| Renal Cancer | ACHN | | > -4.22 |
| Renal Cancer | CAKI-1 | | > -4.22 |
| Renal Cancer | SN12C | | > -4.22 |
| Renal Cancer | TK-10 | | > -4.22 |
| Renal Cancer | UO-31 | | > -4.22 |
| Prostate Cancer | DU-145 | | > -4.22 |
| Breast Cancer | MCF7 | | > -4.22 |
| Breast Cancer | HS 578T | | > -4.22 |
| Breast Cancer | BT-549 | | > -4.22 |

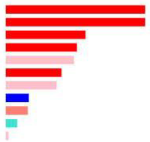

Log10 High Conc : -4.22

Figure S31. Anticancer screening data of compound 9d at a 5-dose assay

| National Cancer Institute Developmental Therapeutics Program<br>Waterfall Graph LC50 | NSC : D - 850906/1 | Units :Molar | SSPL :0XHQ | EXP. ID :2405HT48 |
|---|---|---|---|---|
| | Report Date :June 18, 2024 | | Test Date :May 6, 2024 | |

| Panel | Cell Name | Hollow Fiber | LC50 |
|---|---|---|---|
| Leukemia | MOLT-4 | | * -5.44 |
| Leukemia | CCRF-CEM | | * -5.43 |
| Leukemia | HL-60(TB) | | * -4.63 |
| Leukemia | SR | | * -4.58 |
| Breast Cancer | MDA-MB-468 | | * -4.57 |
| Leukemia | RPMI-8226 | | * -4.46 |
| Breast Cancer | T-47D | | * -4.43 |
| Leukemia | K-562 | | > -4.22 |
| Non-Small Cell Lung Cancer | A549/ATCC | | > -4.22 |
| Non-Small Cell Lung Cancer | HOP-62 | | > -4.22 |
| Non-Small Cell Lung Cancer | HOP-92 | | > -4.22 |
| Non-Small Cell Lung Cancer | NCI-H226 | | > -4.22 |
| Non-Small Cell Lung Cancer | NCI-H23 | * | > -4.22 |
| Non-Small Cell Lung Cancer | NCI-H322M | | > -4.22 |
| Non-Small Cell Lung Cancer | NCI-H460 | | > -4.22 |
| Non-Small Cell Lung Cancer | NCI-H522 | * | > -4.22 |
| Colon Cancer | COLO 205 | * | > -4.22 |
| Colon Cancer | HCC-2998 | | > -4.22 |
| Colon Cancer | HCT-116 | | > -4.22 |
| Colon Cancer | HCT-15 | | > -4.22 |
| Colon Cancer | HT29 | | > -4.22 |
| Colon Cancer | KM12 | | > -4.22 |
| Colon Cancer | SW-620 | * | > -4.22 |
| CNS Cancer | SF-268 | | > -4.22 |
| CNS Cancer | SF-295 | * | > -4.22 |
| CNS Cancer | SF-539 | | > -4.22 |
| CNS Cancer | SNB-19 | | > -4.22 |
| CNS Cancer | SNB-75 | | > -4.22 |
| CNS Cancer | U251 | * | > -4.22 |
| Melanoma | LOX IMVI | * | > -4.22 |
| Melanoma | MALME-3M | | > -4.22 |
| Melanoma | M14 | | > -4.22 |
| Melanoma | MDA-MB-435 | * | > -4.22 |
| Melanoma | SK-MEL-2 | | > -4.22 |
| Melanoma | SK-MEL-28 | | > -4.22 |
| Melanoma | SK-MEL-5 | | > -4.22 |
| Melanoma | UACC-257 | | > -4.22 |
| Melanoma | UACC-62 | * | > -4.22 |
| Ovarian Cancer | IGROV1 | | > -4.22 |
| Ovarian Cancer | OVCAR-3 | * | > -4.22 |
| Ovarian Cancer | OVCAR-4 | | > -4.22 |
| Ovarian Cancer | OVCAR-5 | * | > -4.22 |
| Ovarian Cancer | OVCAR-8 | | > -4.22 |
| Ovarian Cancer | NCI/ADR-RES | | > -4.22 |
| Ovarian Cancer | SK-OV-3 | | > -4.22 |
| Renal Cancer | 786-0 | | > -4.22 |
| Renal Cancer | A498 | | > -4.22 |
| Renal Cancer | ACHN | | > -4.22 |
| Renal Cancer | CAKI-1 | | > -4.22 |
| Renal Cancer | SN12C | | > -4.22 |
| Renal Cancer | TK-10 | | > -4.22 |
| Renal Cancer | UO-31 | | > -4.22 |
| Prostate Cancer | PC-3 | | > -4.22 |
| Prostate Cancer | DU-145 | | > -4.22 |
| Breast Cancer | MCF7 | | > -4.22 |
| Breast Cancer | MDA-MB-231/ATCC | * | > -4.22 |
| Breast Cancer | HS 578T | | > -4.22 |
| Breast Cancer | BT-549 | | > -4.22 |

Log10 High Conc : -4.22

Figure S32. Anticancer screening data of compound 9d at a 5-dose assay

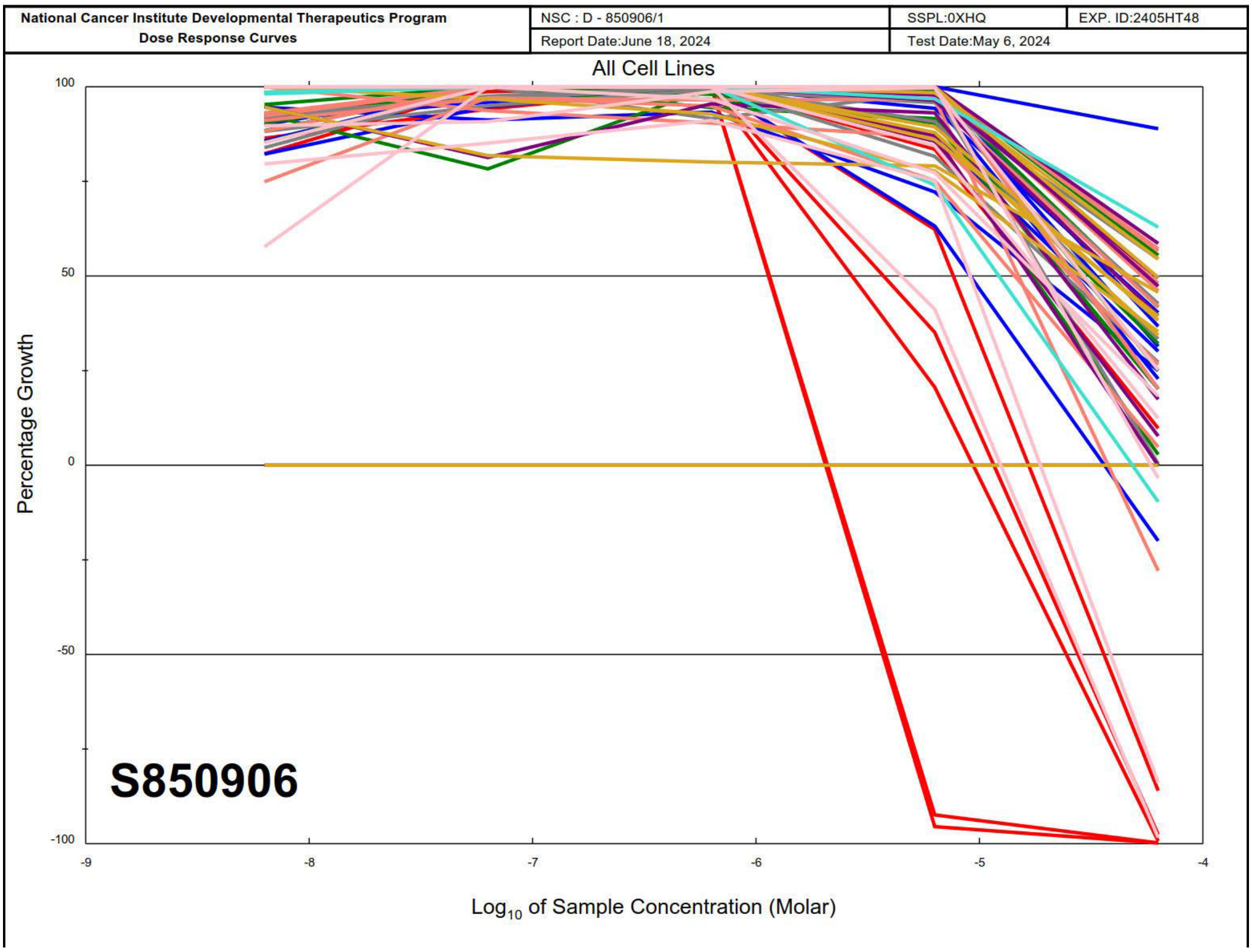

Figure S33. Anticancer screening data of compound 10d at a 5-dose assay

## National Cancer Institute Developmental Therapeutics Program
## In-Vitro Testing Results

| | | | |
|---|---|---|---|
| NSC : D - 850907 / 1 | Experiment ID : 2405HT48 | Test Type : HTS | Units : Molar |
| Report Date : June 18, 2024 | Test Date : May 6, 2024 | QNS : | MC : |
| COMI : SA24 | Stain Reagent : | SSPL : 0XHQ | |

Log10 Concentration

| Panel/Cell Line | Time Zero | Ctrl | Mean Optical Densities -8.0 | -7.0 | -6.0 | -5.0 | -4.0 | Percent Growth -8.0 | -7.0 | -6.0 | -5.0 | -4.0 | GI50 | TGI | LC50 |
|---|---|---|---|---|---|---|---|---|---|---|---|---|---|---|---|
| **Leukemia** | | | | | | | | | | | | | | | |
| CCRF-CEM | 0.669 | 2.838 | 3.372 | 3.307 | 2.574 | 0.013 | 0.003 | 125 | 122 | 88 | -98 | -100 | * 1.60E-6 | * 2.97E-6 | * 5.51E-6 |
| HL-60(TB) | 1.211 | 5.868 | 5.628 | 5.223 | 4.442 | 0.024 | 0.001 | 95 | 87 | 69 | -98 | -100 | * 1.31E-6 | * 2.60E-6 | * 5.17E-6 |
| K-562 | 0.743 | 8.968 | 7.952 | 8.638 | 9.137 | 0.343 | 0.002 | 88 | 96 | 102 | -54 | -100 | * 2.16E-6 | * 4.52E-6 | * 9.45E-6 |
| MOLT-4 | 0.968 | 4.279 | 4.760 | 4.728 | 4.195 | 0.014 | 0.001 | 115 | 114 | 98 | -98 | -100 | * 1.75E-6 | * 3.15E-6 | * 5.66E-6 |
| RPMI-8226 | 5.315 | 17.383 | 15.141 | 17.397 | 16.839 | 2.224 | 0.005 | 81 | 100 | 96 | -58 | -100 | * 1.98E-6 | * 4.18E-6 | * 8.85E-6 |
| SR | 1.414 | 5.975 | 6.403 | 6.559 | 6.261 | 0.355 | 0.001 | 109 | 113 | 106 | -75 | -100 | * 2.05E-6 | * 3.86E-6 | * 7.29E-6 |
| **Non-Small Cell Lung Cancer** | | | | | | | | | | | | | | | |
| A549/ATCC | 0.758 | 5.377 | 5.202 | 5.461 | 4.986 | 2.725 | 0.256 | 96 | 102 | 92 | 43 | -66 | * 7.07E-6 | * 2.46E-5 | * 7.10E-5 |
| EKVX | 0.660 | NaN | NaN | NaN | NaN | NaN | NaN | 0 | 0 | 0 | 0 | 0 | | | |
| HOP-62 | 1.006 | 2.539 | 2.378 | 2.812 | 2.563 | 2.796 | 0.504 | 90 | 118 | 102 | 117 | -50 | * 2.52E-5 | * 5.02E-5 | > 1.00E-4 |
| HOP-92 | 8.105 | 13.512 | 13.606 | 14.009 | 13.669 | 9.329 | 0.025 | 102 | 109 | 103 | 23 | -100 | * 4.56E-6 | * 1.53E-5 | * 3.92E-5 |
| NCI-H226 | 8.365 | 13.833 | 12.769 | 13.380 | 13.664 | 11.961 | 2.086 | 81 | 92 | 97 | 66 | -75 | * 1.29E-5 | * 2.93E-5 | * 6.64E-5 |
| NCI-H23 | 5.909 | 16.258 | 15.357 | 15.644 | 16.035 | 6.958 | 0.052 | 91 | 94 | 98 | 10 | -99 | * 3.51E-6 | * 1.24E-5 | * 3.55E-5 |
| NCI-H322M | 7.676 | 18.605 | 16.956 | 18.974 | 19.532 | 18.243 | 5.220 | 85 | 103 | 109 | 97 | -32 | * 2.31E-5 | * 5.64E-5 | > 1.00E-4 |
| NCI-H460 | 1.046 | 14.611 | 15.058 | 14.779 | 15.195 | 12.297 | 0.020 | 103 | 101 | 104 | 83 | -98 | * 1.52E-5 | * 2.87E-5 | * 5.43E-5 |
| NCI-H522 | 8.799 | 15.782 | 15.753 | 16.123 | 15.896 | 0.155 | 0.038 | 100 | 105 | 102 | -98 | -100 | * 1.81E-6 | * 3.22E-6 | * 5.74E-6 |
| **Colon Cancer** | | | | | | | | | | | | | | | |
| COLO 205 | 0.280 | 2.013 | 1.781 | 2.209 | 2.283 | 0.432 | 0.001 | 86 | 111 | 115 | 3 | -100 | * 3.81E-6 | * 1.07E-5 | * 3.28E-5 |
| HCC-2998 | 3.611 | 12.959 | 10.358 | 12.651 | 12.981 | 12.547 | 1.227 | 72 | 97 | 100 | 96 | -66 | * 1.93E-5 | * 3.92E-5 | * 7.97E-5 |
| HCT-116 | 0.468 | 4.957 | 4.708 | 4.772 | 4.741 | 0.564 | 0.004 | 94 | 96 | 95 | -10 | -99 | * 2.68E-6 | * 7.99E-6 | * 2.80E-5 |
| HCT-15 | 1.955 | 16.026 | 15.185 | 16.905 | 15.456 | 8.754 | 0.061 | 94 | 106 | 96 | 48 | -97 | * 9.20E-6 | * 2.15E-5 | * 4.75E-5 |
| HT29 | 0.854 | 6.582 | 6.304 | 6.398 | 6.714 | 5.127 | 0.015 | 95 | 97 | 102 | 74 | -98 | * 1.39E-5 | * 2.70E-5 | * 5.26E-5 |
| KM12 | 0.558 | 3.578 | 3.475 | 3.368 | 3.918 | 2.737 | 0.018 | 96 | 93 | 112 | 72 | -97 | * 1.35E-5 | * 2.67E-5 | * 5.28E-5 |
| SW-620 | 0.670 | 4.418 | 4.112 | 4.318 | 4.409 | 0.062 | 0.003 | 92 | 97 | 100 | -91 | -100 | * 1.83E-6 | * 3.34E-6 | * 6.11E-6 |
| **CNS Cancer** | | | | | | | | | | | | | | | |
| SF-268 | 1.531 | 3.971 | 3.670 | 3.725 | 3.728 | 2.421 | 0.014 | 88 | 90 | 90 | 36 | -99 | * 5.60E-6 | * 1.86E-5 | * 4.35E-5 |
| SF-295 | 2.119 | 4.891 | 5.047 | 4.954 | 4.981 | 4.193 | 2.032 | 106 | 102 | 103 | 75 | -7 | * 2.01E-5 | * 8.19E-5 | > 1.00E-4 |
| SF-539 | 3.584 | 12.803 | 12.680 | 12.570 | 13.146 | 4.587 | 0.004 | 99 | 98 | 104 | 11 | -100 | * 3.80E-6 | * 1.25E-5 | * 3.55E-5 |
| SNB-19 | 1.306 | 4.951 | 4.466 | 4.992 | 4.913 | 2.467 | 0.037 | 87 | 101 | 99 | 32 | -97 | * 5.37E-6 | * 1.76E-5 | * 4.31E-5 |
| SNB-75 | 2.147 | 3.873 | 3.689 | 3.828 | 3.764 | 2.756 | 0.034 | 89 | 97 | 94 | 35 | -98 | * 5.59E-6 | * 1.83E-5 | * 4.34E-5 |
| U251 | 1.315 | 5.918 | 5.602 | 5.710 | 5.791 | 1.939 | 0.030 | 93 | 96 | 97 | 14 | -98 | * 3.67E-6 | * 1.32E-5 | * 3.72E-5 |
| **Melanoma** | | | | | | | | | | | | | | | |
| LOX IMVI | 0.775 | 6.026 | 5.997 | 5.395 | 5.654 | 1.141 | 0.012 | 99 | 88 | 93 | 7 | -98 | * 3.16E-6 | * 1.16E-5 | * 3.47E-5 |
| MALME-3M | 9.192 | 13.815 | 14.719 | 15.043 | 15.121 | 12.269 | 0.029 | 119 | 127 | 128 | 66 | -100 | * 1.26E-5 | * 2.51E-5 | * 5.02E-5 |
| M14 | 5.061 | 15.142 | 14.209 | 15.282 | 15.018 | 9.458 | 0.026 | 91 | 101 | 99 | 42 | -100 | * 7.29E-6 | * 1.99E-5 | * 4.47E-5 |
| MDA-MB-435 | 1.306 | 3.821 | 3.591 | 4.026 | 4.260 | 3.772 | 0.086 | 91 | 108 | 117 | 98 | -93 | * 1.78E-5 | * 3.25E-5 | * 5.93E-5 |
| SK-MEL-2 | 2.498 | 6.005 | 5.793 | 6.000 | 5.765 | 2.351 | 0.002 | 94 | 100 | 93 | -6 | -100 | * 2.73E-6 | * 8.73E-6 | * 2.95E-5 |
| SK-MEL-28 | 2.074 | 4.559 | 4.318 | 4.558 | 4.768 | 3.545 | 0.023 | 90 | 100 | 108 | 59 | -99 | * 1.14E-5 | * 2.37E-5 | * 4.91E-5 |
| SK-MEL-5 | 4.050 | 16.902 | 13.641 | 16.666 | 16.123 | 10.682 | 0.023 | 75 | 98 | 94 | 52 | -99 | * 1.03E-5 | * 2.20E-5 | * 4.71E-5 |
| UACC-257 | 2.506 | 5.907 | 6.053 | 6.495 | 6.256 | 3.707 | 0.007 | 104 | 117 | 110 | 35 | -100 | * 6.31E-6 | * 1.82E-5 | * 4.27E-5 |
| UACC-62 | 0.762 | 2.500 | 2.418 | 2.693 | 2.713 | 1.170 | 0.004 | 95 | 111 | 112 | 24 | -100 | * 5.04E-6 | * 1.56E-5 | * 3.96E-5 |
| **Ovarian Cancer** | | | | | | | | | | | | | | | |
| IGROV1 | 1.580 | 4.838 | 4.879 | 5.060 | 5.139 | 4.671 | 0.095 | 101 | 107 | 109 | 95 | -94 | * 1.73E-5 | * 3.18E-5 | * 5.85E-5 |
| OVCAR-3 | 3.561 | 11.424 | 11.974 | 11.925 | 11.215 | 5.988 | 0.020 | 107 | 106 | 97 | 31 | -99 | * 5.17E-6 | * 1.73E-5 | * 4.18E-5 |
| OVCAR-4 | 6.720 | 10.763 | 11.163 | 11.256 | 10.974 | 5.888 | 0.024 | 110 | 112 | 105 | -12 | -100 | * 2.95E-6 | * 7.85E-6 | * 2.70E-5 |
| OVCAR-5 | 7.651 | 17.282 | 18.144 | 17.624 | 18.140 | 14.957 | 2.770 | 109 | 104 | 109 | 76 | -64 | * 1.53E-5 | * 3.49E-5 | * 7.96E-5 |
| OVCAR-8 | 1.001 | 4.164 | 4.481 | 4.361 | 4.229 | 2.805 | 0.057 | 110 | 106 | 102 | 57 | -94 | * 1.11E-5 | * 2.38E-5 | * 5.10E-5 |
| NCI/ADR-RES | 6.594 | 17.239 | 17.172 | 18.238 | 17.543 | 14.239 | 0.651 | 100 | 109 | 103 | 72 | -90 | * 1.36E-5 | * 2.78E-5 | * 5.65E-5 |
| SK-OV-3 | 3.385 | 8.802 | 8.337 | 8.142 | 8.890 | 6.727 | 0.742 | 92 | 88 | 102 | 62 | -78 | * 1.22E-5 | * 2.77E-5 | * 6.30E-5 |
| **Renal Cancer** | | | | | | | | | | | | | | | |
| 786-0 | 1.052 | 4.985 | 5.129 | 5.161 | 5.151 | 3.597 | 0.006 | 104 | 105 | 104 | 65 | -99 | * 1.23E-5 | * 2.48E-5 | * 5.00E-5 |
| A498 | 4.985 | 13.176 | 13.756 | 14.172 | 14.039 | 10.749 | 1.379 | 107 | 112 | 111 | 70 | -72 | * 1.39E-5 | * 3.11E-5 | * 6.97E-5 |
| ACHN | 1.200 | 4.467 | 4.727 | 4.587 | 4.474 | 2.265 | 0.006 | 108 | 104 | 100 | 33 | -100 | * 5.53E-6 | * 1.77E-5 | * 4.22E-5 |
| CAKI-1 | 1.194 | 4.909 | 4.952 | 4.656 | 4.499 | 2.292 | 0.005 | 101 | 93 | 89 | 30 | -100 | * 4.53E-6 | * 1.69E-5 | * 4.13E-5 |
| RXF 393 | 2.364 | NaN | NaN | NaN | NaN | NaN | NaN | 0 | 0 | 0 | 0 | 0 | | | |
| SN12C | 1.357 | 4.501 | 4.205 | 3.850 | 4.213 | 3.532 | 0.009 | 91 | 79 | 91 | 69 | -99 | * 1.30E-5 | * 2.57E-5 | * 5.10E-5 |
| TK-10 | 7.325 | 15.791 | 16.469 | 16.615 | 16.767 | 13.344 | 0.099 | 108 | 110 | 112 | 71 | -99 | * 1.33E-5 | * 2.62E-5 | * 5.17E-5 |
| UO-31 | 1.566 | 4.750 | 5.110 | 4.926 | 4.735 | 1.887 | 0.008 | 111 | 106 | 100 | 10 | -100 | * 3.58E-6 | * 1.24E-5 | * 3.54E-5 |
| **Prostate Cancer** | | | | | | | | | | | | | | | |
| PC-3 | 4.573 | 16.417 | 16.493 | 17.634 | 16.642 | 9.287 | 0.476 | 101 | 110 | 102 | 40 | -90 | * 6.85E-6 | * 2.03E-5 | * 4.94E-5 |
| DU-145 | 1.298 | 4.171 | 4.102 | 4.180 | 4.219 | 3.886 | 0.033 | 98 | 100 | 102 | 90 | -97 | * 1.64E-5 | * 3.02E-5 | * 5.59E-5 |
| **Breast Cancer** | | | | | | | | | | | | | | | |
| MCF7 | 2.355 | 12.995 | 14.429 | 14.174 | 13.732 | 6.646 | 0.032 | 114 | 111 | 107 | 40 | -99 | * 7.17E-6 | * 1.95E-5 | * 4.47E-5 |
| MDA-MB-231/ATCC | 5.369 | 11.133 | 11.381 | 11.652 | 12.337 | 11.558 | 0.097 | 104 | 109 | 121 | 107 | -98 | * 1.90E-5 | * 3.33E-5 | * 5.83E-5 |
| HS 578T | 1.822 | 3.614 | 3.158 | 3.303 | 3.404 | 1.995 | 0.018 | 75 | 82 | 88 | 10 | -99 | * 3.08E-6 | * 1.23E-5 | * 3.54E-5 |
| BT-549 | 6.607 | 13.936 | 12.420 | 13.868 | 13.656 | 5.667 | 0.063 | 79 | 99 | 96 | -14 | -99 | * 2.62E-6 | * 7.43E-6 | * 2.64E-5 |
| T-47D | 4.991 | 12.143 | 10.778 | 10.906 | 11.040 | 2.083 | 0.012 | 81 | 83 | 85 | -58 | -100 | * 1.75E-6 | * 3.91E-6 | * 8.75E-6 |
| MDA-MB-468 | 7.624 | 8.604 | 8.644 | 8.917 | 8.164 | 1.529 | 0.008 | 104 | 132 | 55 | -80 | -100 | * 1.09E-6 | * 2.56E-6 | * 6.00E-6 |

Figure S34. Anticancer screening data of compound 10d at a 5-dose assay

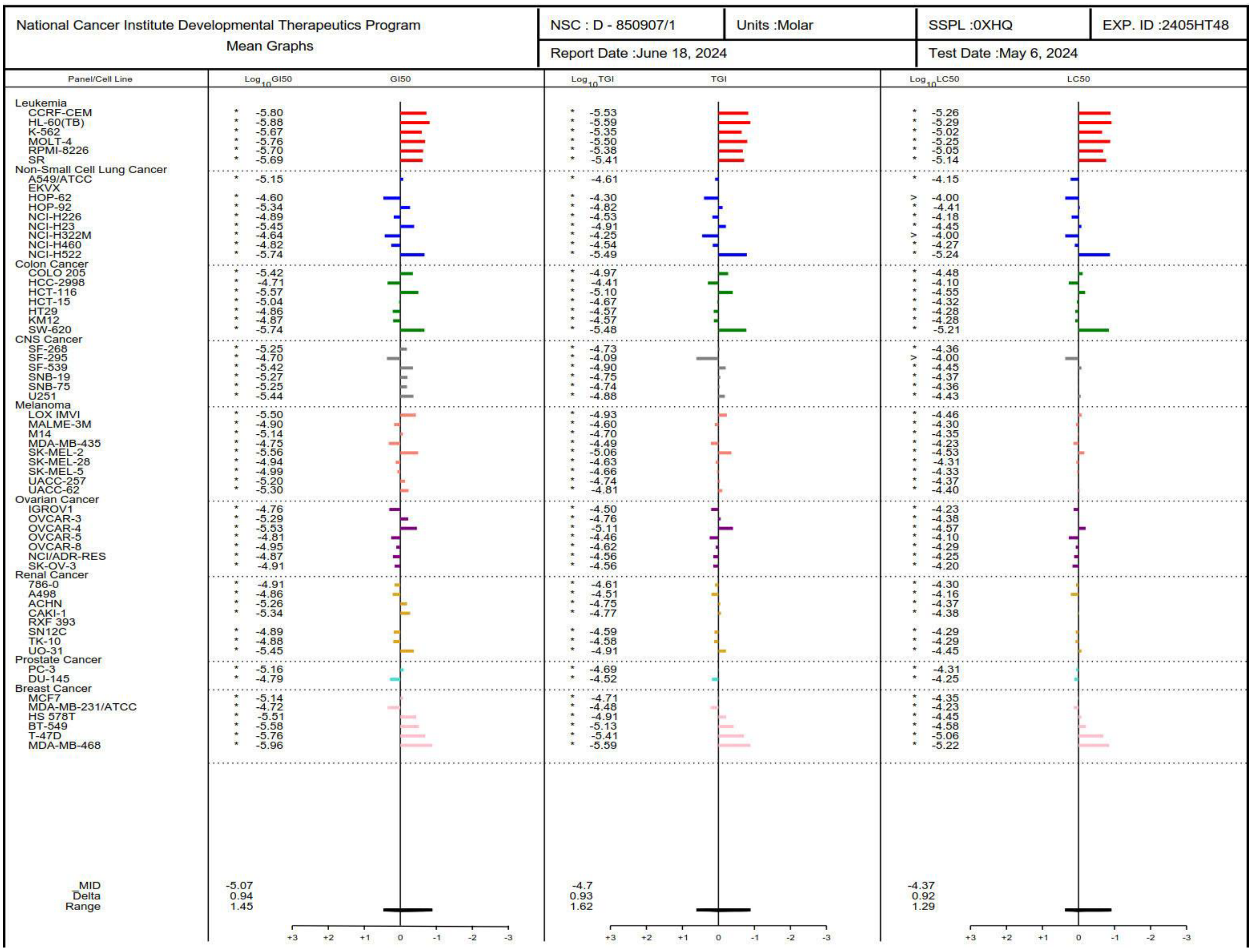

Figure S35. Anticancer screening data of compound 10d at a 5-dose assay

| National Cancer Institute Developmental Therapeutics Program Waterfall Graph GI50 | NSC : D - 850907/1 | Units :Molar | SSPL :0XHQ | EXP. ID :2405HT48 |
|---|---|---|---|---|
| | Report Date :June 18, 2024 | | Test Date :May 6, 2024 | |

| Panel | Cell Name | Hollow Fiber | GI50 |
|---|---|---|---|
| Breast Cancer | MDA-MB-468 | | * -5.96 |
| Leukemia | HL-60(TB) | | * -5.88 |
| Leukemia | CCRF-CEM | | * -5.80 |
| Breast Cancer | T-47D | | * -5.76 |
| Leukemia | MOLT-4 | | * -5.76 |
| Non-Small Cell Lung Cancer | NCI-H522 | * | * -5.74 |
| Colon Cancer | SW-620 | * | * -5.74 |
| Leukemia | RPMI-8226 | | * -5.70 |
| Leukemia | SR | | * -5.69 |
| Leukemia | K-562 | | * -5.67 |
| Breast Cancer | BT-549 | | * -5.58 |
| Colon Cancer | HCT-116 | | * -5.57 |
| Melanoma | SK-MEL-2 | | * -5.56 |
| Ovarian Cancer | OVCAR-4 | | * -5.53 |
| Breast Cancer | HS 578T | | * -5.51 |
| Melanoma | LOX IMVI | * | * -5.50 |
| Non-Small Cell Lung Cancer | NCI-H23 | * | * -5.45 |
| Renal Cancer | UO-31 | | * -5.45 |
| CNS Cancer | U251 | * | * -5.44 |
| CNS Cancer | SF-539 | | * -5.42 |
| Colon Cancer | COLO 205 | * | * -5.42 |
| Renal Cancer | CAKI-1 | | * -5.34 |
| Non-Small Cell Lung Cancer | HOP-92 | | * -5.34 |
| Melanoma | UACC-62 | * | * -5.30 |
| Ovarian Cancer | OVCAR-3 | * | * -5.29 |
| CNS Cancer | SNB-19 | | * -5.27 |
| Renal Cancer | ACHN | | * -5.26 |
| CNS Cancer | SNB-75 | | * -5.25 |
| CNS Cancer | SF-268 | | * -5.25 |
| Melanoma | UACC-257 | | * -5.20 |
| Prostate Cancer | PC-3 | | * -5.16 |
| Non-Small Cell Lung Cancer | A549/ATCC | | * -5.15 |
| Breast Cancer | MCF7 | | * -5.14 |
| Melanoma | M14 | | * -5.14 |
| Colon Cancer | HCT-15 | | * -5.04 |
| Melanoma | SK-MEL-5 | | * -4.99 |
| Ovarian Cancer | OVCAR-8 | | * -4.95 |
| Melanoma | SK-MEL-28 | | * -4.94 |
| Ovarian Cancer | SK-OV-3 | | * -4.91 |
| Renal Cancer | 786-0 | | * -4.91 |
| Melanoma | MALME-3M | | * -4.90 |
| Non-Small Cell Lung Cancer | NCI-H226 | | * -4.89 |
| Renal Cancer | SN12C | | * -4.89 |
| Renal Cancer | TK-10 | | * -4.88 |
| Colon Cancer | KM12 | | * -4.87 |
| Ovarian Cancer | NCI/ADR-RES | | * -4.87 |
| Colon Cancer | HT29 | | * -4.86 |
| Renal Cancer | A498 | | * -4.86 |
| Non-Small Cell Lung Cancer | NCI-H460 | | * -4.82 |
| Ovarian Cancer | OVCAR-5 | * | * -4.81 |
| Prostate Cancer | DU-145 | | * -4.79 |
| Ovarian Cancer | IGROV1 | | * -4.76 |
| Melanoma | MDA-MB-435 | * | * -4.75 |
| Breast Cancer | MDA-MB-231/ATCC | * | * -4.72 |
| Colon Cancer | HCC-2998 | | * -4.71 |
| CNS Cancer | SF-295 | * | * -4.70 |
| Non-Small Cell Lung Cancer | NCI-H322M | | * -4.64 |
| Non-Small Cell Lung Cancer | HOP-62 | | * -4.60 |

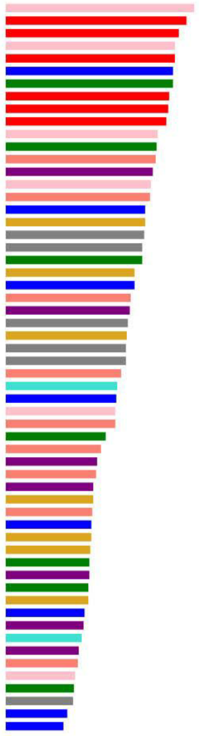

Log10 High Conc :-4.0

Figure S36. Anticancer screening data of compound 10d at a 5-dose assay

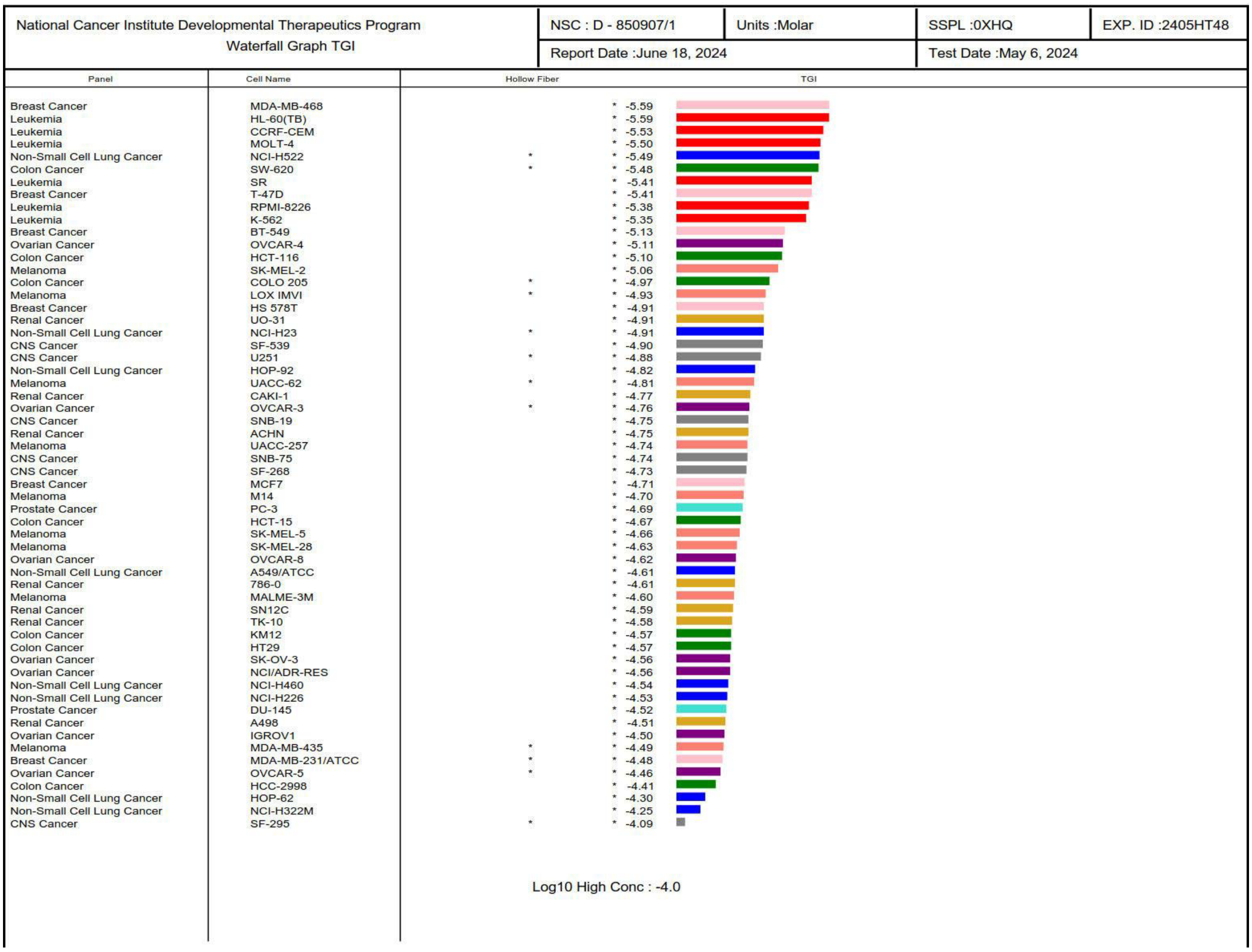

Figure S37. Anticancer screening data of compound 10d at a 5-dose assay

| National Cancer Institute Developmental Therapeutics Program Waterfall Graph LC50 | NSC : D - 850907/1 | Units :Molar | SSPL :0XHQ | EXP. ID :2405HT48 |
|---|---|---|---|---|
| | Report Date :June 18, 2024 | | Test Date :May 6, 2024 | |

| Panel | Cell Name | Hollow Fiber | LC50 |
|---|---|---|---|
| Leukemia | HL-60(TB) | | * -5.29 |
| Leukemia | CCRF-CEM | | * -5.26 |
| Leukemia | MOLT-4 | | * -5.25 |
| Non-Small Cell Lung Cancer | NCI-H522 | * | * -5.24 |
| Breast Cancer | MDA-MB-468 | | * -5.22 |
| Colon Cancer | SW-620 | * | * -5.21 |
| Leukemia | SR | | * -5.14 |
| Breast Cancer | T-47D | | * -5.06 |
| Leukemia | RPMI-8226 | | * -5.05 |
| Leukemia | K-562 | | * -5.02 |
| Breast Cancer | BT-549 | | * -4.58 |
| Ovarian Cancer | OVCAR-4 | | * -4.57 |
| Colon Cancer | HCT-116 | | * -4.55 |
| Melanoma | SK-MEL-2 | | * -4.53 |
| Colon Cancer | COLO 205 | * | * -4.48 |
| Melanoma | LOX IMVI | * | * -4.46 |
| Renal Cancer | UO-31 | | * -4.45 |
| Breast Cancer | HS 578T | | * -4.45 |
| CNS Cancer | SF-539 | | * -4.45 |
| Non-Small Cell Lung Cancer | NCI-H23 | * | * -4.45 |
| CNS Cancer | U251 | * | * -4.43 |
| Non-Small Cell Lung Cancer | HOP-92 | | * -4.41 |
| Melanoma | UACC-62 | * | * -4.40 |
| Renal Cancer | CAKI-1 | | * -4.38 |
| Ovarian Cancer | OVCAR-3 | * | * -4.38 |
| Renal Cancer | ACHN | | * -4.37 |
| Melanoma | UACC-257 | | * -4.37 |
| CNS Cancer | SNB-19 | | * -4.37 |
| CNS Cancer | SNB-75 | | * -4.36 |
| CNS Cancer | SF-268 | | * -4.36 |
| Breast Cancer | MCF7 | | * -4.35 |
| Melanoma | M14 | | * -4.35 |
| Melanoma | SK-MEL-5 | | * -4.33 |
| Colon Cancer | HCT-15 | | * -4.32 |
| Melanoma | SK-MEL-28 | | * -4.31 |
| Prostate Cancer | PC-3 | | * -4.31 |
| Renal Cancer | 786-0 | | * -4.30 |
| Melanoma | MALME-3M | | * -4.30 |
| Renal Cancer | SN12C | | * -4.29 |
| Ovarian Cancer | OVCAR-8 | | * -4.29 |
| Renal Cancer | TK-10 | | * -4.29 |
| Colon Cancer | HT29 | | * -4.28 |
| Colon Cancer | KM12 | | * -4.28 |
| Non-Small Cell Lung Cancer | NCI-H460 | | * -4.27 |
| Prostate Cancer | DU-145 | | * -4.25 |
| Ovarian Cancer | NCI/ADR-RES | | * -4.25 |
| Breast Cancer | MDA-MB-231/ATCC | * | * -4.23 |
| Ovarian Cancer | IGROV1 | | * -4.23 |
| Melanoma | MDA-MB-435 | * | * -4.23 |
| Ovarian Cancer | SK-OV-3 | | * -4.20 |
| Non-Small Cell Lung Cancer | NCI-H226 | | * -4.18 |
| Renal Cancer | A498 | | * -4.16 |
| Non-Small Cell Lung Cancer | A549/ATCC | | * -4.15 |
| Ovarian Cancer | OVCAR-5 | * | * -4.10 |
| Colon Cancer | HCC-2998 | | * -4.10 |
| Non-Small Cell Lung Cancer | HOP-62 | | > -4.00 |
| Non-Small Cell Lung Cancer | NCI-H322M | | > -4.00 |
| CNS Cancer | SF-295 | * | > -4.00 |

Log10 High Conc : -4.0

Figure S38. Anticancer screening data of compound 10d at a 5-dose assay

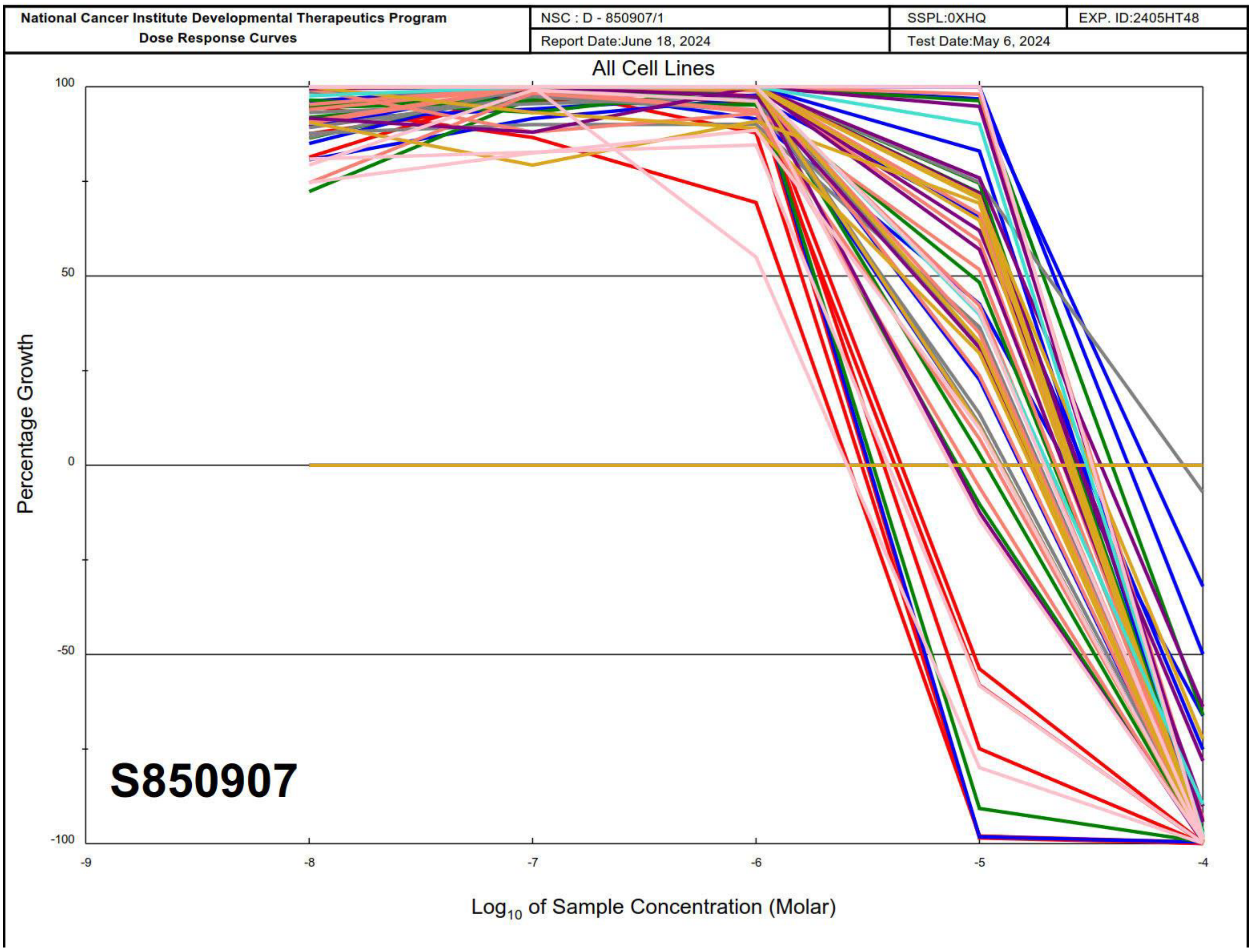

Figure S39. Anticancer screening data of compound 1d at a one dose assay

| Developmental Therapeutics Program | NSC: D-814440 / 1 | Conc: 1.00E-5 Molar | Test Date: Apr 29, 2019 |
|---|---|---|---|
| One Dose Mean Graph | Experiment ID: 1904OS99 | | Report Date: May 16, 2019 |

| Panel/Cell Line | Growth Percent |
|---|---|
| Leukemia | |
| CCRF-CEM | 107.12 |
| HL-60(TB) | 106.12 |
| K-562 | 101.52 |
| MOLT-4 | 95.43 |
| RPMI-8226 | 110.53 |
| SR | 118.98 |
| Non-Small Cell Lung Cancer | |
| A549/ATCC | 95.43 |
| EKVX | 107.43 |
| HOP-62 | 94.99 |
| HOP-92 | 110.66 |
| NCI-H23 | 98.79 |
| NCI-H322M | 100.36 |
| NCI-H460 | 108.19 |
| NCI-H522 | 93.37 |
| Colon Cancer | |
| COLO 205 | 115.75 |
| HCC-2998 | 104.85 |
| HCT-116 | 96.17 |
| HCT-15 | 104.98 |
| HT29 | 107.77 |
| KM12 | 98.36 |
| SW-620 | 108.18 |
| CNS Cancer | |
| SF-268 | 98.73 |
| SF-295 | 98.60 |
| SF-539 | 102.30 |
| SNB-19 | 92.01 |
| SNB-75 | 94.85 |
| U251 | 97.98 |
| Melanoma | |
| LOX IMVI | 100.68 |
| MALME-3M | 99.39 |
| M14 | 104.77 |
| MDA-MB-435 | 103.57 |
| SK-MEL-2 | 94.35 |
| SK-MEL-28 | 102.52 |
| SK-MEL-5 | 99.58 |
| UACC-257 | 98.96 |
| UACC-62 | 93.50 |
| Ovarian Cancer | |
| IGROV1 | 107.07 |
| OVCAR-3 | 106.74 |
| OVCAR-4 | 100.33 |
| OVCAR-5 | 97.88 |
| OVCAR-8 | 101.44 |
| NCI/ADR-RES | 98.07 |
| SK-OV-3 | 103.21 |
| Renal Cancer | |
| 786-0 | 100.56 |
| A498 | 105.76 |
| ACHN | 98.10 |
| CAKI-1 | 96.47 |
| RXF 393 | 111.37 |
| SN12C | 100.55 |
| TK-10 | 110.88 |
| UO-31 | 91.24 |
| Prostate Cancer | |
| PC-3 | 100.65 |
| DU-145 | 109.53 |
| Breast Cancer | |
| MCF7 | 99.65 |
| MDA-MB-231/ATCC | 103.72 |
| HS 578T | 93.47 |
| BT-549 | 102.27 |
| T-47D | 96.98 |
| MDA-MB-468 | 94.86 |
| Mean | 101.65 |
| Delta | 10.41 |
| Range | 27.74 |

Mean Growth Percent - Growth Percent

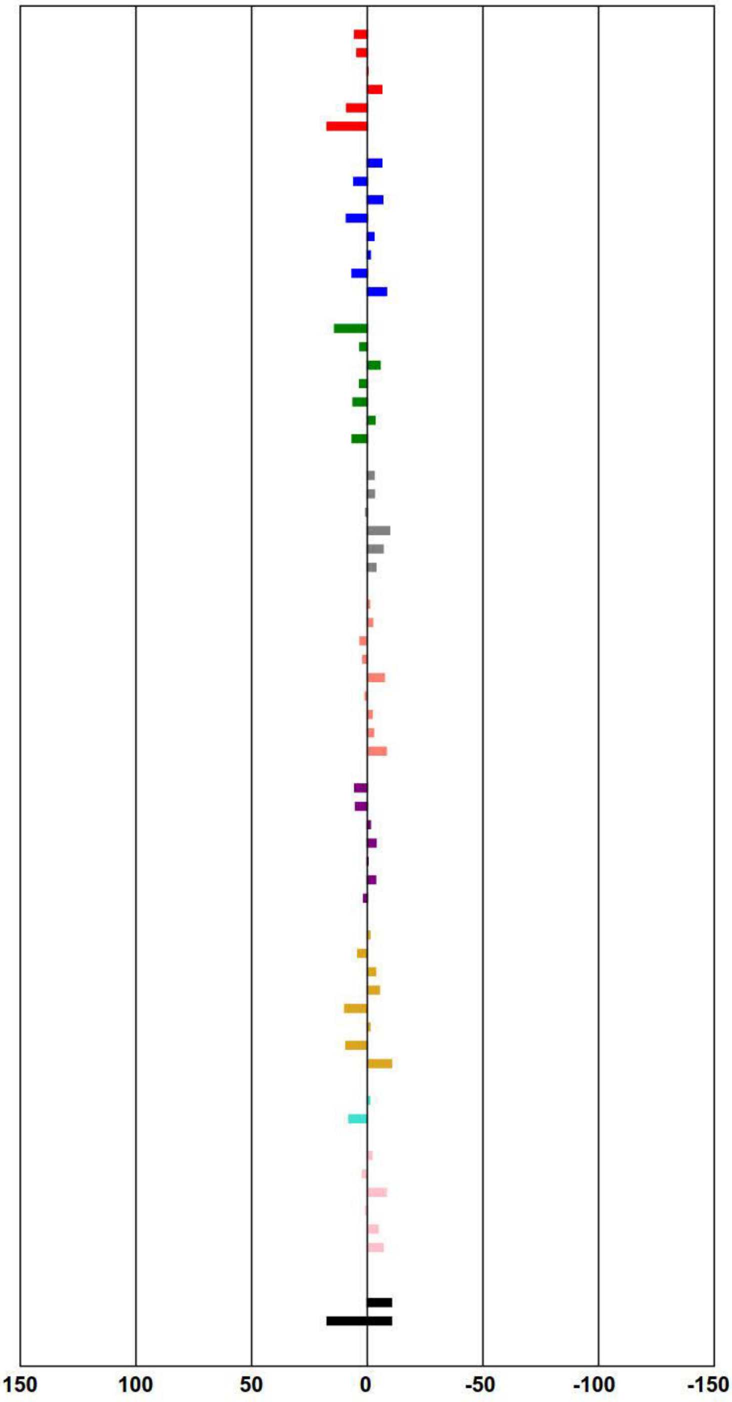

Figure S40. $^{1}$H NMR spectrum of 2**a** ($CDCl_3$)

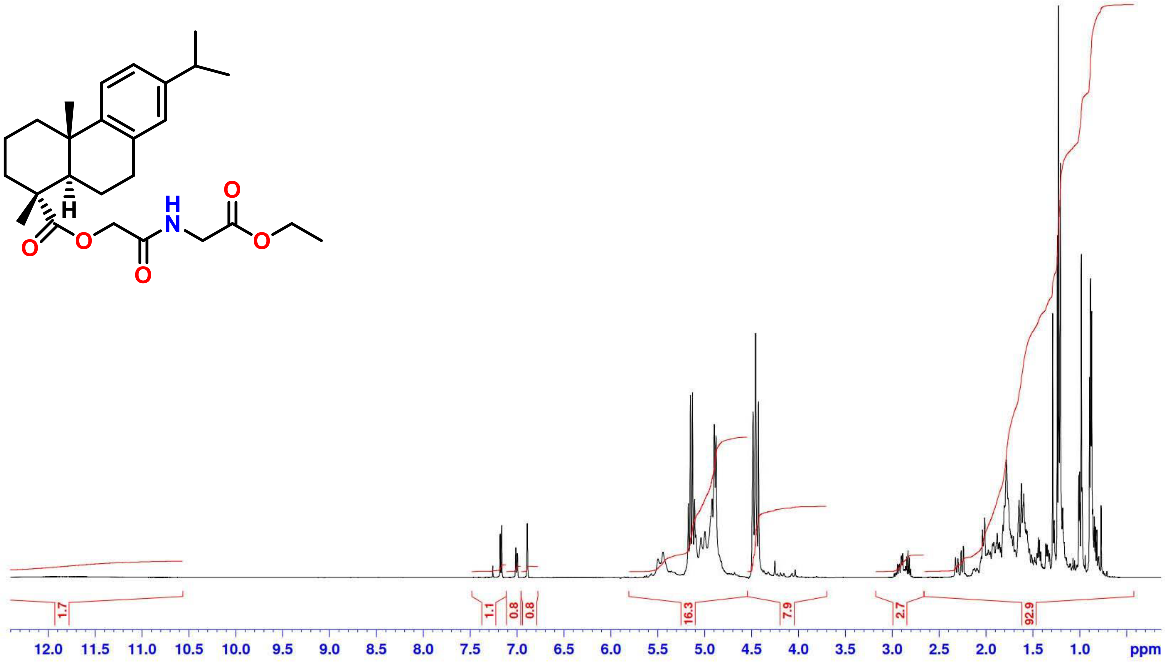


Figure S41. $^{13}$C-NMR spectrum of 2**a** ($CDCl_3$)

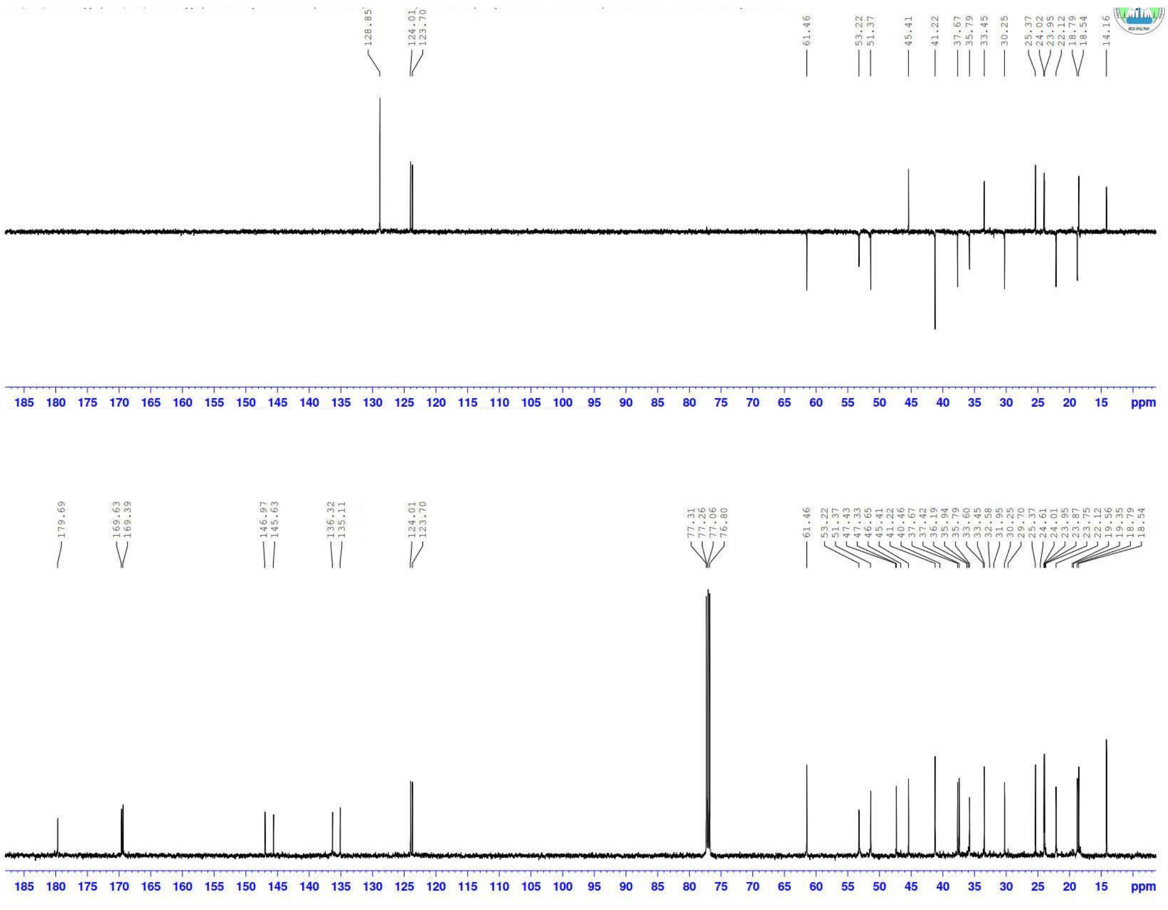

Figure S42. $^{1}$H NMR spectrum of 2b (CDCl$_3$+MeOD)

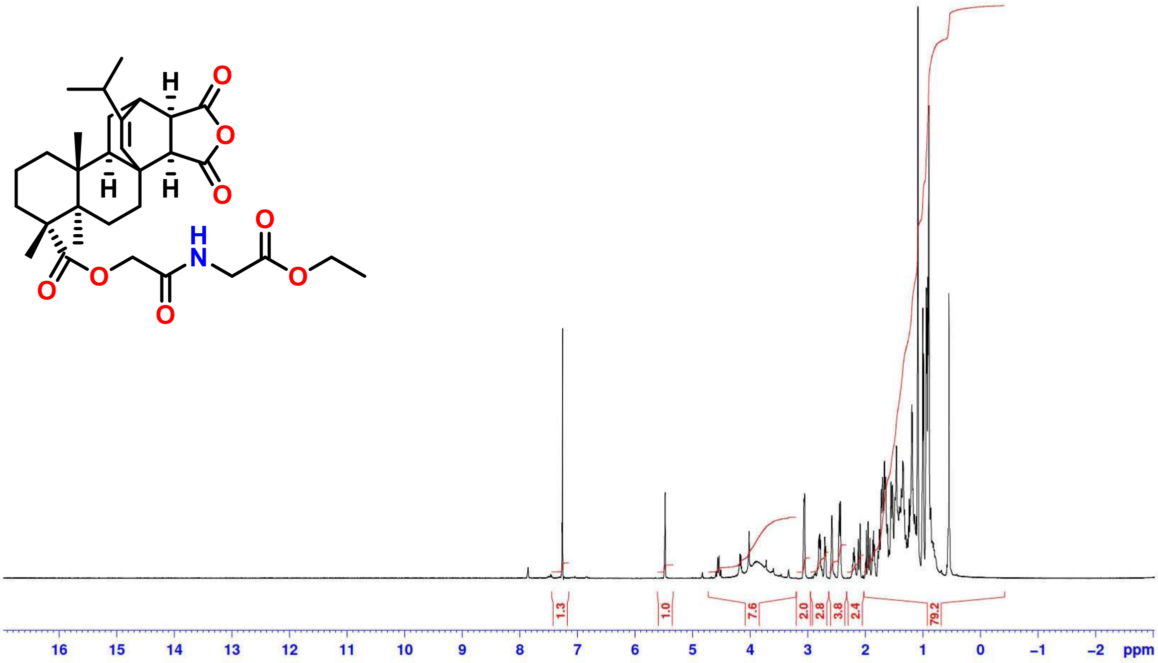

Figure S43. $^{13}$C-NMR spectrum of 2b (CDCl$_3$+MeOD)

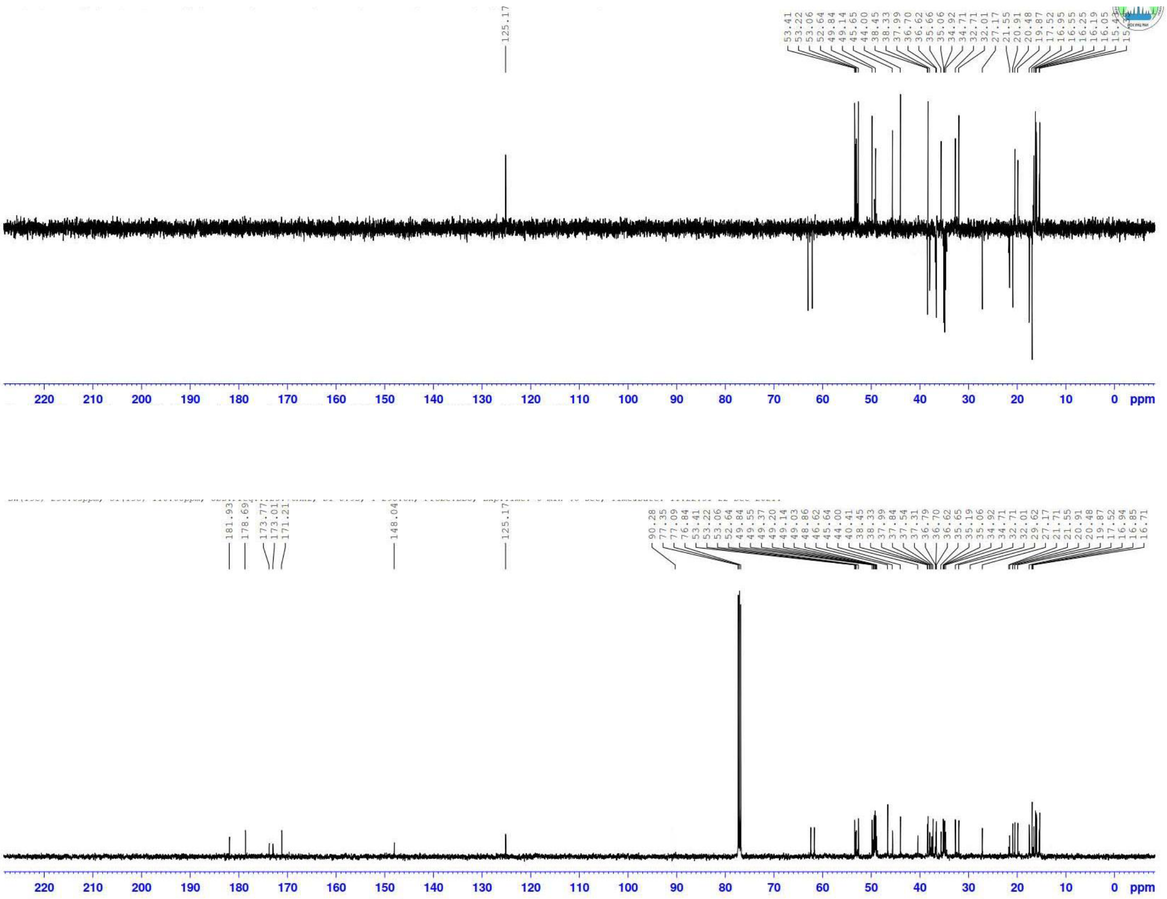

Figure S44. $^{1}$H NMR spectrum of 2d ($CDCl_3$)

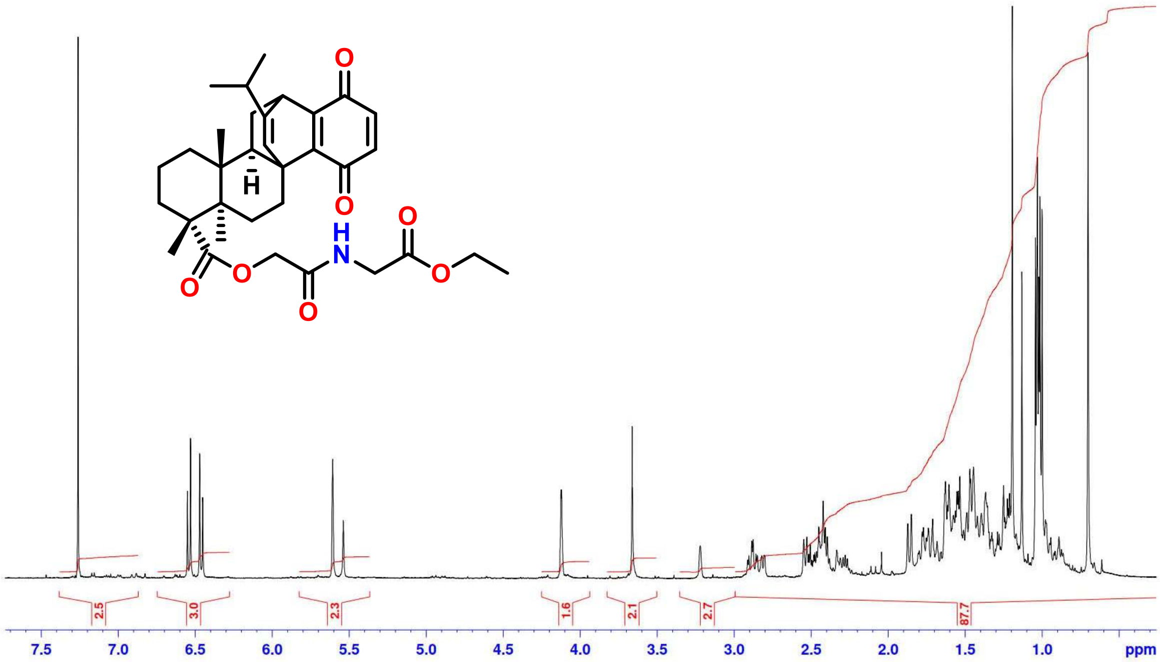


Figure S45. $^{13}$C-NMR spectrum of 2d ($CDCl_3$)

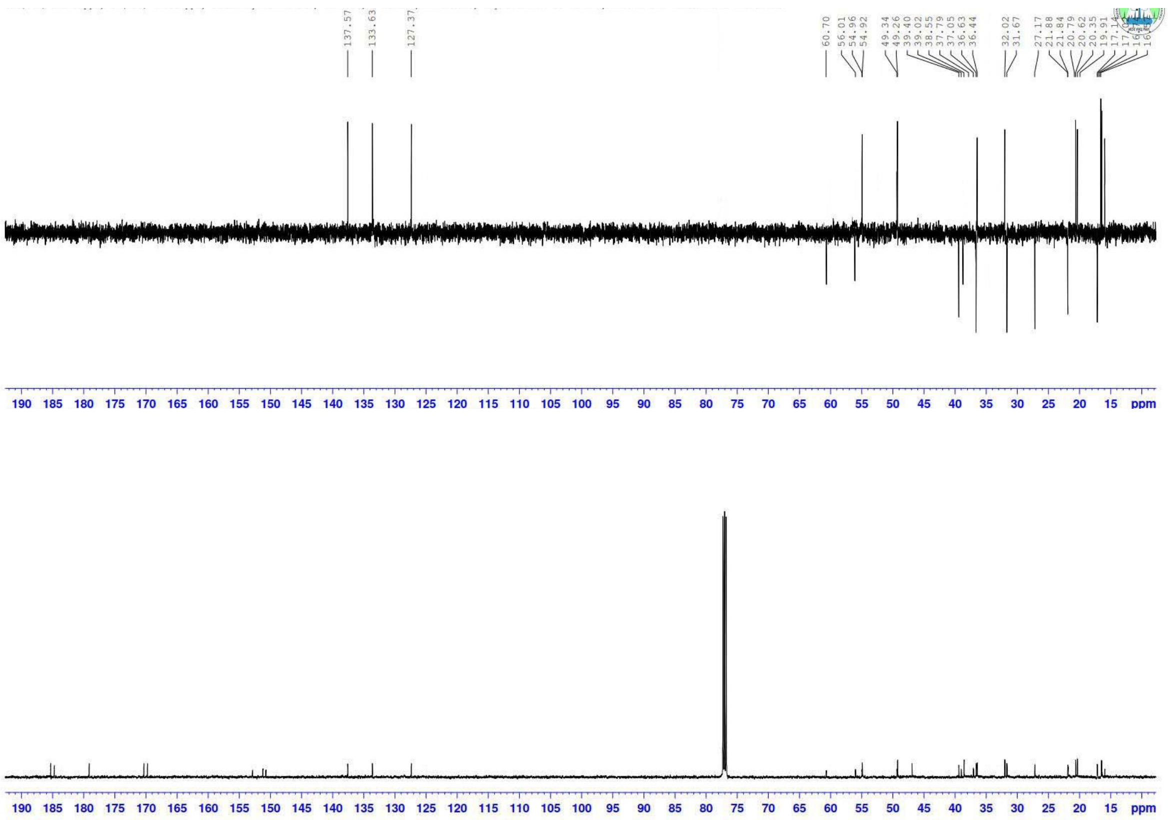

Figure S46. $^{1}$H NMR spectrum of 3**a** ($CDCl_3$)

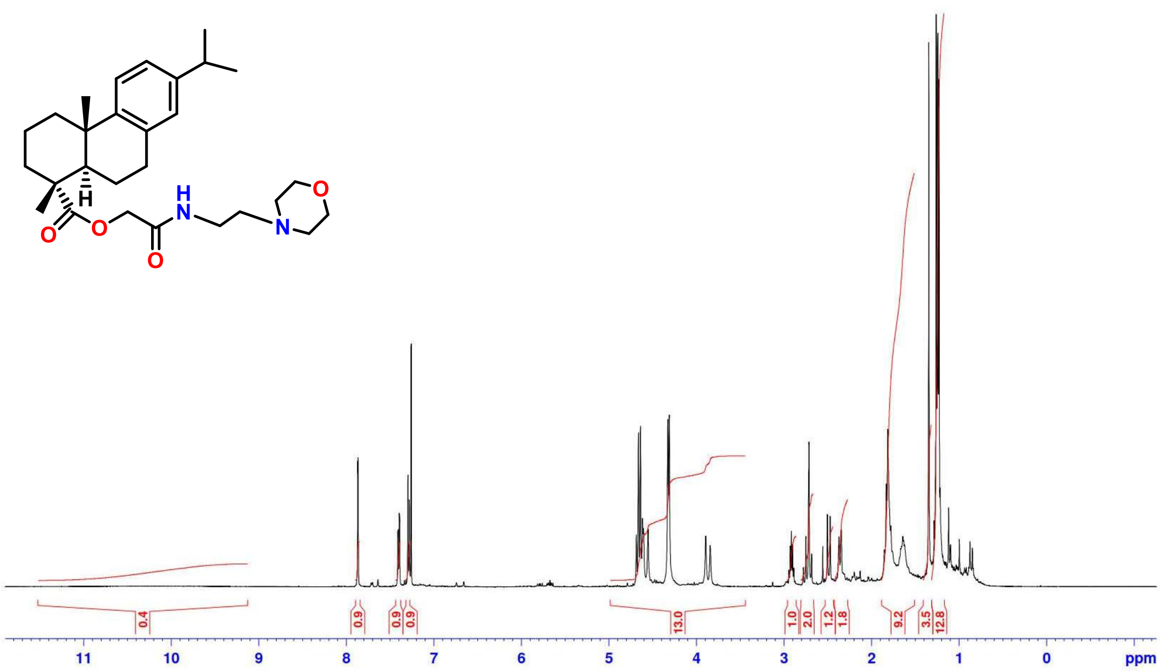

Figure S47. $^{13}$C-NMR spectrum of 3**a** ($CDCl_3$)

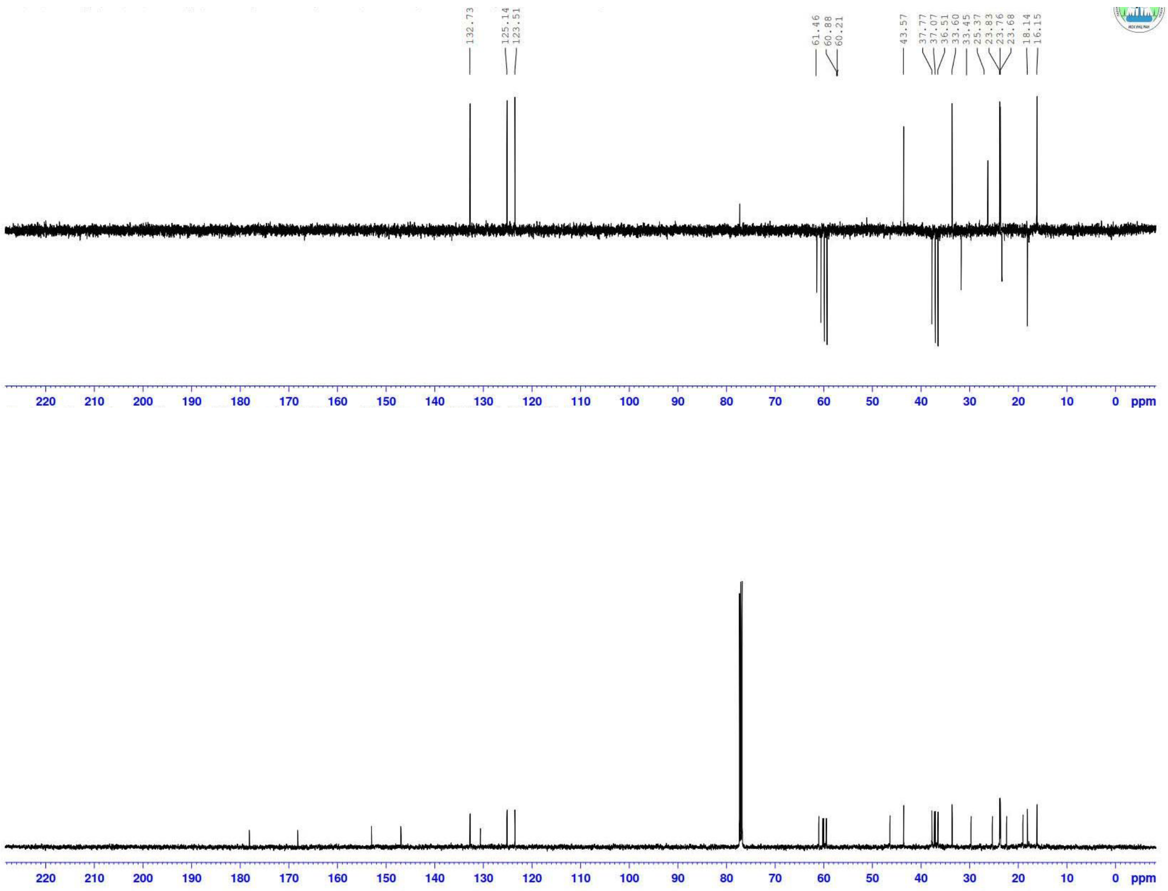

Figure S48. $^{1}$H NMR spectrum of 3b ($CDCl_3$+MeOD)

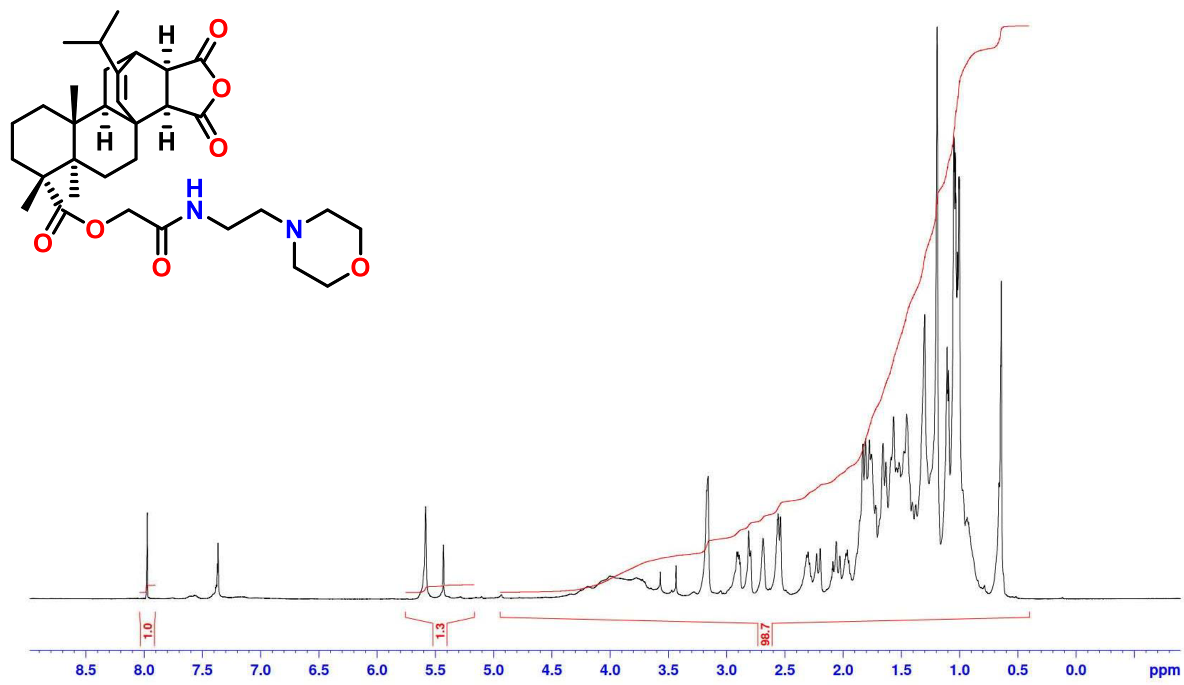


Figure S49. $^{13}$C-NMR spectrum of 3b ($CDCl_3$+MeOD)

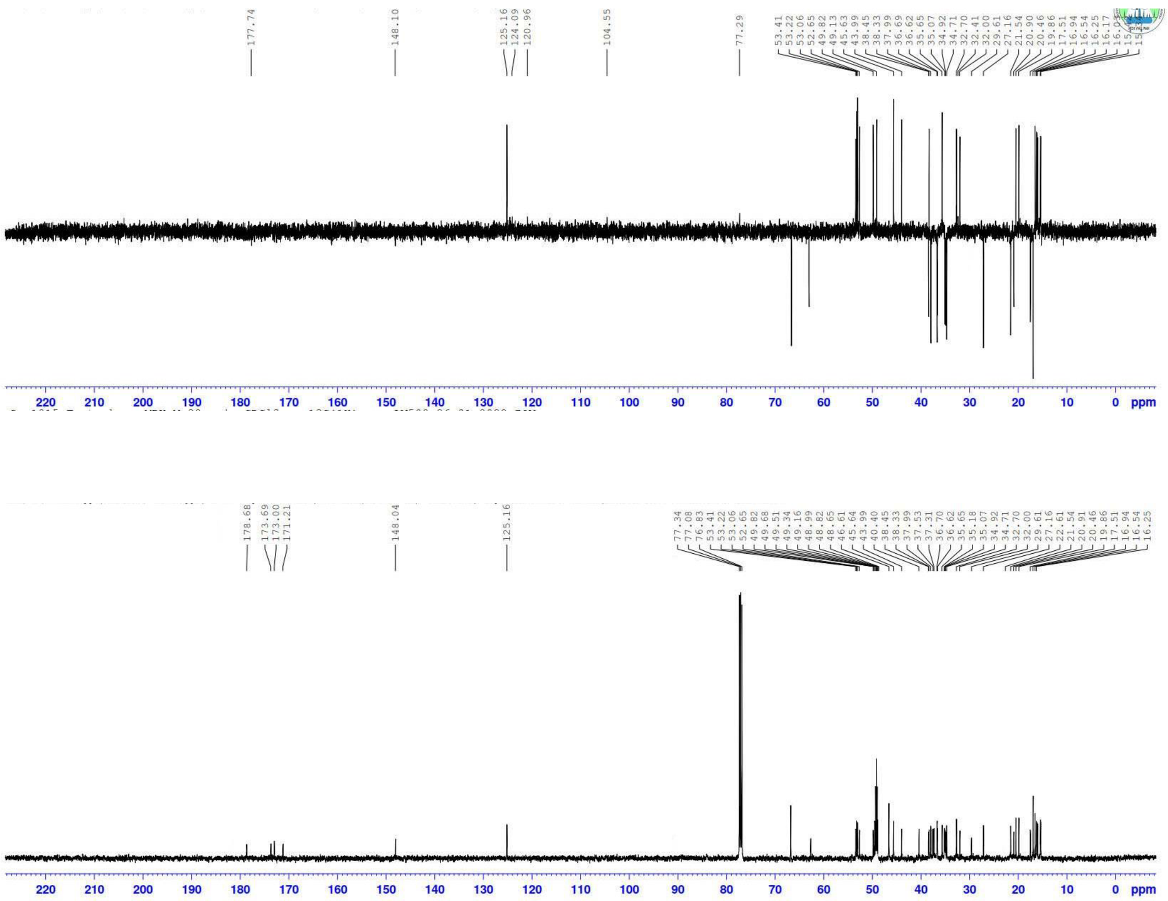

Figure S50. $^{1}$H NMR spectrum of 3c

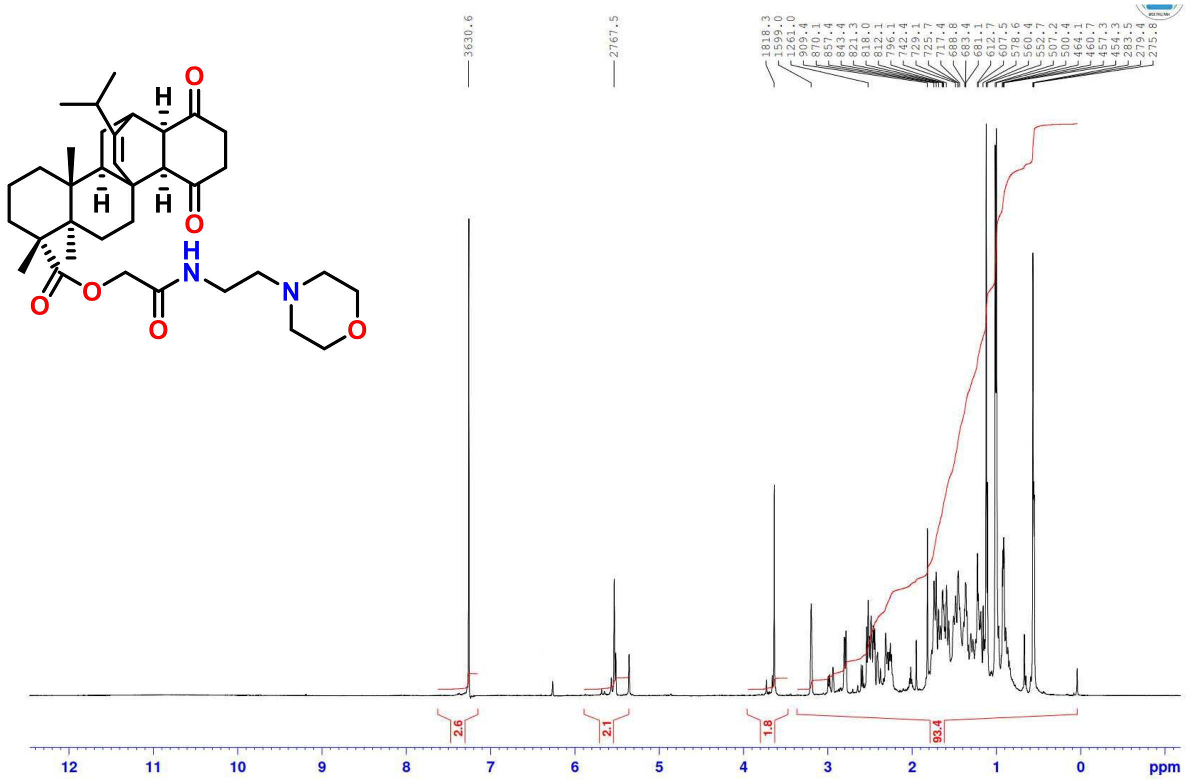

Figure S51. $^{13}$C-NMR spectrum of 3c

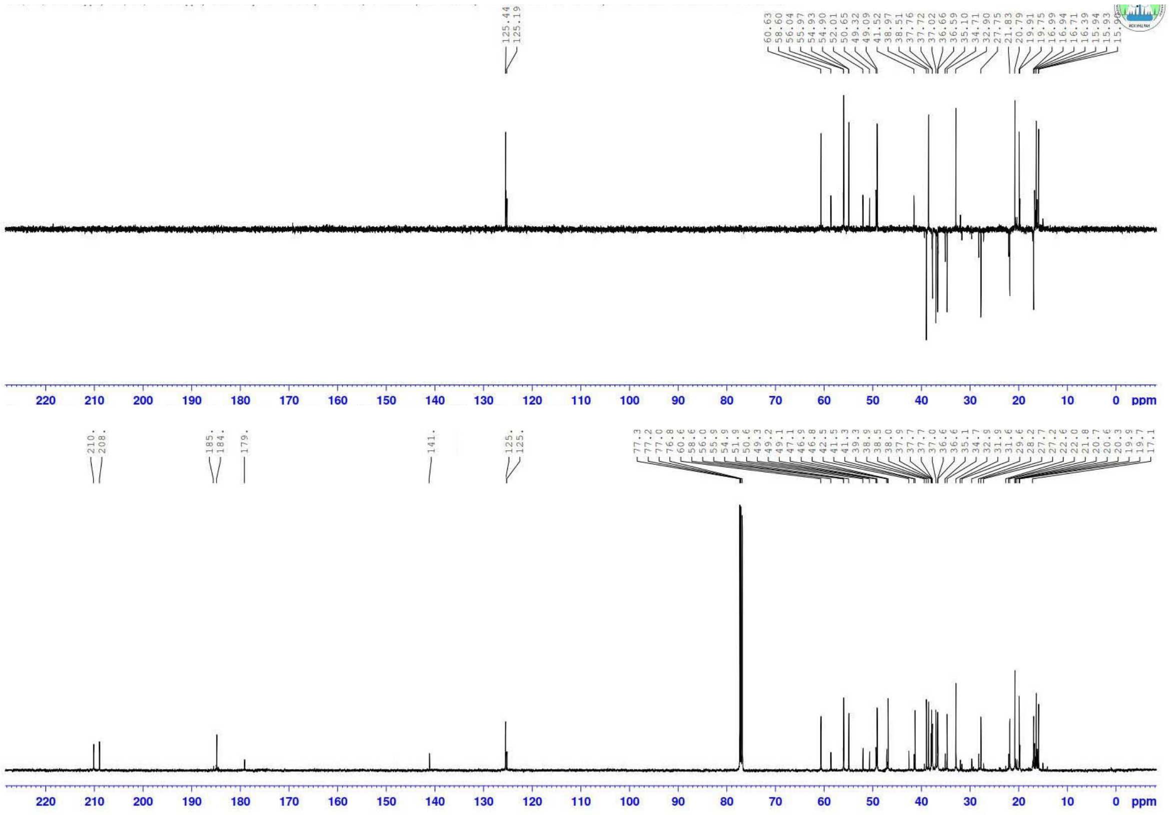

Figure S52. ¹H NMR spectrum of 3d

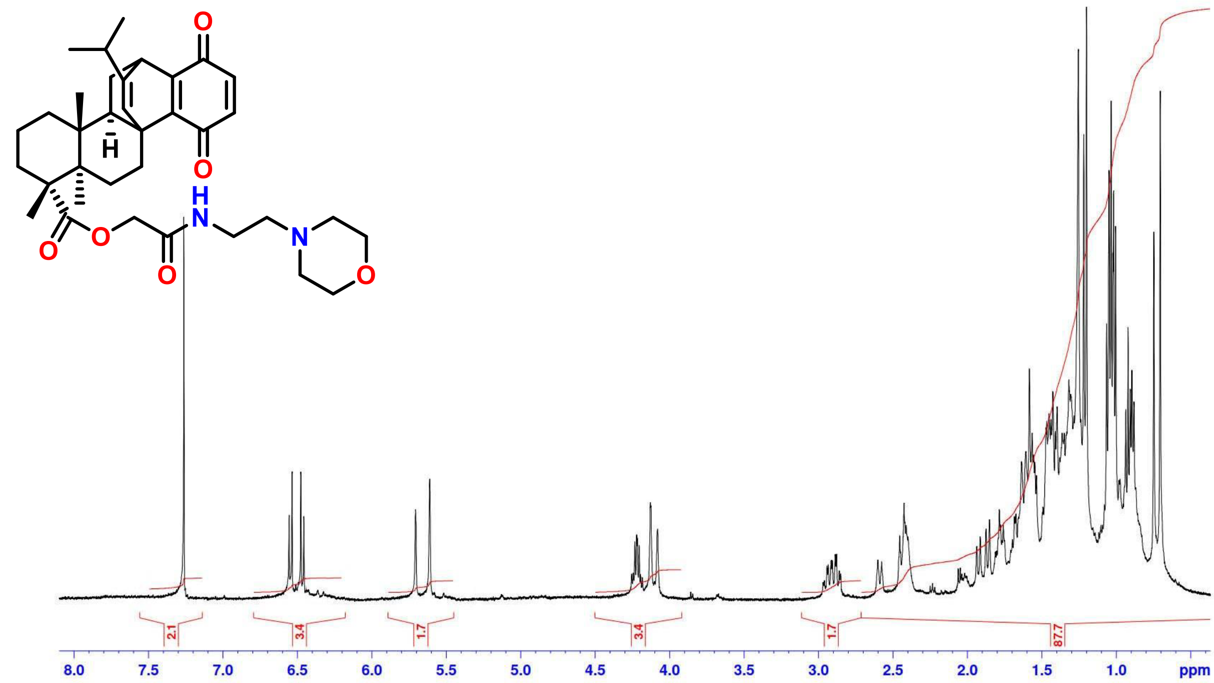


Figure S53. $^{13}$C-NMR spectrum of 3d

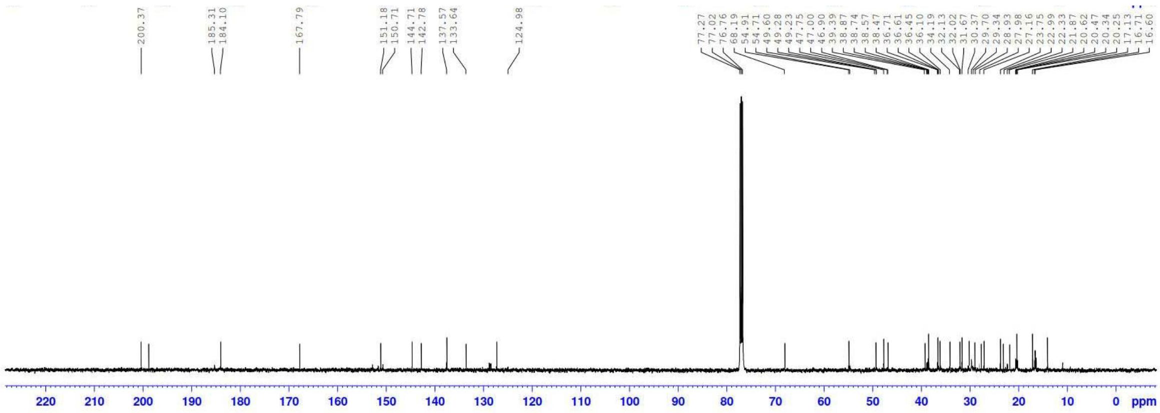

Figure S54. $^{1}$H NMR spectrum of 4**a** ($CDCl_3$)

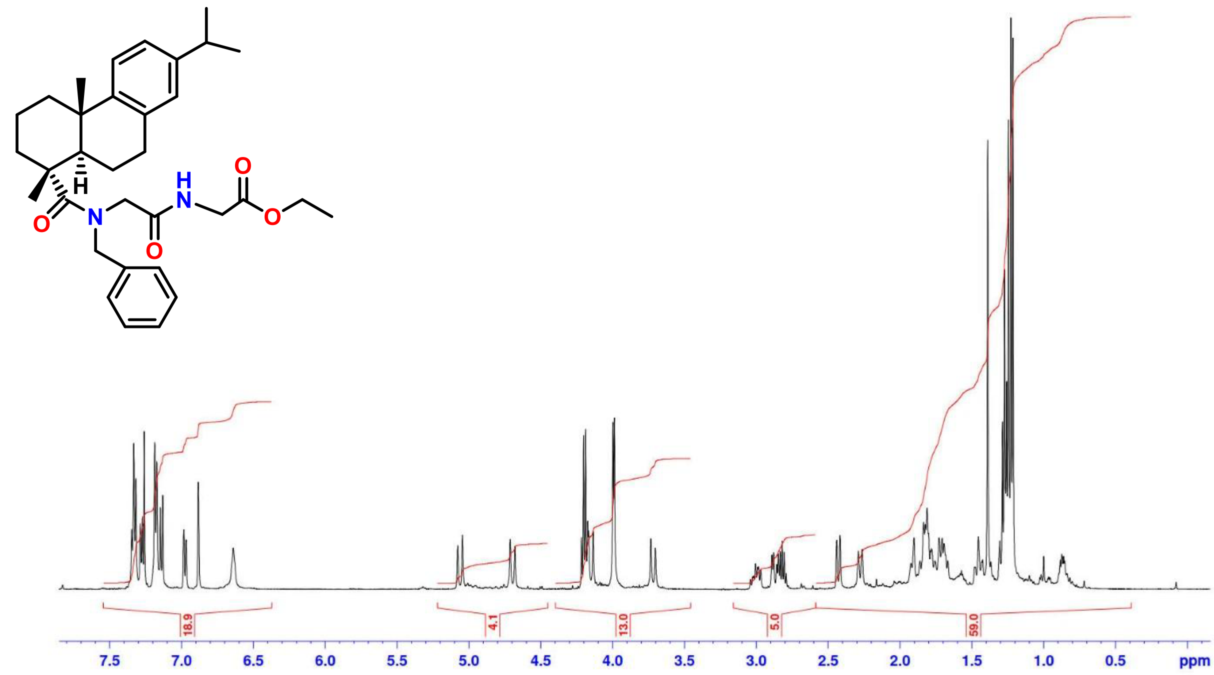


Figure S55. $^{13}$C-NMR spectrum of 4a ($CDCl_3$)

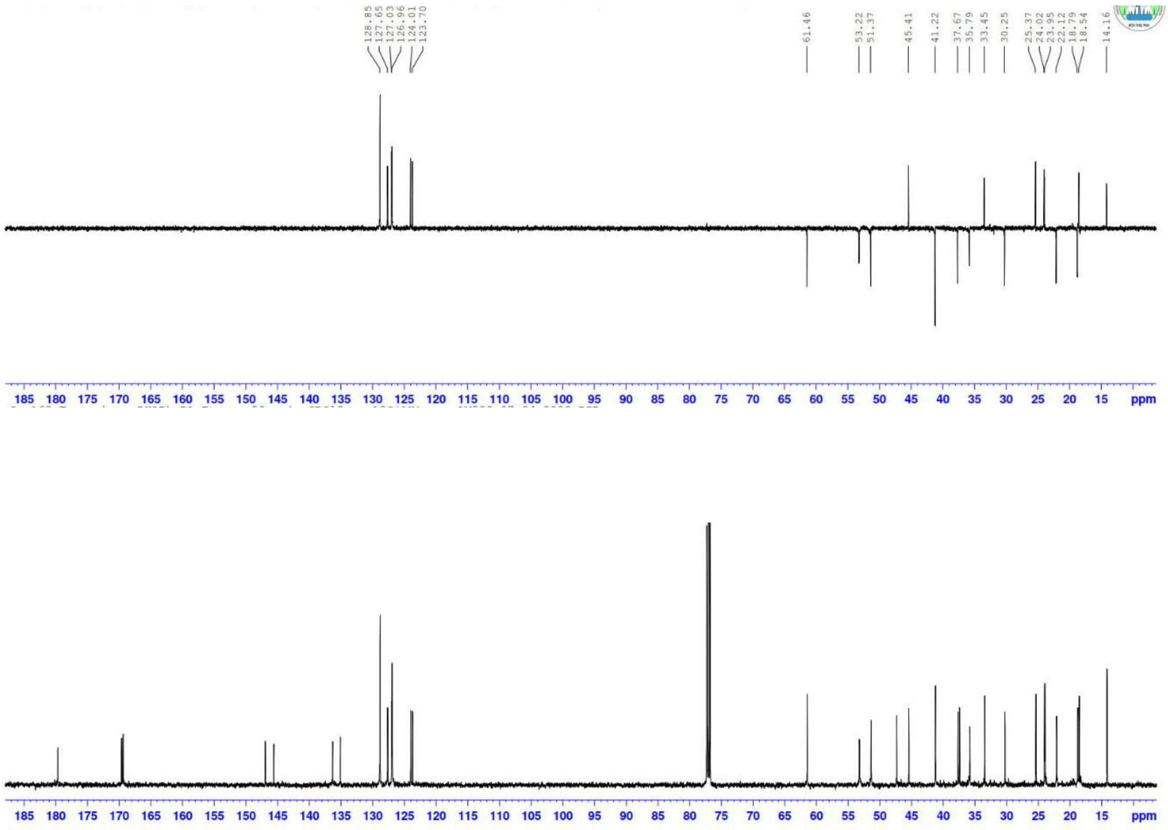

Figure S56. $^{1}$H NMR spectrum of 5**a** ($CDCl_3$)

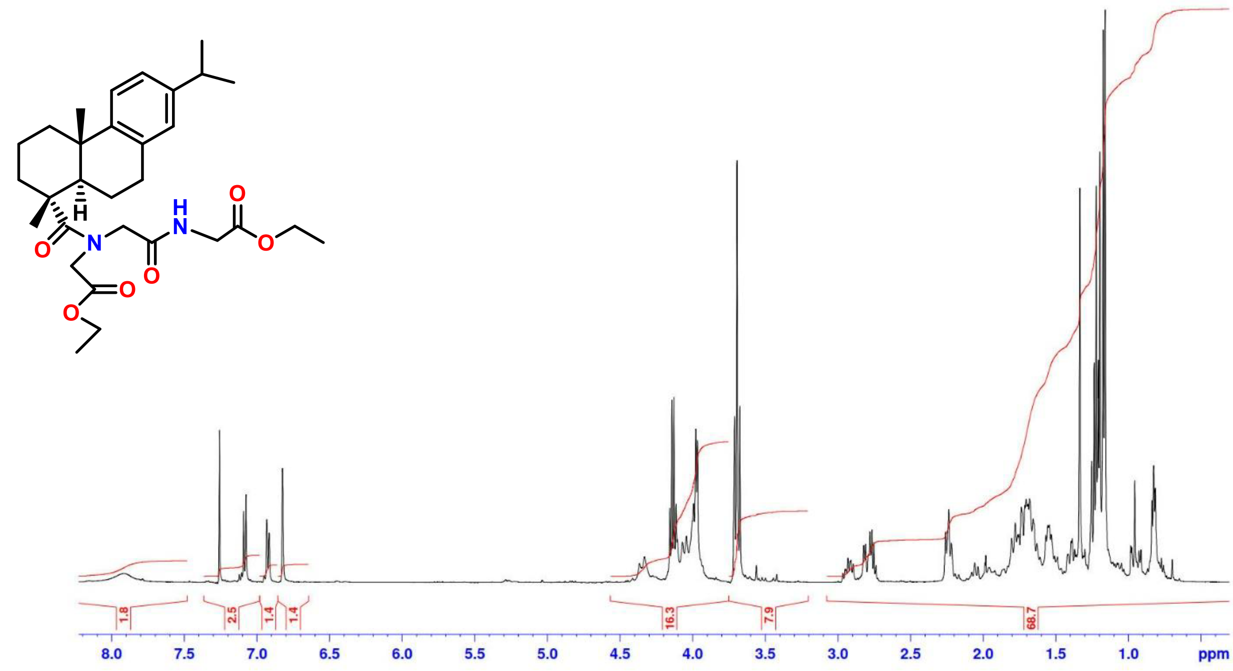


Figure S57. $^{13}$C-NMR spectrum of 5a ($CDCl_3$)

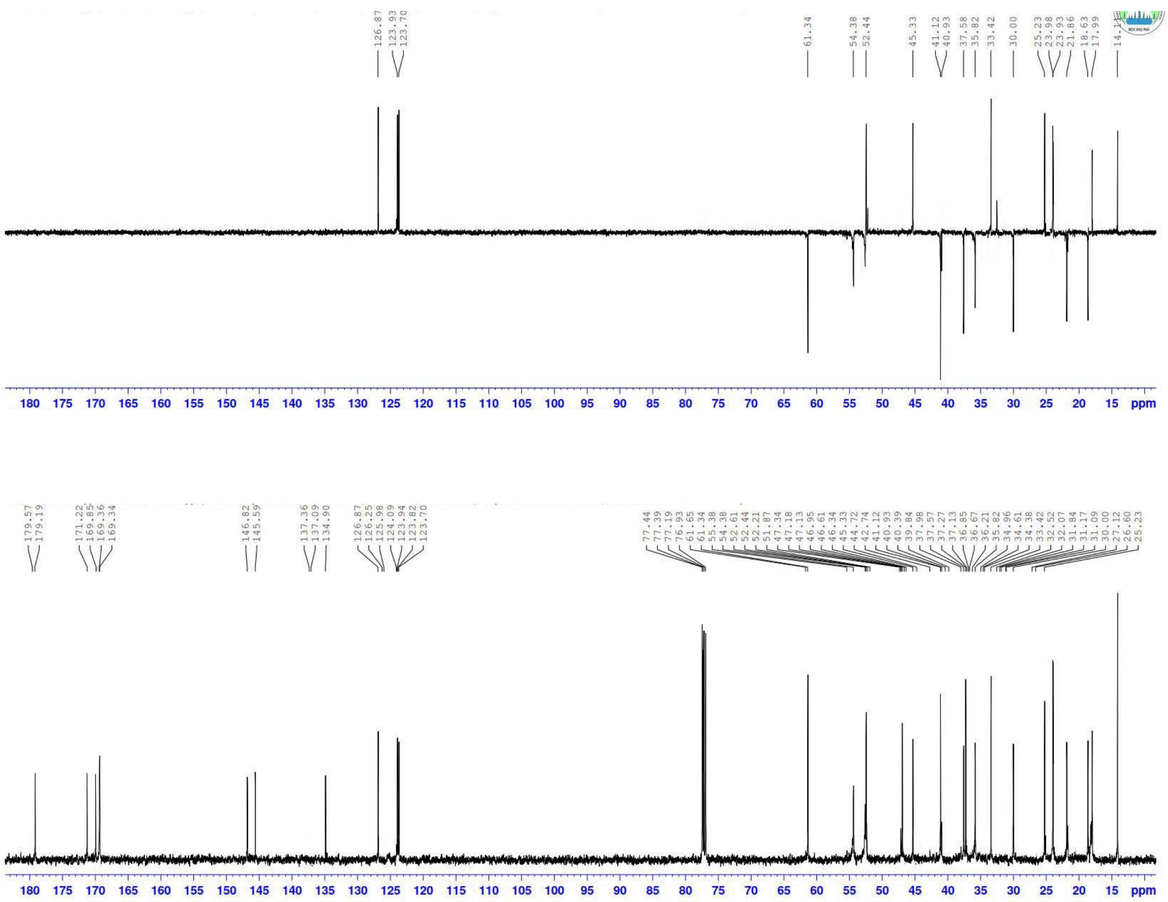

Figure S58. $^{1}$H NMR spectrum of 5b ($CDCl_3$)

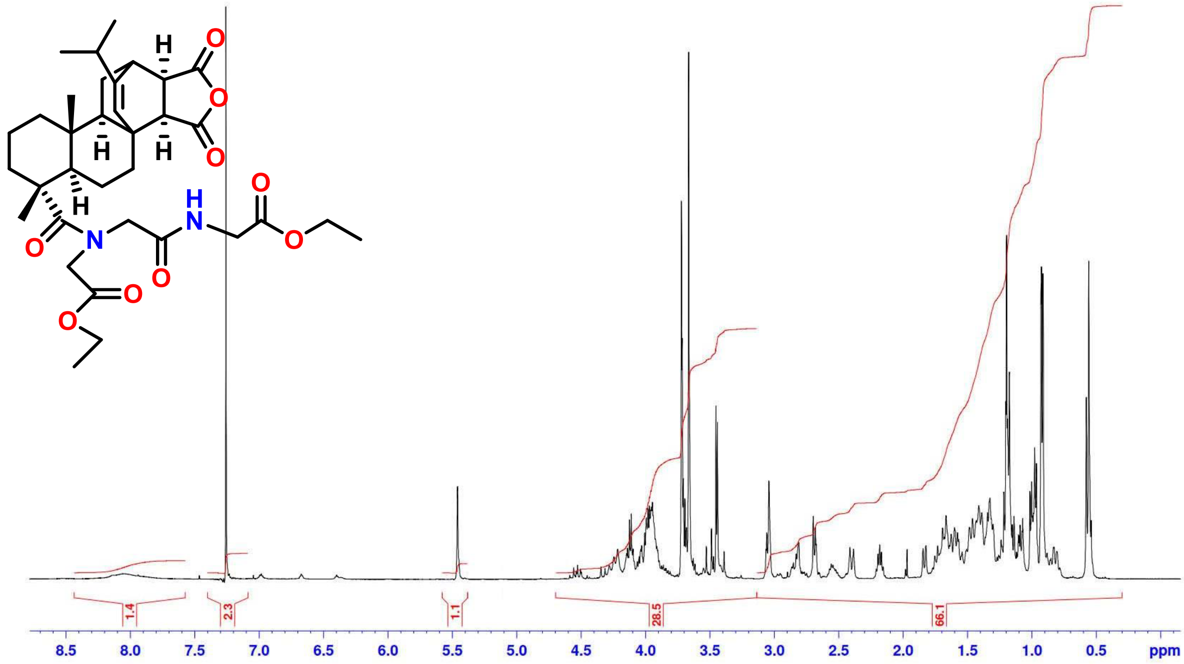


Figure S59. $^{13}$C-NMR spectrum of 5b ($CDCl_3$)

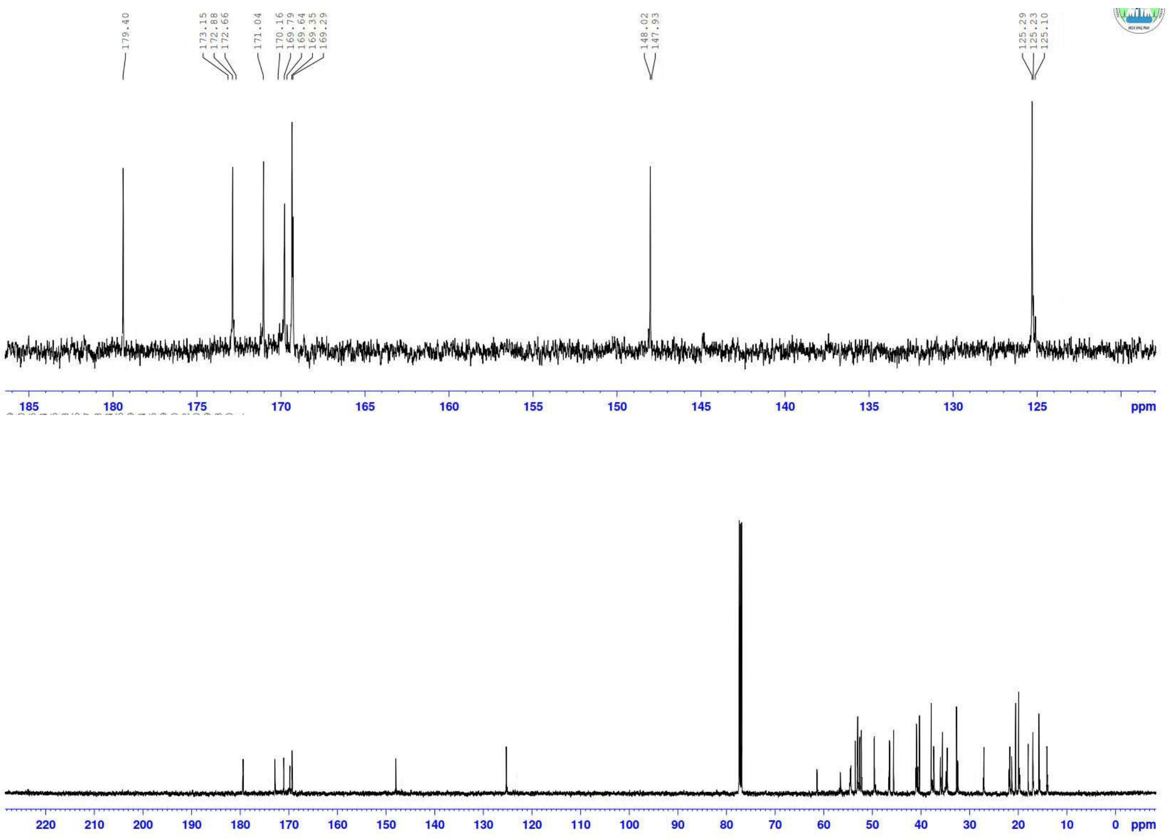

Figure S60. $^{1}H$ NMR spectrum of 5c ($CDCl_3$)

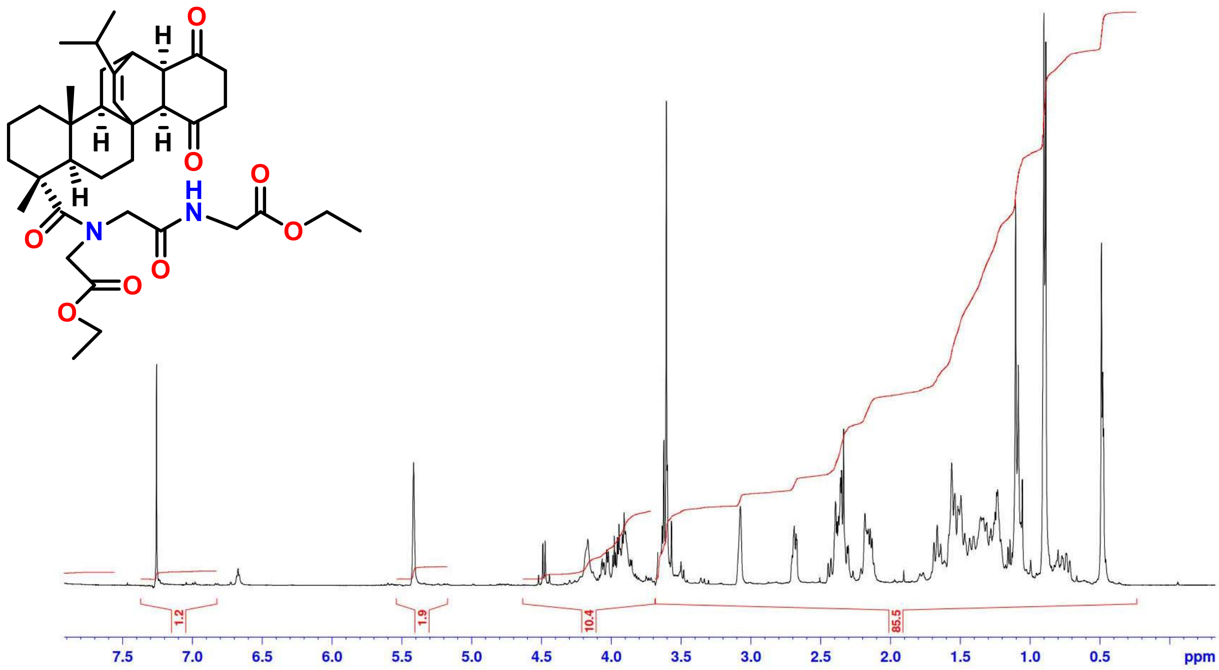


Figure S61. $^{13}C$-NMR spectrum of 5c ($CDCl_3$)

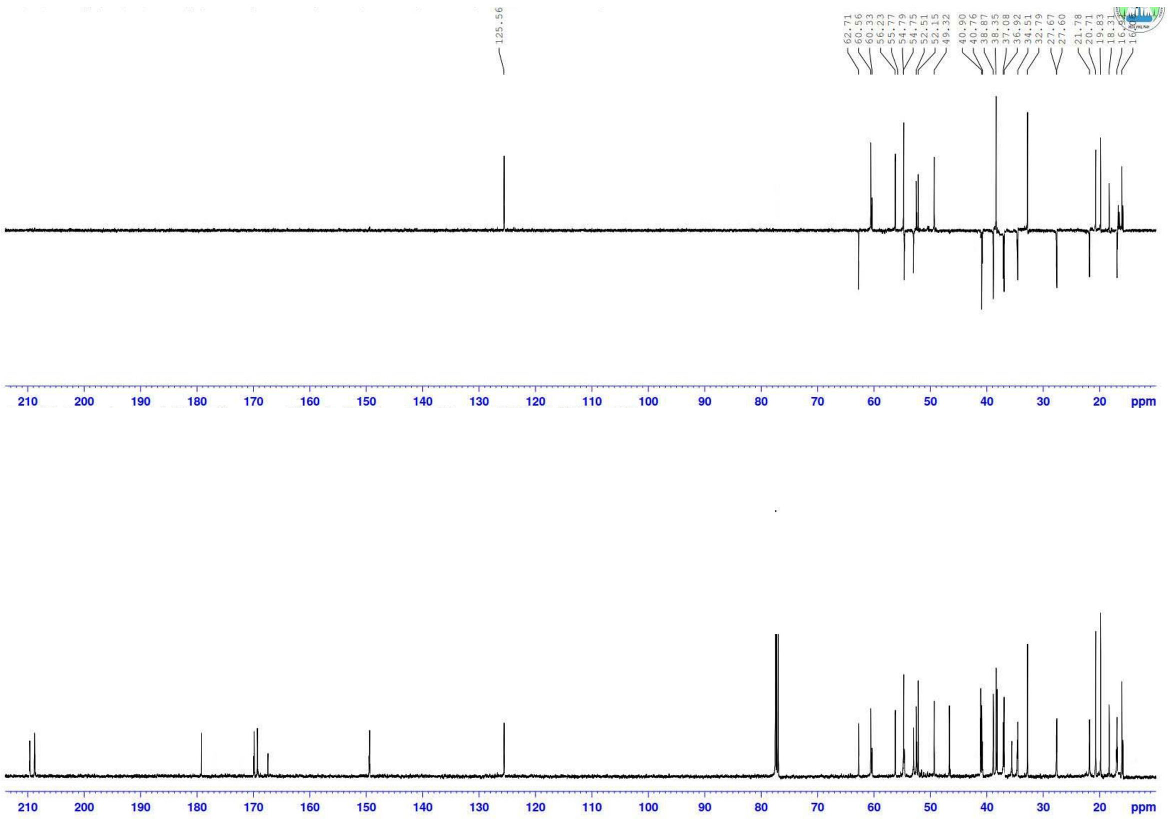

Figure S61. $^{1}$H NMR spectrum of 6**a** ($CDCl_3$)

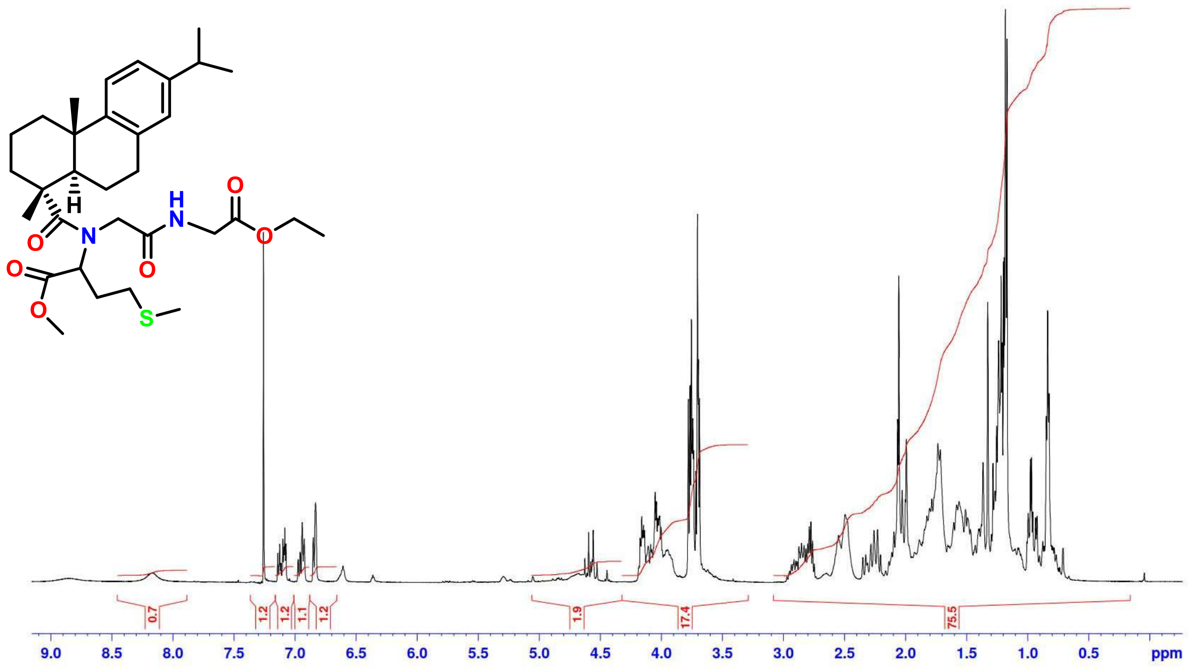


Figure S63. $^{13}$C-NMR spectrum of 6a ($CDCl_3$)

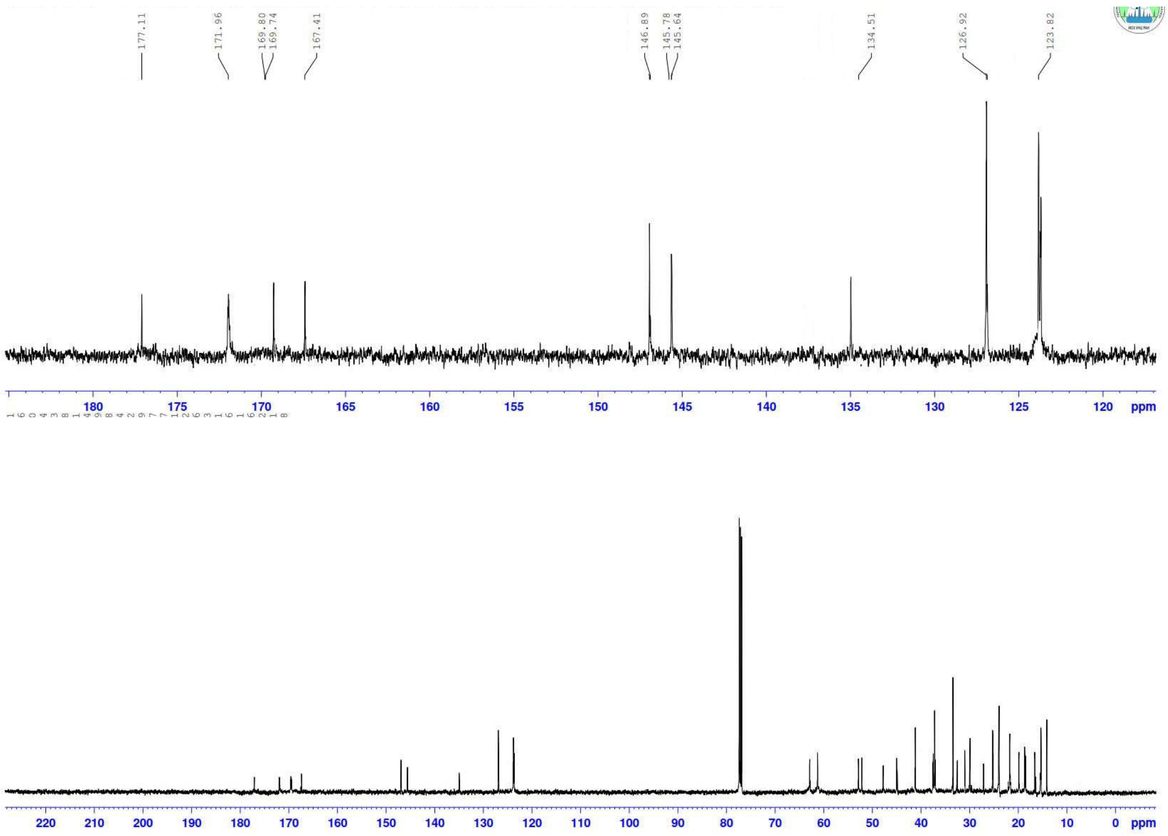

Figure S64. $^{1}$H NMR spectrum of 6c ($CDCl_3$)

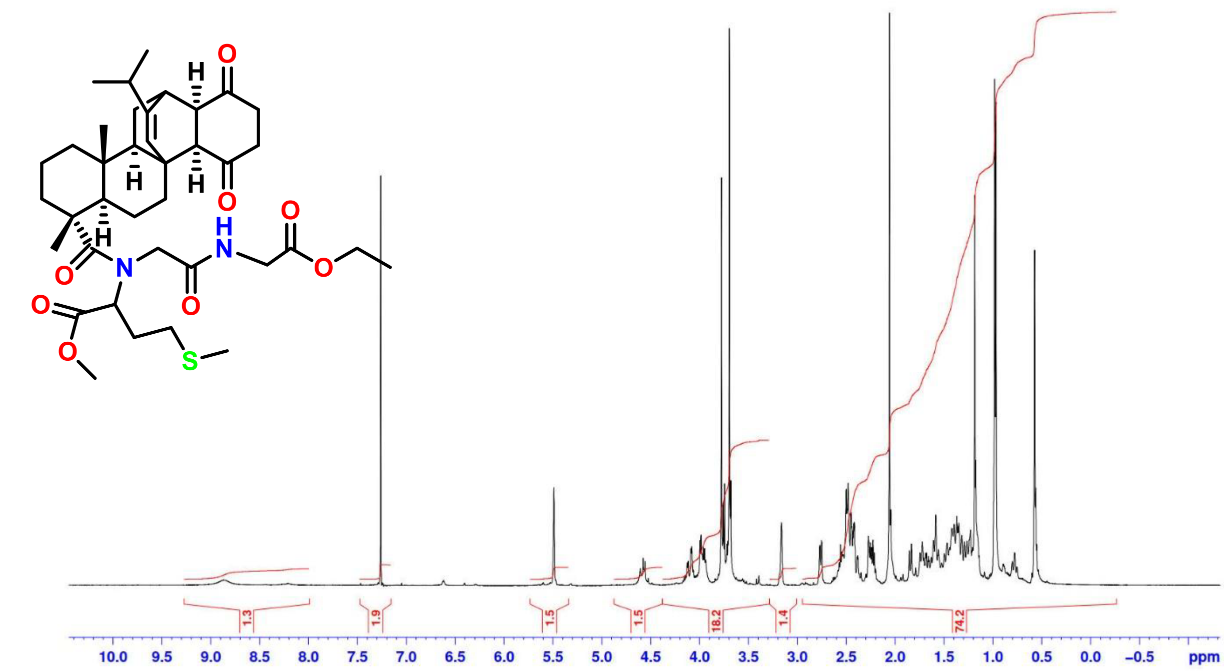


Figure S65. $^{13}$C-NMR spectrum of 6c ($CDCl_3$)

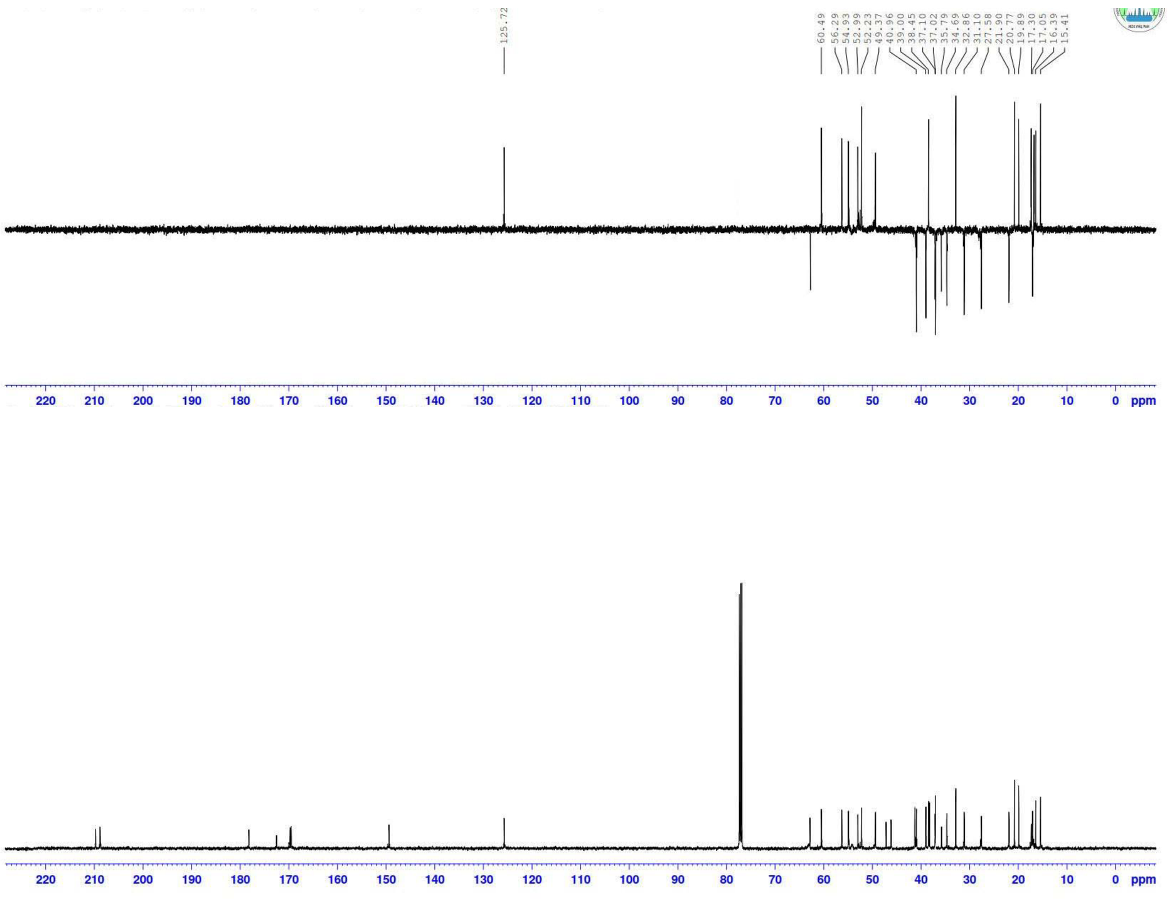

Figure S66. $^{1}$H NMR spectrum of 7b ($CDCl_3$)

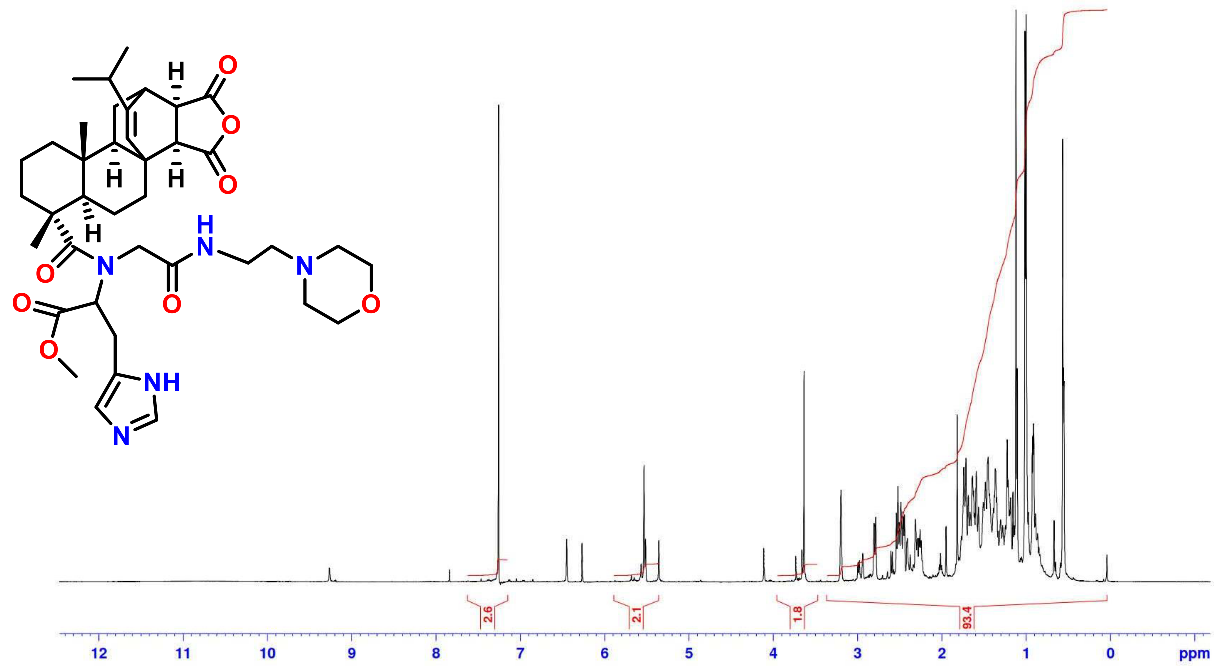


Figure S67. $^{13}$C-NMR spectrum of 7b ($CDCl_3$)

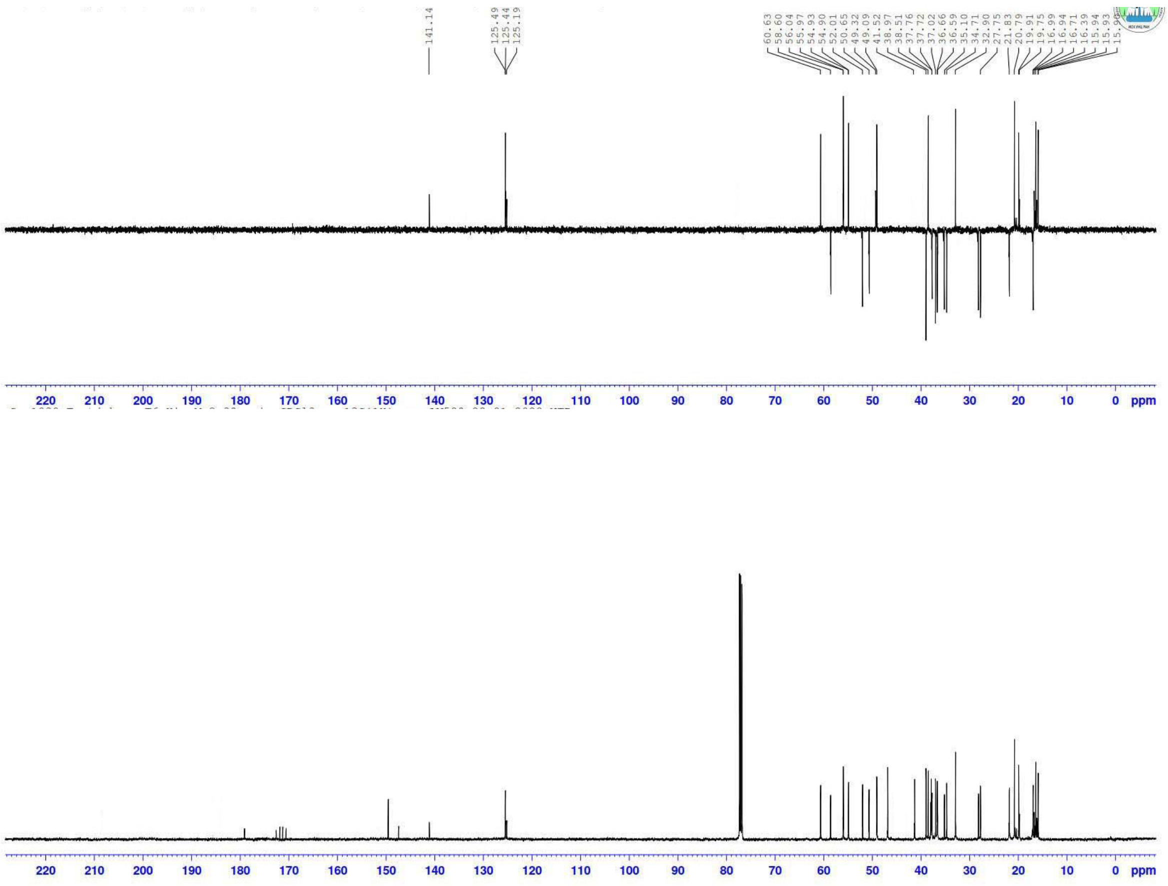

Figure S68. $^{1}$H NMR spectrum of 7c ($CDCl_3$)

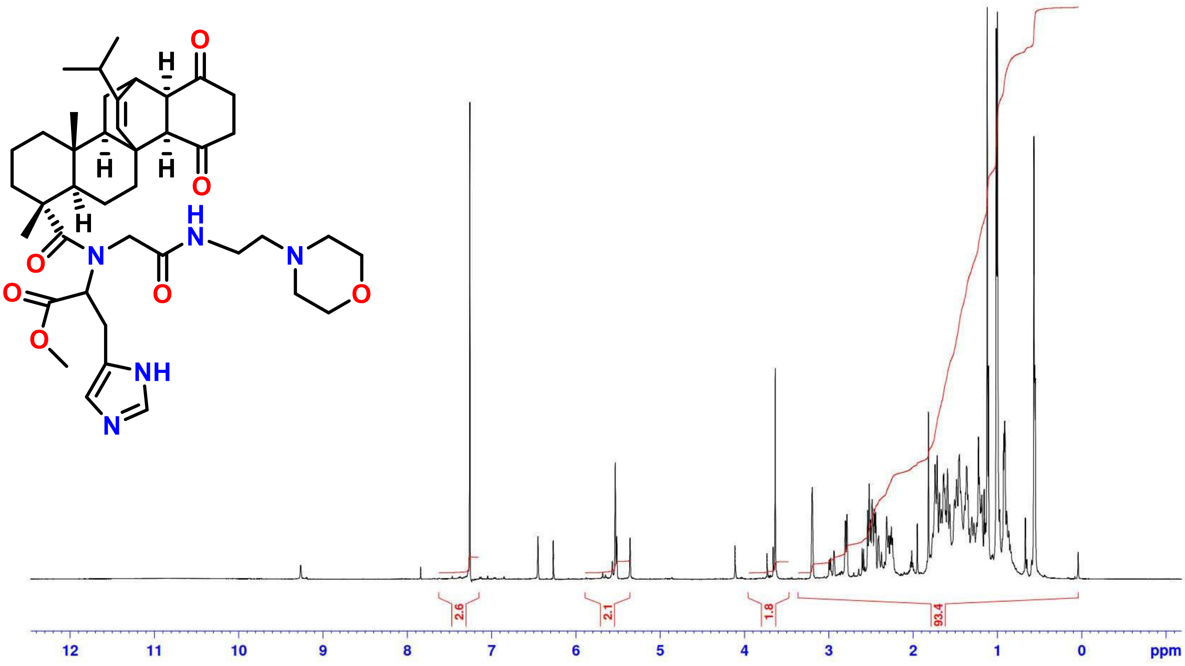


Figure S69. $^{13}$C-NMR spectrum of 7c ($CDCl_3$)

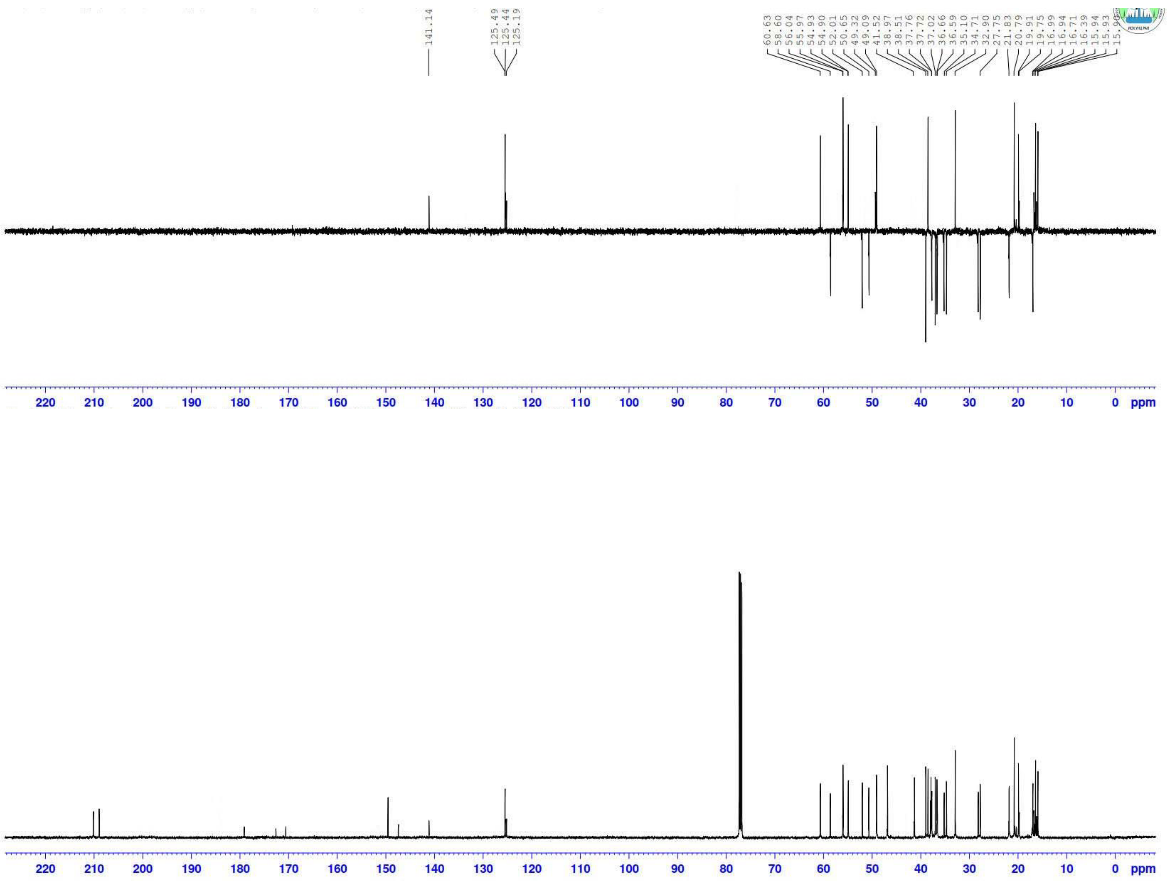

Figure S70. $^{1}$H NMR spectrum of 8b ($CDCl_3$)

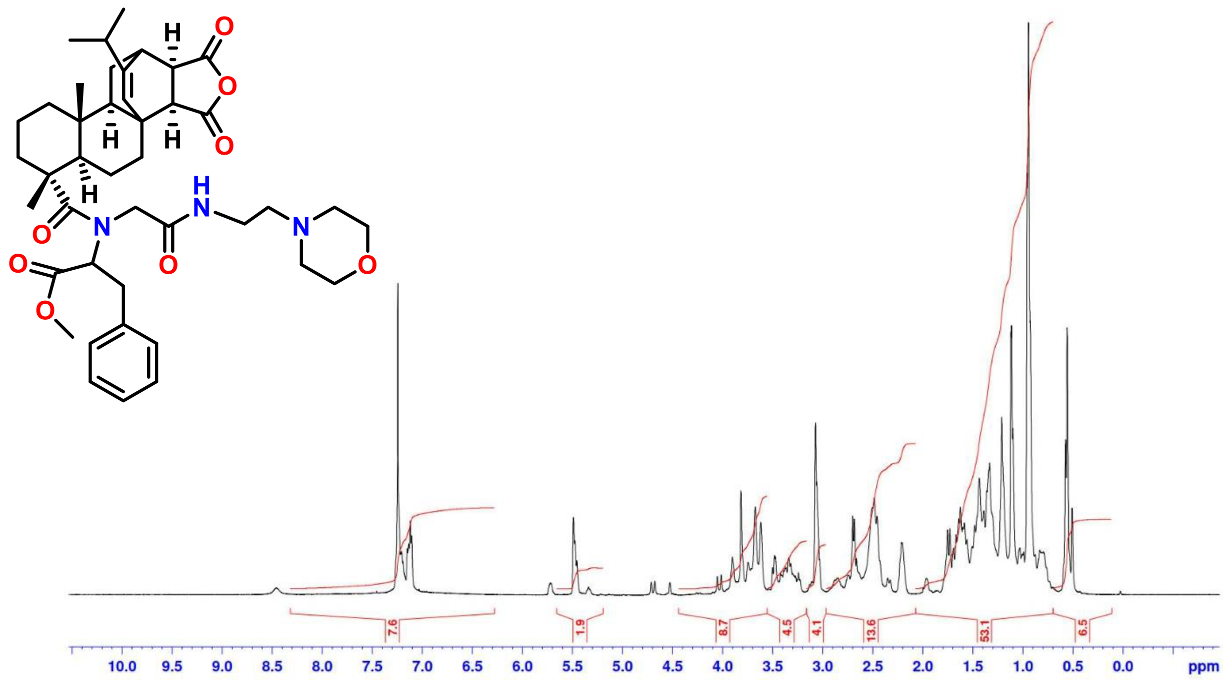


Figure S71. $^{13}$C-NMR spectrum of 8b ($CDCl_3$)

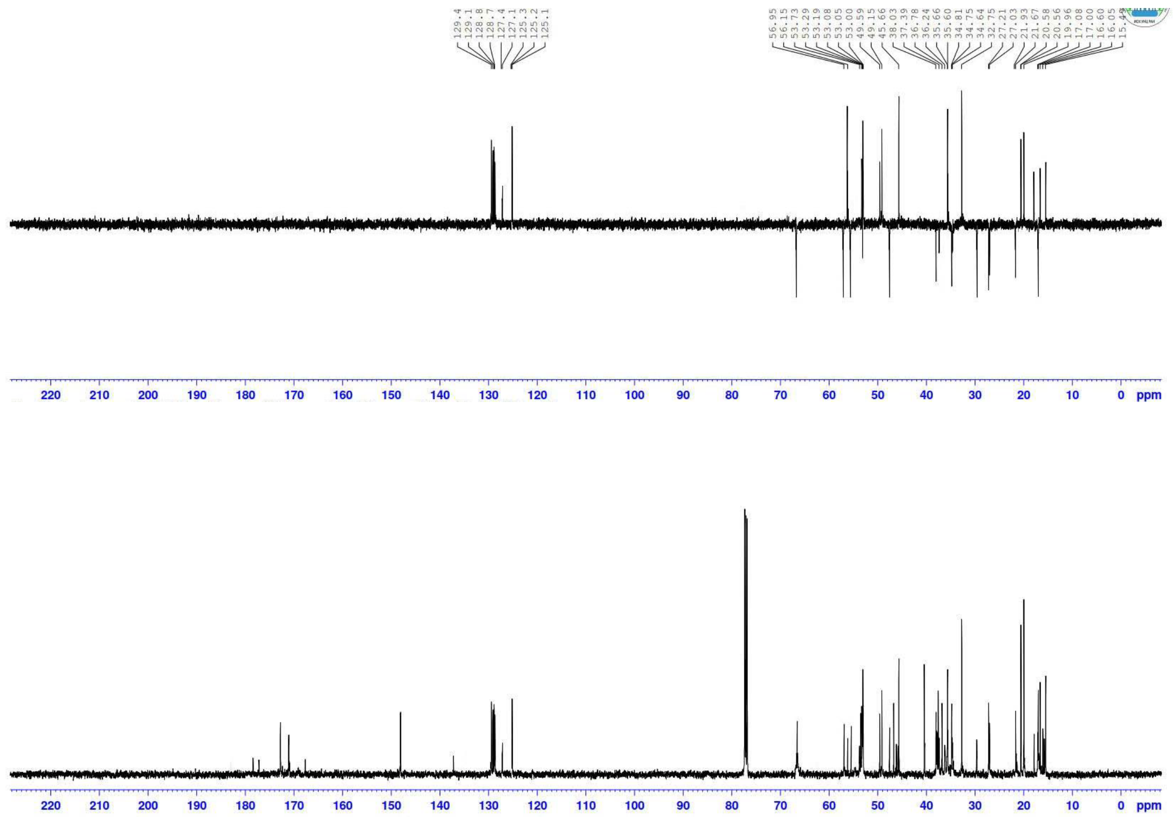

Figure S72. $^{1}$H NMR spectrum of 9d ($CDCl_3$)

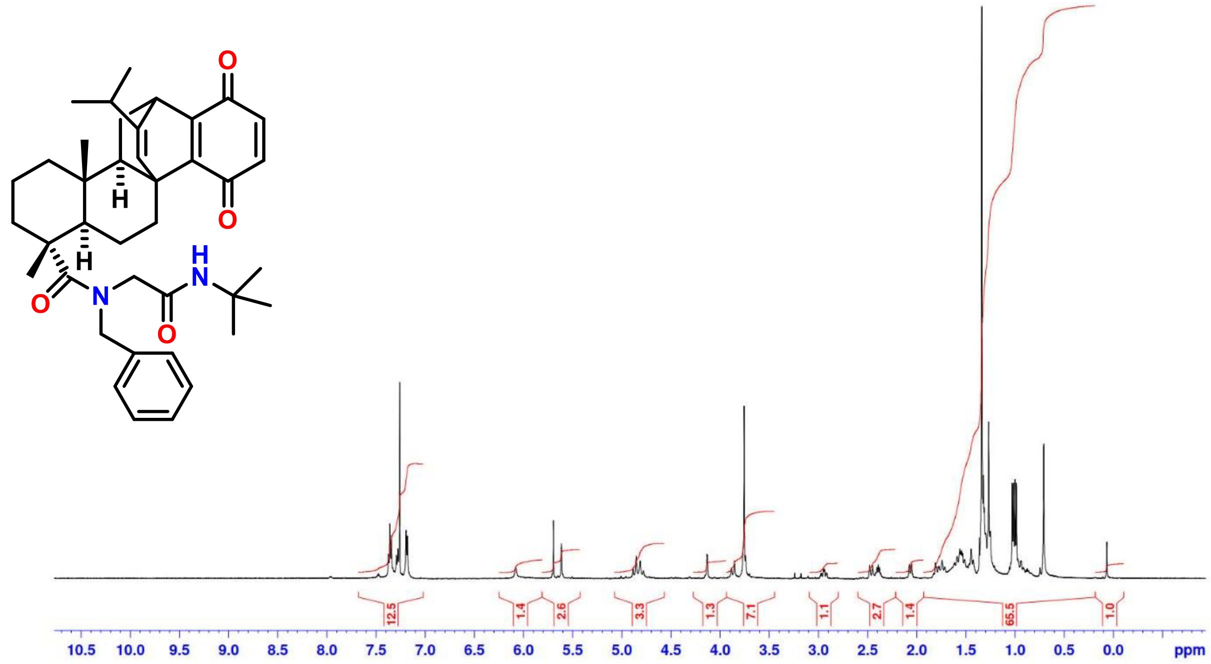


Figure S73. $^{13}$C-NMR spectrum of 9d ($CDCl_3$)

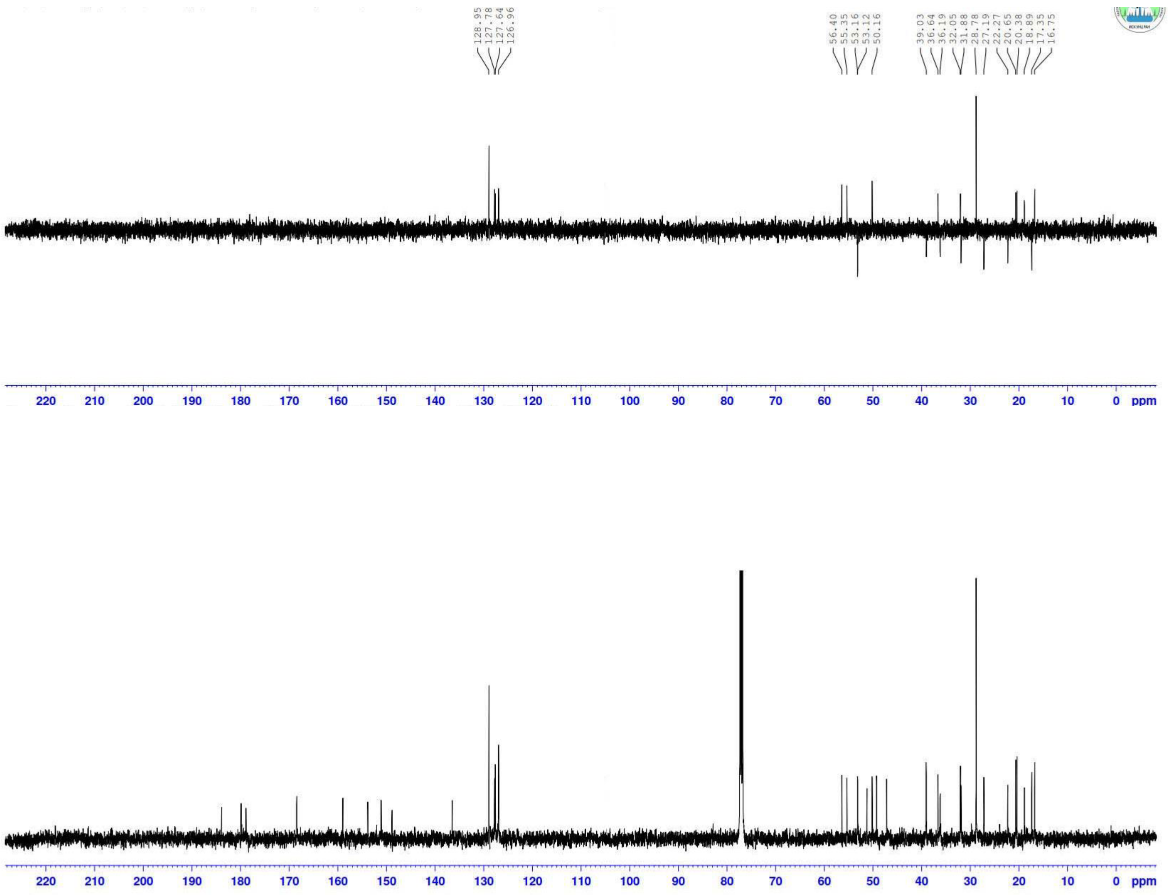

Figure S74. $^{1}$H NMR spectrum of 10d ($CDCl_3$)

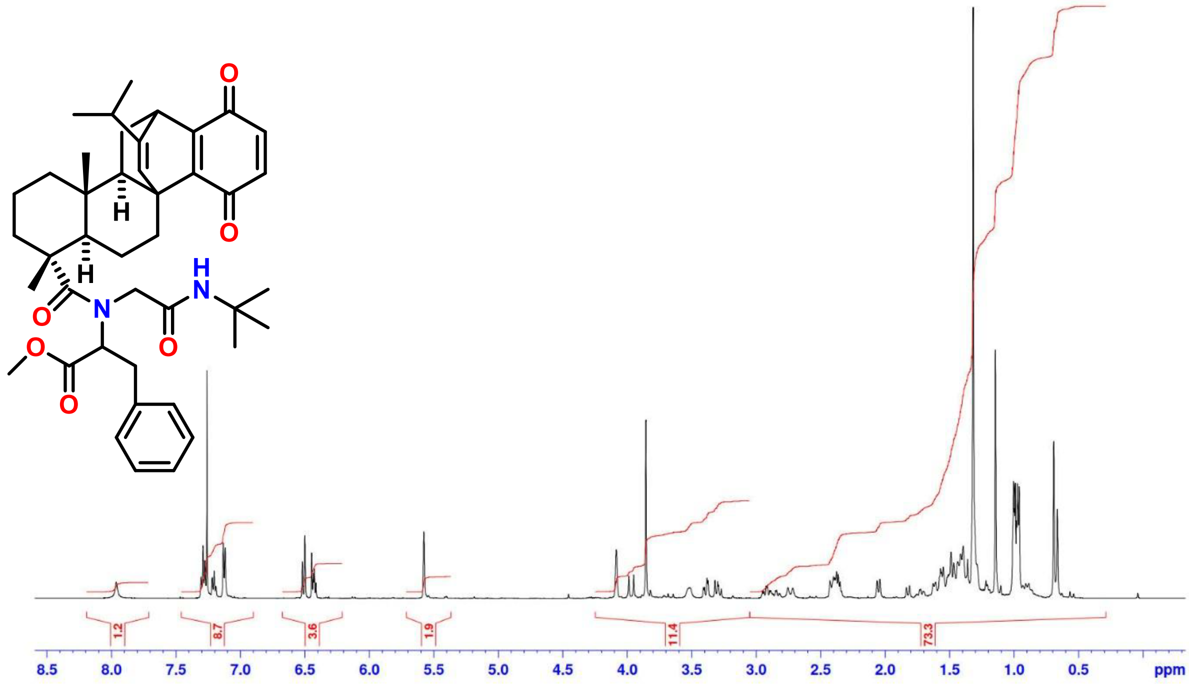


Figure S75. $^{13}$C-NMR spectrum of 10d ($CDCl_3$)

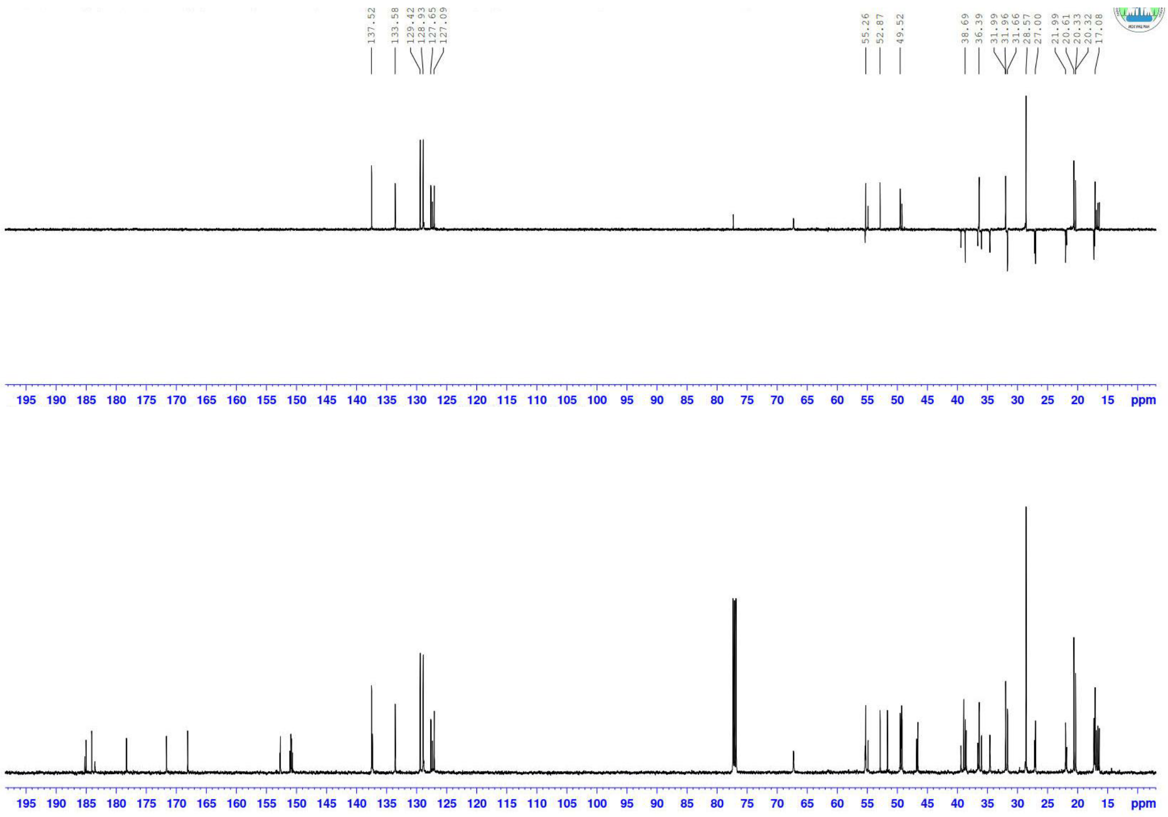

Supplement: Supplementary file 1 [file molecules-29-03532-s001.zip › molecules-3123724-supplementary.pdf]
